# Genome-wide systematic survey and analysis of the RNA helicase gene family and their response to abiotic stress in sweetpotato

**DOI:** 10.1186/s12870-024-04824-z

**Published:** 2024-03-16

**Authors:** Fangfang Mu, Hao Zheng, Qiaorui Zhao, Mingku Zhu, Tingting Dong, Lei Kai, Zongyun Li

**Affiliations:** 1 Jiangsu Key Laboratory of Phylogenomics and Comparative Genomics, School of Life Science, Jiangsu Normal University, Xuzhou, 221116 China; 2 The Key Laboratory of Biotechnology for Medicinal Plants of Jiangsu Province, School of Life Sciences, Jiangsu Normal University, Xuzhou, 221116 China

**Keywords:** Abiotic tress, Genome-wide analysis, RNA helicase, Sweetpotato

## Abstract

Sweetpotato (*Ipomoea batatas* (L.) Lam.) holds a crucial position as one of the staple foods globally, however, its yields are frequently impacted by environmental stresses. In the realm of plant evolution and the response to abiotic stress, the RNA helicase family assumes a significant role. Despite this importance, a comprehensive understanding of the RNA helicase gene family in sweetpotato has been lacking. Therefore, we conducted a comprehensive genome-wide analysis of the sweetpotato RNA helicase family, encompassing aspects such as chromosome distribution, promoter elements, and motif compositions. This study aims to shed light on the intricate mechanisms underlying the stress responses and evolutionary adaptations in sweetpotato, thereby facilitating the development of strategies for enhancing its resilience and productivity. 300 RNA helicase genes were identified in sweetpotato and categorized into three subfamilies, namely IbDEAD, IbDEAH and IbDExDH. The collinearity relationship between the sweetpotato RNA helicase gene and 8 related homologous genes from other species was explored, providing a reliable foundation for further study of the sweetpotato RNA helicase gene family's evolution. Furthermore, through RNA-Seq analysis and qRT-PCR verification, it was observed that the expression of eight RNA helicase genes exhibited significant responsiveness to four abiotic stresses (cold, drought, heat, and salt) across various tissues of ten different sweetpotato varieties. Sweetpotato transgenic lines overexpressing the RNA helicase gene *IbDExDH96* were generated using *A.rhizogenes*-mediated technology. This approach allowed for the preliminary investigation of the role of sweetpotato RNA helicase genes in the response to cold stress. Notably, the promoters of RNA helicase genes contained numerous cis-acting elements associated with temperature, hormone, and light response, highlighting their crucial role in sweetpotato abiotic stress response.

**Supplementary Information:**

The online version contains supplementary material available at 10.1186/s12870-024-04824-z.

## Introduction

RNA helicases are found in all organisms, including prokaryotes and eukaryotes, ranging from viruses to humans [1–3]. They play a crucial role in unwinding double-stranded RNA by utilizing the energy derived from NTP molecules [4]. During RNA metabolism, RNA molecules undergo various modifications [5]. However, their inherent instability can lead to disruptions in RNA metabolism, ultimately impacting the development and ability of plants to withstand abiotic stress [6–8].

Although structurally similar, RNA helicases exhibit diverse functions. These remarkable proteins play crucial roles in multiple biological processes [9], such as RNA splicing, RNA metabolism, ribosome formation, and nuclear-cytoplasmic transport. By actively participating in these intricate molecular mechanisms, RNA helicases contribute significantly to the proper functioning and regulation of cellular activities [10–12]. These genes have many functions in RNA metabolism, among which RNA helicase is associated with resistance to stress. The helicases are divided into 6 superfamilies, namely SF-1, -2, -3, -4, -5, and -6. Among them, the most representative and largest family is SF2. According to the change of DEAD (Asp-Glu-Ala-Asp) motif, the RNA helicase superfamily is DEAD, DEAH and DExD / H, respectively [13, 14]. Almost all the helicase proteins contain nine conserved motifs [15]. Each of these nine motifs plays distinct roles, which are essential for helicase enzymatic activities [16, 17].

RNA helicases are found in numerous plant species, including *Arabidopsis thaliana* [18], *Oryza sativa* [19], *Ipomoea trifida* [20], *Glycine max* [21], *Zea mays* [21], *Gossypium spp* [22], soybean [23], *Gossypium raimondii* [22] and *Solanum lycopersicum* [14]. A total of 32 DEAD were initially identified in *Arabidopsis thaliana* [24]. Then, 113 and 115 RNA helicase genes were identified in *Arabidopsis* and *Oryza sativa* [19]. Studies have shown that RNA helicase genes in *Arabidopsis*, *Oryza sativa*, *Gossypium spp*, *Gossypium raimondii* and *Zea mays* are divided into three subfamilies, the numeral of genes in apiece subfamily is as follows: DEAD-box (50, 51, 87, 51, and 57 genes), DEAH-box (40, 33, 48, 52, and 31 genes), and DExDH-box (71, 65, 78, 58, and 50 genes).

Sweetpotato is a valuable food source and industrial raw material, with high economic importance [25]. Being a hexaploid plant with 90 chromosomes, high heterozygosity, and abundant repetitive sequences, the identification and functional studies of genes in sweetpotato have been challenging [26, 27]. Given that sweetpotato is susceptible to abiotic stress, which significantly affects potato chip production [28], a genome-wide identification of sweetpotato RNA helicase genes is highly warranted. Genomic-assisted breeding technology can potentially improve sweetpotato yield by developing new or improved varieties. To explore the biological basis of cold resistance in sweetpotato, it is essential to identify differentially expressed genes in response to low temperature stress and apply them in production. Proteins and enzymes play critical roles in controlling numerous metabolic pathways by regulating RNA occurrence and metabolism. RNA helicases are involved in various molecular functions, including stress tolerance and development regulation. Therefore, their identification in sweetpotato and efforts to improve sweetpotato varieties hold significant practical value and bear great significance for enhancing the productivity and resilience of this important crop.

Therefore, this study seeks to elucidate the role and involvement pathway of RNA helicase genes in sweetpotato. The main objectives of this study are to conduct a thorough and comprehensive genome-wide validation of RNA helicase genes in sweetpotato. Additionally, we aim to analyze the intricate molecular mechanisms that underlie their active participation in various biological processes specific to sweetpotato. By successfully accomplishing these goals, our study aims to significantly enhance our understanding of the functional significance of RNA helicases in sweetpotato. This newfound knowledge will ultimately contribute to advancements in sweetpotato research and provide valuable insights for crop improvement efforts.

## Materials and methods

### Identification of the RNA helicase genes in sweetpotato genomes

To identify the members of the Arabidopsis RNA helicase gene family, we employed BLASTP to search for all known RNA helicase gene sequences of *Arabidopsis* (113) and rice (115) across multiple databases. Subsequently, utilizing this as a reference, duplicate genes were eliminated, and the DEAD-box RNA helicase family genes of sweetpotato were retrieved [29]. And all the information about the RNA helicase genes in *Arabidopsis* (https://www.arabidopsis.org/) and rice (http://rice.plantbiology.msu.edu/) was downloaded [30]. The whole sweetpotato genome sequence was derivative by *Ipomoea genome Hub* (https://ipomoea-genome.org) [31]. Subsequently, all protein were covered and each member of the RNA helicase gene was verified using the Pfam database (http://pfam.xfam.org/), the CD-search (https://www.ncbi.nlm.nih.gov/cdd/Structure/cdd/wrpsb.cgi), and the PROSITE (https://prosite.expasy.org/), and members lacking typical conserved RNA helicase domains were deleted. The sequence information of all sweetpotato RNA helicase proteins can be found in the Supplementary File 1.

### Phylogenetic relationships of RNA helicase proteins in sweetpotato

The Clustal X program was utilized to compare the sequence of RNA helicase, while the MUSCLE program was employed for multiple sequence alignment to support Clustal X (http://www.clustal.org/) [32]. The maximum likelihood (ML) method was utilized to construct the phylogenetic tree. The optimal evolutionary model LG + G + F was applied to DEAD-box, while the DEAH-box and DExDH-box utilized the optimal evolutionary model WAG + G + F for phylogenetic tree construction. These methodological choices ensure robust and accurate representation of the evolutionary relationships among RNA helicase genes in sweetpotato. Additionally, the bootstrap value was set to 1000 for increased accuracy and reliability of the results [33].

### Protein property and conserved domain of helicase genes in sweetpotato

The physicochemical properties of sweetpotato RNA helicase protein were predicted utilizing the ExPASy online database (http://expasy.org/). To predict the subcellular site and phosphorylation site of RNA helicase genes in sweetpotato, default parameters were utilized for both the Plant-mPLoc (http://www.csbio.sjtu.edu.cn/bioinf/plant-multi/) and NetPhos (http://www.cbs.dtu.dk/services/NetPhos/) databases, respectively. Furthermore, the gene structure of sweetpotato RNA helicase was obtained by comparing its gene sequence with the genome sequence, using Tbtools [34]. Finally, MEME (https://meme-suite.org/meme/tools/meme) was employed to determine the conserved domains [35], and the maximum number of bases was set to 15 in the program settings.

### Chromosomal location and collinearity analysis of the RNA helicase genes in sweetpotato

The isoelectric points and molecular weights of these proteins were determined using ExPASy (http://expasy.org/) [36]. Additionally, the structural characteristics of these sweetpotato RNA helicase genes were analyzed in conjunction with genomic data. To investigate collinearity among RNA helicase genes and other plant genes, the genome sequence information of sweetpotato, *Arabidopsis thaliana*, and rice was downloaded and examined. MCScanX was employed to establish gene duplication and collinearity relationships, utilizing default parameters [37]. The resulting data was visualized using TBtools [34, 38]. Default parameters were utilized throughout all steps of the analysis.

### Plant materials and sample collection

The 11 sweetpotato varieties were selected as plant materials, which were collected and conserved in the Xuzhou Sweetpotato Research Center in China, and no permission was required for plant collection. Stem segments of XuShu18, exhibiting stable growth and measuring about 15 cm in length, were chosen for abiotic stress and hormone treatment, subsequently, the treated leaves were collected to detect the expression of related DEAD-box RNA helicase genes. Young leaves, mature leaves (leaves), stems, and roots were collected from ten sweetpotato varieties (PuShu 32, AoZhouZiBai, LongShu 9, XinXiang, XuZiShu 8, ShangShu 19, JiShu 26, YanShu 25, Marsali, and Hami) during the same period to analyze their tissue-specific expression.

### Expression analyses of the sweetpotato RNA helicase genes

The RNA helicase gene of sweetpotato was analyzed in conjunction with the published transcriptome results under cold treatment on XuShu18 (Xie *et. al* (2019), the supplementary data can be found online at https://doi.org/10.1016/j.ygeno.2018.05.014). XuShu18 was exposed to four abiotic stress treatments and four hormonal stress treatments at time points of 0 h, 1 h, 3 h, 6 h, 12 h, 24 h, 48 h, and 72 h. Four plant tissue parts (YL, L, S, and R) were collected from ten sweetpotato varieties, the objective was to examine the tissue-specific expression of the DEAD-box RNA helicase gene. RNA was extracted from these samples for use in subsequent experiments.

The total RNA was excavated using RNA extraction kit (TianGen, Beijing, China), and reverse transcriptions using TransScript® gDNA removal (TransGen, Beijing, China). All the sweetpotato RNA helicase promoter regions were examined by plantCARE (http://bioinformatics.psb.ugent.be/webtools/plantcare/html/).

### A. rhizogenes-mediated sweetpotato transformation

ZiShu 8 was chosen as the experimental material, and the following methods were employed for mediating penetration. The constructed *IbDExDH96* overexpression vector (OE-IbDExDH96) and the control vector (CK) were introduced into *A.rhizogenes* K599 and cultured in 30 mL LB liquid medium containing 20 mg/L rifampicin and 50 mg/L kanamycin at 28 °C for 12 -16 h. After the OD_600_ value reached between 0.4—0.6, the solution was centrifuged at 4000 rpm for 10 min, and then resuspended in the infiltration buffer. Subsequently, 1 mL of the rhizobium suspension was injected into the stems of sweetpotato cuttings using a needle and syringe. Following this, the cuttings were transplanted into the soil and grown in a high-humidity environment [39]. After 3 weeks of cultivation in the greenhouse, transgenic positive plants were collected for further analysis.

### Statistical analysis

Statistical analysis was conducted using Microsoft Excel 2019, Graphpad Prism 5.0, and SPSS statistical software (IBM Corp, Armonk, NY, USA). In order to ensure the biological significance of the results, a two-fold cut-off value was applied for differential gene expression analysis. The data were subjected to ANOVA (one-way analysis of variance) using the SPSS software, and differences in means were considered significant if they had a *p*-value < 0.05, as determined by Dunnett's test.

## Results

### Identification of RNA helicase family genes in sweetpotato

To identify the components of the sweetpotato RNA helicase gene, we utilized bioinformatics methods to gather information on numerous sweetpotato RNA helicases. A total of 300 sweetpotato RNA helicase proteins were identified. Based on conserved motifs within the RNA helicases, the 300 sweetpotato RNA helicase genes were categorized into the DEAD-box subfamily (53 genes), the DEAH-box subfamily (54 genes), and the DExDH-box subfamily (193 genes) (Supplementary File 1 and Supplementary Fig. 1). The amount of RNA helicase proteins in *Arabidopsis* and rice was 113 and 115, respectively. It is predicted that there are 300 RNA helicase proteins in sweetpotato, which is much more than the number of *Arabidopsis* and rice [19]. Subsequently, the 300 RNA helicase genes located on the 15 sweetpotato chromosomes were systematically named from top to bottom as *IbDEAD1* ~ *IbDEAD53*, *IbDEAH1* ~ *IbDEAH54*, and *IbDExDH1* ~ *IbDExDH193*, following a consistent nomenclature convention (Supplementary Fig. 2). Subsequently, the physicochemical properties of 300 sweetpotato RNA helicase proteins were examined. The length and relative molecular mass of the RNA helicase vary greatly. The length of DEAD ranges from 323 aa (IbDEAD34) to 1301 aa (IbDEAD2), the relative molecular mass ranges from 3584.09 to 145,261.6 Da, and the isoelectric point ranges from 5.02 (IbDEAD40) to 9.87 (IbDEAD37). The length of DEAH is between 206 aa (IbDEAH5) and 2904 aa (IbDEAH44), the relative molecular mass is between 23,377.03 and 323,370.3 Da, and the isoelectric point is between 5.16 (IbDEAH22) and 9.25 (IbDEAH36). The length of DExDH ranged from 128aa (IbDExDH7) to 2801aa (IbDExDH30), the relative molecular mass ranged from 13,904.77 to 306,450.01 Da, and the isoelectric point graded from 4.81 (IbDExDH7) to 9.67 (IbDExDH45). The subcellular location showed that most of the RNA helicase proteins were positioned in the nucleus. Furthermore, the potential phosphorylation sites showed that IbDEAD contains 29 (IbDEAD28) to 141 (IbDEAD2) phosphorylation sites, IbDEAH contains 19 (IbDEAH5) to 280 (IbDEAH44) phosphorylation sites, and IbDExDH contains 18 (IbDExDH141) to 362 (IbDExDH30) phosphorylation sites, all of these sweetpotato RNA helicase proteins restrain more Ser sites than the Tyr and Thr sites (Supplementary Table 1).

### Phylogenetic analysis of the RNA helicase family proteins in sweetpotato

**Fig. 1:**
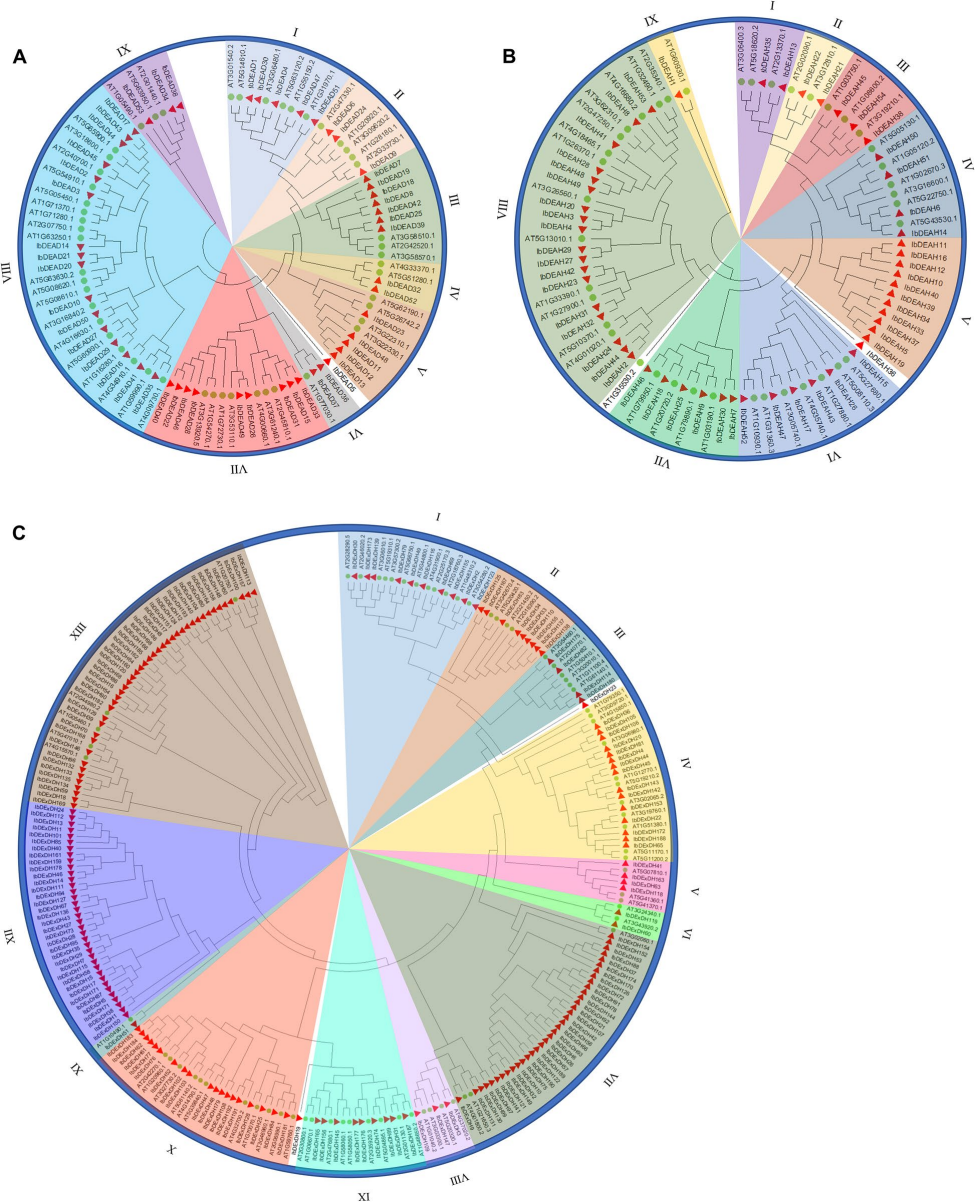
Phylogenetic tree analysis of RNA helicase in *Arabidopsis* and sweetpotato. **A** The DEAD-box RNA helicase proteins in two species. **B** The DEAH-box RNA helicase proteins in two species. **C** The DExDH-box RNA helicase proteins in two species. These three subfamilies were used to construct the maximum-likelihood (ML) phylogenetic tree by MEGA X with 1,000 bootstrap replicates. Different subgroups are named based on the reports in *Arabidopsis* and are distinguished with different colors. The gene names are marked at the end of the branch, the red triangle and green circle represent the sweetpotato RNA helicase and Arabidopsis RNA helicase, respectively

To investigate the evolutionary patterns and classification of RNA helicases in sweetpotato, we constructed a rootless phylogenetic tree, comparing the identified RNA helicases in sweetpotato with the known RNA helicases in *Arabidopsis thaliana* (Fig. 1). The RNA helicases in Arabidopsis were classified into three subfamilies: DEAD, DEAH, and DExDH. Among the 53 IbDEAD proteins, except for IbDEAD5, they were further divided into nine subgroups. Similarly, the 54 IbDEAH proteins, excluding IbDEAH36, were separated into nine subgroups. Furthermore, the 193 IbDExDH proteins were categorized into 13 subgroups, with the exception of IbDExDH19 and IbDExDH23. Notably, the V subgroup in IbDEAH and the XII subgroup in IbDExDH were unique to sweetpotato. The distribution of RNA helicase proteins varied across the different subfamilies. Subgroups VI, VIII, and XI of IbDExDH contained the fewest sweetpotato RNA helicase genes. Intriguingly, IbDEAD5, IbDEAH36, IbDExDH19, and IbDExDH23 did not belong to any of the aforementioned three subfamilies, suggesting that they might possess alternative functions.

### Gene structure and motif composition analyses in sweetpotato

**Fig. 2:**
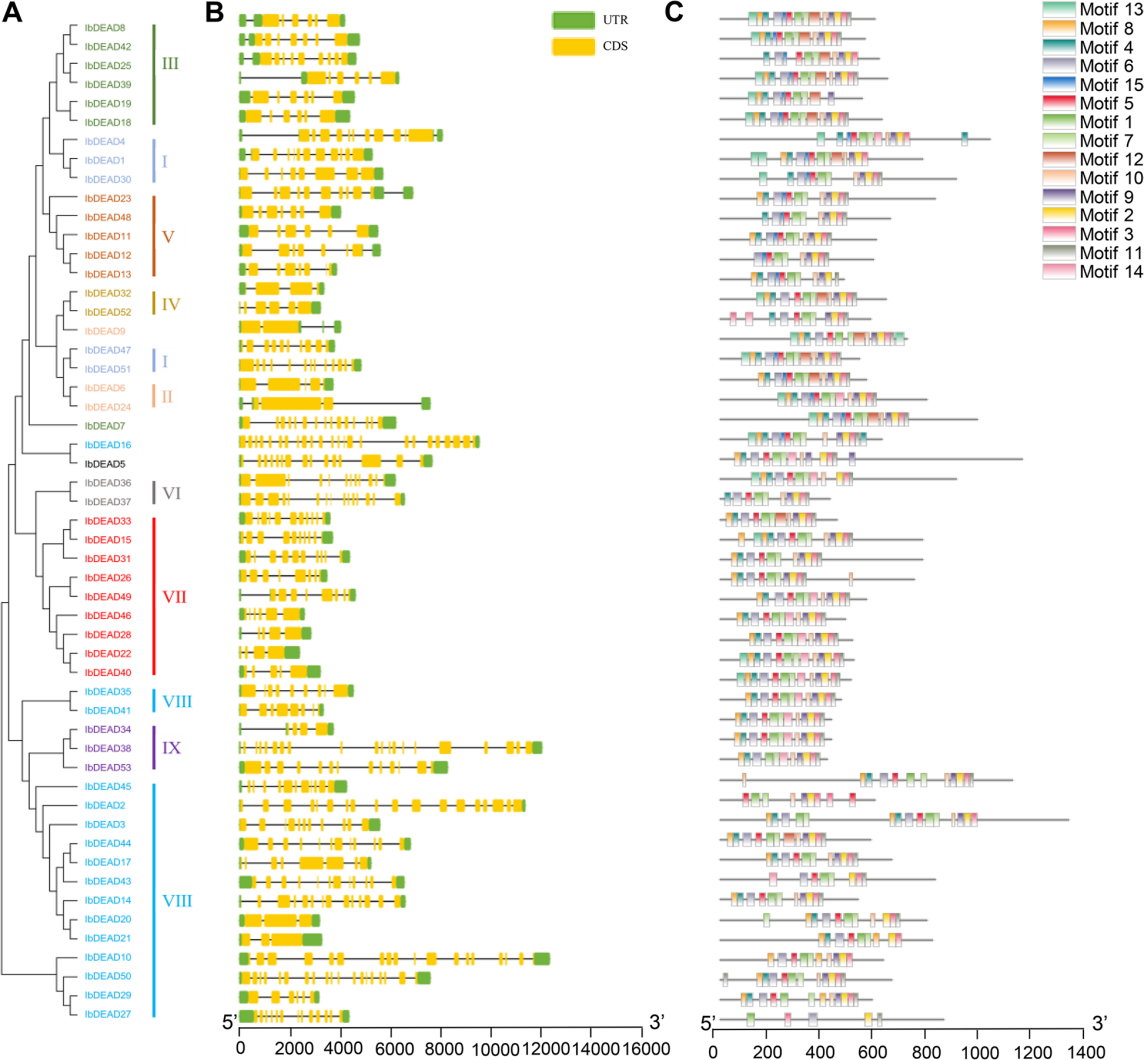
Phylogenetic tree, gene structure and motif distribution analysis of 53 IbDEAD-box members. **A** Phylogenetic tree of DEAD-box members in sweetpotato. A total of 53 IbDEAD-box proteins were used to construct the maximum-likelihood (ML) phylogenetic tree by MEGA X with 1,000 bootstrap replicates. The different groups are marked with different colors. **B** The exon–intron structure analysis of 53 IbDEAD-box genes. Exon and introns lengths are displayed proportionally, yellow boxes represent exons, black lines represent introns, and green boxes represent 5’ and 3’ UTR. **C** Motif distribution of 53 IbDEAD-box proteins. The different colored boxes represent 15 motifs, and the box lengths represent motif lengths

**Fig. 3:**
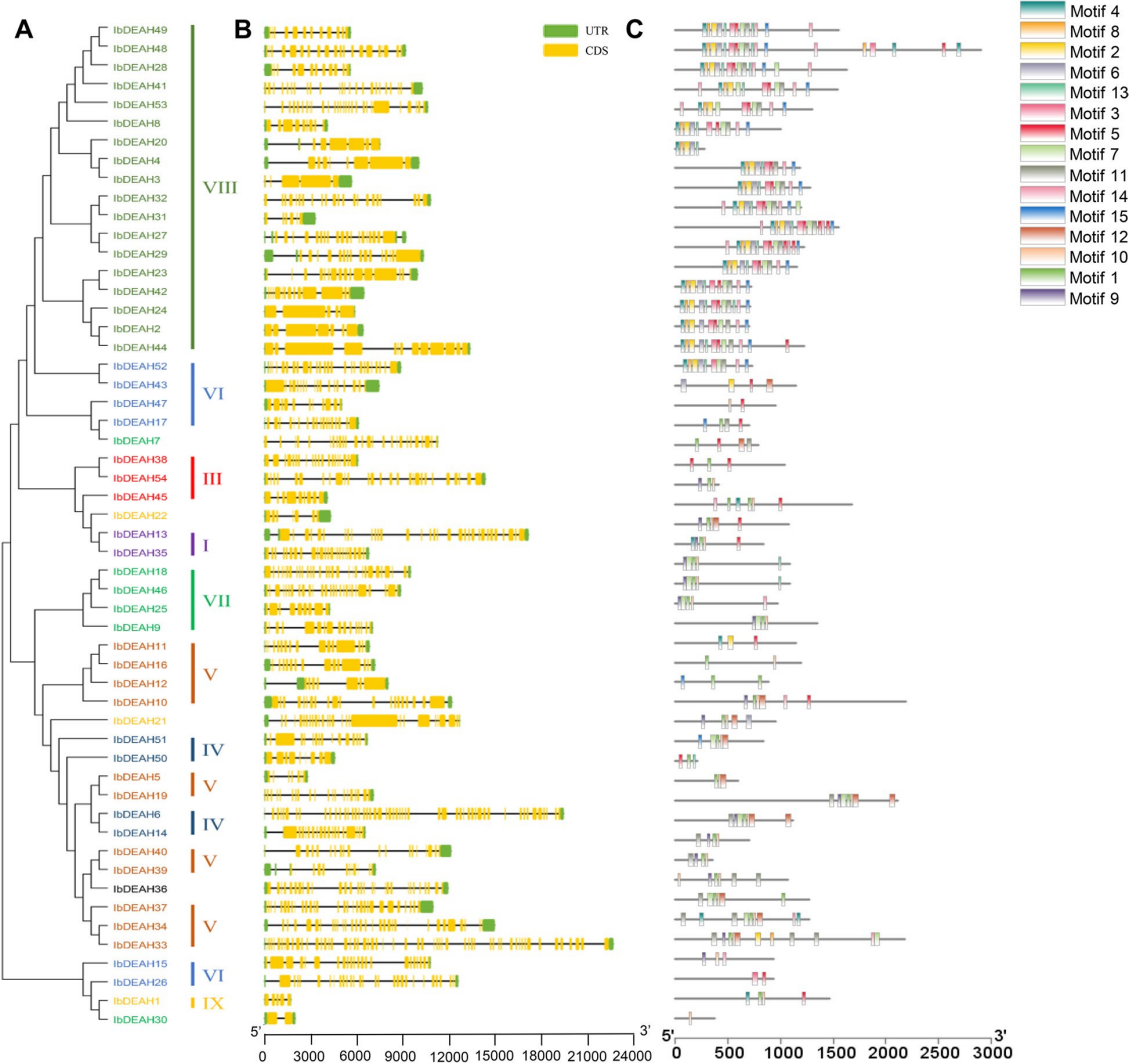
Phylogenetic tree, gene structure and motif distribution analysis of 54 IbDEAH-box members. **A** Phylogenetic tree of DEAH-box members in sweetpotato and the consistent parameters shown in Fig. 2(A). The different groups are marked with different colors. **B** The exon–intron structure analysis of 54 IbDEAH-box genes and the consistent parameters shown in Fig. 2(B). **C** Motif distribution of 54 IbDEAH-box proteins. The different colored boxes represent 15 motifs, and the box lengths represent motif lengths

**Fig. 4:**
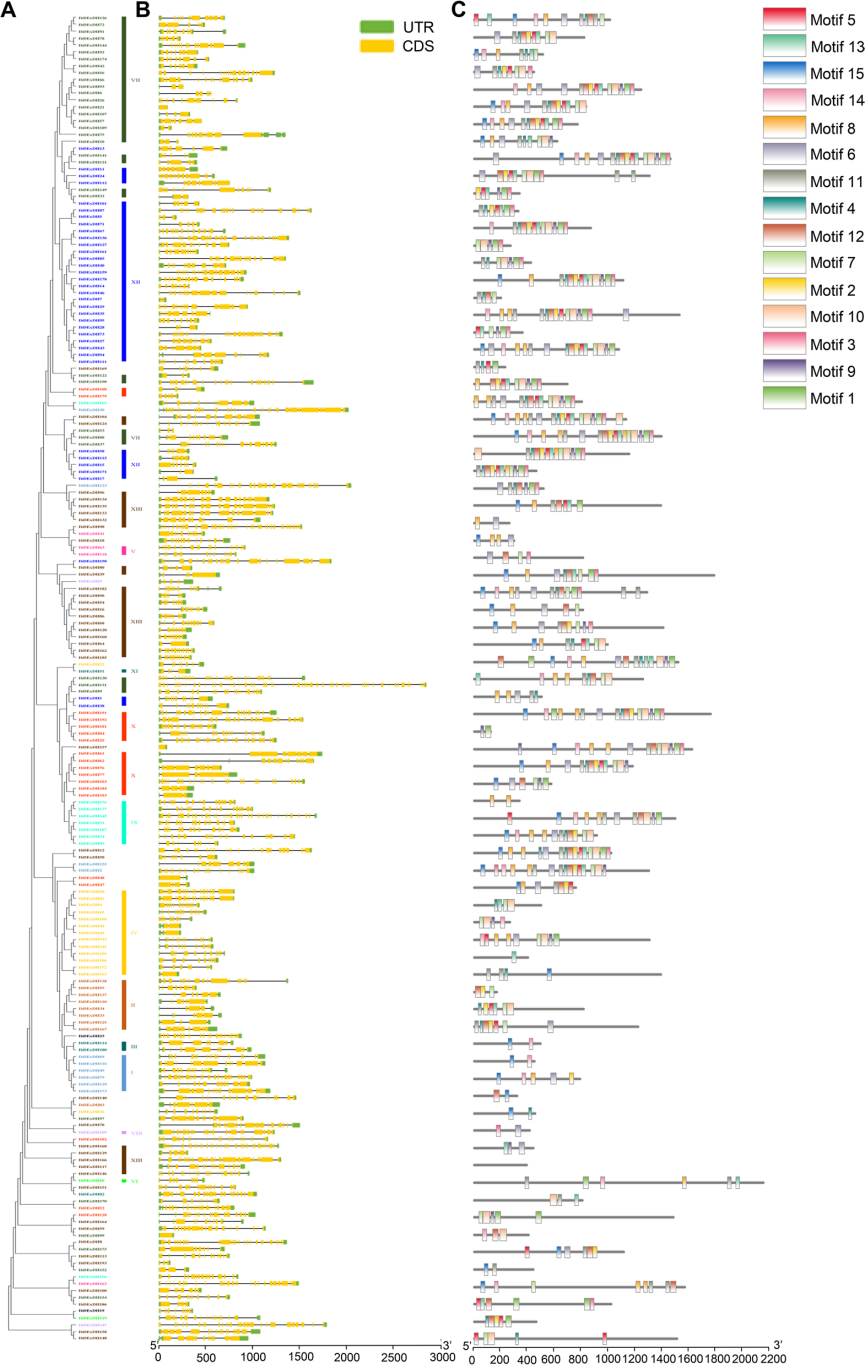
Phylogenetic tree, gene structure and motif distribution analysis of 193 IbDExDH-box members. **A** Phylogenetic tree of DExDH-box members in sweetpotato and the consistent parameters shown in Fig. 2(A). The different groups are marked with different colors. **B** The exon–intron structure analysis of 193 IbDExDH-box genes and the consistent parameters shown in Fig. 2(B). **C** Motif distribution of 103 IbDExDH-box proteins. The different colored boxes represent 15 motifs, and the box lengths represent motif lengths

To gain further insights into the structural characteristics of the sweetpotato RNA helicase family, we conducted a comparative analysis of conserved motifs and intron/exon composition based on the phylogenetic tree of sweetpotato RNA helicases (Figs. 2 and 4). The structural features of IbDEAD, IbDEAH, and IbDExDH subfamily genes exhibit notable variations across different subgroups. However, it is worth mentioning that genes within the same side branch are homologous, sharing similar genetic structures. This observation suggests a strong correlation between the exon–intron structure and phylogenetic relationships within the sweetpotato RNA helicase family (Figs. 2, 3 and 4). The genetic architecture of the RNA helicase family members in sweetpotato is remarkably intricate, with the *IbDEAD* and *IbDEAH* gene members exhibiting a higher number of exons. In contrast, among the *IbDExDH* gene members, only a small fraction of genes (2.6%) were found to comprise a single exon, while the rest were composed of multiple exons. To gain further insight into the structural conservation of these RNA helicases, we performed motif analysis for each member using the MEME website, revealing the presence of 15 highly conserved motifs across all sweetpotato RNA helicases. This finding underscores the potential functional significance of these motifs in regulating RNA helicase activity in sweetpotato (Figs. 2, 3 and 4). Sweetpotato RNA helicases exhibit 15 conserved motifs, with motifs 1, 2, 3, 4, 5, 7, 8, 9, and 15 being widely present in most proteins. Motif 5 is particularly noteworthy because it contains highly conserved sequences, including DEAD, DEAH, and DExDH, which are essential for RNA helicase function. Although these conserved motifs are shared among sweetpotato RNA helicases, significant variations exist in their domains and amino acid sequences. These differences suggest that despite having common structural features, sweetpotato RNA helicases may have diverse functions and regulatory mechanisms (Figs. 2, 3 and 4). In general, phylogenetic tree examination of data shows that the system development and features and the divergence of genetic structure and sequence distribution are closely related.

### Chromosome localization of the RNA helicase family in sweetpotato

**Fig. 5:**
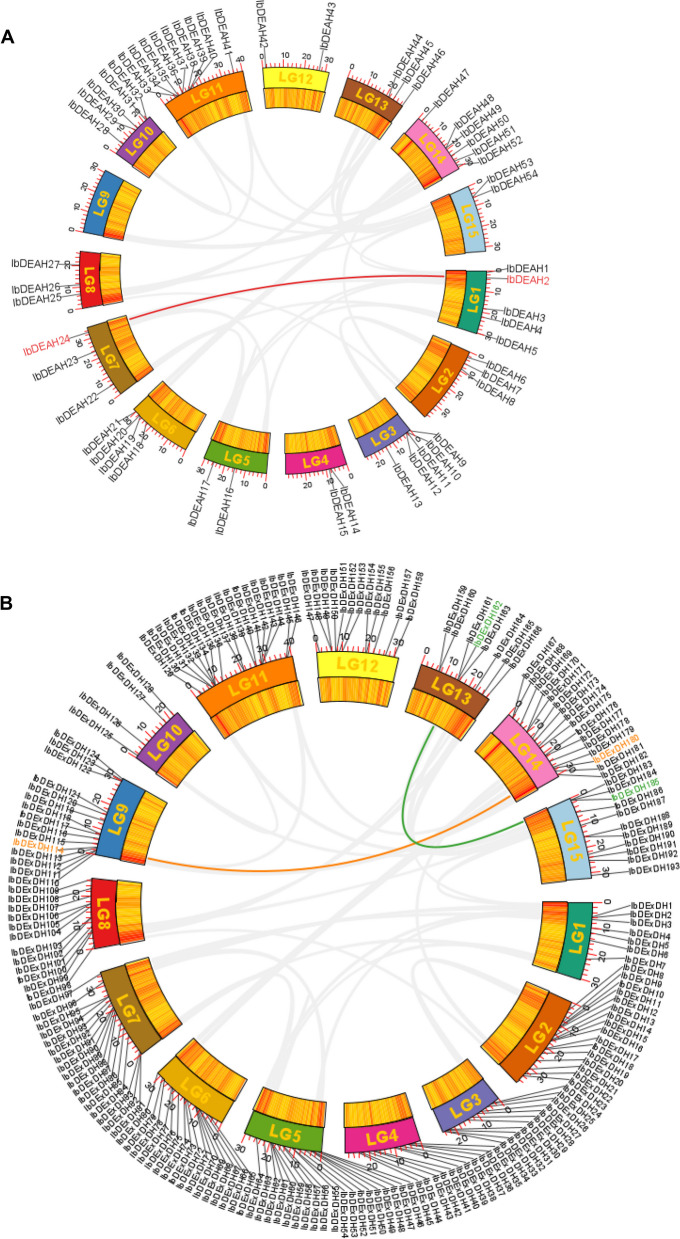
The interchromosomal relationship of RNA helicase genes in sweetpotato chromosomes. **A** IbDEAH, **B** IbDExDH. Circular visualizations of RNA helicase genes mapped to the LG1- LG15 chromosomes are indicated by colored rectangles. The colored curves represent duplicated RNA helicase gene pairs. The corresponding RNA helicase genes located in segmental duplications are marked with colors

Using GFF3 genome annotation, physical location detection analysis revealed that 53 *IbDEAD* genes, 54 *IbDEAH* genes, and 193 *IbDExDH* genes were distributed across all 15 sweetpotato chromosomes. Notably, chromosome 6 harbored the highest number of *IbDEAD* genes, with 7 members, while chromosome 11 contained the largest number of *IbDEAH* genes, with 9 members. Strikingly, chromosome 9 had no *IbDEAD* or *IbDEAH* genes. Furthermore, most chromosomes exhibited a high abundance of *IbDExDH* genes, with the exception of chromosome 10, which contained only four *IbDExDH* genes. These findings shed light on the genomic distribution of RNA helicases in sweetpotato, suggesting potential functional diversification among different gene family members, and providing a foundation for further investigations into their biological roles and regulatory mechanisms (Figs. 5 and Supplementary Fig. 3). The distribution of the three subfamilies of RNA helicases across the 15 chromosomes exhibits notable divergence. Collinearity analysis revealed a cluster of tandem duplicated *IbDEAH* genes and two clusters of tandem duplicated IbDExDH genes, specifically *IbDEAH2 / 24* and *IbDExDH114 / 168* as well as *IbDExDH162 / 185*. However, no tandem duplicated genes were identified in the IbDEAD subfamily (Supplementary Table 7). These findings provide insights into the evolutionary dynamics and genomic organization of RNA helicase genes in sweetpotato, suggesting potential mechanisms underlying their expansion and functional diversification.

### Cis element analysis of the RNA helicase family gene promoters

**Fig. 6:**
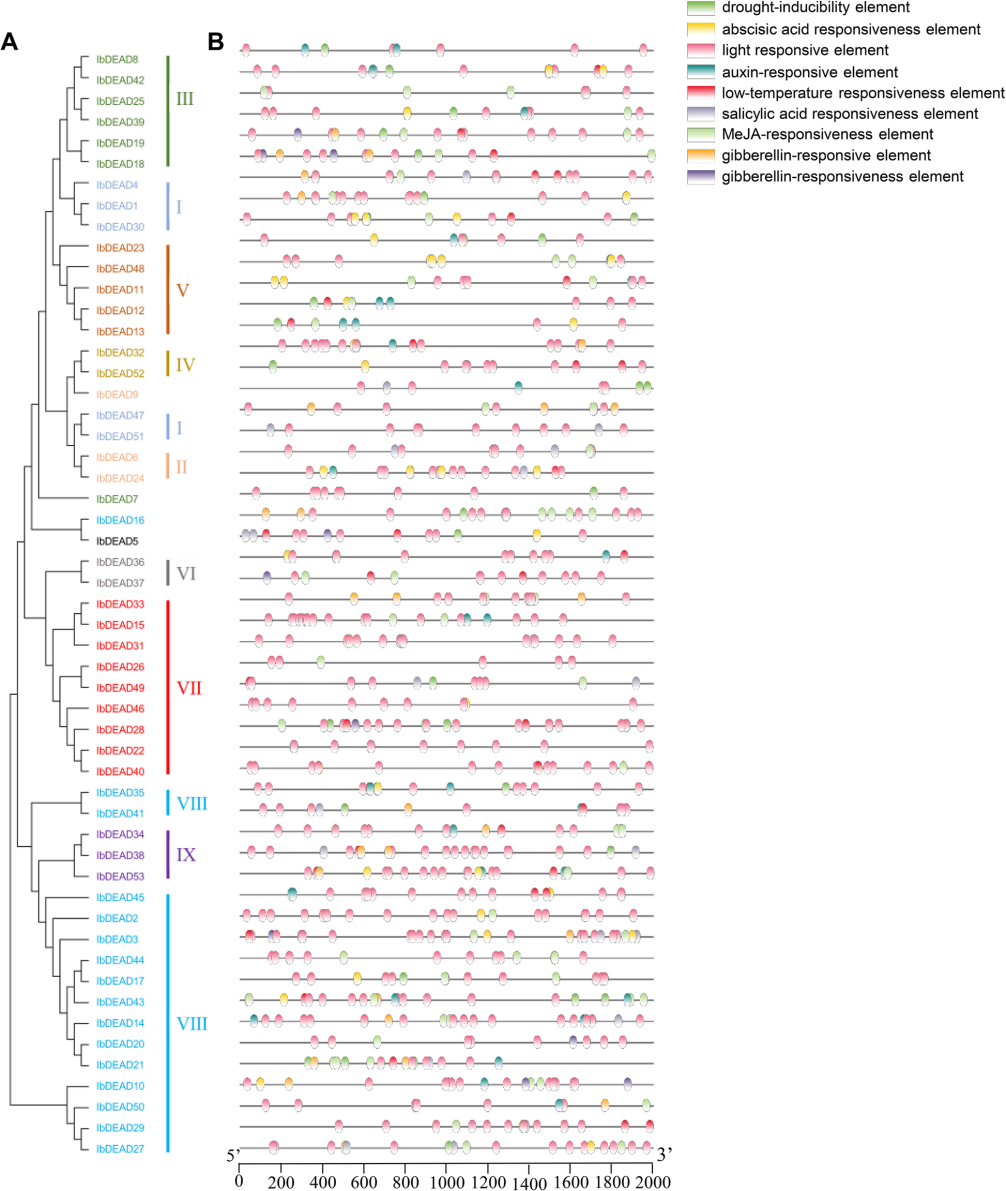
Phylogenetic clustering and predicted stress- and hormone-related cis-elements in the promoters of *IbDEAD* genes. **A** The phylogenetic tree of 53 IbDEADs was constructed by MEGA X based on the consistent parameters used in Fig. 2(A). **B** Predicted cis-elements in the IbDEAD promoters. 2000 bp promoter regions of each *IbDEAD* gene were detected by PlantCARE database. Different colored rectangles represent different cis-elements that are potentially involved in stress or hormone regulation

**Fig. 7:**
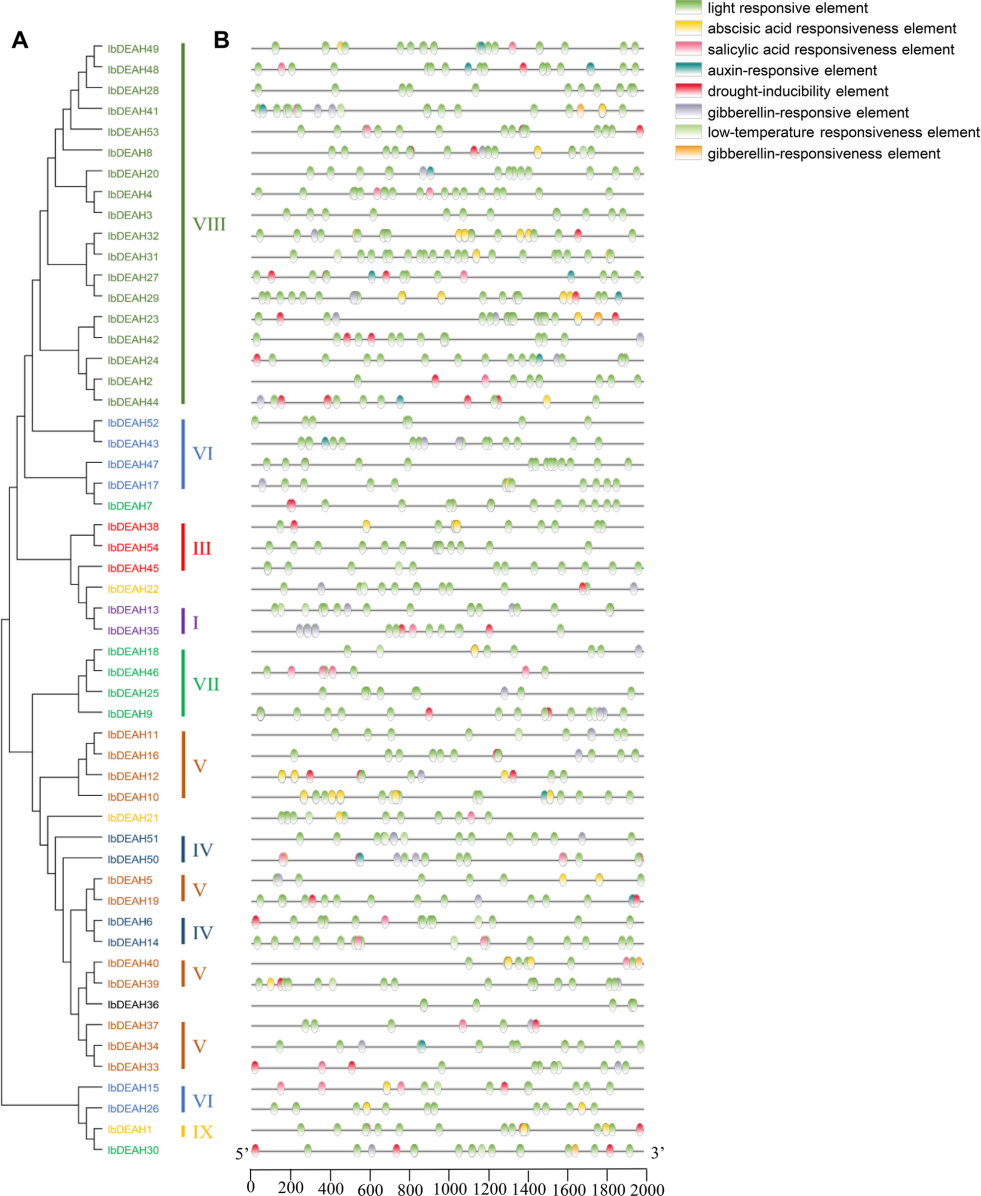
Phylogenetic clustering and predicted stress- and hormone-related cis-elements in the promoters of *IbDEAH* genes. **A** The phylogenetic tree of 54 species of IbDEAH and the consistent parameters shown in Fig. 2(A). **B** Predicted cis-elements in the IbDEAH promoters. Different colored rectangles represent different cis-elements that are potentially involved in stress or hormone regulation

**Fig. 8:**
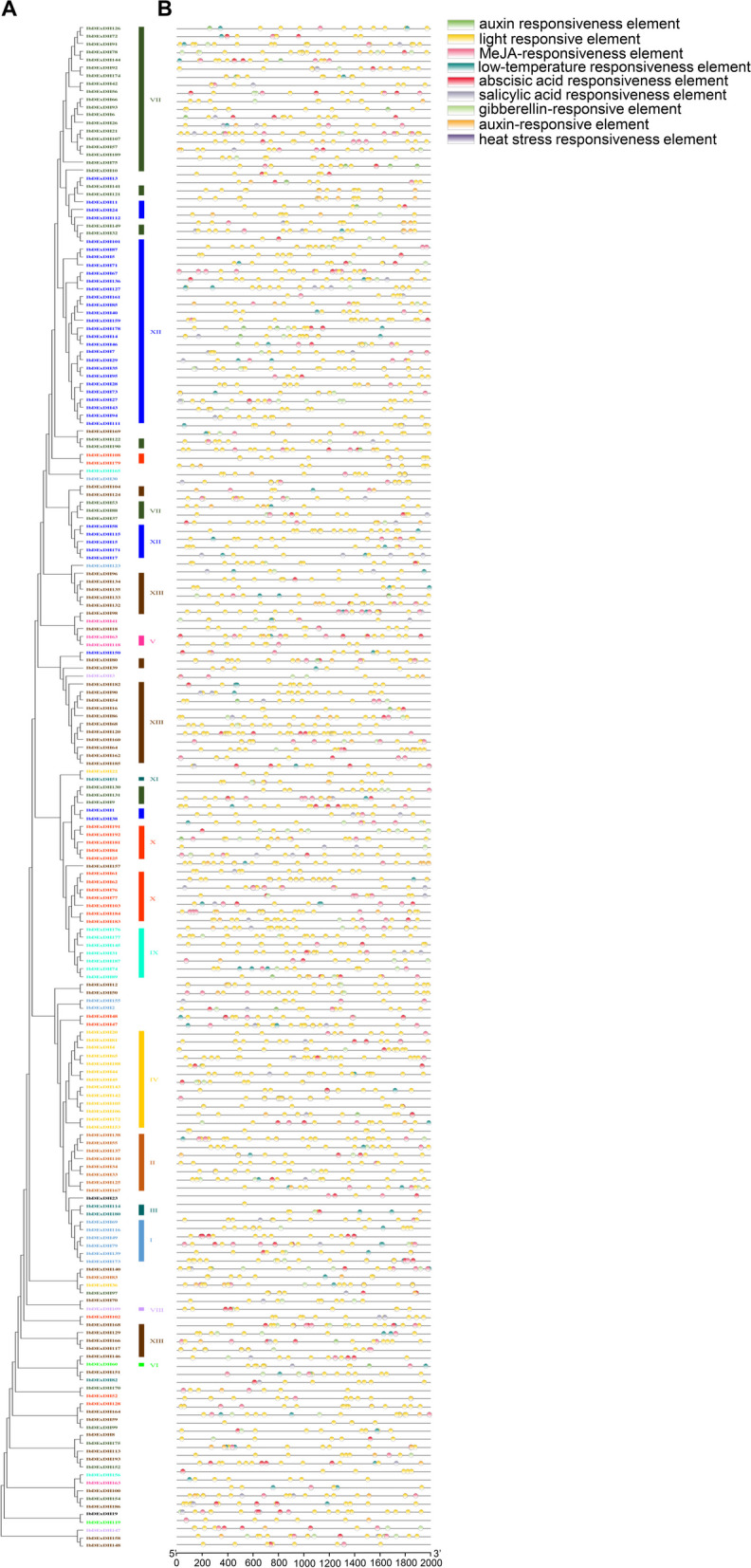
Phylogenetic clustering and predicted stress- and hormone-related cis-elements in the promoters of *IbDExDH* genes. **A** The phylogenetic tree of 193 species of IbDExDH and the consistent parameters shown in Fig. 2(A). **B** Predicted cis-elements in the IbDExDH promoters. Different colored rectangles represent different cis-elements that are potentially involved in stress or hormone regulation

To investigate the potential regulatory mechanisms of sweetpotato RNA helicases in response to abiotic stress and hormones, we conducted a comprehensive analysis by scanning the cis-acting elements present in the 2 kb upstream promoter region of these genes. This analysis was performed using the PlantCare database, which provides valuable insights into the putative regulatory elements involved in the modulation of gene expression (Figs. 6, 7 and 8, Supplementary Table 2).

**Fig. 9:**
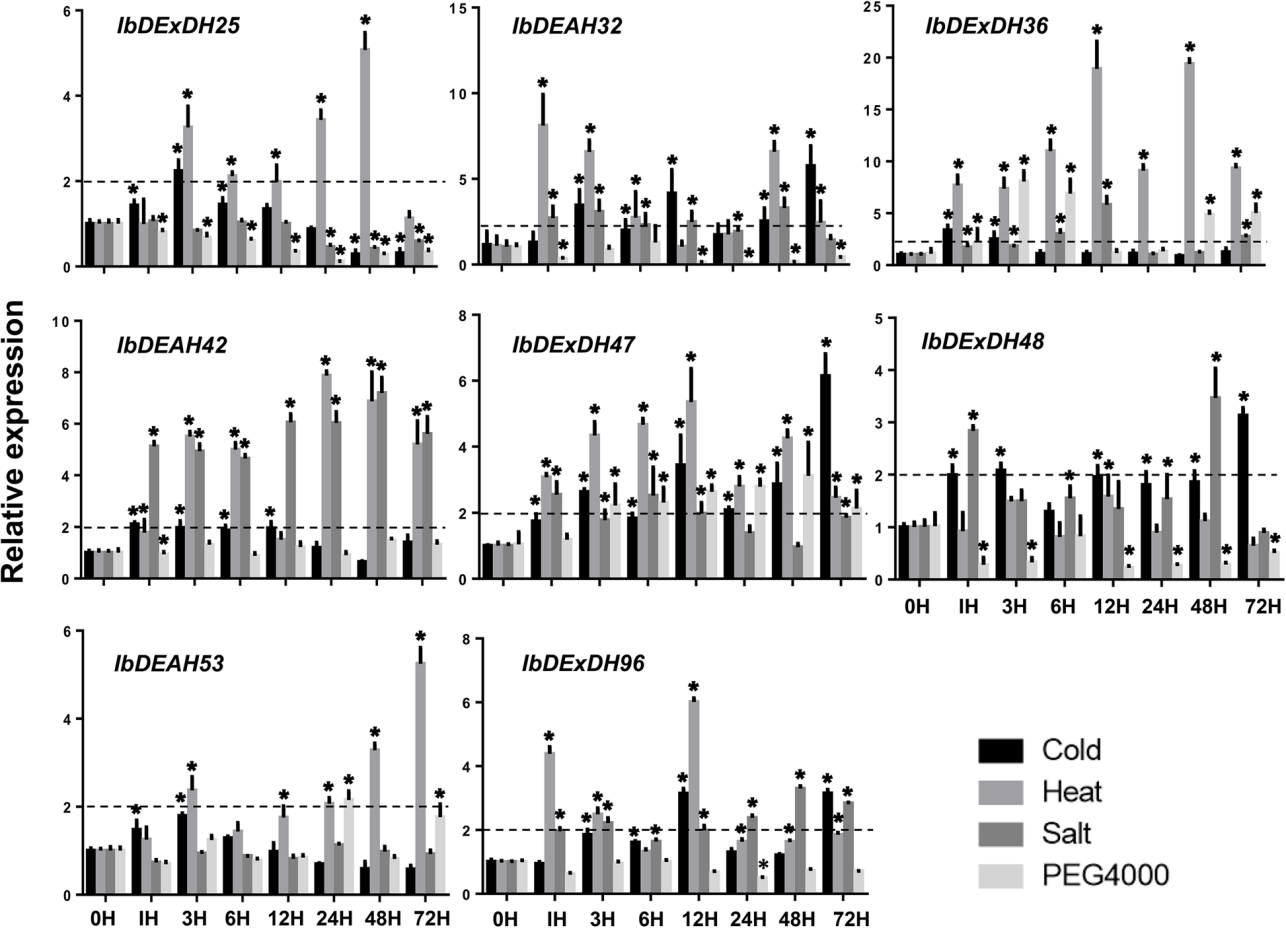
Relative expression levels of 8 RNA helicase genes in response to abiotic stresses detected by qRT-PCR. The abiotic stress treatments include cold (4 °C), heat (42 °C), salt (200 mM NaCl) and drought (200 mM PEG4000). The expression levels at 0 h were normalized to 1, and the Y-axis delineates the fold changes of relative expression comparing with 0 h. Bars represent the mean of three biological replicates ± SE. The two-fold threshold is presented by a dotted line. Values are presented relative to untreated plants (0 h). Asterisks indicate a significant difference (*P* < 0.05) between untreated and treated plants

**Fig. 10:**
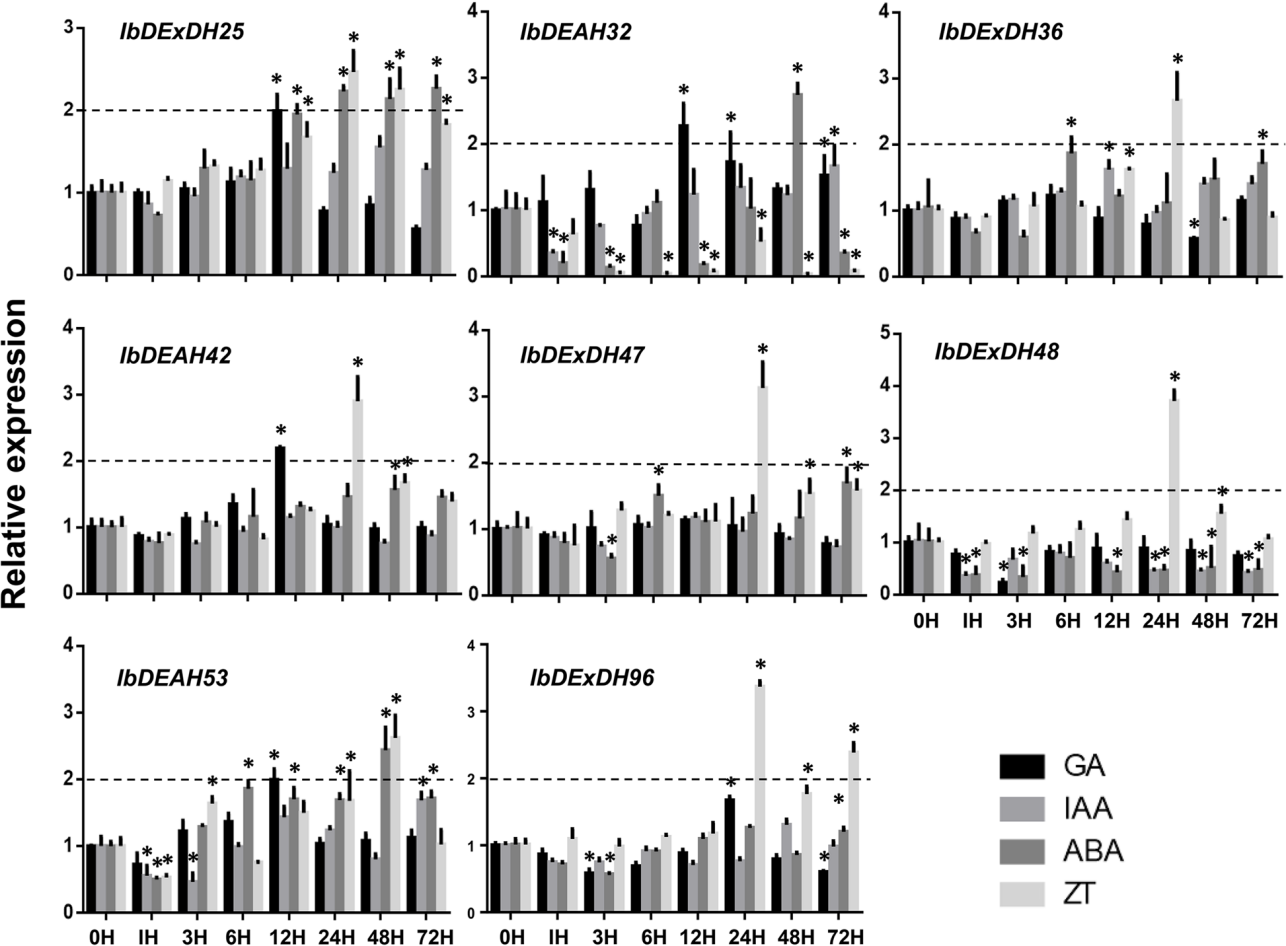
Relative expression levels detected by qRT-PCR under different hormone treatments including GA, IAA, ABA and ZT. The expression levels at 0 h were normalized to 1, and the Y-axis delineates the fold changes of relative expression comparing with 0 h. Bars represent the mean of three biological replicates ± SE. The two-fold threshold is presented by a dotted line. Values are presented relative to untreated plants (0 h). Asterisks indicate a significant difference (*P* < 0.05) between untreated and treated plants

The conclusions showed that each promoter region of RNA helicase had multiple cis-acting elements connected with stress- and / or hormones. Among others, nearly 90% of RNA helicase promoters contain multiple stress cis-elements, for instance stress response elements (TC-rich repeats), low temperature response elements (LTR), MAJA response elements (TGACG-motif), drought response elements (MBS), etc. These cis-acting elements can be associated with expression. For example, the expression of several RNA helicase genes providing *IbDEAH-32 / -42* and *IbDExDH-36 / -47 / -96* was increased under different stresses. Correspondingly, stress-related repeats of MBS, TC, or LTR cis elements are more numerous in their promoter regions. However, TC-rich repeats, MAJA response elements and LTR elements were found on the promoters of the *IbDExDH-25* / *-48* and *IbDEAH53* genes, their expression was not particularly significant under salt, drought, or cold stress, but there was a certain degree of response under high-temperature stress (Fig. 9 and Supplementary Table 5). Furthermore, all sweetpotato RNA helicase promoters restrain many hormone elements, such as abscisic acid response element (ABRE), Me-JA response element (CGTCA motif and TGACG motif) or auxin response element (TGA-box) (Figs. 6, 7 and 8, Supplementary Table 2). Nevertheless, the expression of the RNA helicase genes in dissimilar tissues of 10 different sweetpotato varieties was also different (Fig. 14). Most of the RNA helicase genes are communicated in high amounts in sweetpotato stems and root tissues, in particular, the expression levels of *IbDExDH36* and *IbDExDH48* in the roots were lower than those in other tissues. It shows that RNA helicase is associated to plant development. Among them, the transcription levels of most RNA helicase genes do not respond significantly to hormonal treatment (Fig. 10). These data indicate that the cis-acting elements of sweetpotato RNA helicase can be concerned in both hormonal and abiotic stresses.

### Identification of RNA helicase family cold-response genes in transcriptomes and their expression profiles under multiple abiotic stress and hormonal treatments

Numerous studies have demonstrated the significant involvement of RNA helicases in diverse abiotic stresses, including cold, salt, drought, and heat, as well as developmental processes. In order to elucidate the potential biological role of the sweetpotato RNA helicase gene under adverse stress conditions, we conducted a comprehensive analysis using our previously generated RNA-seq data. Specifically, we focused on investigating the expression pattern of the XuShu 18 RNA helicase gene under cold stress [40]. The consequence indicated that eight RNA helicase genes were screened out. Subsequently, we further studied the expression patterns of eight genes (*IbDEAH-32 / -42 / -53*, *IbDExDH-25 / -36 / -47 / -48 / -96*) screened by qRT-PCR under salt, drought, heat and cold stresses, and explore a two-fold cut off value [41]. The results showed that all selected genes were up-regulated to varying degrees after salt, drought, cold, and heat treatment. Among them, four stresses could up-regulate *IbDEAH32* and *IbDExDH-36 / -47* transcription, two stresses could up-regulate *IbDEAH42* and *IbDExDH-48 / -96* expression, and one stress could up-regulate *IbDExDH25* and *IbDEAH53* transcription. All RNA helicases could be up-regulated under high temperature stress and salt stress. In particular, *IbDEAH32* and *IbDExDH47* had the highest level of induction after cold treatment, about six times, while the level of induction was relatively low in the transcription of other RNA helicases. Except for *IbDExDH48*, the expression of other genes was significantly enhanced under high temperature conditions, which was 2.3 and 19.3-fold that of the control. Under salt stress conditions, the expression of *IbDEAH42* and *IbDExDH-47 / -48* was enhanced, and the expression of other genes was weakly induced by salt stress. In particular, the response of all genes to drought stress was not very strong, which may be affected by other factors (Fig. 9). Overall, these data suggest that multiple sweetpotato RNA helicase members can take the lead in reaction to abiotic stress.

Moreover, qRT-PCR was acclimated to further detect the transcription profiles of eight RNA helicase genes under distinct hormone treatments, encircling ABA, IAA, GA and ZT. Unexpectedly, most of the RNA helicase genes were down-regulated when we used double as the cut-off value for differential expression. Just the stress hormone ZT could prompt the expression of *IbDExDH-36 / -47 / -48 / -98* and *IbDEAH42* (Fig. 10). It is reported that the response of the RNA helicase to hormone treatment is not obvious, mainly related to development and abiotic stress [42, 43]. Overall, these data propose that multiple members of the sweetpotato RNA helicase can be important players in answer to hormone and / or abiotic stresses.

### Collinearity analysis of the RNA helicase genes between sweetpotato and other plants

**Fig. 11:**
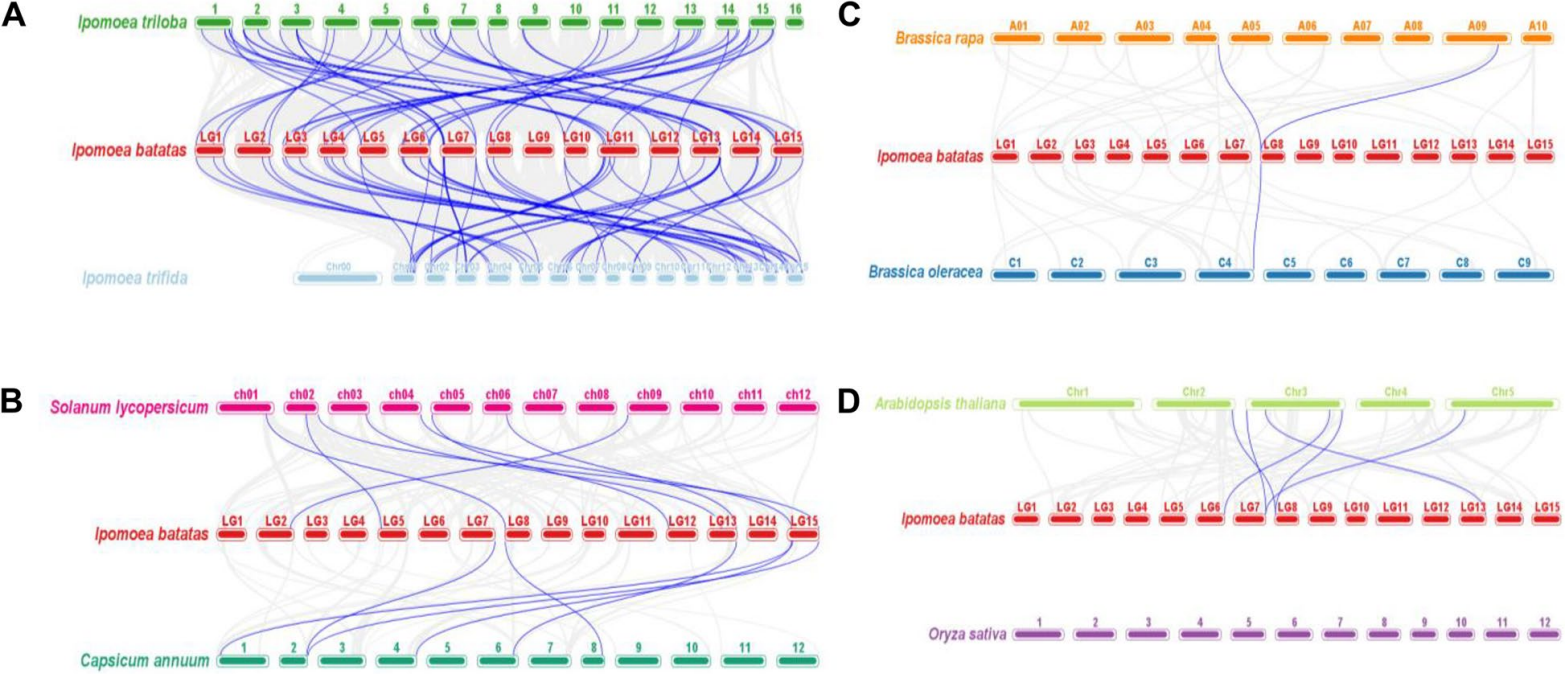
Synteny analyses of *IbDEAD* genes between sweetpotato and eight representative plant species from *Ipomoea triloba* and *Ipomoea trifida* **A** *Solanum lycopersicum* and *Capsicum annuum* **B** *Brassica rapa* and *Brassica oleracea* **C** and *Arabidopsis thaliana* and *Oryza sativa* **D** The chromosomes of different plants are distinguished with differential colors. The blue lines connecting two different chromosomes highlight the syntenic *IbDEAD* gene pairs within sweetpotato and other plant genomes, respectively

**Fig. 12:**
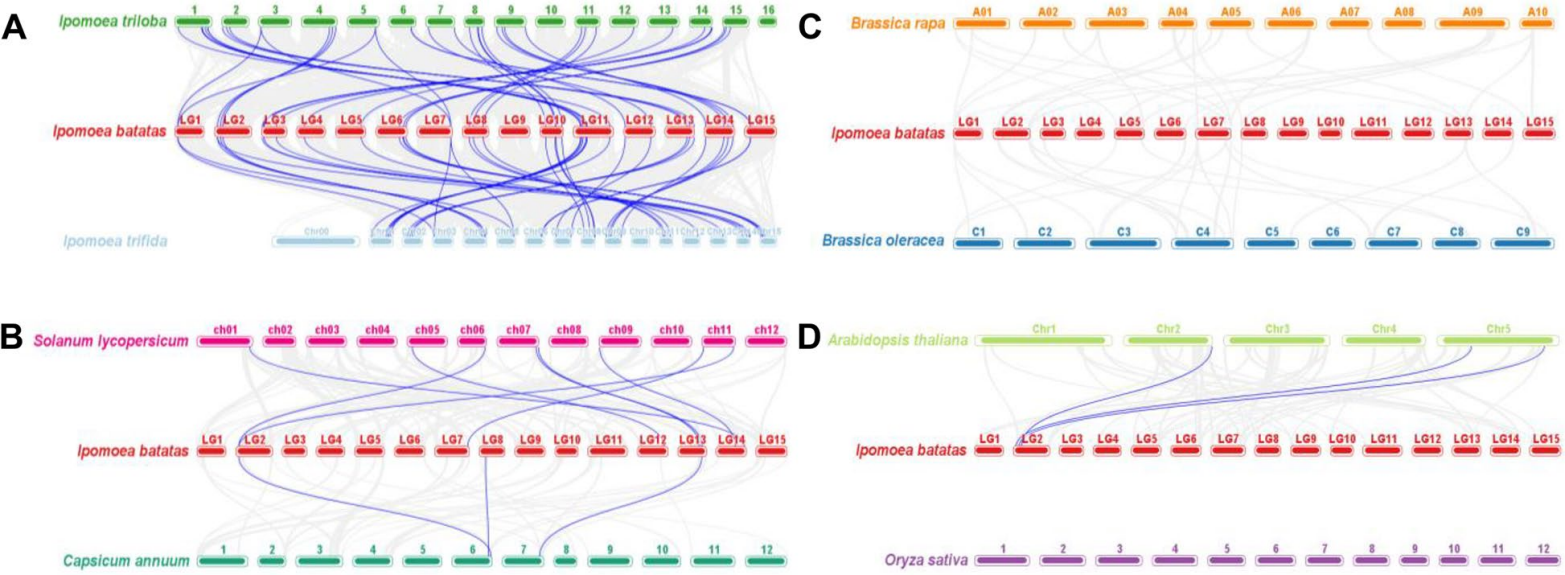
Synteny analyses of *IbDEAH* genes between sweetpotato and eight representative plant species from *Ipomoea triloba* and *Ipomoea trifida* **A** *Solanum lycopersicum* and *Capsicum annuum* **B** *Brassica rapa* and *Brassica oleracea* **C** and *Arabidopsis thaliana* and *Oryza sativa* **D** The chromosomes of different plants are distinguished with differential colors. The blue lines connecting two different chromosomes highlight the syntenic *IbDEAH* gene pairs within sweetpotato and other plant genomes, respectively

**Fig. 13:**
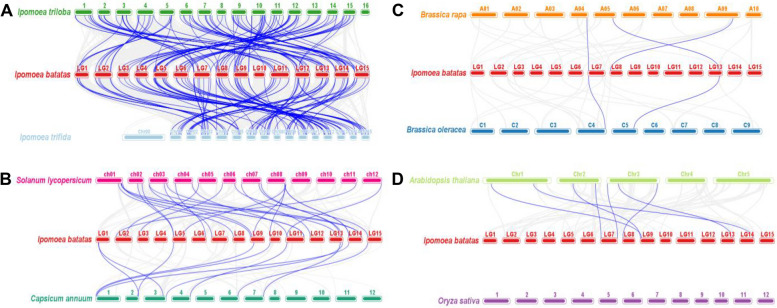
Synteny analyses of *IbDExDH* genes between sweetpotato and eight representative plant species from *Ipomoea triloba* and *Ipomoea trifida* **A** *Solanum lycopersicum* and *Capsicum annuum* **B** *Brassica rapa* and *Brassica oleracea* **C** and *Arabidopsis thaliana* and *Oryza sativa* **D** The chromosomes of different plants are distinguished with differential colors. The blue lines connecting two different chromosomes highlight the syntenic *IbDExDH* gene pairs within sweetpotato and other plant genomes, respectively

To furthermore explore the origin and evolutionary mechanism of sweetpotato RNA helicase genes, we compared the homology of 300 RNA helicase genes with 8 representative species-related genes. These species include wild diploid relatives of sweetpotato (*Ipomoea trioba* and *Ipomoea trifida*), two model plants (*Arabidopsis thaliana* and *Oryza sativa*), two cruciferous plants (*Brassica rapa* and *Brassica oleracea*) and two Solanaceae plants (*Solanum lycopersicum* and *Capsicum annuum*). Among them, 42 (79.2%) and 43 (81.1%) *IbDEAD* genes were homologous to genes in *Ipomoea trioba* and *Ipomoea trifida*, respectively, accompanied by *Solanum lycopersicum* (8), *Capsicum annuum* (6), *Arabidopsis thaliana* (6), *Brassica rapa* (2), and *Brassica oleracea* (1), but no homologous genes were found both sweetpotato and rice. 39 (72.2%) and 40 (74.1%) *IbDEAH* genes were also homologous to the genes in *Ipomoea trioba* and *Ipomoea trifida*, respectively, accompanied by *Solanum lycopersicum* (8), *Capsicum annuum* (3) and *Arabidopsis thaliana* (3), nevertheless, no similar homologous genes were noted between sweetpotato and *Brassica rapa*, cabbage and rice. Similarly, 89 (46.1%) and 87 (45.1%) *IbDExDH* genes were homologous to the genes in *Ipomoea trioba* and *Ipomoea trifida*, followed by tomato (24), pepper (10), *Arabidopsis* (7), *Brassica rapa* (3), and cabbage (2), however, no homologous genes were found with rice (Figs. 11, 12 and 13). It should be mentioned that the collinearity of sweetpotato RNA helicase genes between the *Ipomoea trioba* and *Ipomoea trifida* genes more than the extra six varieties, which can be associated to the wild diploid relationship of sweetpotato.

Moreover, the mass of the RNA helicase genes in our sweetpotato are one-to-one gene pairs with sweetpotato diploids, and a few are one sweetpotato gene corresponding to two or three diploid gene pairs. For example, the sweetpotato genes *IbDEAD* (10), *IbDEAH* (2) and *IbDExDH* (19) have a one-to-many relationship in *Ipomoea trioba*, and *IbDEAD* (10), *IbDEAH* (2) and *IbDExDH* (17) sweetpotato genes have a one-to-many relationship in *Ipomoea trifida* (Supplementary Table 3). Interestingly, we found that *IbDEAH* did not find some collinear gene pairs between sweetpotato and *Oryza sativa* / *Brassica rapa* / *Brassica oleracea*, and there was no collinear gene pair between sweetpotato RNA helicase and rice.

### Tissue expression patterns of cold-response genes of the RNA helicase family of different sweetpotato cultivars

**Fig. 14:**
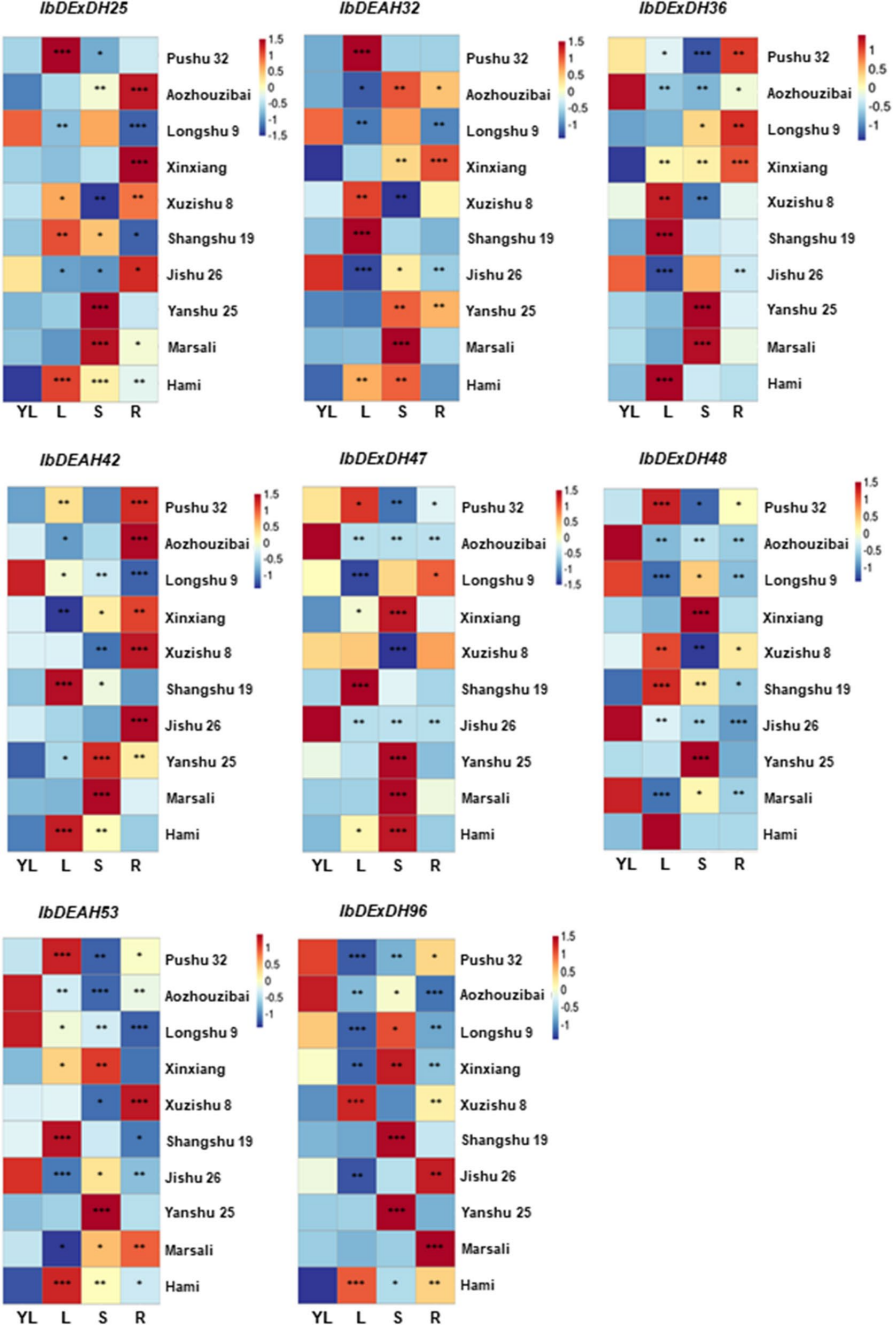
Heat map representation for the organ-specific RNA helicase gene expression profiles in different sweetpotato varieties. YL, Young leaves, L, leaves, S, stems and R, roots. The expression levels of genes are presented using fold-change values transformed to Log^2^ format. The Log^2^ (fold-change values) and the color scale are shown at the bottom of heat map. For all the columns labeled with “*,” “**,” and “***” indicate significant differences at *P* < 0.05, *P* < 0.01, and *P* < 0.001, respectively, compared with the YL

Objective to preliminarily understand the role of the RNA helicase gene in the development stage of sweetpotato, the expression contours of the RNA helicase genes in diverse tissues of 10 sweetpotato varieties were analyzed by qRT-PCR, such as young leaves, leaves, stems and roots. The expression contours of eight RNA helicase genes were grouped together on their respective heat maps (Fig. 14). The expression of the RNA helicase in various tissues is different, and the expression in different varieties of sweetpotato is also quite different. This also shows that the RNA helicase is related to growth and development [44, 45]. Some the RNA helicase genes were highly communicated in stems and roots of sweetpotato, such as *IbDExDH25*, *IbDEAH42*, *IbDExDH47*, and *IbDExDH96*. Similarly, some RNA helicase genes were also highly communicated in sweetpotato leaves, including *IbDExDH36*, *IbDExDH48*, and *IbDEAH53*. This shows that the expression of the RNA helicase in unequal growth stages of sweetpotato is diverse and have an influence in the development stage of plants. This is steady with the results of other plants in RNA helicase. HS3 is located in the DEAD-box RNA helicase 22 in *Arabidopsis* plastids, which is necessary for proper accumulation of plastid gene mRNA throughout seed germination and plant growth [46, 47]. The RNA helicase can also concern the development of plants under chilling stress. AtRH7, one of the RNA helicases in *Arabidopsis*, is an RNA chaperone involved in cold adaptation [48]. The mutation of rh7 affects the abnormal development of flowers in *Arabidopsis* thaliana, and makes the leaves of *Arabidopsis thaliana* smaller in chilling stress [49].

### Analysis of cold stress related genes in *IbDExDH96* overexpressing sweetpotato

**Fig. 15:**
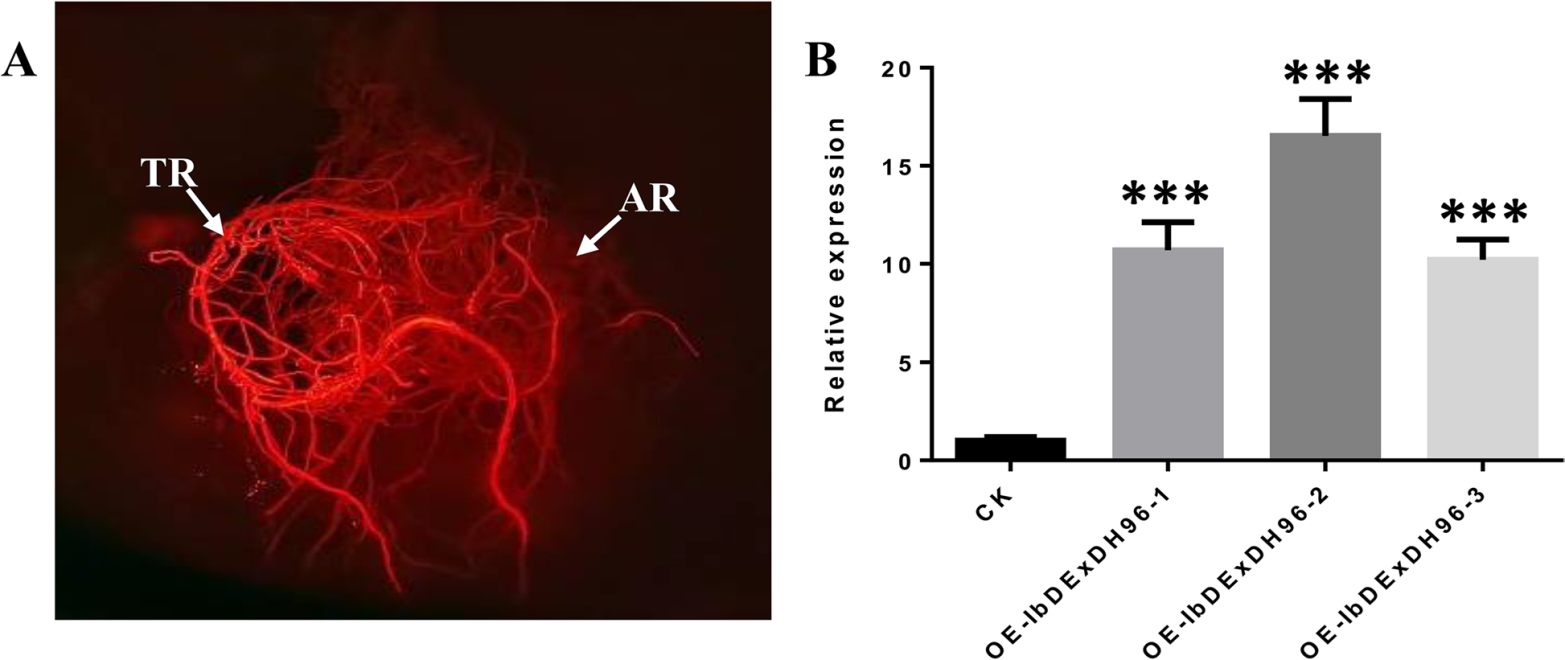
*A. rhizogenes*-mediated transgenic sweetpotato. **A** *pUBI.U4::IbDExDH96-CaMV35S::DsRed* expression diagram, TRs: transgenic roots; ARs: adventitious roots. **B** Identification of OE-IbDExDH96 transgenic lines, the columns labeled with “***” indicate significant differences at *P* < 0.001

**Fig. 16:**
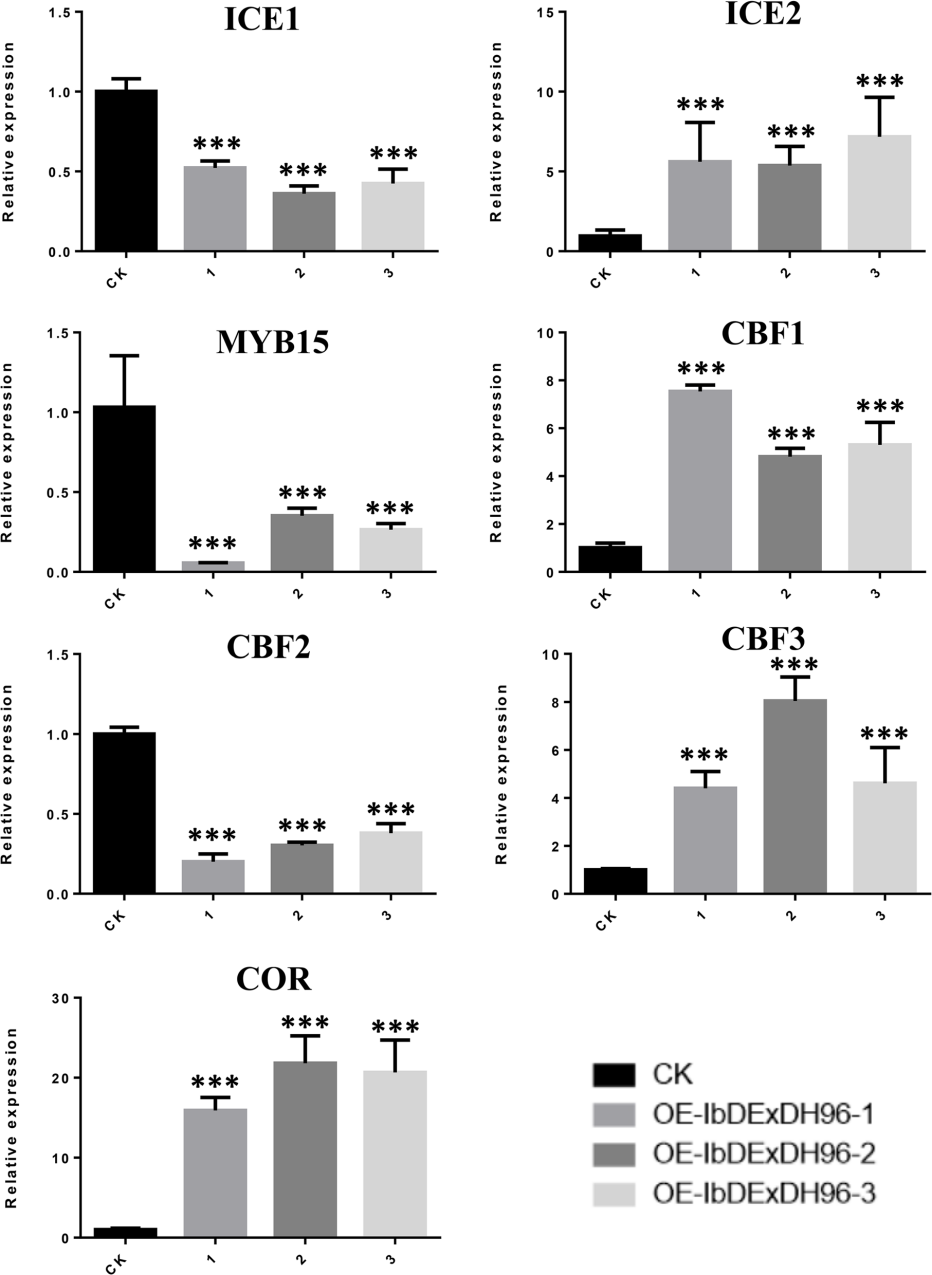
Expression of cold stress-related genes in OE-IbDExDH96 transgenic lines, the columns labeled with “***” indicate significant differences at *P* < 0.001

From the transcriptome of sweetpotato subjected to cold stress, we screened eight RNA helicase genes. Among them, four genes, namely *IbDEAH32*, *IbDExDH47*, *IbDExDH48*, and *IbDExDH96*, exhibited responsive behavior to cold stress and were selected for further amplification. Successfully constructing the *IbDExDH96* overexpression vector, we mediated its integration into sweetpotato hair roots using rhizobium. Through qRT-PCR analysis, it was observed that the overexpression of OE-IbDExDH96 reached a peak level approximately 15.5 times higher than CK (Fig. 15). Upon analyzing cold-related genes in the transgenic sweetpotato hair roots lines, significant down-regulation was observed in *MYB15*, *ICE1*, and *CBF2* compared to the CK, while *ICE2*, *CBF1*, *CBF3*, and *COR* genes showed significant up-regulation in the transgenic sweetpotato hair roots lines (Fig. 16 and Supplementary Table 5). These results indicate that the overexpression of *IbDExDH96* in sweetpotato significantly enhances the expression of key genes under cold stress conditions, providing a solid foundation for further exploration of cold stress tolerance in transgenic sweetpotato.

## Discussion

The RNA helicases are ubiquitous in miscellaneous organisms [50]. It is involved in almost all processes of RNA metabolism, such as transcription, mRNA splicing and output, mRNA translation, etc. It involves almost all aspects of organisms [4, 51, 52]. In the past, the study of the RNA helicase family in plants mainly focuses on dicotyledonous model plant *Arabidopsis thaliana* and monocotyledonous rice [53–55], these play an essential role in plant growth and stress response [56, 57]. Sweetpotato is a significant food crop, which is broadly used in food, feed and industrial raw materials. It is a hexaploid plant, the seventh largest food crop in the world, with the strong ability to adapt to the environment, varieties of high yield, strong stress resistance [58, 59]. However, only 17 RNA helicase genes have been nominated in *Ipomoea trifida* [20]. There is no comprehensive study on RNA helicase in sweetpotato. This study systematically identified RNA helicase genes in sweetpotato, which laid a solid basis for further study on the engagement of the RNA helicase in plant abiotic stress and development.

In the present study, we conducted a comprehensive reasoning of the sweetpotato RNA helicase gene family, containing phylogenetic tree, expression profiles of different sweetpotato varieties under common growth circumstances, and expression profiles under diverse abiotic stresses and hormone stresses. 300 RNA helicase genes were nominated in sweetpotato genome, which is a very large gene family. A larger family of the RNA helicase gene exists in sweetpotato, suggesting that RNA helicase plays a leading role in regulating environmental responses. According to the family classification of *Arabidopsis* and rice RNA helicases [21], they were divided into three subfamilies, including IbDEAD (53), IbDEAH (54) and IbDExDH (193) (Supplementary File 1). Studies have indicated that the DExDH subfamily in *Arabidopsis*, rice, and maize is larger than the DEAD and DEAH subfamilies. Additionally, being a hexaploid plant, sweetpotato possesses a greater number of genes in each subfamily compared to diploid and tetraploid plants.

The sweetpotato RNA helicase gene contains different numbers of exons and different lengths of introns (Figs. 2, 3 and 4). Intriguingly, five genes in the IbDExDH subfamily contain only one exon, while the IbDEAD and IbDEAH subfamilies do not contain this case and contain multiple exons. In fact, the length of RNA helicase family proteins varies greatly. IbDEAD is 323 to 1301 amino acids, IbDEAH is 206 to 2904 amino acids, and IbDExDH is 128 to 2801 amino acids. The highly different amino acid sequences of these sweetpotato RNA helicases induce diverse protein structures and functions in dissimilar developmental and stress-resistant environments [60].

At the same time, the sweetpotato RNA helicase motif was analyzed, the most conserved motif in different species is Asp-Glu-Ala-Asp, which is divided into three subfamilies according to its difference [16, 61, 62]. According to the structural characteristics and phylogenetic analysis of the Motif V region, the determined helicases can be moreover divided into these subfamilies, comprising IbDEAD, IbDEAH, and IbDExDH. Phylogenetic analysis showed that IbDEAD, IbDEAH and IbDExDH RNA helicase proteins can be moreover divided into nine, or thirteen great subgroups (Fig. 1). The gene structure results showed that the main RNA helicase genes in *Arabidopsis* were uniform to the AtRH family genes [24], but the position and length of introns were not fixed. However, this is the first genome-wide examination of the RNA helicase genes family in sweetpotato. The distinct subfamilies and gene structures of sweetpotato RNA helicase genes indirectly indicate the different functions in RNA metabolism, stress resistance and growth and development [49, 63].

In fact, we establish that most RNA helicase gene promoters contain some cis-regulatory elements, such as plant development [64], abiotic stress [65], plant hormones, and light response elements [50, 53, 66]. It is worth noting that IbDEAD and IbDEAH have more cis-regulatory elements associated to low temperature and light response, and IbDExDH has more cis-acting elements associated with abscisic acid and plant hormones (Figs. 6, 7 and 8). According to the study that temperature, abscisic acid and jasmonic acid are involved in abiotic stress processes in plants [67–69]. Under hormone and abiotic stress treatments, qRT-PCR data showed that RNA helicase mainly responded to abiotic stress (Figs. 9 and 10).

It was observed that under drought stress, the DEAD-box RNA helicase *CmRH56* inhibited rhizome growth by disrupting gibberellic acid (GA) biosynthesis [70]. By regulating salt-tolerant genes, the DEAD-box RNA helicase BnRH6 plays a negative role in promoting the adaptation of Brassica plants to salt stress [71]. By modulating the extent of membrane lipid peroxidation, *Triticum aestivum* DEAD-box RNA helicase *TaDEAD-box57* has the potential to enhance the tolerance of transgenic plants to drought, salt, and low-temperature stresses [72]. In *Arabidopsis*, DEAD-box LOS4 is able to participate in the process of cryogenic stress processes [73, 74]. Among them, in the cold stress response pathway, LOS RNA helicase plays a key role in target gene output, maturation and reaction to temperature stress. The transcription of STRS1 and STRS2 was inhibited under salt stress. The salt tolerance of mutants strs1 and strs2 was enhanced, and the expression of RD29A, DREB1A and DREB2A was enhanced [75]. The development of the germ and leaf of the *Arabidopsis* rh7 mutant was seriously delayed under low temperature stress [49]. The growth of rh3 mutants was severely inhibited under salt or cold stress [76]. Our previous findings showed that the tolerance of tomato SlDEAD31 was enhanced in salt and drought stress [41]. The rice SUV3 protein has DNA and RNA helicase and ATPase activities, and *SUV3* expression can be induced by salt stress [77, 78]. Low temperature and high salt stress can induce the expression of GmRH in soybeans, and GmRH plays a significant in RNA processing [79]. Tobacco P68 can enhance tolerance to salt stress [80]. AvDH1 increased salt tolerance [81]. The *Arabidopsis* RCF1 gene plays an integral role in maintaining normal splicing of mRNA precursors, and some cold stress-induced genes were error spliced in the rcf-1 mutant [82]. Maize DRH1 can interact with the nucleoprotein fiber MA16, which is involved in ribosomal RNA metabolism [83]. DEVH-box RNA helicase AtHELPS play a key role in K^+^ deprivation in *Arabidopsis thaliana* [84]. The presence of numerous cis-regulatory elements in the promoter regions of sweetpotato RNA helicase genes highlights their crucial role in conferring stress resistance to the plant. This discovery has led us to identify novel candidate genes involved in regulating sweetpotato ability to withstand abiotic stress through RNA metabolism. Given that sweetpotato ranks as the seventh largest food crop globally, its economic significance and environmental adaptability pose significant challenges. The unveiling of stress-related genes has laid a strong groundwork for advancing our understanding of the molecular mechanisms underlying sweetpotato resistance and expediting the development of resilient sweetpotato varieties through breeding efforts.

RNA helicases play a crucial role in regulating plant development and responding to environmental cues. To gain insights into their functionality, we conducted an analysis of the expression profiles of RNA helicase genes in various tissues of different sweetpotato varieties. Additionally, their response patterns were examined under four abiotic stresses and four hormone stresses (Fig. 14). Our findings revealed significant variations in the expression of sweetpotato RNA helicase genes across different tissues, indicating their involvement in plant development. Furthermore, these genes exhibited prominent responsiveness to abiotic stresses, such as cold, heat, and salt, underscoring their significance in enhancing plant stress resistance.This is consistent with the functional studies of other plant RNA helicases, for instance *Arabidopsis* [48], rice [85], tomato [86], rapeseed [71], chrysanthemum [70], Zea mays [87], barley [72] and soybean [88]. Furthermore, we proceeded to develop sweetpotato lines through *A.rhizogenes*-mediated overexpression of *IbDExDH96*, an RNA helicase gene. This allowed us to conduct initial validation of its involvement in cold stress response in sweetpotato. Based on our findings, we propose that RNA helicase proteins not only serve as functional genes influencing the growth and development of sweetpotato but also act as regulatory factors under diverse environmental conditions. Hence, our study presents novel experimental insights and provides valuable leads to support the aforementioned speculation.

In summary, delving into the reception of the RNA helicase gene in sweetpotato holds the potential to enhance transgenic research aimed at improving the crop's yield and stress resistance. The exploration of the identification, classification, and phylogenetic tree construction of sweetpotato RNA helicase genes through bioinformatics has yielded valuable insights for a comprehensive investigation into the biological functions of these genes. Furthermore, these studies significantly contribute to our understanding of the molecular basis underlying several crucial agricultural traits in sweetpotato cultivation. However, the precise regulatory mechanism governing the development and stress response of sweetpotato RNA helicase genes remains elusive and warrants further investigation.

## Conclusion

In this research, we conducted an exhaustive genome-wide reasoning of the sweetpotato RNA helicase family, containing chromosome distribution, promoter elements, and protein motif analysis. All of 300 RNA helicase genes were detected, containing IbDEAD, IbDEAH and IbDExDH subfamilies. The expression patterns of eight RNA helicase genes in different sweetpotato varieties and their responses to abiotic stress and hormonal stress were analyzed by qRT-PCR. The expression of the RNA helicase genes was significantly distinct in individual tissues of 10 sweetpotato varieties and notably raised under divergent abiotic stresses. The overexpression of the RNA helicase gene *IbDExDH96* in transgenic sweetpotato hair roots, mediated by rhizobium, led to alterations in the expression levels of cold-related genes. This enabled us to investigate the role of sweetpotato RNA helicase in alleviating cold stress. We obtained preliminary evidence supporting its involvement in endogenous cold stress tolerance. However, further exploration is still required to elucidate the specific mechanism of the sweetpotato RNA helicase gene. The results showed that RNA helicase was complicated in the direction of extension and the resistance to stress of sweetpotato. This study supplies new inspirations into the development and exploration of the RNA helicase gene families.

### Supplementary Information

**Supplementary Material 1.**

## Data Availability

The RNA-seq data in this study was acquired from NCBI with the accession number GSE255226 (https://www.ncbi.nlm.nih.gov/geo/query/acc.cgi?acc=GSE255226). The datasets supporting the conclusions of this article are included in the article and its supplementary material.
