# Supplementary material for: Genome-wide systematic survey and analysis of the RNA helicase gene family and their response to abiotic stress in sweetpotato
Source: BMC Plant Biol. 2024 Mar 16;24:193. doi: 10.1186/s12870-024-04824-z (PMC10944623; doi:10.1186/s12870-024-04824-z)
Supplement: Supplementary file 1 — Supplementary Material 1. [file 12870_2024_4824_MOESM1_ESM.zip › Supplementary data/Supplementary Figure.2.docx]

**C**


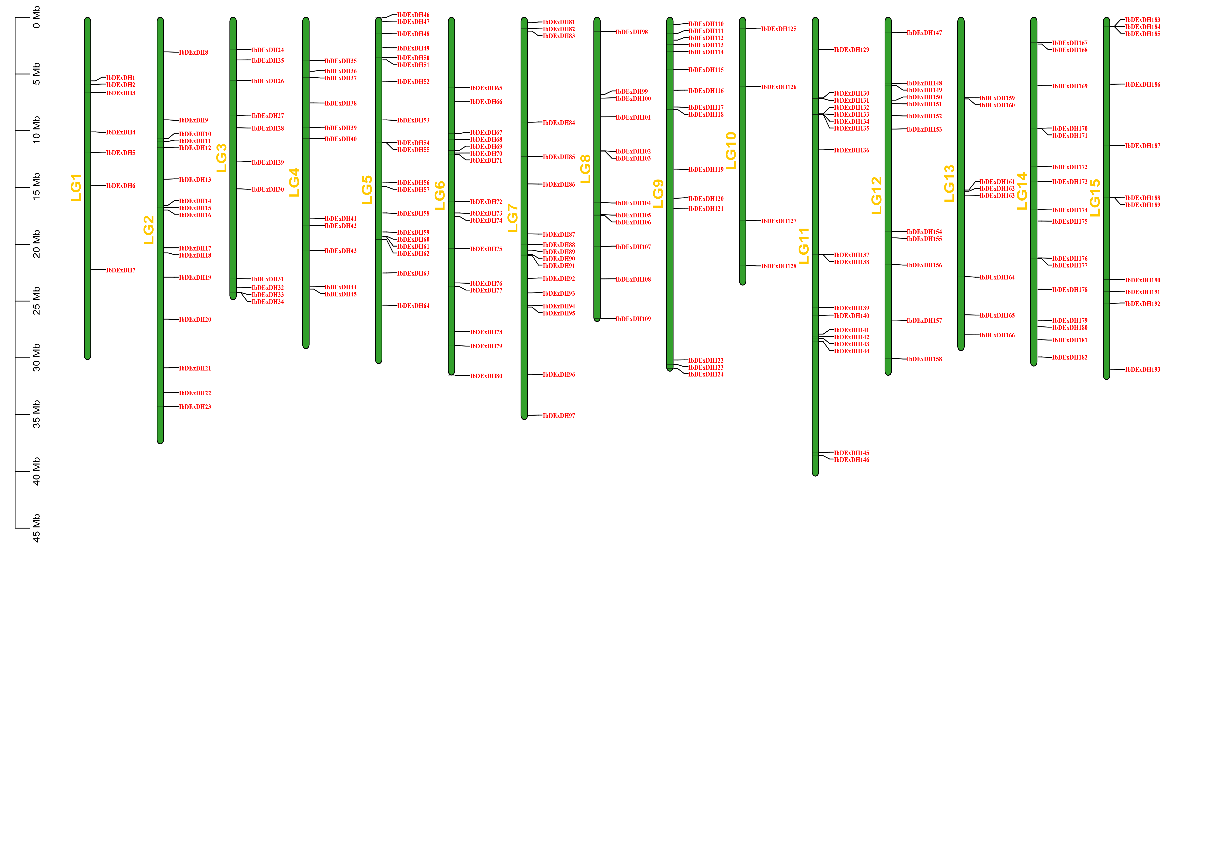


**B**


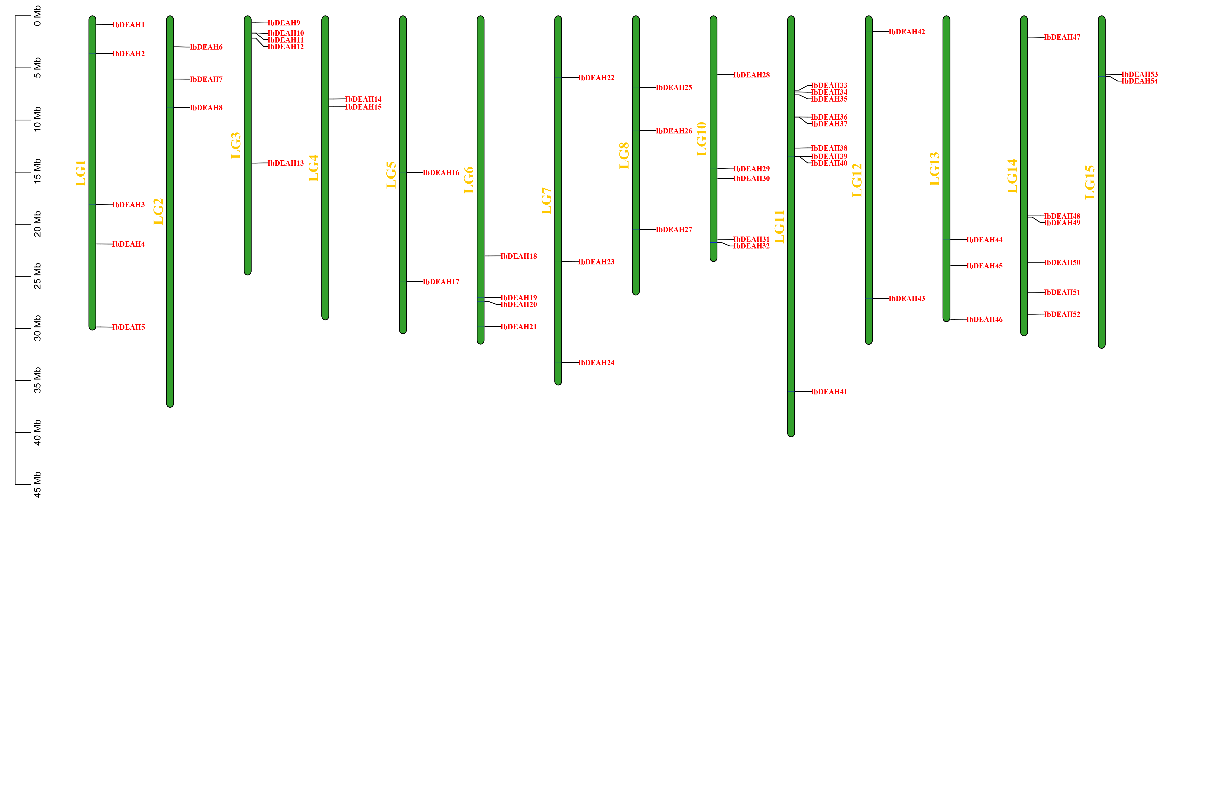

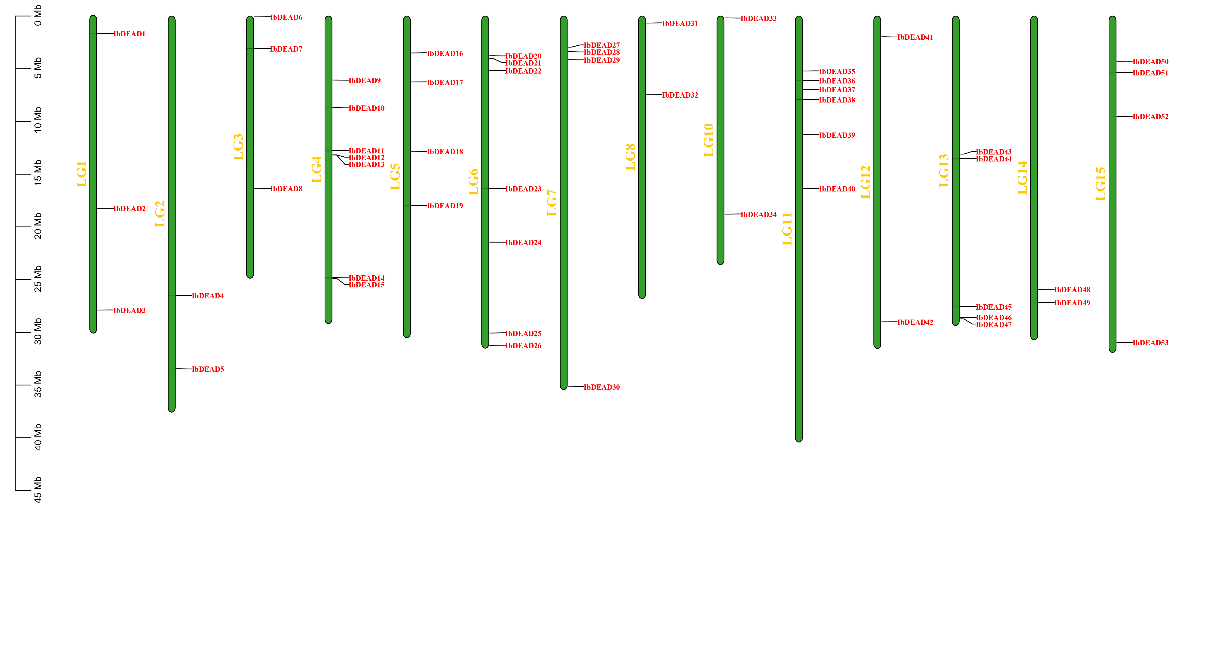


**A**

**Supplementary Figure.2** Schematic representations of the chromosomal distribution of the RNA helicase genes on 15 sweetpotato chromosomes. (A) Chromosome locations of 53 *IbDEAD* genes. (B) Chromosome locations of 53 *IbDEAH* genes. (C) Chromosome locations of 53 *IbDExDH* genes. The chromosome numbers 1-15 are indicated to the left of each chromosome as LG1-LG15. The scales were indicated the genome size of sweetpotato genome (Mb).
