# Supplementary material for: Genome-wide systematic survey and analysis of the RNA helicase gene family and their response to abiotic stress in sweetpotato
Source: BMC Plant Biol. 2024 Mar 16;24:193. doi: 10.1186/s12870-024-04824-z (PMC10944623; doi:10.1186/s12870-024-04824-z)
Supplement: Supplementary file 1 — Supplementary Material 1. [file 12870_2024_4824_MOESM1_ESM.zip › Supplementary data/Supplementary Figure.3.docx]

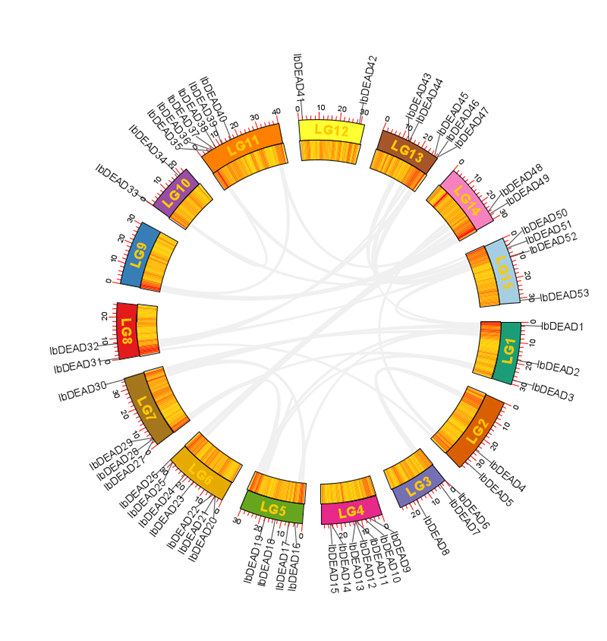


**Supplementary Figure.3** The chromosome relationship of *IbDEAD* genes in sweetpotato chromosome. Circular visualizations of RNA helicase genes mapped to the LG1- LG15 chromosomes are indicated by colored rectangles.
