# Supplementary material for: Genome-wide systematic survey and analysis of the RNA helicase gene family and their response to abiotic stress in sweetpotato
Source: BMC Plant Biol. 2024 Mar 16;24:193. doi: 10.1186/s12870-024-04824-z (PMC10944623; doi:10.1186/s12870-024-04824-z)
Supplement: Supplementary file 1 — Supplementary Material 1. [file 12870_2024_4824_MOESM1_ESM.zip › Supplementary data/Supplementary File.1.docx]

## **Supplementary File.1 The amino acid sequences of 300 RNA helicase identified in sweetpotato genomes.**

53 IbDEAD

>IbDEAD1

MAAIASADPRYAPEDPSLPKPWKGLVDGKTGYLYYWNPETNITQYERPGGSAPIHKSSAGFVSSSVQKSSHGQHRDNDDVDRYGRGHRGSSKPSGGEDYQSERNNYDHSRGVSNMPLVGNGASAYKNSSSTVESDLSPDAYRRRHEISVTGDNVPPPLTSFNSTGFPSEILREVRYLLLGGVKCRPSGSSMFVAGTAVGDDSICHVVRMEEEESSCSSLQQYALAAYVHQAGFSAPTPIQAQSWPVALQGRDIVAIAKTGSGKTLGYLIPGFIHLKQRRNNSRLGPTVLVLSPTRELATQIQDEAVKFSKSYQISCTCLYGGAAKGPQLRDLDRGVDIVVATPGRLNDILEMRRVSLNQVSYLVLDEADRMLDMGFEPQIRKIVKEVPTRRQTLMYTATWPKEVRKIAADLLINPVQVNIGNVDELVANKSITQYVEVLPQTDKRRRLEQILRSQEQGSKVIIFCSTKKMCNLLAGNLTRQFGAAAIHGDKSQGERDFVLNQFRTGRSPVLVATDVAARGLDIKDIRVVINYDFPTGIEDYVHRIGRTGRAVVEWEGQDVHGVLAQLGAMEVVGRYDSGNGVRDGARGSWGMSSSSDRGGGRGYDRDSRDRHGQGSRDADSFGSYNSRNFNDSVAHTSDRHGRSRSRSPNRASGWSGDNKSQGRSRSRSVDRFKPSNPSREGPTGRSFQEAVIPRAGTSSLALKQRSPPYDSENLKRSYTNGQKDWERSPPLKGQSNREYANGSHQTCRRRKRKA

>IbDEAD3

MAATNKALTATRFSDLKPPLSQPVLEALTNGGFDFCTPVQAATIPLLCSHKDVAVDAATGSGKTLAFVVPLVEIIRRSSPPKPHQVMGIVISPTRELSSQIFHVAQPFISTLPNIKPVLLLGGAEVKTDMKKIEEEGANLLIGTPGRLFDIMERMDMLDFRNFEGVVTLFSNDSKKDEADRLLDMGFQRQINSIISRLPKLRRTGLFSATQTEAVEELSKAGLRNPVRVEVRAEAKSLNDSTSTGQLASSRTPAGLQDEYLVCETDKKSSQLADLLIRNKSNKVIIYFMTCACVDYWGLVLPRISSLKKLSLIPLHGKMKQAAREKALSTFTSLSSGILLCTDVAARGLDIPGVDCIIQYDPPQDPDVFVHRVGRTARLGRQGHAIIFLSPKIRLAAKKDRDVMEKGIRAFVSYIRAYKEHNCSYIFRWKELEIGKLGMGYGLLQLPSVPEVKHHSLSTESFIPVKDINLEEIKYKDKSREKQRKKNLEAKKAVKQQEVRKPKRDSSLSKAEMKKQTAKKRRATQTAEDDDEMAKEYRLLKKLKKGAIDETEFAKLTGTEDLL

>IbDEAD4

MATAEAATSSLGPRYAPDDPTLPQPWKGLIDGNTGLLYYWNPETNVTQYEKPSALPPPLPPGPPPASTTPKLAPIPGASAVPSSDVQGQQGLHMGGQNLQQGQHMAPLLQQHPQVTQQGPSQVRLDSNRDHLATDFSTNNAAHSTATGPASAAIPGSADRKTPRFSIYSAPPDFTWILSGSASGQTPTLGANPSLGPQYSGSSVNMQQPTSQVQWQHSGTDSVHHQHSSRFQSQMGPGSSHGQQLNVPPLGSKTSYEENSHGRVGNKYFDNTSKDAHAMPPQASHPTLAAIPHARSQHEMRMGDITVQNPAHGFPGGYSNAGGPPLHNTYGQATGGPQFPNHAQMRPPAAVMGPSDAEDYRQKHEVSTMGDNVPDPFITFEATGFPPEILRDTCIKLNSSRLSQVRYLLRRRSTCHSFCWISISYTNSSTNMAHCTTKSDIVAIAKTGSGKTLGYLIPAFIQLSRRRNNPQNGPTCLYGGAPKGPQLKELDRGADIVVATPGRLNDILDMKRIDFRQVSLLVLDEADRMLDMGFEPQIRKIVNVIPSNRQTLMYTATWPKEVRMIASDLLRNPVQVNIGSVNELEANKSITQYVELVPQMEKQRLEQILRSQERGSKVIIFCSTKDCVTSLQSIGRSFGAAAIHGDKSQVERDWALNQFRSGRSPILVATDVAARGLDIRDVRVVINYDFPTGIEDYLTLLKFWRVPTSKCLNKSEIWLGNGPNFGKDRAEMNRSDSGMDNGVRTRWDSGGRGGMRDGGFGGRGMRDGGFGGRGAGRDGGFGGLGGRDGGFMGRGGRDGNFGGRGGMRDGHFGGRGGRVAPGGHTGWDRNDRGPRDRFNNLDSRGRGRGRGAGRFDNRRDTSNMSRGRGHSSSPERVRTWGRGSRSTSRSLEAGVTAGVVVEAGAGAAVIAQDGAGVAAAVSAGAGVAAAMIDTVGGLSVSKFDQMEVPGVEVAPELARQPVALHPQGSGFAGADTAEQKSGVEAAPESAMSPMSPGTQGFSGANPPSH

>IbDEAD5

MDRDASFLFAGTNFNPKKFTDVFDRFKGKKESDEAEEKLNLIDNGSTQKEEHTSLRVSKKRKRKGAVSDPVEGFSVFTSSKSKTEADGEGKEHIGSEILEGKKEYYRQLERDAIFRKKHNIHVSGSNVPSCLQSFTELKSRFGCKSYLLRNLAKLGFKEPTPIQMQAIPVILSGRECFACAQTGSGKTFAFVCPILMKLKQTSKDGVRAVILCPTRELAAQTARECKKLTKRKEFHIKLMTKQLAKSADFSVLHCDILISTPFRLQYAINKRKLDLSRVEFLVLDEADKLFELGLMEQVDSVFKACSNPSILHSLFSATLPDEVEKIARTIMHDAVRVIIGRKNSASETIKQKLVFVGSEEGKFLALRQTFSESLNPPVLIFVQNKERAKELYNEVKFDDIRADVIHSDLSQKQASLQPIPCVIPIRENAVDNFRAGKTWVLIATDVIARGMDFKGVNYVINYDFPDSSSAYIHRIGRSGRAGRSGEAITFYTEADVQFLRNIANVITASGGEPAISPTDSQILQRIRLCIMGSMERSKKKVQLWKKAIVHFLLCFVMGFFTGFAPAGKASIFSGRLSVSKAQANNTGKPVEIPHGTRTQGENLNGTLLGRNFSAEPARSNNASKPALSKKLQAKGVAEDLDPRRLIIIVTPTSGKNELRGVLIRRLASTLRLVPQPLVWVVVEQQSEDSEVSEILRKTGIMYRHLVSKENFTDIQEELDHQRNVALNHIEHHRLSGIVHFAGLFNVYDLSFFHQLRSIEVFGTWPVALLSANKNEVVIEGPVCDSSEVIGWHLKRANINNNNQTDDDQKPAIRVSNFAFNSSILWDPERWGRTSSLQDTSQDSLKFVRKEVIEDEAKLMGIPQEDDCSKVLLWNLQFSTL

>IbDEAD8

MCLRILEIEDFLHRPFYPTPAASYAGAIASGNDRVGYGGPPAGGSRWNGPKNEFRSSYGGSTSSSSWNSRTGGWDRAGEREVNPFGDEGEKPFDEHENSGINFDAYEDIPVETSGENVPPPVNTFAEIDLGEALNNNIRRCKYVKPTPVQRYAIPISLTGRDLMACAQTGSGKTAAFCFPIISGIMRGQFPRPPHPRMAFPLALILSPTRELSCQIHDEARKFAYQTGVKVVVAYGGAPINQQLRELERGVHILVATPGRLVDLLERARVSLEMIRYLALDEADRMLDMGFEPQIRKIVQQTDMPPPGMRQTMLFSATFPKEIQRLAADFLSNYIFLAVGRVGSSTDLIVQRVEYVQETDKRSHLMDLLHAQRANNVNGKQALTLIFVETKKGADSLEHWLYLNGFPATSIHGDRTQQEREYALRSFKSGKTPILVATDVAARGLDIPHVAHVINFDLPNDIDDYVHRDRKNWTSRKTGLATAFFNENNSSIAKPLADLMQEANQEVPAWLSRFAALSFSYGGKNRRGGNRFGGRDFRKEPAYNKGSADYYSTSNSNTGYGGSSGYNAASSAGVASAWD

>IbDEAD7

MSYVPPHLRNSTAATTVVSNGGDHLHSKLNSRQLPSFTQSKNGFTSNGEYCSNDSGRSSCETVNYLSPRGLVAPDPVFPQWKPSERVLLLKLEQIEEIRLRLNVDVAVALDSHPAPAPVESFTDMCLHPSIMKDIALHNYTIPTSVQAQAMPVALSGRDLLGCAETGSGKTAAFTIPMIQHCLAQPPLRRGDGPLALVLAPTRELAQQIETEVKAFSRSLDSFKTAIVVGGTNIGEQRSELRAGVNIVVATPGRLIDHLQQGNTSLSRISFVVLDEADRMLDMGFEPQIREVMHNLPKKHQTLLFSATMPAEIEALAQNIGSESPCLGPIGLSGGITPSEEYLTDPVGKGWKCEQSNSKCSSDFGENLLVEESAQAEKSGHPFSLTIVFVERKVKVKTTVKRSCSSVATNSVFLLNPVAYWQTKCEEVAEALMKQGLLATALHGGRSQNEREAALRDFRRGPTSILVATDVASRGLDVNGVAHVINLDLPKTMEDYVHRIGRTGRAGSTGQATSFYTDRDMYLVAQIRKAIADVGSGNTVTFATGKTARRKEREAAAAHKEANTALSKLSLMGSTPINVEDKYRHMFAPAMARKEGAADGAWDD

>IbDEAD13

MVQKHLACMPLPEEIFVVAGPEKVRRRRPIPSIPFIVVRDRDPHFWNVLQFHEPTPIPKPSTIGKSSSGALSLRQIVRGFHASRPLAAGYAVADLPDDDEGLEIAKLGISQEIVSALAQKGITKLFPIQKAVLEPAMKGSDMIGRARTGTGKTLAFGIPIMDKIIRYNEKHGRGRNPLALILAPTRELAKQVDKEFYESAPNLDTLCVYGGVPIGRQMGQLERGVDVVVGTPGRIIDLIRRGALNLAEIQFVVLDEADQMLNVGFAEDVETILENVPRKRQTMMFSATMPNWIMKLTQKFLRTPVHIDLVGDSDQKLADGISLYSIACDMRSRPVILGPLITEHAKGGKCIVFTQTKRDADRLAYAMQRSHRCEALHGDISQNQRERTLSGFRQGHFNILVATDVAARGLDVPNVDLSTISYGMQVMTGKKGSAVLIYSAQQLRDVRGIEREVGCRFVEV

>IbDEAD15

MSQCVHCYAFLIIINRPPRRSRRHPGVRLTLSAFYNIDQDVTATKGNEFEDYFLKRELLMGIYEKGFERPSPIQEESIPIALTGSDILARAKNGTGKTAAFCVPALEKIDTETNVIQVVILVPTRELALQTSQVCKELGKHLKIQVMVSTGGTNLKDDIMRLYQPVHLLVGTPGRILDLAKKGICILKDCAMLVMDEADKLLSPEFQPSVEHLITFLPEYRQILMFSATFPVTVKDFKDRYLRKPYVINLMDELTLKGITQYYAFVEERQKVHCLNTLFSKLQINQSIIFCNSVNRVELLAKKITELGYSCFYIHAKMLQDHRNKVFHDFRNGACRNLVCTDLFTRGIDIQAVNVVINFDFPKNSETYLHRVGRSGRFGHLGLAVNLITYEDRFNLYKIEQELGTEIKPIPPHIDQAIYCQIFSLWFWGGVMCCLTVPRDMTGEGKLDFPKDTTVVTPEAVATGC

>IbDEAD11

MGSLFLRKSSSSPAPKRFAVAALSHLFHSSSSATEILISGNVLQFHEPTPIPKPSTIGKSSSGALSLRQIVRGFHASRPLAAGYAVADLPDDDEGLEIAKLGISQEIVSALAQKGITKLFPIQKAVLEPAMKGSDLIGRARTGTGKTLAFGIPIMDKIIRYNEKHGRGRNPLALILAPTRELAKQVDKEFYESAPNLDTLCVYGGVPIGRQMGQLERGVDVVVGTPGRIIDLIKRGALNLAEIQFVVLDEADQMLNVGFADDVETILENVPRKRQTMMFSATMPNWIMKLTQKFLRTPLHIDLEHAKGGKCIVFTQTKRDADRLAYAMQRSQRCEALHGDISQNQRERTLSGFRQGHFNILVATDVAARGLDVPNVDLVVHYELPNSSEIFVHRSGRTGRAGKKGSAILIYSAQQLRDVRGIEREVGCRFVELPRIEVDAGTTDMIGDMGVDGSHFGSNRGMGGGRFGGGYGGSGSGRSSGGRSGGGFGGGRSSGGFGGGRSSGGFGGGRSGRFDDFGSSLSGGFGGQSSSRRSGGFGNYDGSDRGGSFGRTQSSNRSGGFGEFGSGRPSGFGFFGDDNKSRNS

>IbDEAD12

MGSLFLRKSSSSPAPKRFALAALSHLFHSSSSATEILISGNVLQFHEPTPIPKPSTIGKSSSGALSLRQIVRGFHASRPLAAGYAVADLPDDDEGLEIAKLGISQEIVSALAQKGITKLFPIQEGKEPLALILAPTRELAKQVDKEFYESAPNLDTLCVYGGVPIGRQMGQLERGVDVVVGTPGRIIDLIRRGALNLAEIQFVVLDEADQMLNVGFAEDVETILENVPRKRQTMMFSATMPNWIMKLTQKFLRTPVHIDLVGDSDQKLADGISLYSIACDMRSRPAILGPLITEHAKGGKCIVFTQTKRDADRLAYAMQRSHRCEALHGDISQNQRERTLSGFRQGHFNILVATDVAARGLDVPNVDLVVHYELPNSSEIFVHRSGRTGRAGKKGSAVLIYSAQQLRDVRGIEREVGCRFVEYRFVCILLIDFGGVSNVKPVKQGAWEVGVLVVDLVQADQVVVLVGARSGGGFGGGRSSGGFGGGRSSGGFGGGRSGRFDDFGSSLSGGFGGQSSSRRSGGFGNYDGSDRGGSFGRTQSSHRSGGFGEFGSGRPSGFGFFGDDNKSRNS

>IbDEAD9

MSRPLDEQGSKVDGAKKPVFLTKAQREQLALQRRQEEIAEQKRRAELILQQARLPSIDAAASSAKDNNKPSSDHDRDRDRDRDRDHHRSSRDKDRERERERDRERDRERERDRERDRDRESERRKREREREDEAKERERARVEKLAEREREKELDSIKEQYLGSKKPKKRVIKPSEKFRFSFDWENTEDTSRDMNSLYQNPHEARLLFGRGFRAGMDRREQKKLAAKNERELREEIARRMVLRRLQKKLEEMTERDWRIFREDFNISYKGSRIPRPMRSWTESKLTPELLKAVERAGYKTPSPIQMAAIPLGLQQRDVIGVAETGSGKTAAFVLPMLTYISRLPPMSEENEAEGPYAVVMAPTRELAQQIEDETVKFAHYLGIKVVSIVGGQSIEEQGFRIRQGCEVVIATPGRLLDCLERRYCVLNQCNYVVLDEADRMIDMGFEPQVVGVLEAMPSSNLKPENEDEELDEKKIYRTTYMFSATMPPAVERLARKYLRNPVVVTIGTAGKTTDLITQHVMMVKESEKTFRLQKLLDELGDKTAIVFINTKKQADTVSKNLDKAGYRVTTLHGGKSQEQREISLEGFRTKRYNVLVATDVAGTWIDIPDVAHVINYDMPNNIEAYTHRIGRTGRAGKTGVATTFLTLQDSEVFYDLKQMLIQSNSPVPPELARHEASKFKPGTIPDRPPRRNDTVFAH

>IbDEAD16

MAKKEKSRIHKEKEEEPNEEQHPEEEQQNAESSDDEEEEQSFEELGLDPRLVRALSKKSIEKPTPIQRVAIPLILEGKDVVARAKTGSGKTFAYLLPLLQKLFSNSPSNNNSAPSAFILVPTRELCQQVYSEAISLLELCRVQLKVVQLTSTMSISDLRTTFAGSPEILVSTPTCIQTCLSNSVLQAEALQNSLSIIVLDEADLLLSYGYEDDLKALTSHIPKRCQCLLMSATSSDDVEKLKKLILHNPYILTLPEVGDVKDDIIPKNVQQFYISCSSNDKLVHILALLKLELVQKRVLIFTNSIDMSFRLKLFFEQFGIKAAVLNAELPQNSRLHILEEFNAGLFDYLIATDDSHSEGKEQIDGRSRKEQKKSKKHPKQKLDSEFGVVRGIDFKNVHTVINFEMPQTAAGYVHRIGRTGRAYNTGASVSLVSPEEAEIIQEVKSLLGENDGNESNFIAPFPLLTKNAVESLRYRAEDVGRSVTKVAVRESRAQDLRNEILNSQKMAGVLSLLITCQSKIVGISSLRLKAHFQDNPRDLDLLKHDKTLSKKAPAPHLRDVPDYLMDPTTQEASKIVKLARAAMGNENAPRRKGGLKGKSRRSRDPLKTFSAEAPKRASKGGMKRKSKDADAGPKHKKSETRRNGAGTPICTDNGECGTRKESDADVIIVGAGVAGAALAHTLGKDGRRVRVIERDLTEPDRIVGELLQPGGYLKLIELGLQDCVDKIDAQRVVGYALFKDGKNTKVSYPLENFSSDVSGRSFHNGRFIQRMREKAAASLPNVQLEQGSVTSLLEENGTIKGVQYKTKDGQEHKAYAPLTIVCDGCFSNLRRSLCNPQVESPSHFVGLVLENCQLPYANHGHVILADPSPILFYPISSTEIRCLVDIPGQKLPSIANGEMAKYLKSVVAPQVPPELHDAFTSAVDNGNIRTMPNRTMPAAPYPTPGAVLLGDAFNMRHPLTGGGMTVALSDIVVLRDLLRPLQNLNDADALCRYLESFYTLRKPVASTINTLAGALYKVFCASPDQARKEMRQACFDYLSLGGTCSTGPVALLSGLNPRPLSLVMHFFAVAIYGVGRLLVPFPSPKRLWIGARLISAASGIIFPIIKAEGVRQMFFPATVPAYFRAPRVK

>IbDEAD19

MASLVVYCKWLVLGTVLIPSPLVILALLGSPTFLPIYATPIPIPFLNVSTVPLVAERPGGGRGHGRGGGQARPRNPVLNAHSDWKFNDLQSEEDVGKGNVNAYDDVPVETSGKDIPKPVRSFSELEFCDSLYENINRCKYVKPTPIQRYAIPVAMNGRDLMACAQTGSGKTAAFCFPIINGILSAGRVGFRNSRPASPLALILVPTRELSCQIHEEAKKFSYETGLKIVVAYGGAPISLQLRILEKGVDILVATPGRLVDMMEKSRVSLREIKYLALDEADRMLDMGFERQVRRIVQDTEMPPPGKRQTMLFSATFPAEIQAILQSLASEFLSNHVFLSAGKVGSSTDLIVQKVEFVHESGKRDILMNLLQEQTANGTQGKSDTSICGDQRGADALERWLLGNGFQAVAIHGDKEQMGANQEIPTWLNEYAEKSYCGNGERVKSYGDKKFGGFDYGNTAAYDSFSENWSSQYTETAPPYYPCTADSNPFTYGAPFAAAGYYVHPEMGSYSAAPSNYGFEQASITADDWN

>IbDEAD18

MAGPWNGSYSESTRHTRASRITYLPPHLRNSDTDTLPERFNRSARGGTARGRGHGRGGGQARPRNPVLNAHSDWKFNDLQSEEDVGKGNVNAYDDVPVETSGKDIPKPVRSFSELEFCDSLYENINRCKYVKPTPIQRYAIPVAMNGRDLMACAQTGSGKTAAFCFPIINGILSAGRVGFRNSRPASPLALILVPTRELSCQIHEEAKKFSYETGLKIVVAYGGAPISLQLRILEKGVDILVATPGRLVDMMEKSRVSLREIKYLALDEADRMLDMGFERQVRRIVQDTEMPPPGKRQTMLFSATFPAEIQAILQSLASEFLSNHVFLSAGKVGSSTDLIVQKVEFVHESGKRDILMNLLQEQTANGTQGKSLTLVFVETKRGADALERWLLGNGFQAVAIHGDKEQMERERALRSFKSGRTPILVATDVASRGLDIPHVSHVINFDLPRSIDYYVHRIGRTGRAGKSGLATAFFSGKIAPLAKDLVELMQGANQEIPTWLNEYAEKSYCGNGERVKSYGDKKFGGFDYGNTAAYDSFSENWSSQYTETAPPYYPCTADSNPFTYGAPFAAAGYYVHPEMGSYSAAPSNYGFEQASITADDWN

>IbDEAD17

MPSVLNMHLVGPMVPGASGSTVWYSTHMCGVGSLLFGDHCDGVMFDEGEGGRVEISLGSTRYWWPACEDGSKPVTGQCFETLDGGMDFYLKYVALVGFDVRHSTVKKDRDGKTSALVPQNAAHCRLRHRRHDCRLTSLQRTPNSSEHEKGEKSLKDKKEEGRIWQRMEKTRMRTWEKGVEGREEKKNLVRKREEDDEGIEEEEEEDEDERKKDIKKAVRSSGSGIMSTELFSFGLSAFRTMKLMKWGLSTTQIKPEQFHQCWKEKMFWVLQGLVLVKPLLSYQLWNCCLMKFTSQWSWSHCHLPHKELAIQTHAVAKELVKYHSQTLGLVIGGATRRGEAERIKKGVNLLIATPGRLLDHLQNTKGFVYKNLKCLMIDEADRILEANFEEEMKQIMKLVPNKGRQTALFSATQTKKVEDLARLSFQTTPIYIDVDDGRKRATNEGLQQGSTQNFFSISKLIALIFMESKAAEKRTTTFFDFCKAEKGILLCTDVAARGLDIPSVDWIIQYDPPDEPKEYIHRVGRTARGEGAKGNALLFLIPEELKFLAYLKAAKVPVKQYEFAERKLANVQSHLEKLVSNNYYLNKSAKEAYRSYILAYNAHSMKDIFNVHRLICRRCSFLFSPPKVNLNIDSSAAKFRKRHKGEGRNGFSERNPYGNKGENDQRQFGASPCQPRQTQKHPPLNAKSWPSQISCGVGVVIVLPYLRDSVVMPRPYTQNLDPSKEIVSSMVDKELSNGQYLNVHDKTESSNFILSPNILLVYHHHRLQRRCGMLLIESLDSLEPVQNHELLWSTATDHTQPKC

>IbDEAD25

MRNSWADLVENAASGNAGTSDGSGASASTRKSSYVPPHLRNKPPSAEAPAPSQGGPQSRNDRSSYGGSASGSVWNGPRSDNGRPRYGGGGRGGGAWGSRGGGWGREQEVNPFGSDDIDGDGESPSEQENSGINFDAYEDIPVETSGNNVPPSRHAIPISLAGRDLMACAQTGSGKTAAFCFPIISGIIKGSFPERPRGGRTVYPLALILSPTRELSMQIHEEAKKFSYQTGVRVVVAYGGAPMHQQDTSSKMEFDFVIPELETECSPETSILTLVLSIVKQLRDLERGVDILVATPGRLVDLLERAKVSLQLIRYLALDEADRMLDMGFEPQIRRIVEQMDMPPPGERQTMLFSATFPREIQRLASDFLSNYVFLTVGRVGSSTDLIVQRVELVQNNDKRSHLMDLIHAQKANGVNGQQALTLVFVETKRGADALEGWLCSNGFPATSIHGDRSQQERELALRSFKRGDTPILVATDVAARGLDIPHVAHVINFDLPNDIDDYVHRIEELAEANQEVPDWLTRYASRSYSGGRNNRRPGGNRFGGRDFRRDSFSRGGSGKDFYGGGSGGGGGGGYGGYGGGGYGSGGANSAWD

>IbDEAD22

MAGMAAEGSQFDARQFDAKMNEILETDAQEFFTSYDEVYDSFDSMGLQENLLRGIYAYGFEKPSAIQQRGIVPFCKGLDVIQQAQSGTGKTATFCSGILQQLDYSLVECQALVLAPTRELAQQIEKVMRALGDYLGVKVHACVGGTSVREDQRILQSGVHVVVGTPGRVFDMLRRQSLRPDNIKMFVLDEADEMLSRGFKDQIYDIFQLLPPKIQVGVFSATMPPEALEITRKFMNKPVRILVKRDELTLEGIKQFYVNVEKEEWKLETLCDLYETLAITQSVIFVNTRRKVDWLTDKMRGRDHTVSATHGDMDQNTRDIIMREFRSGSSRVLITTDLLARGIDVQQVSLVINYDLPTQPENYLHRIGRSGRFGRKGVAINFVTKDDDRMLFDIQKFYNVVVEELPANVADLL

>IbDEAD23

MAYTSSIIGVSSIFQANPSLEHSKRPTSTPPLSFPQSFEKLHFNPLKAFSSSSLVRSKQCSSSSSSPFVASAVVTPNSSLLSEEAFKGLGGFAKDSLNVSDAEYDSELDEDESELGAADSDDELAISKLGLPRQLVETLEKRGITNLFPIQRAVLIPALEGRDIIARAKTGTGKTLAFGIPIIKRITEDEQSKFSQRRGRLPRVLVLAPTRELAKQVEKEIKESAPHLNTACIYGGVSYVTQESALSRGVDVVVGTPGRLIDLINNNSLKLGEVQYLVLDEADQMLAVGFEEDVEVILEKLPSQRQSMLFSATMPGWVKKLARKYLNNPLTIDLVGDEEEKLAEGIKLYAISTTATSKRTILSDLITVYAKGGKTIVFTQTKKDADEVSMALTNIIPSEALHGDISQHQRERTLNGFRQGKFTVLVATDVASRGLDIPNVDLVIHYELPNDPETFVHRSGRTGRAGKEGNAILMFTSSQRRTVRSLERDVGCRFDFVSPPSVNEVLGSSAEQVVVSLGGVHSESIEYFMPTANKLMEEQGVNALAAALALLGGFAKPPSSRSLITHEQGWVTLQLTRDTGFSRGFMSARSVTGFLSDVYSAAADEVGKIQIIADERVNGAVFDLPEEVATELLNQPLPPGNTITKITKLPALQDDGPPSDFYGRFSNRDRGSRGGGFRDRRGGRGASRSWSSGRFSDNDSDDLDDGFRRGGRSRRGGSDLDGLDDGFRRGGRSRRGGSDWLIGDMRSNRSSSAGGNRDRNSGGACFNCGRTGHRASECPSKRAVAIRTDGCRKLLNLPYLH

>IbDEAD26

MADNTTSVAASSAMEEEMKPQLSKSWADVADETEEAEASSTSDVKPNSELKMDSLAMDESKSVYKTLSDPDDASIEAVTSGDTLYKSAKRFEDLNLTPELLKGLYVEMKFERPSKIQEISLPMILTPPNKNLIAQAHNGSGKTTCFVLGMLSRVDPKLAAPQALCICPTRELAIQNMEVLLKMGKYTGITSELAIPADAANYIPINKRPPVTAQVIIGTPGTINKWVIAKKLGTSCMKILVFDEADHMLAESGFQDDSIKIMKAIVRGKADCQVLLFSATFNETVKGFVTKIVKDLFVKDYNQLFVKKEELSLESVKQYKVQCPDELSKVMVIKDKILELAQKVGQTIIFVRTRNSAHMLHKSLVDYGYEVTTIQGALKQEDRDKIIKEFKEGLTQVLISTDLLARGFDQSQVNLVVNYDLPVQYGHWSDPDYEVYLHRIGRAGRFGRKGAVFNLLCNDTDTMLMSKIENHFNSPVAEVTSWQSDTEFEAALKTAGLL

>IbDEAD27

MAENGEEDKSFKELGVCDQLVEACESLGWKTASKIQAEAIPHALEGKDIIGLAQTGSGKTGAFAIPILQALIDSPQAFFACVLSPTRELAIQISQQFEALGAGVGVKCAALVGGLDQVQQSIMLGKRPHILVATPGRLLDHLSNTKGFSLRTLKYLVLDEADRLLNEDFEKALDEILNNTPRERRTYLFSATMTKKVRKLQRACLRNPVKIEAASKYSTVDTLKQQYRFIPAKYKDCYLVYVLTEMSGSTSMVFTRTCDATRLLALMLRNLGLRAIPISGQMSQGKRLGALNQFKAGESNILICTDVASRGLDIPSVDVVINYDIPTNSKDYIHRVGRTARAGRSGVAISLVNQYELQWYLQIEKLIGKKLPDYPAPEEEVLLFLDRVTEAKRISITKIKETGGKKKRRGDDEDGIDGYLDRKHGKSSKKPKRK

>IbDEAD30

MAATATASSAGPRYAPEDPTLPKPWRGLVDGKTGYLYFWNPETNVTQYERPGGSAPSNKSSSGPVSSSIQKSSQGQHPDNGDDDRYDRGSNGGSSRLSTGAEGGSSDHYRDVSQMPEAGGGPSAPKSNSGLSMGSDLSPDSYRRQHEISVTGDNVPPPFTSFKATGFPSELLREGFLNHPCSWLGMLLVMIPSATLSKWKKRSPPALACCSIGCLCKGDVSSCAGQGHAKTSNSRGTLKLVHPAQSWPIALQGRDIVAIAKTGSGKTLGYLIPGFMHLKRRNNNPRMGPTVLVLSPTRELATQIQDEAVKFGTTSRISSTCLYGGAPKGPQLRELDKGVDIVVATPGRLNDILEMKRVTLHQVSYLVLDEADRMLDMGFEPQIRKIVKEVPSQRQTLMYTATWPKDVRKIAADLLVNPVQVNIGNVDELVANKSITQVVALLVFEAPLPHHHHILSMHEKVKKVDAFQFPTLLQYVEVLQSMEKRRRLEQILRSQEPGSKIIIFCSTKKMCDMLAGNLNNKFGAAAIHGDKSQSDRDYVLSQFRTGRSPVLVATDVAARGLDVKDIRVVINFDFPTGIEDYVHRIGRTGRAGATGVAYTFFGDQDGRYASDLIKILEGASQRVPDELRDIASRGGGMGRARRQWSAAPGGRDGGNSGQDSGRAGWSMSFSSDKGGGHGNNRDGGNSGQDSGRAGWSMSFSSDKGGGHGNNHDSYDSDRYGNSSQDADGSGSFQACSFHERMMKASEKPRRSPSPSRSPNRGSGWGGDNNSRGRSRSRSVERFDKPSPVGQEPIGGSFHETMMAKAGTSLVVQNLMLPTWDSVNVIDSGKNLNDDPKGCESSPPKQQNDDQYADGSCLNYGEEGNIAKEDSTFQDASENPSPTHP

>IbDEAD28

MAGAAPEGSQFDARQYDAKMQELLTVEGGQEFFTSYEEVHDSFDAMGLKENLLRGIYAYGFEKPSAIQQRGIVPFCKGLDVIQQAQSGTGKTATFCSGVLQQLDYETVECQALVLAPTRELAQQIEKVMRALGDYLGVKVHACVGGTSVREDQRILSSGVHVVVGTPGRVFDMLRRQSLRPDYIKMFVLDEADEMLSRGFKDQIYDIFQLLPPKIQVGVFSATMPPEALEITRKFMNKPVRILVKRDELTLEGIKQFYVNVDKEEWKLETLCDLYETLAITQSVIFVNTRRKVDWLTDKMRGRDHTVSATHGDMDQNTRDIIMREFRSGSSRVLITTDLLARGIDVQQVSLVINYDLPTQPENYLHRIGRSGRFGRKGVAINFVTKDDERMLFDIQKFYNVMVEELPA

NVADLL

>IbDEAD29

MKKPTPVQRHCIPRILAGQDVLGIAQTGSGKTAAFALPLLHLLAQDPYGVFALVVTPTRELAYQLAEQFRALGSCLNLRCVEVVGGMDSINQAKTLMRRPHVVIATPGRIKTLLQQNPDIPSIFSKTKFLVLDEADRVLDVGFEEELKVIFQCLPKNRQTLLFSATMTSELQTLLELSVNKAYFYEAYEGFKTVESLKQQYLLTPSNIKEVSLYCVLSKMEDMGIRSAIVFVSTCRTCHFLSLLLEELDLEAAALHSFKSQSLRLSALHKFKSGKVPILLATDVASRGLDIPTVDLVINYDLPRYPRDYVHRVGRTARAGRGGLAASIYDVELLHEIEAVLGKQLEEFECKDKDSNDNITKVFKARRVAIMKMMDDGFEEKAKSRKAQKLKMLEEKGLLKNKKRKRDRTGMQ

>IbDEAD31

MGGGGRGDGGLNYNANLAFQPRNPHQYVQRSPLPNQQWLRRNNQLTADSAVDEVEKTVQSEAIDSSSQDWKAQLRLPPPDTRYRTEDVTATKGNEFEDYFLKRELLMGIYEKGFERPSPIQEESIPIALTGSDILARAKNGTGKTAAFCIPALEKIDQDKNAIQCIILVPTRELALQTSQVCKELAKHLQIEIMVSTGGTSLKDDIMRLYQPVHLLVGTPGRILDLVRKGVCILKDCSMLVMDEADKLLSPEFQPSIGQLIRFLPANRQILMFSATFPVTVKDFKDRYLQRPYVINLMDELTLKGITQFYAFVEERQKVHCLNTLFSKLQINQSIIFCNSVNRVELLAKKITELGYSCFYIHAKMLQDHRNRVFHDFRNGACRNLVCTDLFTRGIDIQAVNVVINFDFPKNSETYLHRVGRSGRFGHLGLAVNLITYEDRFNLYRIEQELGTEIKQIPPHIDQEFGELLSVKVPNVLGAYFEWKLRCQCWICCA

>IbDEAD32

MAANINMEIEEDDDYVEYIPVAKRRALEAQKILQRKGQSAALEEEAEKIKQVEAKPSLLVKATQLKKELPEVTETEQMVQQEKEMIEHLSDRKTLMSVRELAKGITYTEPMFTGWKPPLAIRRMSRKACDTIRKQWHIIVDGDDIPPPIKNFKEMRFPEPILEKLKAKGIVQPTPIQVQGLPVILSGRDMIGIAFTGSGKTLVFVLPLIMVALQEEIMMPIVPGEGPFGLIICPSRELARQTYEVIEQFLEPLREIGYPELRPLLCIGGVDMKSQLDVVKKGVHIVVATPGRLKDMLAKKKMNLDNCRYLTLDEADRLVDLGFEDDIREVFDHFKAQRQTLLFSATMPTKIQNFARSALVKPITVNVGRAGAANLDVIQEVEYVKQEAKLVYLLHCLQKTPPPVLIFCENKADVDDIHEYLLLKGVEAVAIHGGKDQEEREYAISSFKSGKKDVLVATDVASKGLDFPDIQHVINYDMPAEIENYVHRIGRTGRCGKTGIATTFINKTQSPTTLLDLKHLLQEAKQRIPPVLAELENPMADDDAIMNASGVKGCAYCGGLGHRIRDCPKLEHQKKPTDCQSKKGLFWICFTAVKVVLWLMLHLKFHVSCIDEKYELH

>IbDEAD34

MPFGNFLPRMHKFNVDVSCKIQFALMELENLLSSWGKSLKDFPEMTIPDESSMGLSENMLIAEELAYDKESLKTEHETLTFVWRTLSSRIRSRGDIVLNVTSSGIASLLLPGGRTTHSRFAIPLSLNEDSTCNISQGSDLPELIIRSKLIIWDEADGHKSCIEALDKIMRDILSVDHDPIATIVESTFPSSRYGMLDESQLEGRAIFSSTLDVVDQINQYMCDMNTVEGRTYLSCDSLCKVGSGGENLSEVHTPKFLNSLRLSGLPNHSLTLKVGAPVMLLRNIDHSLGLCNKTRRPKFLSPGCLLVLLIRDCPSSSSVNNSR

>IbDEAD33

MNARGRYSPGTGNGRGGGGDGYGGGGFQSNHNSRGGYRQGRNPHSQPQQYDKRSLQNEPHQQQQQWLRRNASAAPPESSYNEVRKNIQSGGIDSSSNVWKARLNIPPPDTRYRTEDVTATKGNEFEDYFLKRELLMGIYEKGFERPSPIQEESIPIALTGSDILARAKNGTGKTAAFCIPALEKIDPDNNGDLSPSVSPLNVLLFDCNVVILVPTRELALQTSQVCKELGKHLKIQVMVSTGGTSLKDDIMRLYQPVHLLVGTPGRILDLAKRGVCILKDCAMIAMDEADKLLSPEFQPSVEQLIMFLPQHRQILMFSATFPVTIKDFKDRYLQKPYIINLMDELTLKGITQFYAFVEERQKVHCLNTLFSKLQINQSIIFCNSVNRVELLAKKITEIGYSCFYIHAKMLQDHRNKVFHDFRNGSCRNLVCTDLFTRGIDIQAVNVVINFDFPKNSETYLHRVGRSGRFGHLGLAVNLITYEDRFNLYKIEQELGTEIKPIPPQIDQAIYLPRGGFLSSYSHDFSWMDMDRGGDGVVNLLIKLTT

>IbDEAD36

MAKGTGILVSSKAELKRKQKEQKKSKSGGFESLGLSHNVFRGIKRKGYRVPTPIQRKTMPLILAGNDVVAMARTGSGKTAAFLVPMLEKLQQHVPQAGTRALILSPTRDLALQTLKFTKELGRFTDLRVSLLVGGDSMESQFEELAQSPDIIIATPGRLMHHLSEVDDFSLRSVEYVVFDEADCLFSMGFAEQLHTVLTHLSENRQTLLFSATLPSALAEFAKAGLRDPQLVRLDLETKISPDLKLTFFTLRHEEKHAAILYLIRELIRSDEQTLIFVATQYHLEFLSILLRDEGIESSVCYGKMDQDARNIHISKFRARKTMLLIVTDVAARGIDIPLLDNVINFDFPCKPKLFVHRVGRVARAGRTGTAYSFVTPEDMPYLLDLHLFLSKPIRAAPTEEEVLQDIDGVMSRMEQATANGETIYGRFPQTALDLLSDRVREIIESSAELISLQKPCKNSFRIYSKTKAKPARESVKRVKDLPREGLHLLFKNLLGGNELTALAFSERLKTFRPKQTILEAEGEAAKSKHQGSQWVDVMKMKRAIHEEVINKVRQQRCSGDHVSKDETEECISSQVKEKPVSGSKRKAKSFKDDEYFISAVPTNQHFEAGLSVKANQGFESNRLDAAVLDLVADDKQGLHKQKLHYHWDKRSKKYIKLNNGDRVTASGKIKTEGGAKVKANKTGIYKKWKEQSHKKVSLKGTNMDDSFGESTSSAGKGIVVNASPRCPGSILDAQPRLKPDVYTLLAGLKSAALTTFD

>IbDEAD39

MRSSWADSVENVPSDNAGTSGGSGVSGRQNYVPPHLRNNAPAAKTPAPSHSAPIFTNDRSGYSGPGSGSRWGGPRQGYGGGGRGGSGWGSRGGGRGREQEVNPFGNDDIDGAAETANPQETTGINFDAYEDIPVETSGDNVPPAVNTFAEIDLGDALNLNIRRCKYVKPTPVQRHAIPISLAGRDLMACAQTGSGKTAAFCFPIISGIMKGNNLQKPHGVRTVFPLALILSPTRELSMQIHEEAKKFAYQTGVRVVVVYGGAPITQQLRELERGVDILVATPGRLVDLLERAKVSLQMIRYLALDEADRMLDMGFEPQIRRIVEEMDMPPPGERQTMLFSATFPREIQRLAADFLSNYIFLAVGRVGSSTDLIVQRVELVQDNDKRSHLMDLLHAQKANGIHGKQALTLVFVETKRGADALEHWLCSNGFPATAIHGDRTQQEREQALRAFKRGDAPILVATDVASRGLDIPHVAHVINFDLPNDIDDYVHRIGRTGRAGNTGLATAFFNENNVSLAKALAELMQEANQEVPDWLTRYASRSYGGGRSKRSGGGRFGGRDFRRDSSFGRGRSGGDYYGGGNYGGGYGNSSGGGNYGGGGYGNSSGGGYGGGYGNSSGGGYGSGSAWD

>IbDEAD35

MSGATVMAKMGGTGRALLGYTFIIPSTAKSLSFRRHFYSLPLNPHRVLQRFRPLCSSATFTAEEDVLQPVRHGILLEKLRLRHLKDSSKAGGTTPVGKLKEAKGNGHVELEGRSGKKKAVEASSFEELGLSEEVMGALEEMSISLPTEIQAIGIPAVLRGKSVVLGSHTGSGKTLAYLLPIVQPSSCCFVPNTELCEQVFRVAKSISHHARFRSTMVSGGGRLRPQEDSLNAPIDMVVGTPAEFFNILRRGTWFMVLDEADTMFDRGFGPDIRKFLRPLKTRAAKSDDQGFQTVLVTATMTKAVQKLVDEEFQGIEHLRTSSFHKKIASARHDFIRLSGSENKLEALLQVLEPSLSKGNRVMVFCNTLNSSRAVDHFLNENQISTVNYHGEVPAEQRVENLEKFKRDDGDCPTLVCTDLAARGLDLDVDHVIMFDFPLNSIDYLHRTGRTARMGAKGKVTSLVAKRDLLLATRIEEALKKNESLESLSVDSIKRDIARSNITEQKEKKEKLVKASNSKNKKQSSSTKLPSTSGKGIAVKKSSKAISRKAPTPSSQKCNQSVKDIKWEAKKCNQSVKHIKWEETVNSVKAKNCNKIVQVTKREDTIREPEARFKTKCCWIQGSEFVVKFKGCLIYLAFWS

>IbDEAD38

MQSVDTFRGTCTHQGPSLLRGNPSLNEKELIVRIDRSGGSRSIQRIKYGARDGDVKLCSAYDVTQALFDLDSQEFRSFRKHSFILKSQHTTILKNPQVIPDLVTISELYTRDKVSEGVLKCFKCNSTWENGVLRFKVQLRVVDMKGNASFMLWDRDAFELIGIAASDLHELHKDASKAILDLVNKCMLFVISKDRKHLSKPDSAFPAFKVNIDQQLTNKIASRFLAFRFSSDEADSPIATLPPLSGEATEGSVKRSLIDRSPQLKGAGRINNEAAVRDYYYRSEKEHPSQDKENALQQTPNTADLHTPQNTKGSQSSNPLVFGVFGNSLNSPLSIVSKSGNLIKSSIRDGKGSEHILSVNKETHSQPLEYENFPLSDVTNVQQYKGKYPQRLYDSSLSRHTLTGQGHALITPSAPASILRAKDRCSQPVEIDRLPLSDLTNDLDTQDSEIVRVHLSDVTNEQKFNAKLHVDIVEAIKQDLDEHNVLVKSFRLAKAQMQTNPTVDYKMRLIGKRSGDARTYNLPTVSEVAALVVGDLDPRAGQEIFWSRLKSGTLKRINELNPKIFVTQYPILFPYEKMDIGRILILILQGVKVLVAESVYLNESSLHTRYMIANKTYEAAKQLAYIDMPTKFVWKKDQSGTKEKGFSIGGIFYVLRAGKRLYGRRSAKIVIKGDIVLNVASSGIASLLLPGGRTAHSRFAIPIVLNEDSTCNITQGSHLAELITMAKLIIWDEAPMMHKHCFEALDDNARYHEGVTEDIVRTINSSYLWESCKVLRLTKNLRLNTPERPESISKNFKNSQLATCIFESGDAPKSRSLVTPMLGNGMLADMHTPEFLNGLKASGIPNHTLTLKVGSPVMLLRNIDHSLGLCNGTRSVITRLARSCS

>IbDEAD40

MYQNLSQQLLSFTVMAGLAAEGSQFDARQYDAKMTELLDTDGQEFFTSYDEVYESFDSMGLQENLLRGIYAYGFEKPSAIQQRGIVPFIKGLDVIQQAQSGTGKTATFCSGILQQLDYSLVECQALVLAPTRELAQQIEKVMRALGDYLGVKVHACVGEQSLSPDHIKMFVLDEADEMLSRGFKDQIYDIFQLLPPKIQVGVFSATMPPEALEITRKFMNKPVRILVKRDELTLEGIKQFYVNVEKEEWKLDTLCDLYETLAITQSVIFVNTRRKVDWLTDKMRSRDHTVSATHGDMDQNTRDIIMREFRSGSSRVLITTDLLARGIDVQQVSLVINYDLPTQPENYLHRIGRSGRFGRKGVAINFVTKDDERMLFDIQKFYNVVVEELPANVADLL

>IbDEAD37

MAKGTGILVSSKAELKRKQKEQKKSKSGGFESLGLSHNVFRGIKRKGYRVPTPIQRKTMPLILAGNDVVAMARTGSGKTAAFLVPMLEKLQQHVPQAGTRALILSPTRDLALQTLKFTKELGRFTDLRVSLLVGGDSMESQFEELAQSPDIIIATPGRLMHHLSEVDDFSLRSVEYVVFDEADCLFSMGFAEQLHTVLTHLSENRQTLLFSATLPSALAEFAKAGLETLSYILLRDEGIESSVCYGKMDQDARNIHISKFRARKTMLLIVTDVAARGIDIPLLDNVINFDFPCKPKLFVHRVGRVARAGRTGTAYSFVTPEDMPYLLDLHLFLSKPIRAAPTEEEVLQDIDGVMSRMEQSTANGETIYGRESVKRVKDLPREGLHLLFKNLLGGNELTALAFSERLKTFRPKQTILEAEGEAAKSKHQGSQWVDVMKMKRAIHEEVINKVPTAAVAIMCLRMRLKNAFLPKSRRNQCSEELPIDIFSGSKRKAKSFKDDEYFISAVPTNQHFEAGLSVKANQGFESNRLDAAVLDLVADDKQGLHKQKTTYHWDKRSKKYIKLNNGDRVTASGKIKTEGGAKVKANKTGIYKKWKEQSHKKVSLKGTNMDDSIGESTSSAGRGIVVKALPRCPVGGDRIPNELASPEMGGLGVKGGNMKFQRGRKNNRSIPNAHVRSEIKDHEQIRKEREKKANRIAHLKSKPSKGKKFKRSGKKGGKGRQSN

>IbDEAD42

MPTSWADSVSAAEGAAAVASSNSTKQAYVPPHLRNKPATSDPPKQPATGSYASAAAAVAVAASGNDRVHYGGPPQQGGSRWNGPRNEFRGGYGGGGGGDQPFDEQENSGINFDAYEDIPVETSGDNVPPPVNTFAEIDLGDAVNNNIRRCKYVKPTPVQRYAIPIALAGRDLMACAQTGIHEEARKFAYQTGVRVVVAYGGAPINQQLRELERGVDILVATPGRLVDLLERARVSLEMIRYLALDEADRMLDMGFEPQIRKIVQQTDMPPPGVRQTMLFSATFPKEIQRLAADFLNYIFLAVGRVGSSTDLIVQRVEYVQETDKRSHLMDLLLAQMANGANGKQCLTLVFVETKKGADSLEHWLCINNFPATAIHGDRTQQEREQALRAFKSGRTPILVATDVAARGLDIPLVAHVVNFDLPNDIDDYVHRIGRTGRAGKTGLATAFFNEGNSSIARSLADLMQEANQEVPAWLSRYAARSSHGGKSRRVAGRFGGRDFRRESNFNRGNPDYYSGGNMGGGYSGFGGGYNPSYGAGVASAWD

>IbDEAD41

MSFQHSFSVLRLCTLSPSTPKLLSFRTCSIAKLQFFRIRAFGTAAVAADKNGGDTFFAEENVSWTSLGVSERLSRSLSSIGLHRPSLVQAACIPSILSGADVVVAAETGSGKTHGYLVPLIDKVCQSSGISGEGVVGREANKSRQLSLVLCPNVMLCEQVVRMANCLRNDGGEPVLRVAAVCGRQTWPVKEPDIMVSTPAAILNYLSTIEAENHGRSDFIRCVKHVVFDEADMLLCGSFQNQVIRLLNMLRFDEKQLSRLRNSEAEIVTGSIPDSPLGSDQEDNRDLLADFSKEVDDTEDDARVMDSEVDSKVAKRTDWRRVRKTYERSKQYIFVAATLPVNGKKTAGAVLKRMFPDASWVSGNYLHCHNPRLEQKWIEVTIDTQVDALISAVRNGIQSALNDGSGILRTMVFANTVDAVEAVAKVLTGAGIECFCYHSNSSLEERTKNLISFQQKGGVFVCTDAAARGLDVPNVSHVIQAEFATSAVDFLHRVGRTARAGQPGLVTSLYNESNRDLVATVRLAEKRGQPVEKAFSRKRSFRNKLKKRGFSNMNDVESVEENALA

>IbDEAD43

MMKEEEDERKKEIKKAVRSSGSGIMSTELFSTLGLSELTMKAINEMGFEYMTQIQARAIPPMLEGKDVLGAARTGSGKTLAFLIPAVELLFNVKFTHRNGAGVIVICPTRELAIQTHAVAKELLKYHSQTLGLVIGGATRRGEAERIKKGVNLLIATPGRLLDHLQNTKGFVYKNLKCLMIDEADRILEANFEEEMKQIMKLLPKEGRQTALFSATQTKKVEDLARLSFQTTPIYIDVDDGRKRATNEGLQQGYCVIPSAKRFTLLYSFLKRNLSKKIMVFFSSCNSVKFHSELLQYIQIDCFDIHGKQKQQKRTTTFFDFCKAEKGILLCTDVAARGLDIPSVDWIIQYDPPDEPKEYIHRVGRTARGEGAKGNALLFLIPEELKFLAYLKAAKVPVKQYEFEERKLANVQSHLEKLVSNNYYLNKSAKEAYRSYILAYNSHSMKDIFNVHRLDMQAVAASFCFSSPPKVTLNIDSSPAKFRKKHKGEGRNGFSERNPYGNKGENDQRQFVRY

>IbDEAD47

MSRYDSRAADPGSYRDRRSDSGFGGGSTYRSSSASRKDYEGSESPRKLDLDGLTPFEKNFYVESRSVAAMTDSEVEEYRRRREITVEGRDVPKPVKSFSDVGFPEYVLKEIEKAGFTEPTPIQSQGWPMALKGRDLIGIAETGSGKTLAYLLPAIVHVNAQPFLAQGDGPIVLVLAPTRELAVQIQQESTKFGASSKIKSTCIYGGVPKGPQVRDLQKGVEIVIATPGRLIDMLESHHTNLRRVTYLVLDEADRMLDMGFEPQIRKIVSQIRPDRQTLYWSATWPKEVEQLARQFLYNAYKVIIGSEDLKANHAIRQHVEIVSESQKYNKLVKLLEDIMDGSRILIFMDTKKGCDQTTRQLRMDGWPALSIHGDKSQAERDWVLSEFKAGKSPIMTATDVAARGLDVKDVKYVINYDFPGSLEDYVHRIGRTGRAGAKGTAYTFFTAANARFAKDLIHILQEAGQKVSPELAAMGRGAPPPPPGGHGGFEIVAEVMEVVAGGIEVELMDFHYSHLQIGTSP

>IbDEAD44

MPEISKVDSAKRKRIRKRDSRKKHSMTDGSLTVAKEEVDKLKENENYGIEHEKGEEKGEETSLKKDMKKKRKDLAKRMEKDEDENLGEQGSKEEKKKKNLVRKREEDDEGREEEEEEEEEEEEEEEEEEEEEVEDERKKEIKKAVRSSGSGIMSTELFSTLGLSELTMKAINEMGFEYMTQIQARAIPPMLEGKDVLGAARTGSGKTLAFLIPAVELLFNVKFTHRNGAGVIVICPTRELAIQTHAVAKELLKYHSQTLGLVIGGATRRGEAERIKKGVNLLIATPGRLLDHLQNTKGFVYKNLKCLMIDEADRILEANFEEEMKQIMKLLPKEGRQTALFSATQTKKVEDLARLSFQTTPIYIDVDDGRKRATNEGLQQGYCVIPSAKRFTLLYSFLKRNLSKKIMVFFSSCNSVKFHSELLQYIQIDCFDIHGKQKQQKRTTTFFDFCKAEKGILLCTDVAARGLDIPSVDWIIQYDPPDEPKEYIHRVGRTARGEGAKGNALLFLIPEELKFLAYLKAAKVPVKQYEFEERKLANVQSHLEKLVSNNYYLNKSAKEAYRSYILAYNSHSMKDIFNVHRLDMQAVAASFCFSSPPKVTLNIDSSAAKFRKKHKGEGRNGFSERNPYGNKGENDQRQFVRY

>IbDEAD45

MQPLGQGKQWHTWLQLFISCRNVNLGFNALMCNRKQITRLVDVLFDSCLNLQQVYEILQKMLHRFHWIVPGYIMGGENRSKEKARLRKGISILVATPGRLLDHLRKTSSFIHINLRWIVFDEADRILELGYGKEIEDILNVLGSFGKEKTAFRGLEVQRQNLLLSATLNEKVNHLAKISLDNPIMVGLEDKKSDNKQNHEQMETSGLNLNDEFETSGKLLSSSCEEYKLPAQLLQRYIKVPCGSRLVVLLSILKHLFEKEASTQKVVVFFSTCDAVDFHHLLLSEFLWNPSLQSDLEVRQKFLSCNILRLHGNMNHDDRRTAFNAFKTEKTALLLSTDVAARGLDFPKVRCIIQYDSPGEATEYVHSAFCFKCATDTFLNRAAGSRLFPPAVHDCINPLSELITSSKSSVKLHIVGRTARLGEKGDSVLFLQPIEVEYLQDLEKHGVTLTEYPLLKLLDSFPVFGMKHLSKKFVSIEMHPWSIFLQRSLESFVTTEANLKKLAKNAFCSWVRAYTAHRGDLKRIFMVKKLHLGHVAKSFALKEQPSLVNKSIQKQTKKRGRDQKQKGVSSKKRKTTK

>IbDEAD46

MFIGCSVLSRVGFLLELILDPYLLFPGNIVRCGLVEIGEISMAGLAPEGQQFDARQFDAKMSELLTVDGQDFFTSYDEVYDSFDSMGLQENLLRGIYAYGFEKPSAIQQRGIVPFCKGLDVIQQAQSGTGKTATFCSGILQQLDYGLIQCQALVLAPTRELAQQIEKVMRALGDYLGVRVHACVGGTSVREDQRILMAGVHVVVGTPGRVFDMLRRQSLRPDCIKMFVLDEADEMLSRGFKDQIYDIFQLLPPKIQVGVFSATMPPEALEITRKFMNKPVRILVKRDELTLEGIKQFYVNVEKEDWKLETLCDLYETLAITQSVIFVNTRRKVDWLTDKMRGRDHTVSATHGDMDQNTRDIIMREFRSGSSRVLITTDLLARGIDVQQVSLVINYDLPTQPENYLHRIGRSGRFGRKGVAINFVTEDDERMLFDIQKFYNVTIEELPSNVADLL

>IbDEAD49

MDDESISTAASATAEEMKPQLSNMEASSNSDVKPNSELNMDSLAMDESQSVHKTLSDPDDASIEAVTSGDTLYKSAKRFEDLNLTPELLKGIYVDMKFERPSKIQEISLPMILTPPNKNLIAQAHNGSGKTTCFVLGMLSRVDPKLVAPQALCICPTRELAIQNMEVLLKMGKYTGITSELAIPADAANYVPINKRPPVTAQVIIGTPGTINKWVIAKKLGTSYMKILVFDEADHMLAESGFQDDSIKIMKAIVRGKADCQVLLFSATFNETVKGFVTKIVKDLFVKDYNQLFGKKEELSLVSVKQYKVQCPDELSKVMVIKDKILERAQKVGQTIIFVRTRNSAHMLHKSLVDYGYEVTTIQGALKQEDRDKIIKEFKEGLTQVLISTDLLARGFDQSQVNLVVNYDLPVQYGHWSDPDYEVYLHRIGRAGRFGRKGAVFNLLCNDTDTMLMSKIENHFNSPVAEVTSWESDTEFEAALKTAGLL

>IbDEAD48

MGSLLLRKSSSSLPSKKLALVALSRLFSPTLSTSETVISRNGGSIHQFHNFATASNFPAVGSSSAAAAAASPRQIVRGLHISRPLAAGAAVARLPEEDEGLEISKLGISQEVVSALARRGITQLFLFRSSIEPKNLEIFFSVYSYKFRAVLEPAMQGIDMIGRARTGTGKTLAFGIPIMDKIIRYNEKHGKGRNPLALILAPTRELAKQVDKEFYESAPNLDTLCVYGGVPIGKQMGALDRGVDMVVGTPGRIIDLLKRGSLDLSELQFVVLDEADQMLNVGFAEDVERILQNAPKNRQTMMFSATMPGWILKLTQQFLKNPVEIDLVGNSDQKLADGISLYSIACEMHQKPAVLGPLITEHAKGGKCIVFTQTKRDADRLASAMQRNFKCEALHGDISQNQRERTLSGFREGHFNVLVATDVAARGLDVPNVDLVIHYELPNSSEIFVHRSGRTGRAGKKGSAILMYSNQQARDAKGIEREVGCKFIELPRIEVDAGAANMFSDIGLGNSRFGSNGGFGSGRSGGGRFGGSSQGSSYGQVGGFGRSGGYGGGRSGNFGDFGNRQSGNRNSGGFEGMNSSNLSGGFGNSRGPAKHGGGFSSFGNSGSSFGSSRPSGFGGFGDNKDSNNQSYGRKHF

>IbDEAD51

MGRKHHDTAILSEPLTSGTIENHDTKSKKKHKRKLEHPEAEPEPEIARESNKEKKKKKKHKHKADEEAKDHVGEIEELNGSVDISGKANNKKENGEGVEVSNNGSVEIMESSEGVVVSGKDVNDLKFKALEKFSDSGLPDKVLECCKNFEKPSPIQSHSWPFLLNGRDFIGIAATGSGKTLAFGIPGIMHVMSKRKGKKPKNPLCLVLSPTRELAQQISDVLCDAGKPTGTQSVCLYGGTSKGPQISALKSGVDIVIGTPGRLKDLIEMGICQLKEVSFVVLDEADRMLDMGFEPEVRSILSQTCSVRQMVMFSATWPPPVHQLAQEFMDPNPVKVVVGSEDLAANHDVMQIVEVLEDRARDERLQNLLEKYHKSRRNRVLVFVLYKKEASRVENMLQRRGWKVVSISGDKAQHARTEALSLFKEGSCPLLIATDVAARGLDIPDVEVVINYSFPLTTEDYVHRIGRTGRAGKKGVAHTFFTKENKGLAGELVNVLKEAGQIVPADLLKFGTHVKKKESKLYGAHFREITPNAAKATKIKFDNSDDED

>IbDEAD53

MAAAKVSIFSHDGTPIPSTRRRARKLERTRILPLNFPHSYCTTRFCVSQALNADLVYTKSTRSRTRPQEAADAELDAISVLNHRIRRDLAKRDQLSITRPVMDADEADKYLQLVKEQQQRGLQKLKAGRARAVDTATPSTKYQVFSYKVDPYTLRSGDYVVHKKVGVGRFVGIKFDVSKDSSIPIEYVFIEYSDGMAKLPVKQASRMLYRYNLPNESKKPQALSKLSDPSAWERRRLKGKIAVQKMVVDLMELYLHRLKQKRPLYPKTAALAEFASQFPYEPTPDQKQAFLDVERDLTERENPMDRLICGDVGFGKTEVALRAIFCVVSAAKQVMVLAPTIVLAKQHFDVISDRFSRYPHITVGLLSRFQTKSEKEEYLHMIKHGHLDIIVGTHALLGSRVEYNKLGLLVVDEEQRFGVKQKEKIASFKTSVDVLTLSATPIPRTLYLALTGFRDASLISTPPPERVPIKTHLSAYMKEKVISAIKFELDRGGQVFYVFPWIKGLEDVMDFLEEVFSHVEIAIAHGKQYSKQLEETMERFARGDIKILICTNIVESGLDIQNANTIIVQDVQQFGLAQLYQLRGRVGRADKEAHAYLFYPDKSLLSQQALERLAALEECCHLGQGFQLAERDMAIRGFGNIFGEQQTGDVGNVGIDLFFEMLFESLSKVDEHRVISVPYHSVKLDIDVNLHLPSEYINHLENPMEIISEAEKGAEKDMWNLMQFTENLRRQYGKEPYSMEILLKKLYVRRMAADLGITRIYALGKIVGMKTNMTKNVFKLITESVASDVHRNSLTFEDGEIKAELLLELPREQLLNWVFQCLAELYASLPALIKY

>IbDEAD52

MQITFSASPAAAAAQTSGGGFPSSLSSKHCSPYPLAANINMEIEEDDDDYVEYVPVAKRRALEAQKILQRKGKSAALEEEAENINQVEAKPSLLVKATQLKKELPEVSETEQMVQQEKEMIEHLSDRKTLMSVRELAKGITYTEPMFTGWKPPLAIRRMSKKACDTIRKQWHIIVQGLPVILSGRDMIGIAFTGSGKTLVFVLPLIMVALQEEIMMPIVSGEGPFGLIICPSRELARQTYEVIEQFLEPLREIGYPELRPLLCIGGVDMKSQIDVVKKGVHIVVATPGRLKDMLAKKKMNLDNCRYLTLDEADRLVDLGFEDDIREVFDHFKAQRQTLLFSATMPTKIQNFARSALVKPITVNVGRAGAANLDVIQEVEYVKQEAKLVYLLHCVEAVAIHGGKDQEEREYAISSFKAGKKDVLVATDVASKGLDFPDIQHVINYDMPAEIENYVHRIGRTGRCGKTGIATTFINKTQSPTTLLDLKHLLQEAKQRIPPVLAELENPMADDDAIMNASGVKGCAYCGGLGHRIRDCPKLEHQKSQQIANQRRDYFGSGGYRGEI

>IbDEAD2

MPRFLTISSPQGTSAGTTDRHGVALLPSNPCTSRSLSLPFIPSLSPPSLSSINSTITKRLYEAPRRSHRRRCSSPLSLSTELRGASKLQLRLSFVPPPSLTEHNLCSLPTEELLEEWIESGKPDCGINPLSVEPLPDKAPVGRLADGSFSRYTGCTRFSQLPLSKKTQDGLALSKYKTMTDIQRASLPHALCGRDILGAAKTGSGKTLAFIIPVLEKLYKARWGPEDGVGCIIMSPTRELADQLFEVLKSVGKHHGFSACVLIGRLLQHMDETPNFDCSQLQVLVLDEADRILDVGFKTQLNAIISQLPKHRQTLLFSATQTKSVKDLARLSLKDPEYVSVHEESTTATPSGLMQTAMVKFVYEAFRKLRPGKMPDRLTGRRRRPPAFRAGRSSQQRFGSRPFFLLHIPIQIHNFFCQVCNASHDAFRGVSPSLPSRGFTSITPVNQLPTPAPLLDWSYCLLTRATSWKVYGSSNTRALRAGYPSPGLSLPFIPSLSPPSLSSINSTITKRLYEAPRRSHRRRCSSPLSLSTELRGASKLQLRLSFVPPPSLTEHNLCSLPTEERRESEVNEIKLLEEWIESGKPDCGINPLSVEPLPDKAPVGRLADGSFSRYTGCTRFSQLPLSKKTQDGLALSKYKTMTDIQRASLPHALCGRDILGAAKTGSGKTLAFIIPVLEKLYKARWGPEDGVGCIIMSPTRELADQLFEVLKSVGKHHGFSACVLIGGRKDIDSEKEHVNGMNILICTPGRLLQHMDETPNFDCSQLQVLVLDEADRILDVGFKTQLNAIISQLPKHRQTLLFSATQTKSVKDLARLSLKDPEYVSVHEESTTATPSGLMQTAMVVPLHKKLDMLWSFIKKHLNKKILVFLSSCKQVKFVYEAFRKLRPGVPLKCLHGRMKLEKRIGIYSQFREEKRSVLFSTDVASRGLDFNKAVDWVVQVDCPEDCATYIHRVGRTARYLSGGKSLLFVMPSEMKMLEKLREKKIPLQVTKAKAEFLQSVSGLMAALLVKYHDLQPLARRAFATYLKSIYKHKDKEIFDVTKLPVEEFSASLGLPMTPKIRFLKQKIKGKTVSEALSVMPENITNENLLELPIKNPNPEKSEEEVEDEDDILLSKDTQNAGEAKTDGAVDALPASRVLKKKKLKINVHRPLGTRVVFDEEGNTLPPLAKLAETKVSADSVHLDKNKVNERYAKMRQELKLMDKEDKALDRQRLKEKRIKEKIKWKRGRDEEEGEEEEEDADDLLGSDANVGRERAKKQKIYFDSDGERDDDSFKTSIKADSVSVAEQEELALKLLSSMHS

>IbDEAD6

MSKRKFGFDGFGINRQATYNFERAEAPQRLYVPPSSRSGGGHDNYEDTDLDNIDYDERDGDEDNNNDVNGGSGGADDEIDPLDAFMEGIHEEMKAAPPPKPKEKLDKYKDDIEDDPMESFLKAKKDIGLQLAAEALNAGYNSDEEVYAAAKAVDAGLVEYDSDDNPIVLDKKKIEPIPALDHSSIDYEPFNKDFYEENPSISGMSEQEVIEYRNSLAIRVSGFDVPRPVKTFEDCGFSVELLKAISKQAYEKPTPIQGQALPIALSGRDIIGIAKTGSGKTAAFVLPLMVHIMDQPELEKEEGPIGVICAPTRELAHQIYVETKKFAKSHGIRVSAVYGGMSKLEQFKELKAGCEIVVATPGRLIDMIKMKAATMTRATYLVLDEADRMFDLGFEPQIRSIVGQIRPDRQTLLFSATMPRKVEKLAREILSDPVRVTVGEIGMANEDITQVVQVIPSDAEKLSWLLEKLPGLIDDGDVLVFASKKATVDEIESQLVQMGFKVVALHGDKDQASRMEILQKFKSGIYHVLIATDVAARGLDIKSIKSVVNFDVAKDMDMHVHRIGRTGRAGDKDGTAYTLITQKEARFAGELVNSLITAGQNVPAELMDLAMKDGRFRSKRDARKGGGKRAKGRGGSGRGVRGVDYGLGIGYSAESSNPSTTTVPSRSAAVNSLRTGMMAQFKSNFVAASSNLQNQALGNSSGMNPNKKMALPGFVSGGTIGGDINASRTSSTSNAATSVQKSREDASHLSSGSSRDRQRERRRPSGWDR

>IbDEAD14

MPVIDRRGLTAPPTVTDSQQPLRPLAQPDSATPQTKSPTHPRPSSQLASQQQHHFSKNFPGRMHFYTPIRLRGCRDFRCVDHCLTFVRAMGGGPRTFPGGLNKWQWKRLHEKKARDKERRLLEHEKQLYQARIRSQIRAKLSPPPQQASAAVTQNPPSDPNQPTYAPMTPQEQIKALADRFMKEGAEDLWNEDDGPIQAPAPEPPQSGWSWRKWKFGKFAYLVKRKAFSHFCTKNWEGTVANVKRMSSAALGKYDMKIKKRIPLQLVEDEYDLSQHVEEIRKEVNQRRLEKSGGEEEEESILSLKRFDECEISPLTVKALTAAGYMQMTRVQEATLSAVLEGRDALVKARTGTGKSAAFLIPAIETVLKPIRSRTVERVPPIYVLILCPTRELASQIAAETNVLLKYHDGIGVQTLVGGTRFKVDQKRLESDPSQIIVATPGRLLDHIENKSGFSTRLMGLKMLILDEADHLLDLGFRKDMEKIVDCLPRQRQSLLFSATIPKEVRRISQLVLKREHAYIDTVGLGLETNEKVKQFYLVAPHEQHFQIVHQLLRGHISEVPDYKIIVFCTTAMMTSLMFSLYREMKMNVREIHSRKPQLYRTRISDEFKEMRGVILITSDVSSRGMNYPDVTLVIQVGIPVDREQYIHRLGRTGREGKDGEGILLIAPWEEYFLDGLKDLPIQKCDLPYLDPNITAKMEESMANIDASVKEAAYHAWLGYYNSIREIGRDKTTLVSLANQFSESIGLEKPPSLFRKTAVKMGLKDIPGIHVRK

>IbDEAD10

MSSTNSKALSSNGCTSTTSIMPAYVAVFLDSREATFGLITKDNYGKYFAAKSGHSRCLLDPHIAEAVTIKEALSWVNKCGHSKVIVYTDCQMVCNLLNGNLPDLSFAGCVINDCHELCRHFEAVLLHHDGSFAAAYNGLLPDCFSPLMAEAMTCKEVFSWIKYRAVLSVVLYTDCSHLQHLLSTPNGDIFSCIAFPIHACRSIMLSFVYCTVSMVPRLVNLGAHTLASIAFSQHHAILQFTFFASRFSASSSPQDHDFHSSTHLLQFQSSHFSRLPVYGSTIGEGDTLRNAYAFAAHFLSPLKLSRSTVSHIISFCWTPVNPLRLRPVTALLQSLRSLQSPDMRSLMASQPSDSKKKQKKPKKRVREDSELLEHLDSLPWNSSLPETDGAFSHLFGDDELGGGFLSLEEVDETTYGLEIPKSSGGNEEPKSKGKPKTKKQKVSESHDDSNGEEGDDKADEEIKQKKKKRSKKKKGSLVNKIEDNTELTAVSNNKNDDEEDSVDETEFYAWNEMRLHPLLMKSIYRLNFKEPTPIQRACIPAAAHQGKDVVGAAETGSGKTLAFGLPILQRLLEEKEKADRLNAEDGEVDEKIAPRGLLRALIVTPTRELALQVTDHIREVAKHTNIRVIAIVGGMSTEKQERLLKRRPEIVVGTPGRLWELMSGGEVHLVEGVDETAFNVLGPSAFAILTILHSLSFFVLDEADRMIETGHFQELQSIVDMLPMASRSTDGHSMETQNCITVSSVQRKKRQTFVFSATISLSADFRKKLKRGSQISKSNDELNSIETLSERAGMRADAAIVDLTNASIMANKLMESFIECREEDKDGYLYYILSVHGKGRTIVFCTSVAALRHLSSMLRILGLNVSTLHAQMQQRARLKGIDRFRSNENGILIATDVAARGLDIPGVRTVVHYQLPHSAEVYVHRSGRTARAFSDGCSIALISSNDASKFASLCKSFAKDKANKTWLERHAESVELELDDDDSEEERVKSFKQKRATSSQLKNLQQELKSLLSLPLQPKTFSNRFLAGAGVSPLLQNQLEELAKLKLGKISNTGDSQRRKLVVIGQDCVEPLQALRSAGPELMQSSGQINANK

>IbDEAD20

MPVKLFPQLSLLNSYIPITASSVMKSIPFSRPSRAIPILSRVFPHKLRYRPFRVALEQPMLEEGQFGSRKFSARSGRGGVSRGRSGSGSGREARAQKSLIEDEADLSDWVSGLRSDSYLKTRVYSDSDDGDDESGNESGGFGGKGSGRGGRDERGGRRGKESGFDDFRRSNRQDVGQDRMQPPSRKGGRFGSEVDRIDETRGMIKCNPFLGKKGDSGVKVNPFSRNGGRSEGEGRRGQKRGAGEIASGYSKERRGSGGGGIARKGGREIRGKSVAVLSEDDDDDEEDDEDVEDNGYKRFRDLIDSEEESEEASGDDEGDDSFEKETVLSSVGNEVRSQPSPPSSLGGSESYLSETRFDKFSLSPLSLKGIKDAGYEKMTLVQEATFPVILKGKDVLAKARTGTGKTVAFLLPSIEIVLKSPPVTRDQKRPPILVLVVCPTRELASQAAAEANKLLKYHSTIGVQVVIGGTRLALEQKRMQANPCQILVATPGRLKDHIENTAGFATRLMGVKVLVLDEADHLLDMGFRRDIERIIDAIPKQRQTLLFSATVPPEVRQICHVALKRDHEFINTVEEGSEETHSQVQQKYLVAPLDKQFSLLYSMLRDHIADDVSYKVLVFCTTAMVTRLVAGLLGIILVTSDVSARGVDYPDVTLVIQIGLPSDRQQYIHRLGRTGRKGKEGQGILVLAPWEEFFLSTIKDLPMTKADAPLVDPETKKKVERALSLVEMKNKEAAYQAWLGYYNSNKTVGRDKCRLVELANEFSRSMGLDNPPAIPKLVIGKMGLRNIPGLRSK

>IbDEAD24

MEEPKQKSSKREDSEKEVEEPRRRSHRDRDEHRDRDKEKNGERHRERDREHYRDKKDRHDREKSTDDKRSDEESSEKKQKNLEEEQAEEQRKLEEEMEKRRRRVQEWQELRRKKEESEREKLGVAAITDEPKSGKTWTLEGESDDEDAALEEKQGMDMDVDGLAKPLDVDGVGSVSSNDVPDLKISQNGGNDASMDDDEIDPLDAFMNSMVLPEVEKLKNAEAPSEDIKSGLKDNTVIYSEEKPKKGVKKAMGRIIPGEDSDSDYGEVENDEDPVEDEDDEEFMKRVKKTKAEKLSIVDHSKIDYPSFRKNFYIEVREISRMTPEEVAAYRKQLELKLHGKDVPKPVKTWHQTGLSTKILDTIKKLNYEKPMPIQAQALPIIMSGRDCIGIAKTGSGKTLAFVLPMLRHIKDQPPLVPGDGPIGLVMAPTRELVQQIHSDIRKFAKVMGLSCVPVYGGSGVAQQISELKRGAEIVVCTPGRMIDILCTSGGKITNLRRVTYLVMDEADRMFDMGFEPQITRIVQNTRPDRQTVLFSATFPRQVETLARKVLNKPVEVQVGGRSVVNKDITQLVEVRPESERFLRLLEILGEWYEKGKILIFVHSQEKCDALFKELLKHGYPCLSLHGAKDQTDRESTIMDFKSNVCNLLIATSIAARGLDVKELELVINFDAPNHYEDYVHRVGRTGRAGRKGCAITFISEEDARYAPDLVKALELSEQVVPDDLKALADSFMAKVNQGLEQAHGTGYGGSGFKFNEEEDEVRRAAKKAQAKEYGFEEDKSDSEDEDEGIRKAGADVSQQVAALAQAAALAAATKANVAQLPGFSPISSATASWWTAFLAAKNLQQNLARLQNEVMPEHYEAELEINDFPQNARWKVTHKETLGPISEWTGAAITTRGYYIPPGKVPGPGERKLYLFIEGPTEQSVKRAKAELKRVLEDITMQATSLPGSAQPGRYSVV

>IbDEAD21

MLEEGQFGSRKFSARSGQRRVSRGGRVRGFGLGNEARAQKSLIEDEADLSDWVSGLRSDSYLKTRVYSDSDDGNDESGNESGGFGGKGRRDERGGRRGGRVVFDDFSRDLIDSEEESEEASGDDEGDDSFEKETVLSSVGNEVRSQLSPPSSLGGSESYLSETRFDKFSLSPLSLKGIKDAGYEKMTLVQEATFPVILKGKDVLAKARTGTGKTRPPILVLVVCPTRELASQAAAEANKLLKYHSTIGVQVVIGGTRLALEQKRMQANPCQILVATPGRLKDHIENTAGFATLLMGVKVLVLDEADHLLDMGFRRDIERIIDAIPKQRQTLLFSATVPPEVRQICHVALKRDHEFINTVEEGSEETHSQVQQKYLVAPLDKQFSLLYSMLRDHIADDVSYKVLVFCTTAMVTRLVAGLLGELNLNVREIHSRKPQSYRTRVSDEFRKSKGLILVTSDVSARGVDYPDVTLVIQIGLPSDRQQYIHRLGRTGRKGKEGQGILVLAPWEEFFLSTIKDLPMTKADVPLVDPETKKKVERALSLVEMKNKEAAYQAWLGYYNSNKTVSRDKCRLVELANEFSRSMGLDNPPAIPKLVIGKMGLRNIPGLRSK

>IbDEAD50

MVTSDFVFEPPSDEEVEYAHDGESEEEEEDDDDIEENETSSRAKNKKSQSPWDFSNYSESVADEHARRSTTSVDYKISKALQQRAAPIAADDDEEDDSDSDYEPHHQEDYVPEDDDDNDVDTSVDRKSFFASAEGVSFHANSFMELHLSRPLLRACEALGYAKPTPIQAACIPLALTGRDICGSAITGSGKTAAFSLPTLERLLYRPKNRPAIRVLILTPTRELAVQVHSMIEKLAQFMTDIRCCLVVGGLSVKEQVSALRSMPDIVVATPGRMIDHLRNSMSIDLDDLAVLILDEADRLLELGFSAEIRELVRLCPKRRQTMLFSATMTEEVDELIKLSLNKPLRLSADPSAKRPSTLTEEVVRIRRMREGNQEAVLLALCSKTFTSKVIVFSGTKQAAHRLKILFGLAGFKAAELHGNLTQAQRLGALELFRKQEVDFLIATNVAARGLDIIGVQTVINFACPRDLTSYVHQVGRTARAGREGYAVTFVTDSDRSLLKAIVKRAGSRLKSRIVAEQSIIKWAKIIEQMEEQVSEILQEEREEMILRKAEMEATKAENMIAHKDEIYSRPKRTWFMTEKEKKLVAKAAKGSMEKGRDSVNKVISAEQAEDLKMKEKRKREREKNLPRKKRRKLEAARELLEDENGSDERNEKTKKDKTGISLVDLAYRRAKSVKAAKKAADAGKIIRKEGKKTKRPTKVNQSRTEEMRDLFKADMSEEKKKRSHSGGVQEKVNSLPQDLDRPDTVTCLVVERIV

54 IbDEAH

>IbDEAH4

MRLIAKRTTDAKTYNLPTVSEVAAVIVGDLDPNMGVQNILVECKSGKLKRINEHNPAYLPLQYPILFPYGEDGYRDDIQFNTVINQAGGSRQRITTREYFAFQIHERRSKLSTLLRARRLFQQFLADGYTMVESGRLLFIRNNQKALRCEVYKGYPNLFITFTCNPKWQKIQRYMGIRGLRVEDRPDIVARVFKMKVDALIKEFNGGKLFGPGVKAGSTAIISSPEVMDSLISAEIPDKENDPEYHQVVEEFMIHGPCGIARKTSPCMVNGKCSKDFPKKFVDSSSFDQDGYPLYRRCDDGKTVKRNNGIQLDNRYVSATNADVVEVVDEINIYYDCRKNISLEGYQLLLEQKVMINVASSGIASILLPGGRTAHSRFTGIYILAALCAYTGLRKMGAQVEEDGDGLKKLEYLSLVSKVCSELETHIGVGDKVLAEFITDMGRNCETVEEFDEKLKESGAEMPDYFVRTLLTIIHAILPPKPKSESEKDSKRTDKGEKFSALKIKDDRDRIKELEREIEVEAKKNRGKDEEEDRRDRDDRRDRRRERDRDDRRDRDRDSRRDWNRDDRRERDRGDRRERGNDAEYRSHDRDRDNRYERRKRRDSDEDRDDDRKNAMYRSEEPELYRVYKGRVSRVMDTGCFVQLDEFRGKEGLVHVSQMATRRVANAKDLVKRDQEVFVKVISVSGQKLRKIHLGEIWRGTRTRIGLSGIRITEDDVVVPSRRPLKRMSSPERWEAKQLMASGVLSVKECPMFDEEGDGLLYQEEGAEEELEIELNEDEPPFLQGQSRYSVDMSPVKIFKNPEGSLSRAAALQSALIKERREVREQQQRTMLDSIPKDLNRPWEDPMPETGERHLAQELRGVGLSAYDMPEWKKDAYGKALTFGQRSKLSIQEQRQSLPIYKLKKELVQAVHDNQVLVVIGETGSGKTTQVTQYLAEAGYTTRGKIGCTQPRRVAAMSVAKRVAEEFGCRLGEEVGYAIRFEDCTGPETVIKYMTDGMLLREILVDESLSQYSVIMLDEAHERTIHTDVLFGLLKQLVKRRPDLRLIVTSATLDAEKFSGYFFNCNIFTIPGRTFPVEILYTKQPESDYLDAALITVLQIHLTEPEGDILLFLTGQEEIDYACQCLYERMKGLGKNVPELIILPVYSALPSEMQSRIFEPAPPGKRKVVVATNIAEASLTIDGIFYVIDPGFAKQNVYNPKQGLDSLVITPISQASAQRAGRAGRTGPGKCYRLYTESAFHNEMSPTSIPEIQRINLGMTVLNMKAMGINDLLSFDFMDPPSPQALISAMEQLFTLGALDEEGLLTKLGRKMAEFPLEPPLSKMLLASVDLGCSDEILTIIAMIQTGNIFYRPREKQAQADQKRAKFFQPEGDHLTLLAVYEAWKAKNFSGPWCFENFVQSRSLRRAQDVRKQLLSIMDKYKLDVVCAGKNFTKIRKAIAAGFFFHAARKDPQEGYRTLVENQPVYIHPSSALFQRQPDWVIYHELVMTTKEYMREVTVIDPKWLVELAPRFFKVADPTKLSKRKRQERIEPLYDRYHEPNSWRLSKRRA

>IbDEAH5

MPPSAARALPPSSTTDLEGKSRLLDFQFEDPAECEAEVFGRGGLSLLVHPDKCKHLQAKEALVIEPPETLMCSQRPYQKQSLYWIILRTEGLKVILSLFVLWHYLVNDDRESSIFLKVDWGRIVLDEAHTIKNWRTMSARAAFTLSAHCRLSHVRTKETKDKEGRPILVLPPTDIQVIVCEQMEASGFYDALYKKSLPGSGKKFSY

>IbDEAH1

MMANEKALVLFICFMILQAAMAAAADLKSCKFDQIYQLGDSTSDTGNYILETSNLTAFPCAHPPYGETFFKKVTGRCSDGLLMIDYIGRRSTVSESVQEKDADFRHGANFAVAGATALPDHVLAAKNIPLTRTHSSLDVQLDWMFTHFNSICLDDHDCAKKLKSALFFVGKIGPDDYSYPLLLQGRPVEEMKAMAPEVVDAIAEGVRRVIKSGGVKIVVPGSFPIGCLPVFLTDFQTHDDADYDDHNCVRKLNDVAKHHNNLVIKAIEKLKKENPNTVIVYGDYYNAFLNLIRNAKKLGFSKIELRKACCGTGGDYNFNVTRLCGTAGVPVCPQPEKFISWDEAHITQHGHQIMSDWVISDILPKLECHQK

>IbDEAH3

MECRNLSIDFNEAITEEDSAITESIGSPVMLMRNIDHTLGLCNGTRFTRIYILAALCAYTGLRKMGAQVEEDGDGLKKLEYLSLVSKVCSELETHIGVGDKVLAEFITDMGRNCETVEEFDEKLKESGAEMPDYFVRTLLTIIHAILPPKPKSESEKDSKRTDKGEKFSALKIKDDRDRIKELEREIEVEAKKNRGKDEEEDRRDRDDRRDRRRERDRDDRRDRDRDSRRDRDRDDRRERDRGDRRERGNDAEYRSHDRDRDNRYERRKRRDSDEDRDDDRKNAMYRSEEPELYRVYKGRVSRVMDTGCFVQLDEFRGKEGLVHVSQMATRRVANAKDLVKRDQEVYVKVISVSGQKLSLSMRDVDQNTGKDLLPLKKSLDDDDLRENPSGGNMEGTRTRIGLSGIRITEDDVVVPSRRPLKRMSSQREGDGLLYQEEGAEEELEIELNEDEPPFLQGQSRYSVDMSPVKIFKNPEGSLSRAAALQSALIKERREVREQQQRTMLDSIPKDLNRPWEDPMPETGERHLAQELRGVGLSAYDMPEWKKDAYGKALTFGQRSKLSIQEQRQSLPIYKLKKELVQAVHDNQVLVVIGETGSGKTTQVTQYLAEAGYTTRGKIGCTQPRRVASMSVAKRVAEEFGCRLGEEVGYAIRFEDCTGPETVIKYMTDGMLLREILVDESLSQYSVIMLDEAHERTIHTDVLFGLLKQLVKRRPDLRLIVTSATLDAEKFSGYFFNCNIFTIPGRTFPVEILYTKQPESDYLDAALITVLQIHLTEPEGDILLFLTGQEEIDYACQCLYERMKGLGKNVPELIILPVYSALPSEMQSRIFEPAPPGKRKVVVATNIAEASLTIDGIFYVIDPGFAKQNVYNPKQGLDSLVITPISQASAKQRAGRAGRTGPGKCYRLYTESAFHNEMSPTSIPEIQRINLGMTVLNMKAMGINDLLSFDFMDPPSPQALISAMEQLFTLGALDEEGLLTKLGRKMAEFPLEPPLSKMLLASVDLGCSDEILTIIAMIQTGNIFYRPREKQAQADQKRAKFFQPEGDHLTLLAVYEAWKAKNFSGPWCFENFVQSRSLRRAQDVRKQLLSIMDKYKLDVVCAGKNFTKIRKAIAAGFFFHAARKDPQEGYRTLVENQPVYIHPSSALFQRQPDWVIYHELVMTTKEYMREVTVIDPKWLVELAPRFFKVADPTKLSKRKRQERIEPLYDRYHEPNSWRLSKRRA

>IbDEAH8

MGTERKRKVSLFDVVDDASTKISKLNGVGAALQPNNAAMSSSVNSWTGRPYSQRYYEILEKRKTLPVWHQKEEFLQVLKANQTLILVGETGSGKTTQIPQFVLDAVDVETPDKRRKYMIGCTQPRRVAAMSVSRRVAEEMDVTIGEEVGYSIRFEDCSGARTVLKYLTDGMLLREAMTDPLLERYKVIILDEAHERTLATDVLFGLLKEVLKNRSDLKLVVMSATLEAEKFQGYFNGAPLMKVPGRLHPVEIFYTQEPERDYLEAAIRTVVQIHMCEPPGDILVFLTGEEEIEDACRKITKEINNLGDQVGPVKAVPLYSTLPPAMQQKIFEAAPPPLVEGGPPGRKVVVSTNIAETSLTIDGIVYVIDPGFAKQKVYNPRTYPEILRSNLANTVLTLKKLGIDDLVHFDFMDPPAPETLMRALEVLNYLGALDDDGNLTKLGEIMSEFPLDPQMGKMLVVSPEFNCSNEILSVSAMLSEVHMYVVLCLLRLSSATVLSGFHAPAGMHHRGSKLVLMQIVAVSQYSLMKQMVSSRLYASADHNEAKGRFSHIDGDHLTLLNVYHAYKQNKEDPQWCYENFVNHRALKSADNVRQQLSRIMTRFNLKLCSTDFNSRDYYVNIRKAMLAGYFMQVAHLERTGHYLTVKDNQVVHLHPSNCLDHKPEWVIYNEYVLTSRNFIRTVTDIRGEWLVDIAPHYYDLSNFPQCEAKHVLDRLYKKRERDREQNKKKK

>IbDEAH11

MVIKWAADLNQYACESLRLNHPGTNEIEPPETLLLNKRGPAIYVNIFSSEATAEFPSALLETAREGGILDNQELAEDMAITQHSKNKRIKGDTLIVCPMALLGQWNDELELHSKPDNISVFVHYGGDRSNDPRVLAEPDVVLTTYDLLTVAYKFDGEISIFLKVDWRGIVLDEAHTIKNWRTMSPRAAFTHIIVCTLQVVQCLTGTPLQNNVQDLYNLINHYEEIWPKYKEVDDLCENFNLLLFCSTFAFWSNPCAIGKYYSSEVLNLSHNKLVGCIPEGRQFNTFEANSYQGNDSLKGKPLSRSSCFFASASTTTEAEALLKWKTSFFMSSSYLDSWSLSNLRNMCNWRGIVCNGSGVTTVSEINLPNAHLYGTLHHLNFTSFPSLTGFNISGNNFNGSIPPAIGDLSNLVFLDLNNNRFDGSIPPQIGKLRELQYLNLTTILLVELFLMRLEIFRSFSGNEFASRFPDFILGCRNLTYLDLSENNFNGSIPDSLFTRLEKLEHLNLSVNAFLRPLSPNIGKLSNLKYLDFNHLNSSIPSELGNCTSLTHLTLAFNSLSGAIPSSLSSLTKLFELDLSYNLLSDRNKISGSIPTHIGNLQHLVELNLLVNNICGSIPQTIGNLTSLKLLLLYNNNLTGTIPPQLGNLHNLVALDLSQNCLYGPIPQTIGNLTNLDSLLLSTNNLIGMLPPQIGDLQKLSDLYLPENNLYGPIPQTIGNLQNLILLDMSKNNLYDPIPQTIKNLTRLKALDLSTNNFTGSLLHKSGNLTSMLISSTICNLHSLQTLVLANNLLTGSIPQCLGNISKDLCVRFTPNQFHGPIPTSFEVGNSLGRLNLRDNRLEGAIPQSLINCQELEVLDLGQNNLSGRFPMWLGALPNLKVLSLRFNKLNGSLTSTYVKGYLFHQLRVFDLSHNEFIGDFPTRLFKHFKAMEKEDPQQQMYLNQSYYYQDSLVVVMKGQDREIVKILVTVPAIDLSCNKFEGHIPDSIGDLLALLNSFENDGLKGEPLSARLWEWHDTTTSSTKGTPPRSDSSFWPDAPFVEEEAYKLAMKINEEDQNKKEKKLASMLTWLLRMTNTQGRGYCC

>IbDEAH9

MAENEDVICFEEGISWLPSHVVDEACHHHHYQQKGQFGYPRSRQHRPMPCPSYSRCSQRTRHPPNCAAAVGGGRGMQAIFLDSGRSCSGTGVFLPRQTGTNGHYASTKHVRPAPPVLLPDRVVQALNLNVHELGMQNKPCRVYQSSEILRHCTLVSGRGSVGDSTEKERRRGEMKFQIEDVEVYFPYDNIYPEQYQYMLELKRALDAKGHCLLEMPTGTGKTIALLSLITSYQLSKPADKIKLLYCTRTVHEMEKTLAELKLLYNYQLKNCGDRAKMLAIGLSSRKNLCVNPDVVSAENRDSVDAACRKLTASWVRSLAAENPNIPTCPFFENYERSSADTSLPSGVYTLQDLKVFGKEKGWCPYFLARHMVQRADVVVYSYQYLLDPKVAGIISKEMQRESVVVFDEAHNIDNVCIEALSVSVRRQTLEGATRNLNRMTQEIDRLKATDAGRLRAEYNRLVDGLAQRGNLPISDAWLANPSLPDDILKEAVPGNIRRAEHFLSVLRRLVQYLKGRLQAENVEKEGPVSFVASINAQVGIDQKMLRFCYDRLHSLMLTLEITDTDEFLHIQTICDLATLVGTYTRGFSIIIEPFDERMPDIPDPVLQLSCHDASIAIKPVFERFQSVVITSGTLSPIDLYPRLLNFNPVCSRSFKMSLTRDCICPMVLTRGSDQLPVSTKYDLRSDPGVEKNYGKLLLEMASVVPDGIVCFFVSYSYMDGIVNSWHNSGLLKDIMQHKLVFIETQDVVETTLALDNYRRACDCGRGAIFFSVARGKVAEGIDFDRHYGRLVIMCGVPFQYTLSRILLARLEYLRETFQIKEGDFLTFDALRQAAQCVGRVIRSKADYGMMIFADKRYSRHDKRSKLPGWILSHLRDAHLNLSTDMAIHIAKEFLRKMAQPYDKSGALGKKTLLSQEDLEKMVTGPDGEMLG

>IbDEAH10

MYIFWSCGGISFLRLRTNIRAELSLHFGRYSPVSREFIRRWRCSDDCACFDGCARRGGAAALLDGDIVEVVGQPAAACPVASHGGGGGPAYLQEKRQIRGGAWLTPIPIGGHVVIMWHHCRGAFARFLFISQSRPGRLRFRRGIHGDDTLRVGDGKVYLVVILVMLLFPPSEGYSNQEILSGHASGGLYASVQAVLKPLFLQVFLPVWHDKLGCPRQFEKVLASHHLCPASSTEANTVIAGVSLPSAVVISFDPPRCCTYLAEGGLRGKSAPGVARSHAMATFGIEWWMQVGFKYFMKGDGSIEGIKATASGKGNLMYTMLLNGALGSVYMCQRLWCMLMISWRWGMIDPCCQFLLKLSTAFRIRDLGEPGFFLGIELVKSSNGVILSQQRYMTDILKRRLGALQYLTITMRIYLRSIKRVNTCMLQRESDWEQLKRVLSVLSLRFNKLNGSLTSTYVKGYLFHQLRVFDLSYNEFTGHIPNSIGGLLALRELNLSHNKFTGHIPTSLGNLSILESLDLSSNQIGRVIPRQLACITMLEVLNLSHNKLMGCITQSTQFNTFEVNSFQGNDGLKGKPLSQGCGNGMTPQLPAPKELHQEDDSSFWRMHQFIARFVEEEAYKLAMKINEEDQNKKEKETSVNVPSSVDFFHHLVLTRKHETLNPYNLKSLAARLRFQVSLSASLLHCNCPLQCLTMNRRGPAIYVNIFSSEATAEYPSALETAREGGILDNQELAEDMAITQHSKNKRIKGDTLIVCPMALLGQWNDELEPHSKPNNISVFVHYGGDRSNDPRVLAEPDVVLTTYDLLTVAYKFDGEISIFLKVDWRGIVLDEAHTIKNWRTMSPRAAFTHIIVCTLQVVQCLTGTPLQNNVQDLYNLINHYEEIWPKYREVDDLCENFNLLALLFNFCLYLSSNQISGAIPVQLASITTLEVLNLSHNKLVGCILEGRQFNTFEANSYQGNDSLKGKPLSRSKLQHLIEVDLSKNSFSGPIPQTIGNLSYLQVLIISSNRLTGTLQPKNGNLTPMHLSPTICNLGALPELKVLSLRFNQLNGSITSTRIKGHLFPQHRVFDLSYNEFTGDLPTWFFKYFKAMTSVDKDRIPQDIYQDSYYCNVINRGCPISASIVHHYGESMVVEMKGQERQLVRTIFILYSAIDLSSNKFEGNIPNSIGDLLALRELNLSCNMLVGHIPISLGNLSMLESLDLSLNQISGAIPGQLASITTLEVLNLSHNKLVGCIPEGRQFNTFEANSYQGNDGLKGKPLSQDCENGIAPQLTAPKELHQEDDSSFLTGCTVKVVAMGYGCGILFGLFIGSLMLLTRKPEFISRFAEEEAYKLAMKVKQRRSKTRRRN

>IbDEAH12

MTIALMFSRLGKGILDNQELAEDMAITQNSKNKRIKGDTLIVCPMALLGQWNDELEPHSKPDNISVFVHYGGDRSNNPRVLAEPDVVLTTYDLLTVAYKFDGEISIFLKVDWRRIVLDEAHTIKNWRTMSPRAAFTHIIVCTLQVVQCLTGTPLQNNVQDLYNLINHYEEIWPKYRSGRFVREFQLIALLFHFCLSCFFASASTTTEAEALLKWKTSFFMSSSYLDSWSLSNLRNMCNWRGIVCNGSGVTTVSEINLPNAHLYGTLHHLNFTSFPSLTGFNISGNNFNGSIPPAIGDLSNLVFLDLNNNRFDGSIPPQIGKLRELQYLNLYNNSFSGVVPYEIGNLQKVWFLDLGLNHYLEASDWSNVKSFPVLRHLSFSGNEFASRFPDFILGCRNLIYLDLSENNFNGLIPESLFTRLEKLEHLNLSVNAFLRPLSPNIGKLSNLKYLDFNHLNSSIPSELGNCTSLTHLTLAFNSLSGAIPSSLSSLTKLSELDLSYNLLSDRNKISGSIPTHIGNLQHLVELNLLVNNICGSIPQTIGNLTSLKLLLLYNNNLTGTIPPQLGNLHNLVALDLSQNCLYGPIPQTIGNLTNLDSLLLSTNNLIGMLPPQIGDLQKLSDLYLPENNLYGPIPQTIGNLQNLILLDMSKNNLYGPIPQTIKNLTRLKVLDLSANNFTGSLLPKSGNLTSMLISSTICNLHSLQTLVLANNLLTGSIPQCLGNISKDLSVLDLHRNQFHGPIPTSFEVGNSLGRLNLRDNRLEGAIPQSLINCKELEILDLGYNNLSGMFPMWLGTLPNLKVLSLRFNKLNGFLTSMRIKGYLFPKLRIFDISYNEFSGDLPARLFKNFKAMKIVDEDRTPMPVYLQSSYYRDTLVVGIKGQDVELVRILITFTTIDLSCNKFEGHIPDNIGDLLALRELNLSHNKFTGHIPTSLGNLSLLESLDLSSNQIGGEIPRQLASITFLEVLNLS

>IbDEAH15

MSKYECRNLRRRLAANALALEKGKKTSQAKSHFSAPIISDDFENHPFKLSTVSDCTFCGAKRFQYEPPGFCCRSGEIKLVSNAMPCVLKNLFTGCDEASKNFMNCVRTYNNTFAFTSLGIHNHDKDLCRRNKGIYTFRVQGQMYHLIDDLIPHGHSPRNLQLYFFDTDAEVDNRVGYTNRLDRSIVSDLVELMSQNPYAQFFRSLKDVKFGDDFVISLNSTTSLDQRVYNLPTASQVAAIWLENDSDKVPNNRSIKVYACSGRSHNVQYYYGCYDPLQYPLLFPLGETGWHEGIDRNVAHTYNKVRKRQSCIIDPTIVSSVEELIATEESIISEGKKKRTQQDQSRIETYQGLVDSVGVGATTGYDVGRRIILPVSFIGGPRDMRRRYMDAMSLVQRFGKPDLFLTMTCNPNWPEIKELLCYNDEAHNRPDLLARVFRARVEVMKDDILKKPIFGEVAAYTYVIEFQKRGLPHAHFLLILRGKHKITSPDQSDKINARWVSPPEAGWRIFSFNLAEMKPSIVHLQVHLPNYQYIKFNKHEKLVSIVDEKNGEQNYRSRTMLTQRWHQRKQREVIGRVVSVMPSKDCGGVFFLDGPSGTGKTFLYRCLLAKVRSQGFIALATASSGIAASILPGGRTAHSRFKIPIDGDNRYVCNISKQSADAELLRHSKLILWDEASMANRKCVESLEVTLRDLMNSEDTFGGKVVVFGGITTMVRKLLISDVSPGEKDWSCKVCVVKKLDAQDSKTTPDAIVSIPRGNLVLPDERYNCLWTLTRKCTIAVVPDEDKLEVMEDPEIDEISFSHFHKYINTMKKISVMAIVIHKLPRKHVDSKNGKIDAADFVLVDKQAKPVIFTLWGKYSKVEGVELEKQLTIGNFPVILAKNVEVTNYSGLSLSTRFGSVIELHIATPTAEELGQWALSNSVTLHSLILTNAFNDAYLKLADLSIEQFVTIADVKSSIIEGKRYCIYASIQIPTKVKKFYYIACDGCWKGTIHNLGEEFECSYCGNSMAIAKPRCMMTIELTENNDFLESVLFGNIVEELLSVSASKLVDMDKKEQKKIAHAKRVKRKLIYDDTDDNTSDEHSKAFGDFDNEAMAYANEIDIFDNKLSLNATYIITDAIVSIPRGNLVLPDERYNCLWTLTRKCTVVVVTDEDRLEVMEDPEIDEISFSHFHKYINTMKKSVCVMAIVIHKLPRKHVDSKNGKIDAADFVLVDKQAKPVIFTLWGKYAQVEGVELEQQLTIGNFPVILAKNVEVTNYSGLSLSTRFGSVIELHIATPTAEELGQWALSNSVTLHSLILTPRFNDAYLKLADLSSEQFVTIADVKSSIIEGKRYCIYANIQIPTKVKNFYYIACDGCWKGTTHNLCEEFECSYCGNSMAVAKPRCMMTIELTENNDFLESILFGNIVEELLSVAAID

>IbDEAH16

MVIKWAADLNQYACESLRLNHPGTNEIEPPETLLLNKRGPAIYVNIFSSEATAEFPSALLETAREGGILDNQELAEDMAITQHSKNKRIKGDTLIVCPMALLGQWNDELELHSKPDNISVFVHYGGDRSNDPRVLAEPDVVLTTYDLLTVAYKFDGEISIFLKVDWRGIVLDEAHTIKNWRTMSPRAAFTHIIVCTLQVVQCLTGTPLQNNVQDLYNLINHYEEIWPKYKEVDDLCENFNLLLFCSTFAFWSNPCAIGKYYSSEVLNLSHNKLVGCIPEGRQFNTFEANSYQGNDSLKGKPLSRSSCFFASASTTTEAEALLKWKTSFFMSSSYLDSWSLSNLRNMCNWRGIVCNGSGVTTVSEINLPNAHLYGTLHHLNFTSFPSLTGFNISGNNFNGSIPPAIGDLSNLVFLDLNNNRFDGSIPPQIGKLRELQYLNLTTILLVELFLMRLEIFRSFSGNEFASRFPDFILGCRNLTYLDLSENNFNGSIPDSLFTRLEKLEHLNLSVNAFLRPLSPNIGKLSNLKYLDFNHLNSSIPSELGNCTSLTHLTLAFNSLSGAIPSSLSSLTKLFELDLSYNLLSDRNKISGSIPTHIGNLQHLVELNLLVNNICGSIPQTIGNLTSLKLLLLYNNNLTGTIPPQLGNLHNLVALDLSQNCLYGPIPQTIGNLTNLDSLLLSTNNLIGMLPPQIGDLQKLSDLYLPENNLYGPIPQTIGNLQNLILLDMSKNNLYDPIPQTIKNLTRLKALDLSTNNFTGSLLHKSGNLTSMLISSTICNLHSLQTLVLANNLLTGSIPQCLGNISKDLCVRFTPNQFHGPIPTSFEVGNSLGRLNLRDNRLEGAIPQSLINCQELEVLDLGQNNLSGRFPMWLGALPNLKVLSLRFNKLNGSLTSTYVKGYLFHQLRVFDLSHNEFIGDFPTRLFKHFKAMEKEDPQQQMYLNQSYYYQDSLVVVMKGQDREIVKILVTVPAIDLSCNKFEGHIPDSIGDLLALLNSFENDGLKGEPLSARLWEWHDTTTSSTKGTPPRSDSSFWPDAPFVEEEAYKLAMKINEEDQNKKEKKLASMLTWLLRMTNTQGRGYCC

>IbDEAH17

MKKSPLPLKNICGPEKKSIGKEALVKLLRWHFGYANFRGKQLEAIEAVLSGRDCFCLMPTGGGKSMCYQIPALAKPGIVLVVSPLIGDFLDPILISYHEGTQATGFFLCWLANDRDMTAFIGLRFVSALMENQVSALKEKGISAEFLSSTQTSQVKTKIYEDIESVKPKLRLLYVTPELIDTVGFMSRLVKIHSRGLLNLIAVDEAHCISSWGHDFRPSYRKLSSLRSRLPNIPVLALTATAAPKVQKDIIGSLSLQSPLVLKSSFNRPNIYYEVRYKDLLDDPYADVCNLLKSCGNVCAIVYCLERTTCDDLASHLTTYGISCAAYHAGLNNKLRTSVLDDWISSKTQVVVATVAFGQFEACITADFAAGWYASNTSEYGIDRKDVRVVCHFNTPKSMEAFYQESGRAGRDQLPSRSVLYYGVDDRRKMEFILSNSERKKQSSSLQDGSSKKALDNFRQMVEYCEESGCRRKKILESFGEMVSASLCEKSCDACKQPNIVAKNLEELKTAATFRQRNGSSRIFITSLSNFKDGDYSEFWNHNDEASGSEEDISDSDDCMNTEALDAAKNVASTGRSTKLRIQDKIEMLQRAEEKYYQNKNPEKQVNKLDKNAIPETLRETGRQRLLNTLKQNKRISDLRMSFEDSASILENECYKKYSKSGKSFYLSQMASTVRWLSTADRTELTNKLGCAPDPPKDLQQPSEANCSSESLTSSIALSAGLNSEKTNSVVLETEAAENVSPATTLPPIPSFSEYINSRKVKSHHESKSKKRSPDMSQKNIQKKMRS

>IbDEAH20

MEMEIAEGAIHDNGSVPGERLCLATAQSPTSDQIDVQRSCGSAGPDTSRLYRVSSFLAGSRLRLTVFTLRPQESQRCTGLRNMGALVEEDGLKKLEYLSLVSKVCSELETHIGVGDKVLAEFITEMGRKCETVEEFDEKLKESGAEMPDYFVRTLLTIIHAILPPKPKSESEKDSKRTGEKFSALKIKDDKERIKELEREIEDRDRDNRYDRRKRRDYDEDRDDDRKNGMHRSEEPELYSVYKGRVSRVMDTGCFVQLDECRGKEGLVHVSQMATRKVANAKDLVKRDQEVYVKVISVSGQKLSLSMRDVDQNTGKDLLPLKKSLDDDGLRENPSRGNMEGTGTRVGLSGIRITEDDVVVPSRRPLKRMSSPERWEAKQLMASGVLSAKECPMFDEEGDGMMYQEEGAEQELEIELNEDEPPFLQGQSRYSVDMSPVKIFKNPEGSLSRAAALQSALIKERREVREQQQRTMLDSIPKDLNRPWEDPMPETGERHLAQELRGVGLSAYDMPEWKKDAYGKALTFGQRSKLSIQEQRQSLPIYKLKKELVQAVHDNQVLVVIGETGSGKTTQVTQYLAEAGYTTRGEEVGYAIRFEDCTGPETVIKYMTDGMLLREILVDESLSQYSVIMLDEAHERTIYTDVLFGLLKQLVKRRPDLRLIVTSATLDAEKFSGYFFNCNIFTIPGRTFPVEILYTKQPESDYLDAALITVLQIHLTEPEGDILLFLTGQEEIDCACQSLYERMKGLGKNVPELIILPVYSALPSEMQSRIFEPAPPGKRKVVVATNIAEASLTIDGIFYVIDPGFAKQNVYNPKQGLDSLVITPISQASAKQRAGRAGRTGPGKCYRLYTESAFHNEMSPTSIPEIQRINLGMTVLNMKAMGINDLLSFDFMDPPSPQALISAMEQLFTLGALDEEGLLTKLGRKMAEFPMEPPLRAIFFTDLGEKQAQADQKRAKFFQPEGDHLTLLAVYEAWKAKNFSGPWCFENFVQSRSLRRAQDVRKQLLSIMDKYKLDVVSAGKNFTKIRKAISAGFFFHGARKDPQEGYRTLVENQAVYIHPSSAVFQRQPDWVIYHELVMTTKEYMREVTVIDPKWLVELAPRFFKVADPTKLSKRKRQERIEPLYDRYHEPNSWRLSKRRLICTYFNNALTISQDLFIISCVHFTMYRAPAWEQFKRMYYSYMQMRAINVIEIFHMMQSF

>IbDEAH19

MCHAFPELWNIEKRFGSVIFGAVQSKIQLRRGTKGKLVLRIYTVPLPITSCFCSLTPCFADTYRCIVASFICMIDNGIGKQYGSYGFNYLVLFCTIFQIILYVELYASIDALTMQVFLSYTLRWEYLGVDDKVICCLMNSCMVEERSAGMVDLCAKGGGEEPLLGHTSLKPCLRFIKVPGEKIQSSVPRGLRLESRVEGDDFCYSVSCQDEAWFDTTSIFESDSDGDDDDDFSSVHGGTVKSPTETKERKRERDNRYKQAAFCYEELILSHPVPLYHLAYADNSDMEVTLTQVRPHAVVTPKPDVWQHLSTTNILYTTIAINILGTEGLKVALSLFVLWHYLVNERTRCGLDNLYGLLSAAYKADGGSSIFHKVDWHRIILDEAHTIKSWRTVSARAAFTLSVHCRWCLTGTPLQNNVQDLYSFLCFLHVEPWCNWAWWNKLIQRPYENGDKRALKLIKAILRPLMLRRTKETKDKEGSFPPPFFHLYIILEKAQLEFFIWRIGKNMGEKFYANLEVFFSKSALIYIDQSQSLNIHMDQPNFGKLLPCIFMLGSENWNVLSRSRAAADGIKFTVDTSMLKVVPHCRYQFLLLPSSI

>IbDEAH18

MKNQKPPPPPPSSATKNPNTTTTKSVVHIGGIPVEFPYQPYGTQLAFMNRVIATLDRAQREGRSHALLESPTGTGKSLSLLCSALAWQQNFKSKSRHSNPTCSKPDPEALADPIGHGGGFIPETQPSGNPEPAPSVTTAGKNQKKKLAPTIFYATRTHSQISQVIREYRKTSYRVPMGVLASRRHYCTNMNLRGMDNIDEKCKLLLKDKEAGCCEFKNAHKVKAHPSLQKGGCHEAHDIEDLVKVGQVVRGCSYFAALSMADDADLVFCPYSYIINPVVRKAMDVDINGAIIILDEAHNIEDISRDAGSMDVEEDVLRQLLTELKQLEVSDAMTYEPLIGMVQEIIDWIGRRTDSLEKHDFQHYFSCWTGDKALKELEEAHVSRQCFPILKECATKAIRVASDAEPDVAHLSGMAATVLEGLFSSLNYFFSENGIRVCDYQLALRRHRNRVAGSAVNDWTHTFSLWCLNPAVVFREIADRSLSVILTSGTLSPMNSFSSELGVPFSTSLEAPHVIDTESQLWAAVISRGPSNYPLNASYKTADSYAFQDSLGTSLEEICKVVPGGCLVFFPSYKLMEKLSSRWQATGQWDILNAKKPLFVEPRGSQEEFESVLKGYYESIHQGRKPVLGRRKGRKSEKNLSGSSEDNKKGAAFLAVCRGKVSEGIDFSDEMARVVIVVGIPFPNINDIQVSLKKKFNDTYKLSKSLLSGSEWYCQQAFRAVNQATGRCIRHKFDYGAIIFLDERFCQVRNRAYISKWLRNSIKQYDCFDDSLDGLKSFFRDIKDRIGKPDDASQNSVINLEDMPSANKSRISRKTNQKVNNSTLPGEKAEANDANRIQKSAGLFKFSKMFTRCDLPNNQVSADIQGFISPNEKNSKDSRSYIDLECASPNGSRCSGEAFGAACTGDPQSTFVMETPGVSGTIHSTSPESFSKEYSNSTIIQESVQDLNNLTCDNMSISDVKQDPESKCLERKGENTWMYHWLAISRERNLILQLPAHLLQCIGIPENNLIVKCSMTSSTKVHLKSLWKRKSDPDMRTSSIPILVSDMESINRQIYNITRETIPAQGIWCKDDGCVFKTVFCPFCANPNNCLGLQVMAADASNVQFLNKILFYSDSLLIKAAETSTMDLSPCLGSTTCGKAGPSSIENFSYTPYQSPTYVKADPSTIENFSYTPDKNSTGWRSTKSKMRLPKRGHL

>IbDEAH24

METPYSTKDCQVSTSSDPPGPTMPANFVINLRSSNQTMTSLELRQLVKKLPKRPQSSSLCDGGGAKLCYERWPETLEAMEHLWRARLAGELLFTPGLIGNVELEKRVMTVFYQRVRELMYGELVKNSGKKLTELTEEANGVTAKLQKPNSLGVASDLLKRRDALMVEVELIRERIKEFKQGIRCVWFHLKDMDDSEESVSVLKLEGEFNWDRLHYLMMRECKRLQDGLPIFSFRQQILREIHCHQVTVLTGETGSGKSTQLVQFLADSGIGGDGAIVCTQPRKLAAISLARRVKVECVGCYEDNSIICHPSYSSSSRFDAKIVFMTDHCLLQHYMSDNDLSKISCIIVDEAHERSLNTDLLLALIKGLLHRRADMRLVVMSATADADQLSKYFIGSGKFHVIGRSFPVDIKYVPCESEGCSGVVAPYASSVVRMALDIHNTHKDGAILAFLTSQMEVEWACENFRSPSAIALPLHGKLSNEDQNQIFLDFPRMRKVIFATNVAETSLTIPGVKYVVDCGLVKEKRFEPGSGMNVLRVCKTSQSSANQRAGRAGRTEPGKCYRLFSETDFESMPCHQEPEIHKVHLGIALLRILAFGIKDVQNFDFIDAPSPKAIETTIKSLIQLGAIVQIDDGYELTAEGLRIVKLGIEPRLGKMLLDCFEHQLGKEGLALAAVMANYSSIFCRVGTEEEKLKSDCQKVQFCHPFGDLFTLLSVYKEWDALPREKRNRWCWDNSVNAKTMRRCQETVQELEACLQHELRIVAPSIWHWNPEVQSEHDDTLKRIILASLAENVAMYSGYDQLGYEVALTGKYFQLHPSCSLLNFCQRPTWIVFGEVLVAAKEYLVCATAFDFGFLAALEPAPSFDFSKMDTRRLQKKVLTGFGSLLLKRFCGKSNCSLNHLVSQIRLSCMDERIGIEVRVDHNEVILNASSGDMEKVLAAVNEALEHKRNLLKNECLEKCLHTGGPGSSASIALFGAGAEIKHLELEKRCLTVDIFHSIGTAVDDKELVMYLERNCGDICAIFKFSGMSQENEELEKWGRVTFLTPDAANLATALNLVEFNGGLLKIVPSKVMYGGDKKMFSFPLLRAKVYWPRRGSRGKAVLRCDPNDVFLIVDDLSDLEIGGRFVRCEPSTSFPDRVVITGIDKDISETEISEAINELTDRKILSLFLVRGNPVFQPEPNDSYMRAIITFDGNLHLEAAKALEELDGKVLLGFLPWQKIKCQQIFQSSVSCPAAAYHVIETELHHLLERLKNHKGVECRLEQNENGPYRVKISANATKTVAEVRKPLEELMKGKAIDHEGVTPTVLQLLFSWEGINVMRVDMAEQKFVSSLLALHENKQLVVRLRGEALPPDLMKRVVDMFGPDLKGLKEMFPEGDFYLNTRRHCIHVKSGTKDLCQRVEDAIYELARASGSHIQRNDDEASCPICLCELEEERYKLESCLHVFCRSCLLEQCESAIRSSEGFPLRCLHNGCNAPILITDIKSLLQVEKLDELFRASVSAFVASNASYRFCPSPDCPSVYHIAHPDSSGSAFMCGVCYSETCTKCHQEYHPYLSCENYKEFKNDPDLSLKAWAEGKENVKVCPVCKSTIEKVDGCNHIECRCGKHVCWVCLEFYETSEECYNHLRSVHLSIV

>IbDEAH27

MQKPGEGEVTGPIDLDKTTTTLEPDGASGGGLYIPGKDRVVFRPPERKSLLGLDMLASAKRGGSTIENSFKVPRERVASVVSAIDEETSTETGQLEDVPSRGSRSHTSRRYRDSVASEASISGITVTEEGRENETLSRPRSDEHYQVPTPSTGRSRTKSSSHDFDHGRERKSRDDYRSKSREVKRDRIDGEEHRHRESSRNHGKEYNDDSRRKRSRYESPRNTRGRSDWDDGRWEWEDTPRRDSSSYSSSRRHEPSPSPMFIGASPDVRLVSPWLGGHTPRSGAASPWDSVTPSPTPIRASGSSVRSSGSRYGGKSSRLSSSANTFRLSEDDGDDRIRGSEDDNQGMEITESMRLEMEYNSDRAWYDREEGSTVFDNDRSSLFLGDEASFQKKEAELAKRLTRKDGTKMSLAQSKKLSQLTADNAQWEDRQLLRSGAVKGTEVQTEFDDEDERKVILLVHDTKPPFLDGRIVFTKQAEPVMPIKDPTSDMAIISRKGSVLVREIHEKQSMHKSRQRFWELAGSKLGDILGVEKTAEQVDADTAAVGEQGEVDFKEEARFSQHLKKGEAVSDFAKSKTLSQQRQYLPIFSVREELLQIIRPRCSSFLCMQLCMEPVKAARYIEVEVVHENQIVVVVGETGSGKTTQLTQYLHEDGFTTNGIVGCTQPRRVAAMSVAKRVSEEMETDLGEKVGYAIRFEDVTGPNTVIKYMTDGVLLRETLKDSDLEKYRVIVMDEAHERSLNTDVLFGILKKVVARRRDFKLIVTSATLNAQKFSNFFGSVPIFHIPGRTFPVQIMYSKTPCEDYVEAAVKQAMTIHITSAPGDILIFMTGQDEIEATCYALQERMEQRRKCIVATNIAETSLTVDGIFYVIDTGYGKMKVYNPRMGMDALQVFPVSRAAADQRAGRAGRTGPGKHTQLHVSAVGIGRALNNVGDLTDLGWKMVEFPLDPPLAKMLLMGEQLECLNEVLTIVSMLSVPSVFFRPKDRAEESDAAREKFFVPESDHLTLLNVYQQWKSNQYRGDWCNDHFLHVKGLRKAREVRSQLLDILKTLKIPLTSCGPDWDIVRKAICSAYFHNAARLKGVGEYVNCRNGMPCHLHPTSALYGLGYTPDHVVYHELILTTKEYMQCVTAVEPQWLAELGPMFFSIKESDTSMLEHKKEAKGRKDCHGGRNGEFEEGASRD

>IbDEAH26

MKLAVENKLGSYSIGDGDFLVLVPFAKKDRQQAEQSATSPAMTRFETSKQSESTWRDVVEDLSVLRSTINNKTQNDIELESVNSENRQAQNVNVSSSGTSQRKRKLKSVSNKTEQPTDELIFDILQSPSSSIDEQASKFVMVLDSVNCLFDPSSGNCICNEARRQNTEMNPRSTESNLCLCPSWLKSKMKLFSFINIYSAVIQLQHGKVTLCSLKQALDQLGKFGFRASITDVEHLSDLCPQVLCIVDNSKGAITSTNAIMIVKTSTEQSDQHENCQFNEEKARVLQNKSFEGHKIINVNMKLNNVLKSVVVSCLHFFVERLGNNISVLINNKTIIMQFEDGNKFSKFSLEDFLQYVKQCNDVASGSKVNTESSHSFEALCHDTNPLLPIEMLEHLRRGIGSKGQVVHVEEINALGMLIVATMTSSGKSLCYNLPVLEALSQNLSACALYLFPTKALAQDQLRALLTMTNEFDHNLNIGVYDGDTSQTDRMWLRENARLLITNPDMLHVSILPFHGQFRRILSNLRFIIIDEAHAYKGAFGCHTALILRRLQRLCSHVYGSNPSFVFSTATSANPVDHAKELANLPALELIQNDGSPSGPKLFVLWNPPLCLRTVQPLQIFQREVGRADANKSTDRSEVARRSSPIMEVSYIFAEMVQHGLRCIAFCKTRKLCELVLSYTREILQEAAPHLVDAICAYRAGYVAEDRRRIERDFFSGNICGIAATNALELGIDVGHIDVTLHLGFPGSISSLWQQAGRSGRRGKPSIAIYVAFEGPLDQYFMKFPNKLFRSPIECCHVDANNQQVLEQHLTCAAFEHPLSLQHDKKYFGPGFEAAVMTLKNKGYLNTDVSRDSSARIWSYIGHEKMPSNAVSVRAIETERYKVIDKQKNELLEEIEESRAFFQVYEGAVYMNQGKTYLVKDLDLSNEGSLQALCSFEKIGNRIVAYPARNSNIQFARTTAQAQFCRVTTTWFGFRRIWKKSNQVFDTIELSLPNYSYESQAVWVPVSETIKKTVEALNYSFRGGLHAACHAILNVVPLYIICNTSDIASECVNPYDARYAPERILLYDPRPGGTGIAAQVQPLFTELLTAALELLTSCHCSSDAGCPNCIQNLACQEYNEVLHKDAAIMIIKGVIQAEKLRD

>IbDEAH32

METVEKNPVVVVIGETGSGKSTQLSQMLHKRGYTKTGMVAVTQPRRVAAVTVSRRVAEELGVQLGEEVGYAIRFEDRTSEKTNIKYLTDGVLLRESLSNPELNQYSVIILDEAHERSLNTDILLGLMKRLIKLRGSNLKVLITSATLDGEKVSRFFFDCPVLNVPGKLFPVEIRHSSEKPKNYVEACLKTAIDIHVQEPEGDVLIFMTGQDDIEKLVLKLEEKIQGLEEGSCMDAIVLPLHGSLPPEMQASLNFHLQFRCILDLLFHLSVVAIGFAILLDYCFMTSPPSEDVRVFNRPPPSCRRFIVATNIAETSLTVDGVVYVIDSGYVKQRQYNPSSGMYSLDVVQISNFKCVEFMPVGIWCCMIYGVGPRAQESTLCRVWSREERVQANQRAGRAGRTRPGKCYRLYPSMIYHDDFLDATVPEIQRSSLAGAVLYLKSLDLPDMDILNFDFLDPPSVESLEDALRQLYLIDAIDENGSITSLGRTMAELPLEPSLSRTLIGANDLSCLSQALTVVAMLSAETTLLPGPRKNTEKKKRKHTPSNLPDGSGWGDHIQLLQIYELWDQTDYSVDWCKENNLQVRGMMFVKNVRGQLTQIMQKMAKESLDVQTSRRHKKGQQDYKNLRKALCTGYANQLAERMIRHNGYRTLGFKSQLVQVHPSSVLKADEDGMLPNYVVYHELISTTRPFMRNVCAVEMQWVVPILGRLEKLNIKKLSGGSDQPEERIQEENSNLEKKVAAHSEPPEERDSRIQAARARFLARKAQKYCLEPVAGKPMRAIAGPENESWSRAPVTVRGWVVLGRRRRPFAEVSRRRSRPPSPAWSVFPATICDGESTGDLAVKKGFYLGGERCLVFQRCRIVLAAAVASEGFQQCRIGHGWRCGIGRGRQFMMKAMEGMVQAWCQGVRVGGSPGVLQMGSTGARGGVDLAYVAAIMEGRRLKNGSGETGLCANKKTENTGNIIRVCGVRGWCVAVLSSDDKVVGYERQVRCGCGVWPGL

>IbDEAH28

MPSMANAAQNNQHDQLANGKDDLLRRQKIEKQRKSLPIASVEKQLVKEVHNNNTLIIVGETGSGKTTQLPQYLYHGGFCRDGGIIGVTQPRRVAAITVAKRVAEECGVELGQRVGYAIRFEDVTSNSTKIKYMTDGLLLREALLDPYLSKYSVIIVDEAHERTVHTDVLLGLLKNVQKARLQNAHEGVSVDSTKPKNELLLEDKQDAHFDGIFKKCRAKNYTPLKLIIMSASLDARVFSEYFCGARAVHVQGRQFPVDIFYTHQPETDYIDAALITIFQIHLEEGPGDVLVFLTGQEEIESVERLVHDRLQHLPEGNRKLLTFPIFSSLPSEKQMKAFMPAPAGFRKVILATNIAETSVTIPGIKYVIDPGLVKARTYDADMGIDSLIVVKTSKAQALQRSGRAGREGPGKCYRLYPESEFEKLDDSTIPEIKRCNLSNVVLQLKALGIDNIVDFDFIEKPKRMALVKSLETLFLLGAITENNELSDPIGHQMARLPLEPMHSKALILASQFGCLEEMLISVAMLSVESIFYAPREKLEESRNALKSFASPEGDHLTLLTVYRASNELFEKSKIANGELKAEKNLRKWCKDNFINSRSLRHARDIHSQIHRNVEQMGLRISSYGEDTLLFRRCLAASFFLNAALKQPDSTYRVLSSGSTVQIHPSSVLFRAKPECIVFNELVQTNYTYVRNISRIDYLWLAELAPHFYALQD

>IbDEAH29

MQKPGEGEITGPIDLDKTTTTLEPDGASGGGLYIPGKDRVVFRPPERKSLLGLDVLASAKRGGSTIENSFKVPRERVASVVSAIDEETSTETGQLEDVPSRGSRSHTSRRYRDSVASEASISGITVTEEGRENETVLRPRSDEDYLVSTPSTGRSRTKSSSHDFDHGRERKSRDDYRSKSREVKRDRIDGEEHRHRESSRNHGKEYNDDSRRRRSRYESPRNTRGRSDWDDGRWEWEDTPRRDSSSYSSSRRHEPSPSPMFIGASPDVRLVSPWLGGHTPRSGAASPWDSVAPSPTPIRASGSSVRSSGSRYGGKSSRLSSSANTSRMSEDDGDDRIRGSEDDNQGMEITENMRLEMEYNSDRAWYDREEGSTVFDTDRSALFLGDEASFQKKEAELAKKLTRKDGTKMSLAQSKKLSQLTADNAQWEDRQLLRSGAVKGTEVQTEFDDEDERKVILLVHDTKPPFLDGRIVFTKQAEPVMPIKDPTSDMAIISRKGSVLVREIHEKQSMHKSRQRFWELAGSKLGDILGVEKTAEQVDADTAAVGEQGEVDFKEEARFSQHLKKGEAVSDFAKSKTISQQRQYLPIFSVREELLQVVHENQIVVVVGETGSGKTTQLTQYLHEDGFTTNGIVGCTQPRRVAAMSVAKRVSEEMETDLGEKVGYAIRFEDVTGPNTVIKYMTDGVLLRETLKDSDLEKYRVIVMDEAHERSLNTDVLFGILKKVVARRRDFKLIVTSATLNAQKFSHFFGSVPIFNIPGRTFPVQIMYSKTPCEDYVEAAVKQAMTIHITSAPGDILIFMTGQDEIEATCYALQERMEQLVSSAKQAVPKLLILPIYSQLPADLQAKIFQKAEEGERKCIVATNIAETSLTVDGIFYVIDTGYGKMKVYNPRMGMDALQVFPVSRAAADQRAGRAGRTGPGTCYRLYTENAYQNEMLPSPVPEIQRTNLGNVVLLLKSLKISNLLDFDFMDPPPQENILNSMYQLWVLGALNNVGDLTDLGWKMVEFPLDPPLAKMLLMGEQLECLNEVLTIVSMLSVPSVFFRPKDRAEESDAAREKFFVPESDHLTLLNVYQQWKSNQYRGDWCNDHFLHVKGLRKAREVRSQLLDILKTLKIPLTSCGPDWDIVRKAICSAYFHNAARLKGVGEYVNCRNGMPCHLHPTSALYGLGYTPDYVVYHELILTTKEYMQCVTAVEPQWLAELGPMFFSIKESDTSMLEHKKKQKEEKTAMEEEMENLRKVQAEIDRRNKEKEREKRERQQQQVSMPGLKKPKPSTYLRPKKLGL

>IbDEAH31

METVEKNPVVVVIGETGSGKSTQLSQMLHKRGYTKTGMVAVTQPRRVAAVTVSRRVAEELGVQLGEEVGYAIRFEDRTSEKTNIKYLTDGVLLRESLSNPELNQYSVIILDEAHERSLNTDILLGLMKRLIKLLGSNLKVLITSATLDGEKVSRFFFDCPVLNVPGKLFPVEIRHSSEKPKNYVEACLKTAIDIHVQEPEGDVLIFMTGQDDIEKLVLKLEEKIQGLEEGSCMDAIVLPLHGSLPPEMQASLSSIYCFPLFSPQRIELQHILSLAF

>IbDEAH30

MFSSSLTLSSFITSFLLLFFSTCVNGDGQESGGLQGKHGNSFSAVYAFGGSFTDTGNAYIMGRLKGNYGVTCQRSGGRLCNGRLAVDFVSAAFSLPSPQPYLNASKNFNSGAATTGVNFAIAGSTSLQHKYFTNEELTHVIWKGIPMTFETQIHWFRNFLKEKNCLGKHAIRCKQEFKNALFWVGPMGISDYYSIQGSSIAHKWLTQLSIEEFTKLIKALINAGAKYIVAESLPPLGCLPLSISSFEPRKLDQMGCVAPVNSAIKLHNEILQSKVRELRRECRDCAILYADFWKAYETIVTSPAKYQLEDTKKACCGTGKGDLNFDPNAVCGAPTTSACTDPAKYVSWDGIHITEAMQRQLADLYLNQSFCEPSFDTMLKHKSSI

>IbDEAH36

MGRLMRARRTVASKKATLKFMVWITILSPRQLKLHRLIAFIFVTIGTYINNWISRIFSRGDLQEEVYMEQPQLLPGRGAITRRASEKISGKLIISLSKTGYLFWVSVLSRFLENPCDLIGITVVDSRRYLSYCVLIGGIDILENSHILHQSVFLERTKHIEVDCHFIGENYVGDVLLRLSTLQNSFFTKEPPGTVRAEGKLLPQVSTPTPHPPGVFVHNYWLMHKLQQLTRTRNVLVLRMWMELLLQMEVPRKPPSQKRATVKPIKPEAVIVISPDTKEEILADAMGLGRTVMTIALILAKGIPDNQELAEDMAITQHSRNRRIKGVTLIVCPMALLGQWKDELEAHSKPDSISVFFSLWWGHKMGGSSIFHKVDWHRIVLDEAHTIKSWRTMSAGAAFTLSLHCRWSLTGSPLQIFTAAFCVSCMLSHGAIEHDLQLRESLLRIKQKNMAGEGRNRRVLGDICNVVTVRGAEGKQQLPQESRRAQLLANAQAAPADKSRRNVLVLRMWMELLLQMEVPRKPPQKRANVKPIKPEAVIVISPDTKEEAASGLKIKLPKEKIEDIDAGDVYNELAVLEYAEDIYKFYKEAESTRCIHIAIMAYVHWVLWMIPTRFEVLFSLLNQVVDEYQKNFGDTWRSLLNLIVLNNGLIYLKLSLADKLKFLSFLNGCFPWNFQALPIFIDALLPAWGAILIPGTLILAFGEIIPQAVCSWYGLCIGMIGGILFTFYQGSNLDSNAKMWRLIADFMKEKNSLRRKTAAKDSSRKIYTATLTARSKAACGLNIKLPKEKIEDIDAGDVYNELAVVEYPEDIYKFYKEAEDMMLCGGSDSVIIPTGTKVVAFCFQGSNLDSNAKMNVDGPSLSSTSIGIFVHSMFGELIQIIYGATRAALTQHFALQDNAADMSAKQIIWPLVVFHSQLLTVKGARFFCHISSRLAKFSLSPKQVSTMEHILPLWMTSWSPKSTLHKNVSLAKYLLLEKNGIVSIVVHKDSTAADILQSFVHSLVMSKLAGEKRDVHMESRESELWIEKHYKIFISKLQSSGWKTERLLSPSIGWRAKWLLEPSDDKAD

>IbDEAH40

MALKCHKNTEVVFDHAAGDTVCSECGLVLESRSIDETSEWRTFADDSGYHDPNRVGGPVNPLLGDVGLSTVISKGPNSNGDASVARLQNRGGDPDRALVMAFKAIANMADSKRTWRQMEGTAGYLGYWQYGYLRGHEGKQQLPQVSSPLTRVFGAQLLANAAADKNKLLLQMEVPRKPPSQKRATVKPIKPEAVIVISPDTKEEAACGLNIKLPKEKIEDIDAGDVYNELAVVEYAEDIYKFYKEAEDPAEVDKPLKIQRELDETINILPPWDLDVENKFILHFLYGGDYNMKAGQELAEDMAITQHSRNRRIKGGTLIVCPMALLGQWKDELEAHSKPDSISVFVHYGGYINGGSSIFHKVDWHRIVLDEAHTIKSWRTVSARAAFTLSVHCRWCLTGTPLQVHSYNRNGLCVLGFMDDHYPVRSAFSLLNQDPAEADKLLKIQWELDGTKIFLVGFNSQPSKLVGSRGSINRYFDFDNWQRVTAIVTTNLNKIIDVNFYPIETAKSSNLRRRRKALYETYEGSPISKKIPEIPDELKLIYKTVWEIKQRTLVDMAADRGCYIDQSQSLNIHMDQPNFGKLTSMHFYAWSEFISLHISDLICLCVLIDYLTYTDTQGLKTGMYYLDHVQRLMDLFEIPAPAWMNVSLRMKHVPENDNNLINKKALALQVELCYEKNGGVATFLFFSLTMFLKVWYD

>IbDEAH39

MEEMMFFYVNMNMIYTGIVSCETLMMAKSSLLKWHEGKQQLPQVSSPLTRVFGAQLLSNAAANKNKLLLQMEVPRKPPSQKRATVKPIKPEAVIVISPDTKEEVKEKNSLRKAAAEDSSRKTYTATLTARSKAACGLNIKLPKEKIEDIDAGDVYNELAVVEYAEDIYKFYKEAEAGQELAEDMAITQHSRNRRIKGGTLIVCPMALLGQWKDELEAHSKPDSISVFVHYGGSVSMFSLELESDGGSSIFHKVDWHRIVLDEAHTIKSWRIVSARDAFTLSVHCRWCLTGTPLQNNVQDLYMSIGIGVQGLADTFTLLGMAFYSPEAQDLNTEIFETIYYHGLKASSDLAAKEGPL

>IbDEAH34

MFQKFNFDTEWAVKLISRITNDEDTPIEGDALLKQLLSLQISVEKYFFGIRKSLVEFDEVLEAACGLNIKLPMEKIEDIDAGDVYNELAVMEYAEDIYKFYNKAMQKTFTTAEGSSGRRAHSVTNFRRPAPGVGKRVSEGFRQKFVLSGLRVSEPIPSWCACRLDNFSHFVALSSSVSVQQNSNSNGDLWTSIFSGGGQRRFPAVVGDEPAIVVDDEVIESSVADDRQQVSKKVRTGICHGLIIENGSRRMVQDEQFVHLTLTKSSWELGKHSNSLLECALICGVKSGVDNGLEVLTETGIGLEGYDSRWEAYSSVGGFIACRALSQRNNDPTRASLPLDSDCDGFVMGEGAGVHRKNLSMRSEQKRGAKSMLNISVEEPHLEGDLTEFQALLRCFGQDPEDLNKEIFETIYYHALKASSDLAAKEAPMPTASTSQILGNNECFEPHTSDIYSRRVLRFLCPPDSVSPNPGLGSCVLDFSRGVFVHNCWLMHKLQQLTKTRNVLVLRMWMELLLQMEVPRKHPSQKRATMKPIKPEAVIVISPGAKEEAACGLNIKLPKEKIEDIDAGDVYNELAVLVVLWMIHTGLNCFVTAKSDAMGLGKTVMTIALILARLGKGIPDNQELAEDMAITQHSRTEGLKVALSLFVLWHYLDELEAHSKPDSISVFVHYGGDISNDPKVIAEPDVVLTTYGLLTAAYKADGGSSIFHKVDWHRIVLDEAHTIKSWRTMSARAAFTLSVHCSWALTGTPLQVLSTSFIMFPIKIMCKIFTAFCVSCMLSHGAIGAWWNELIQRPYENGDKRALKLIKAILRQLMLRRTKETKDKEGRSEILEVGQFKGSLPDFNERNLEFLTSETDLEALQGVLDKYKNAVVEIADSLQLSRNVLIALLQSWKPFMQRWIRAHIMLLDTKVHSPVLDKTSNAAKEILKALPLSDHAIKSTASKFLLSWLFQHEHEYRQWSAAISLGLISSFLHVTDRKQKVEYINALLEVLSVSKSTTCERSLWSWVGFSCQDLLTKSGGKDNFHLSKGALRMEEIDLLKKIIRNLSKFICPLTQSSDVLINAFSWLRLGSDDFDSNITPKFLGQCKDYFEEDVWGVAGLILGLGSSVGVIYTAGFYDAVLNLKTLLFQANTIEEWSEAVRCLGKSEQGWLLDLLQISEVNVIEANGQLESVKKIQVKSILVQTGSVPFSELGKLKAYMMNCRCEGQFLAPMFTPIIWDALLGVAETLHYADGSVKRQWLDPAEADKLLKIQRELDETKIILIVNLSVLCDARKG

>IbDEAH37

MNFTETMILLELHDLGIVTAMCLSWVKELEFCFWKNLSMRRKEMQQSMLNFSMEEPHPEGAITGVVLCMEKAIANSGVHINGFLIIFSSNHPKLTHGDYTLLSWSSAWKNLSMRRFAVQGIFVGEIKQKNMATDGRNPGTVRAEGKLLPQVSPPSHKGFCAQLLANAQAAAADKNKIGVVPCLKSCCCKWRCLTKEEVKEKNSLRRKSAAEDSSRKTYTATLTARSKAACGLNIKLPKEKIEDIDAGDVYNKLSVLDARSMSIFFRGVCWARVSLIIKNLLRIWLLLSILGTEGFKVALSLFVLWHYLDELEAHSKPDSISVFVHYGGDISNDPRVIAEPDVVLTTYGLLTAAYKADGGSSIFHKVDWHRIVLDEAHTIKSWRTMSARAAFTLSVHCRWSLTGTPLQNNVQDLYSLMCFLHVEPWCNWAWWNELIQRPYENGDQRASKLIKAILRQLMLSRTKEIKDKEGRECKLENWRNGKLEEWRSGEMEKQRNEVGQFQGSLPDFNERNLEFLTPETDLEVLRALEAFEVKIINHEHVTRRRVVKQKRLPKSKIEKLLDVFPQFMFASGVLDKYKNAVVEIVQHEHEYRQWSAAISLGLISSFLHVTDRKQKVEYINALLERVLQVLSVSKSTLVKGACGVGLGFSCQDLLTKSDGEDNFHLSKGALRMEEIDLLKDHWELIQMILIQISPPKFLGQCSDDFEEDVWGVAGLILGLGSSVGAICRAVQKSELLFSVGACLAAPIVMAGFLSQSRTDGCTLHQSLLIASCIGAGSLLGTILNGSLHSLKVEHVKDLPALFRKSNSDPNPPLIHLGATIGTVNALGAGAGTLIQDHPSFSSHATNNQKESSYINGPLLSSPALEPDLTSLVQEIFLVAQNPDADQLQQYAAWAASFLRHSLQLQEHYNEATTVSGAGTKNVSHIFSEDSTVLKLSLWLSHLKHPGTGNILHVNTISVVLRCLEHAPRLPSLDWVAIIGRFMRYESEVAELLSQDVTFKKGHLREECLLFSLSHAYQFDHLLIFLDELCDLSRMRTLEPTLQSCILLHLADLIKIFSSSRLVKLFNDVACFLSWEEHHAEISFMVSEFLISLLKEYRVMGNGLYFVQMRHPDWQIAAVKNLRNFIQNMKESAQAQRNCVLQSGFNCFATDLSGKGPLGLVSKADTFILLGMAFYSPEVNYLDATAQDKEIFETIYYHVLKASSDLALQRKTLYETYEGSPISKGTLQPDMWAVTPSNQWDWVALRAMIEKNEVMNSLLAAPMPTASTSQILGNN

>IbDEAH41

MAQFWKPGTERPRLVDDEDGGVLLYTSSPSSSSAFGYGNIEKQRQRLPVHKYRTAILYLVETHATTIIVGETGSGKTTQIPQYLKEAGWAEGGRMIACTQPRRLAVQAVASRVAEEMGVKLGEEVGYTIRFEDITNTDLTRIKFLTDGVLLREMMDDPLLSKYSVIMIDEAHERSISTDILLGLLKKIQRRRPELRLIIASATIEAKSMADFFITRRRRLSEQEENGPSREPAILSVEGRGFNVEVFYADNPVSDYVQSAVSTVLSIHDHEPMGDILVFLTGQDDIDTAVQMLTDEAHSKQKKGLIFVPLYSGLPRADQDIVFTPTPRGKRKVIISTNIAETSLTLEGIVYVVDSGFSKQRFYNPKHLLLADFQMGHQLAMTSLVNINWKISDIENLIVAPISKASARQRAGRAGRVRPGKCFRLYTEEYFLNEMSPQGIPEIQRSNLVSCVIQLKALGIDNILGFDWPASPSPEAMIRALEILYSLGVLDDDAKLTSPAGFQVAEIPLDPMISKMILASSESGCSEEIITIAAVLSIQSIWISLRGVQKEMDEAKLRFAAAEGDHVTFLNVYKGFIQSNKSSKWCQKNFINYHAMKKVMEVREQLRRIVLRLGLSLKSCEGDMQVVRKAITMGFFANACRLEAFSHNGMYKTVRGSQEVYIHPSSVLFRVNPKWVIYQSLVSTDRQYMRNVISIEPSWLREAAPHFYQPQHPNSIPR

>IbDEAH43

MDSDSDSDGSHISATPPSSPQPKPRPAPPPKALLLSSTKFRTKLKPTGATAGKASRPIIRPKSCRKPKKPPPDSTPVELGPEKPPPPVQPPPKLPPPDTANLPFQIHRSTYSNQAFASTTNDSIETFIPGDLRPSKFASFSKIQKKELNFESVEPDSFGTSSFSASQSEAEAGEANIAPQESETKVATSGNSAKILKRFPNLIGRDSSASLPVKKPKCVNEGNFVKLNINGSGRKYAYKGKRKKFGSYSTGRKFYRSKRKFKGKGQGEKEEGGVYDEEGLAMDFKQREENLNYDEVLIQEAVMSVRTEASDENLLRLLKVTYGYDSFRSGQLEAIKMLLSGKSTMLLLPTGAGKSLCYQLPSMVLEGITLVISPLVALMIDQLNQLPPALPGGLLCSSQTPEETSETLQSLQEGSIKVLFVSPERLLSSDFISIFSSSPLISLVVVDEAHCVSEWSHNFRPSYMRLRASLLRARLKAECILAMTATATVKALHNVMQALDIPSTNLIQTTKLRNNLQMSISMSSNRLKDLMALIKSSPYSDVKSIIIYCKFQSETDIVCKYLRDNNISAKSYHSAIPAKDRRRTQELFCANKIRVVVATVAFGMGLDKQDIGAVIHYSLPESLEEYVQEIGRAGRDGRQSYCHLFFDDISYYKLRSLIHSDGVDEYAVNKFLCQVFSNGTGSFGKVYSIVKEAASRKFDMKEEVLLTILTQLELGEVKYLCLLPEMNVTCTLNFHQTSPALLAAKDIVIAAVMKKSEIKDGQYVFDIPTIANSIGLQPADLSNHLQSLKDQAYCFTVMDIPKDICSLAAQLTNWLSEVERCKVRKMDAMFNAAVAAVKGCDKVHGCNDYDHTPCLQRKILEYFESNDDIDVPNKMAQCSPFLRADIKVFLQSNSHAKFTPRAVARILHGLSSPAFTSAFWSKCHFWGRYMQTDFDVVMEAAKAELMGFVGKDSL

>IbDEAH46

MPTYKIRGIDVDFPFQAYDCQLVYMEKVIQSLQNQTIGMVPECQLELSLTRGIIINQRCNALLESPTGTGKTLCLLCATLAWRKSLGAFSSRKYERRGQNGSTQELDPYSQSQSESSRLPAIVYASRTHSQIKQVINELKRTNYRSELDCDVWLTFDVQLWPKMVVLGSREQLCIHEEVQLFFAFNSAYCVCSLLNKHCIAKFVKGNPNLGDEPIDIEDLVNIGRSSSTCPYYISRDLHKSVDILFAPYNYLIDRENRKSLSIEWSNSILIFDEAHNLLASLRQKNCVDLSIARREKSSDKSCNPDNFAILRALLLKLEKRIGEVPIDSKELGFTKPGTYMYELLADLNINQKTANMLIDIIEEATLLLEEDASVANNEGLNKSKGSVCRLESMGDILRMIFRDDGNPHAKYYRVHVQEVEGSGPDAFKGKASRTLSWWCFNPGIAMEEFSRLGVASIILTSGTLSPMDSFAEELKLEFPIRLENPHVISENQVWAGVVPAGPSGYPFNSSYRSRDSLQYKLDLGNAIVNLARIVPDGLLVFFPSYYFLDQCIGCWKSTGNGNQTNSSSIWERICKHKLPVVEPRQSSLFPSAIEDYMSKLKDRSASGAAFFAVCRGKVSEGLDFADHAGRAVVITGIPFATRNDPKVRLKREYLDQQTQLQQSISKGLTGEEWYSQQASRAVNQAVGRVIRHKHDYGAIIFCDERFTNPNHQARVSLWIKPHIKCYSKFGDVVFSLTRFFRNGGIHGPTKLEMMRPNARENASQVKDCKPQLDIKISNPLTLPVEQPRSVDSVSSALKAKHCRSSSNLDEIVPANRSSLKSDKLVQKLDMKRSSSLFEFENKFLPSLRKTTLASNHKLIDLTERTLLDKKSDEVIAPCSSKRPRLVITGPDKVCCKSSSENQCCNMLNDEKLTLSGSQDKIEMLPKDEPTLQTKVTLSNDHGKPSGSVTSSEDAENKGSMFLVQVREKLSDVEYKEFVGYLKALKSKAMKIGHVLESVARLFSLPDRLPLLHRKMGDVEDERRGRKKGAMAATPTLHSRTQSSFEDNLEKMTTDDFSGRRMRKCYSDNSGNRCSHDEQPPASAAAKPTKNRRRPLQKRSLLSCPNRLHHLFSPKDINAIILSSENARLRCSIIIAVLVVMSCVSVPHGMTKSNSVIASRPLYIIILTDVTIVVARLLILGKPEDPDTAAGGHNEDDEYNWSQAFAMLEIDSATIVYMGGFGFESSEVILSLYASIG

>IbDEAH45

MSLNAFKEALKPCNNQSSSNSSQTSLISTQFDSSTVNPRKPPKSSLSRQLLLLEDSFTSSSWTQSQRPQKQTHSSIRVKDEKEEEEAVEEEKPLTFGRSKLESFVLDHTGPYEPFVLSPPGETPVVQVPASINCRLLEHQRVGVKFLYGLYRNNHGGVLGDDMGLGKTIQTIAFLAAVYGKDADVTDSISITGDLERKGPVLVVCPTSVIHNWENEFSKWATFSVIIHGSILSDVQWEIVIVDEAHRLKNDKSKLYQACLEIKTKKRYGLTGTIMQNKIMELFSLFDWVVPGCLGTREHFREFYEDPLKHGQRSTASNRLVQVADERKQHLVSVLQKYLLRRTKEETIGHLMLGKEDNVVFCAMSALQKRVYQRILQLPDIQCLINKDNPCSCGSPLKQVECCKRIVPDGIIWPYLHRDNPDGCDSCPFCLVLPCLVKLQQVSNHLELIKPNPRDDPDKQRKDAEFAAAVFGPDIDLVGGNTQNESFMGLSDVKHCGKMRALEKLMFTWISQGDKILLFSYSVRMLDILEKFLIRKGYCFSRLDGSTPTGLRQSLVFLISTRAGGLGLNLVSANCVVIFDPNWNPAQDLQAQDRSFRFGQKRHVVVFRLLAAGSLEELVYSRQVYKQQLSNIAVSGKMEKRYFEGVQDCKEFQGELFGICNLFRDLSDKLFTSDIIELQENKGKEDGGSLNLSDLGMHFLPEKVITGLSSLTSECQEPKKGDTKLKLEDLGIVYAHRNEDVVNLRPMAIPTSEEQNMQQQKFPGVGRRKLDATNGKEKAGNSDEVKMHKKRQYCRIAQFMGMKELQFSKWILSATPAEREKVLKDYKKRKEKVLNG

>IbDEAH49

MPSMANHVNAPNKNHQNQLNNRKGDFLRRQNIEKQRKSLPIATVEKRLVEEVHNNDTLIIVGETGSGKTTQLPQYLYHGGFCNDGRIIGVTQPRRVAAITVAKRVAEESGVELGQRVGYAIRFEDVTSNLTRIKYMTDGLLLREALLDPYLSKYSVIIVDEAHERTVHTDVLLGLLKNVQKARSKNVGGGVNIDSMKTKNELLEDKKEANSDGIFKKFHAKKYTPLKLVIMSASLDARVFSEYFGGARAVHIHMEEGPGDILVFLTGQEEIESAERLVHDHLQHLPEGNRKLLTFPIFSSLPSEKQMKVFMPAPAGYRKVILATNIAETSVTIPGIKYVIDPGLVKARTYDADMGIDSLIVVKTSKAQALQRSGRAGREGPGKCYRLYPENEFEKLDDSTIPEIKRCNLSNVVLQLKALGIDNIVDFDFIEKPKSIVIIMNIALSPSPVLLYNRIALVKSMETLFLLGAITENNELSDPTGHQMARLPLEPMHSKALILAGEFGCLEEMLISVAMLSVESIFYAPREKLEESRNALKSFASLEGDHLTLLNVYRASNEFFEKSKITNGELKAEKNLRKWCKDNFINSRSLRHARDVHSQIRRNVEQMGLRISSCGDDMLIFRRCLAASFFLNAALKQPDGTYRVLSSGSTVQIHPSSVLFRAKPECIVFNELVQTNYTYVRNISRIDYLWLAELAPHFYALQD

>IbDEAH47

MQSGFLAGGAKTMKARENARRQPPRRRTRKAEINDPVPEPEPHEGPDMEVEKARLISLAIEFGFDEDSARKCLDSLIQLYGEDGKEFITVEHFGDDFLTALADSLEDTEDWEQAVELEACGTLADILDKNYEAENDGNTPCYILEDSPEGKGRMNPVLLDSSSDSEDMDFEKPSKSNTPLTPAFGMNARPSSSAFGRDRSDEGVASSSHRHPSRSTLIVYTWVIVLILNSMSSTSCARHDFCRSSIYPPVSTVTQGSISPLSSKMRPSWASKSEGVTLTYEELQRLDDMELANVVVFGNQSFRPLQHQACQASLQKRDCFVLMPTGGGKKSVLPASCNSATWCYSCSVTITVSHSRPDNHINSKVWNTSNLFEFSAKFFTIGCCIARIKARKDKPSCKLLYVTPERIAGNLTFQEVLNSLHRKGQLAGFVVDEAHCVSQWGHDFRPDYRVLGCLKRNFPSVPVMALTATATRTVREDVLSALNIPHALVLETSFDRSNLKYEVIAKSKEPLKQLGELLVNRFKNLSGIVYCLSKSECVDVSKFLNEKYKIKTAYYHAGMSARQRVTAQRRWHSGEIDVVCATIAFGMGIDKPDVRFVVHNTMSKSIESYYQEAGRAGRDNLPATCVVLYQKKDFSRVVCMLRSGQGYKKQSLKLAMDQARKMQKYCELKDECRRCALVGHFGELINRSSCRNGPSPCDNCLKFSS

>IbDEAH48

MPSMANPVNAPNKNHQNQLNNRKGDFLRRQNIEKQRKSLPIATVEKRLVEEVHNNDTLIIVGETGSGKTTQLPQYLYHGGFCNDGRIIGVTQPRRVAAITVAKRVAEESGVELGQRVGYAIRFEDVTSNLTKIKYMTDGLLLREALLDPYLSKYSVIIVDEAHERTVHTDVLLGLLKNVQKARSKNVGGGVNIDSMKTKNELLEDKKEANSDGIFKKFHAKKYTPLKLLIMSASLDARVFSEYFGGARAVHVQGRQYPVDIFYTLKPETDYIDAALITIFQIHMEEGPGDILVFLTGQEEIESAERLVHDHLQHLPEGNRKLLTFPIFSSLPSEKQMKVFMPAPAGYRKVILATNIAETSVTIPGIKYVIDPGLVKARTYDADMGIDSLIVVKTSKAQALQRSGRAGREGPGKCYRLYPENEFEKLDDSTIPEIKRCNLSNVVLQLKALGIDNIVDFDFIEKPKSIVIIMNIALPPSPVLLYNRIALVKSMETLFLLGAITENNELSDPTGHQMARLPLEPMHSKALILAGEFGCLEEMLISVAMLSVESIFYAPREKLEESRNALKSFASLEGDHLTLLNVYRASNEFFEKSKITNGELKAEKNLRKWCKDNFINSRSLRHARDVHSQIRRNVEQMGLRISSCGDDMLIFRRCLAASFFLNAALKQPDGTYRVLSSGSTVQIHPSSVLFRAKPECIVFNELVQTNYTFTSSEVNPELHNLVSVVITPPNYKHNLHGISNSGDIEDLQHRLEAFHLQVWLQKGDSNTKYFHRFASHRKKNNTLLRLKNHHGEWVEEAAMHSEVFSYYENIFMSTTCDSEILSVVQPKITGDELKSATRLNLMSSCYKVLANRLKDVLDVIISPSQSAFVQDRLITDNIIIAGEVGHYLQRKRVGNDGWAALKLDMAKAYDRMEWSFLEGTLVALGFDSRWVDLIRLCVPTVKYNILVNGDSVGSLIPTRGIRQGDPLSPYLFIICAEKRFLPGGKEVLHKSVTQAMPIFAMSVFLLPVNICDNIEKSMNRFWWNKGSAPASLICAIGKASYVWRSILAGQSVPREGIARRVGNGRDTHVWGSPCLANSGDPFLHTDCPDHLRDARVCNLLDESGTWDEELLHDLFVESDVIRILRTPITPTMSDVCSHSLASTSYLPRSGGDDYEPDILRFVQMVSIAISAVCFRSVYPSITMGDNPRFMLYASEHYHGYFHRLATVENKNQEMSISSPIDDY

>IbDEAH52

MLFNVKGESHEAMKAIGVRNLALETSSLNLIEQKPHAREAMAVRSTICPVSNVRLSDPLAEKAWQLLSSLRLSSKSYTKPGKTLPLTIDASASAIGKSGQANKKWSSNVNSTSFEHAPLHQNFSIKYCIYIDGKYFSESSPDRSASSGADDANKINGSNRTFVAGVDDDDILEHIDVDQIVMEHYQSNCTPQPSVSKFPSFTPVTSSKCMGRSEDTNLPLELSSNCSHGLQLGLCPEASNHLQDMKDKLICISNDLLDNVTNLSPDQVENLRKERLQLNTQIQQLEKYLRTVSVNEERIMSQFSASTTTSAFQYETPRTVPFRIDPMRLETQFQYNGPVGFDQWNSSSMSFASNDISTAPVNREAYIPTYIDVNYVDGSSDKKWSSRDFPWTKKLEANNKKVFGNHSFRPNQREVINATMSGNDVFVLMPTGGGKSLTYQLPALISPGITLVISPLVSLIQDQIMHLLQVNIPATYLSASMEWAEQQEIFRELNSGVCKYKLLYVTPEKVAKSDVLLRHLERLHASDSLSRIVIDEAHCVSQWGHDFRPDYQCLGILKQKFPSVPVLALTATATLSVKEDVVQALGLSNCIVFRQSFNRPNLQYSVVPKTKKCLEDIDAFIKENHFDECGIIYCLSRMDCEKVSEKLQEYGHKAAYYHGSMDAAQRAYVQKQWSKDEVNIICATVAFGMGINKPDVRFVIHHSLPKSIEGYHQECGRAGRDGQHSSCVLYYSYSDYVSYCENDVDCRRLLQLIHFGEKFDSANCLNTCDNCSKSQSCIDKDVTGTAKQLVELVKMTGQQFSSAHILEVFRGSLNQFVKKHRHENLSLHGAGKHLAKGEASRVLRHLVTEDVLVEEVKKSDMYGSVSSVLKVNEFKANNLFGQGHIVRLRFPSSAKASKPIRAETSQPSRVETSQTTPAKGTLTSMMQSEVDLDLSAKLYNALRMLRTNLVKEADEGVMAYHIFGNATLQLISKRVPRNKCELQDINGIGKIKVTKYGDRVLETIEATIKGHSNSGSSNDSNDSGKRRRESANIIANAEFEEDDFAAESTARSKKKAPKKHRKQNEPPIDYADLGYFDEFMDADLEENSDPQIHGGNGGRVLPSWRAPGTNRIVSRSNLHFTGQYFYAKIIKFTSHHTTIICSSFVQ

>IbDEAH53

MSRKLPLPGIEPGSFGRQFQDSIADFPTSVQAFIASSQSAARRHSHPGAAVRQPDDRSEMGSDLKNWVSDNLMSLLGYSQSTLVNYVIGLAKKASSPNELVSKLIEVGVSSNHKTQAFALEIFSRVEHKAAGPNLYQRQEREAAILARKQKTYALLDDDEDGDGDVSGTVGSNAAPKIKEAEEARRVRRRTSEVDDDGADSSESEEERLRDQREREELERHIRERDAASTKKLTEPKLSRKEEEEAIRRSDALERDEIGTLRKVSRQEYLKKREQKKLEELRDEIEDEQYLFDGVKLTEAEYKEMRYKKELYDLIKKRTEEADNTNEYRMPDAYDVEGGINQEKRFAVALQRYRDPEANDKMNPFAEQEAWEDHQISKATLKFGSKDTKAKSEDYQFVFEDQIDFIKAAVMEGVNVDQESAVDELETSVAKSAFEKLQADRKTLPIYPYRDELLQAINDHQVLVIVGETGSGKTTQIPQYLHEAGYTKRGMVGCTQPRRVAAMSVAARVSQEMGVKLGHEVGYSIRFEDCTSEKTLLKYMTDGMLLREFLGEPDLASYSVIMVDEAHERTLSTDILFGLVKAIVDIHSSHVDISCSVCFSVQIVDISRFRPDLKLLISSATLDAEKFSDYFDSAPIFKIPGRRFPVEIHYTKAPEADYLDAAIVTALQIHVTQPPGDGDILVFLTGQEEIETAEEIIKHRVKGLGTKIAELIICPIYANLPTELQSKIFEPTPERARKVVLATNIAETSLTIDGIKYVIDPGFCKMKSYNPRTGMESLLVTPVSKASANQRAGRSGRTGPGKCFRLYTAYNYYNDLEDNTVPEIQRTNLANVVLSLKSLGIHDLLNFDFMDPPPAEALLKALELLFALSALNKLGELTKVGRRMAEFPLDPMLSKMIVASEKYKCSDEIITIAAMLSIGNSIFYRPKDKQVHADNARLNFHMGNVGDHIALMKVYNSWKETNFSTQWCYENYIQVRSMKRARDIRDQLEGLLERVEIELTSNLNDLEPIKKCITSGFFPHSAKLQKNGSYRTVKHPQTVHIHPSSGLAQVLPRWVVYHELVLTSKEYMRQVTELKPEWLVEIAPHYYQLKDVEDSMLSLSLISLPLPLCVSFVVARNLVRLTSMSAFHIEAPRIPRSYLMQKSELSHESAAVVLSVI

>IbDEAH2

MQRPGFSDTARRNRPSEKLPYMEAGYCYRPRQPYKNQWKSHLSNRRDRPPEPPKTLPKFVIQFRSSSRGLKRIELDELIEKLPSPPQSSHVFERGCVVGTLFYEQWSEAVEVVVELWRIRLDGGLPMTPSLVENAEVSSKKEELKDRVEFLEAEIEKVSKLLARPKRIALANELLNKKEAFMAERDLIAKRIAEFKNGVSCILLHLEGKYSGEDVYGDSTAVFTFDTEEFNWDHIHHLIKRECRRLEDGLPIFASREEILKQIDSEQATVLIGETGSGKSTQLVQFLADSGVAGNGAVVCTQPRKLAAISLADRVKEESIGCYQEKSIACYPSYSSIHQFDSKVIFMTDHCLLQHYMRDKSLCKISCIIIDEAHERSLNTDLLLALLRNLLHQRHDLRLIIMSATADADQLTNYFFGCRTFYVSGRTFPVDIKYVPSEYEGSFVSGSDTVPSYVSDVVRTVTDIHKTEGEGTILAFLTSQMEVEWAVETFQTPSAIALPLHGKLSYEDQRRVFQNFPGKRKVIFTTNVAETSLTIPGVKYVVDSGRVKESRFEPGTGMNVLKVCPVSKSSANQRAGRAGRTEPGRCYRIYSENDFENMPCHPEPEIRKVHLGIAVLRILALGIKDVQSFDFVDAPSPKAIEMAIRNLIHLGAIACVDDGYELTADGHYLVKLGIEPRLGKIILSCFHRRLGKEGLVLAAVMANSNSIFCRVGTEVDKLKSDCLKVQFSHPDGDLFTLLSVYKEWDAVSQEKRNAWCWNNSINAKTMRRCQETVEELETCLQNELSIVVPTYWHWDPHMHTEHDESLKHIILSSLSENVAMFSGSDQLGYEVALAGKHVKLHPSCSLLNFCQRPTWVVFGDILASAKEYLVCVTAFDFRDLATLFPPPLFDFLKMDAQKLQKKSLTGFGSMLLKRLCGKSNCHINRFVSRMRSLDMERVFENLNEAVDYEYKLLKNECLEKCLYNGGSASSVPTALFGAGAEIKHLELEKRCLTFDVFLSKGNSFDDKELLMFLERNAGDICVVHRFSGVGQDSEEMERWGRVTFVTPNAAEHASALNSVPLSGGVVKVVPSKTMHDGDQNMISSHLLKAKVHWPRRFSKGVAIVKCHPKDITFMVDVFSSVVIGGSIVRCERSIRCSDNIVIKGLDRELSEAEIFEMLTALTDRKILDLFLLRGNAVEGPPLVFPPEPKDTFMKAAIAFDGSLYLEAARALEEMNGKVLPGCLPWQKIQCQHLFSSSVSCPAAVYHVIKTQLDTLLASFRHRKGVECNLVRNENGSFRVTICAGATKIVTEVRKPLEQLMKGKIIDHDGITPTIDMAEKKFVDLLLSLHENKQLEVHLRGEALPPNLMKSVVQRFGADLNGIKQMFPEGNFSLNVRHHCISISGPKEVKQKVEDVIYEMAQTSSPQNQRNDDEADCPICLCEVEESFKLENCFHVFCRSCLVEQCESAIRSREGFPMCCMHKGCKAPILVADLKSLLSIEKLEELFRASLGAFVAASGGSYRFCPSPDCPSVYRAADPSATYRGTIHLRSMLCRNMHELPPRASSVHFMCQVPRD

>IbDEAH6

MQGLTPYEKKKYVWKMLYIYMLGYDVDFGHMEAVSLISAPKYPEKQVGYIVTSCLLNENHDFLRLAINAVRNDIIGRNETFQCLALTLVGNIGGREFAESLAPDVQKLLISSSCRPLVRKKAALCLLRLFKKNPDVVNADGWSDWMAQILDERDLGVLTSSMSLLVALVSNNHEAYWSSLPKCVRILERLARNQDVPQEYTYYGIPSPWLQVKTMRALQYFPTIEDPNTRRSLFEVLQRILMGTDVVKNVNKNNASHAVLFEALSLVMHLDAEKEMMSQCVALLGKFIAVREPNIRYLGLENMTRMLMITDVQDIIKRHQAQIVTSLKDPDISIRRRALDLLYGMCDVSNAKDIVEELLQYLSSADFAMREELSLKIAILAEKFAPDLSWYVDVILQLIDKAGEFVSDDIWFRVVQFVTNNEDLQPYAALKAKEYLDKPAIHETMVRSTSSQLSTYCFTMHFVLFHPCIEFGIGNPLEQVDLESYICQKNDEMRFELQFTAYSPSPWSSLLIEGCARENGQPPMTRASVLHCNGILLHNPTPKRLTKHFSSPVSAYILGEYSHILARRPGCSPKEIFSSIHEKLPTVSTSTIPILLSTYAKILMHTQQPDPDLQNQIWAIFRKYESCIDVEIQQRAVEYLELSKKGAVLKDVLAEMPKFPERQSALIKKAVETEADTADQSAIKLRAQQQTSNALVVTDQHLTNGSPPVNQLGLVKIPTMSNVDNSSADEGVTQANGTLTVVDPQPQPSSTPSPDLLGDLLSPLAIEGPPAGGNQADTNLVSDAKGAPMPPEALALAPVEEQTNTVQPIGNIAERFHALCLKDSGVLYEDPYIQIGIKAEWRAHHGRLVLFLGNKNTAPLASVRALVLPPTHLKMELSLVPETIPPRAQVQCPLEVINLHPSRDLAVLDFSYKFGTQAVNIKLRLPAVLNKFLQPITVTAEEFFPQWRSLSGPPLKLQEVVRGVRPMPLLEMTNLFSSLQLMVCPGLDPNANNLVVSTTFYSESTRAMLCLIRIETDPADRTQLRMTVASGDPTLTFELKEFVKEQLVSIPTTAPGPAMPAPSQPRAASPPPAASDPGALLAGITGMGNKVPEELVSTVRSIVGDEYTEMDIIRALHMANNDATAAINIIFDTPGSTTRRSSSEDKKCERSSNSDNGSQQKTENRESNNGCRSDADGCEMESEWWFVGTSEVSGLSTCKGRSLKPGDEVYFTFPAEKKLNSPSLGKFGRGRQVVACSEIVRFSSKALGEIGRIPNEWARCLLPLVREKKARVEGYCKSAPNVLGIMDTIDLSISVYINSSMFRKSHKTLLKVATNNSTDESIVYPLPTLFRLLRLTPFQKAEFTPGDLYMRKRRLTEENSSGIHTPSLHANKFKKLVTNEGEAEGDESISDTDLENIVGFADNSKLEEMEPPSTLQCELRSYQKQALHWMTQLEQVHSVNDAKTTLHPCWEAYRLADKRDLVIYLNAFSGDATTEFPSTLQMARGGILADSMGLGKTIMTIALLLSCTERGGSPGSQSTSLPSHENGDTSDISDQSPTPSKKAARFPGLEKFLKQKPTLKSGGNLIVCPMTLLGQWKAEIEMHACPGTLSLYLHYGQSRSKDPKFIAQSDVVLTTYGVLASEFSSENAEENGGLFSVRWFRVVLDEAHTIKSSKSQISIAASALIAERRWCLTGTPIQNNIEDVYSLLRFLRIEPWGSWAWWNELVQKPFEEGDERGLRLVQSILRPIMLRRTKSSTDREGRPILVLPPADIQVIYCELTEAEKDFYEALFKRSKVKFDQFVEQGRVLHNYASILELLLRLRQCCDHPFLVLSRGDTQEFSDLNKLAKRFLKGGQKTGENHVEDVPTRAYIQEVVEELRKGEQGECPICLEACEDAVLTPCAHRLCRECLLASWRSPASGFCPVCRKTVSKQELITAPTDSRFQIDVEKNWVESSKVTALLHELEQLRAVNSKSIVFSQWTAFLDLLQIALARCGLFSLCYLCLLRLNDIPFLRLDGTLNQQQREKVIKRFSEEDSVLVLLMSLKAGGVGINLTAASNAFVLDPWWNPAVEEQAVMRVHRIGQTKRVAIKRFIVKGTVEERMEAVQARKQRMISGALTDQEVRTARIEELKMLFT

>IbDEAH7

MEKMMLEKTNQCGSSSFHLWRRCGSPSPLSHHHHLTHPSAPTTSPHLSFQQIASMEAEHYQFGPYEINPKEVFYSSKLSYALVNLRPLVPGRLGGLWELFYLIWREVKRFIDLTADETSDLWSAAQKIGRQLESYHNASSLTFAIQDGPQAGQTVPHVHVHIIPRKSGDFEKNDEIYDAAPAAEAMENMDSTLKKYFGYSTYRPYQKEIIEKILEGKDCLVVMATGSGKSLCYQAPPLITKKTAIVISPLISLMQDQVMALKERGIKAEFLSSAQTNRSVQSNAESGLYDILYMTPEKACLLSTSFWSRLLKSGICLLAVDEAHCISEWGHDFRMEYKQLDRLRDALVEVPFVGLTATATEKVRRDIMNSLKMKDPHVAIGSFDRKNIFYGVKSFTHGSTFVNELVEEISKYVENANSTIIYCTTIKDTEEIFRSLIAAGIKAGIYNGQMSNKAREDAHRSFIRDEFYVMVATVAFGMGIDKPNIRHVIHYGCPKSLESYYQESGRCGRDGILSICWLYYTRSDFAKADYYSREAQSVCCPSVIVLGWQISKAIMESLCCCTTLLLAGRNCDNCTSSKKENDVSREAFLLIACIQSCGGRWGLNLPVDVLRGSRSKKILEAQFDKIPFHGLGKELPANWWKALAYQLISRGYLIEKFDDVYKFVRVSPKGLQFLKSCNPDYQPPLFLPMTSEMVVDEGSGDTSTETRGNNGLASTEFEGLSQAETQLYKMLLEERMKVAKVNGTAPYAICGDVTLKKISLTRPSTKPRLANIDGVNQHFMKTYGDHFLQSIKHLCEGLNLSLDGERCTQNIQPSVSAKIVTVPSNKKLTPAKLEAWKMWHEDGLSIQKIAEIFMNIASVVSRVGREKLKPIKTELPEEVTYSQIKAYLAMQEFGISENVFPSNHQASLATGELSEVEEPIDHTSSGSPCEEIHSVSNLQTESTVTDEPDFSPISAKRQKIYAPEGRSPMKLEATEESLLSWLKKFDDGASLSDLLEHFNGSTENSLVDLLSNLEGNSSDVYANLRNRPVIAKELEAQGNKD

>IbDEAH13

MAFYRNYSNETVDFEEKRQGQGHRDPGIAGNEEVEATSSDNDDASRLQDGASEDARRIRDEQQSTRIMGVAGKWGSSCWKDSQPMHNNGRSESREESKSGSDYKNEEESEDVSSDGSREDRLESEDDGQQKEAGKGGSVPADEMLSDEYYEQDGDDQSDSLHHRAANHTTSGYTTKLPARPVVASSYTSRKPKTSKACQYDDDADYGDEEEDEDDPDDVDFDPDFGTTSDRRGTKEKDEDWEGEDSDEENNSEDDDDLDILDEANDYYKKTRGKQQTRGGRNVKSTRELKNAAPSARRKRGRTSFEDEESSEQDSEGDSDEDFRSRRSVRKVSYAESEESEELDECKKKKNQKEELEEEDADSIEKVLWHQPKGIAEEAMRSNKSTHPMLLSHLFDSEPDWHEMEFLIKWKGQSHLHCQWKPYSELQNLSGFKKVLNYIKKVTEDVRYRKTVSREEIEVNDVSKEMDLDIIKQNSQVERVIADRIGKDSLDNVVPEYLIKWQGLSYAEATWEKDTDIAFAQDAIDEYKAREAAMMIQGKTVDFQRKKSKGSLRRLDEQPEWLKGGKLRDYQLEGLNFLVNSWRNDTNVILADEMGLGKTVQSVSMLGFLQNAQHIHGPFLVVVPLSTLSNWAKEFKKWLPNMNVIVYVGTRASREVCQQYEFYNDKKAGSSMRFDTLLTTYEVLLKDKAVLSKIRWSYLMVDEAHRLKNSEASLYTTLLEFSTKNKLLITGTPLQNSVEELWALLHFLDHDKFKSKDDFIQNYKNLSSFNENELANLHKELRPHILRRVIKDVEKSLPPKIERILRVEMSPLQKQYYKWILERNFHDLNKGVRGNQVSLLNIVVELKKCCNHPFLFESADHGYGGDANFFGSTKLERVILSSGKLVILDKLLDRLHETKHRVLIFSQMVRMLDILAEYLSLKGFQFQRLDGSTKAELSQQAMEHFNALGSEDFCFLLSTRAGGLGINLATADTVIIFDSDWNPQNDLQAMSRAHRIGQQEVVNIYRFVTSKSVEEDILERAKKKMVLDHLVIQKLNAEGRLEKKESKKGAVFDKNELSAILRFGAEELFKEDKNDEESKKRLLSMDIDEILERAEKVEEKGADEEQGSELLSAFKARISVANFCGAEDDATFWSRWIKPEAIAEAEEALAPRAARNIKSYAETNPLVETNKRKKRGTEAQERFPKRRKADTGYSAPAIEGAAAQVRGWSYGNLSKRDATRFSRVKLVGLLKRLQLKPRLNFFDSLIDGSREAVKGEIVDPKGPLLDFFGIPVKADELLSRVEELQLLAKRISRYDDPISQFRALAYLKPATWSKGCGWNQKDDARLLLGIHYHGFGNWEKIRLDEKLGLTKKIAPAELQHHETFLPRAPQLKERASQLLEMVYQITELPIILNFILENEDCIAKLLGPRSALLPREVAAIGGKNPNVKVGRKGNKKQKDSLPNITAPHSKAKHGKPSNKGLAQKPQKTEQLVKEEGEMSDNEEVYKQFKEGKWMEWCEDVMIDEEKTLKRLQKLQTTSADLPKEKVLSKIRNYLQLLGRRIDQIVFEYAQESYKQERMTTRLWNYVSTFSNLSGERLQQIYSKLKQEQQVSGVGPSQMNGSASGFINKGFDTEKFEAWKRRKRAESDAHSQFQQRPITNGVRVPEPNSSSGILGAAPNNGRPFRMHQSGFSQRQGFSSGIK

>IbDEAH14

MEIEMESEKEINLIRSVVGSRVSEPDILKALSCCNNNAAAAINYIIDNPLPPDVKKTVTSTGARISGPVKQEIGEEELKGSDYPEKGVKKEPSWYGEYYKWLDEQDTEKMKKEGEFKVKAEPDFGDDSKAIVIAEPILESDIKPRVKAEPILESDIKPRVIAEPILESDIKPIVKAEPISSSVVKEEKGGEVLSVQPLSARPVSGEYVERFLSCLPGRKTEKKVDMTLSTVVIEDGDFPEEADWMLVGRNVVTGLSTTKGRKIENNEIVHFAFPKAVSSRYSKVTSSIVRFSTKRFGEIGRLPMEWGNCLIPLVNSKKVKVLGRCVAAPANLQLMQEIMLYVSFYIHSSVFKDGDKSSWRIDCPSEIETTIYPLLTLFKLLKINPFQKAEFKPDDFDSRKRPLDLEGDSVGVVAKRIKGCIEQNKTEQGLSESTLNKLVGAAEMYDLKEIEPPETLVCSLRPYQKQALYWMSELEKGTEAEQTAKVLHPCWAAYRICDERGPAIYVNIFSGEATAEFPSALETARGGILADAMGLGKTVMTIALILARPGKGIPDNQELDEPITQHYRNRRIKGGTLIVCPMALLGQWKDELEAHSKPDSISVFVHYGGDRSDDPRVIAEPDVVLTTYGLLTAAFKADAESSIFHKVDWHRIVLDEAHTIKSWKTISARAAFKLSAHCRWCLTGTPIQNNLQDLYSLMCFLHVEPWCNWAWWNKLIQRPYENGDQRALKLIKAILRPLMLRRTKETKDKEGRPILVLPPTDVQVIECEQSEAERDFYDALYKKSKVQFDEFVAQGKVLHNFANILELLLRLRQCCNHPFLVMSRGDTDKYANLNKLAKKFLDTNPDLDKAAETNPDSASQNVPTRAFVEEVVEEIRKGENTECPICLESADDPVLTPCAHTMCRECLLSSWRNSSCGMCPMCRRLLNRNELITCPSANKFRIDVQKDWKESSKVTKLMDCLQRIRDSGSGEKSIVFSQWTLFLDLLEIPLRKSGIGFLRFDGKTQQKQREKVLHEFSETTEKMVLLMSLKAGGVGLNLTAASNVFLMDPWWNPAVEEQAIMRIHRIGQKRTVCVRRFIVKETVEERMQQVQARKQKMIAGALTDDEVRSARIEELKMLFR

>IbDEAH21

MEMQNINSDNDNNNGLNQQPRGCTMCLGKCRNDLEVGFQEVEAAKLENCVFQFGSKTKPDHETRARRQKINDTFIEHLGRVKGIREILKFHVQQTPPSYSREQQENLEEKNNIGIDILSQTSTQNLMALEAPKEPPRPKTHWDHVLEEMVWLSKDFESERKWKLAQAKKVAIRASKGVLDQASRGEKRVKEEEQRLRKVALNISKDVKKFWLKIEKLVLYKHQLELDEKKKKTLDKQLEFLLGQTERYSTMLAENLVSSPTQCQQLNVSSCQEHLRIQHEEGTQDDVKRNAEEKVESQSVAPDKDDDYDLMSEDGSEDDEHTIEEDEALITREEREEELEALQKEMDLPIEELLKRYHKEQASRGSSPDENGDVPEATVPREDNAKDSDFAITPEIKRVISSASPGRGCVESNGVLSVSDNHLPELEPDKHSKPPKKLQESDKVHSLDEINDEQDGDDDEDFVATFEETECYMDDETTLLEEEELAKAEPNDATNEITLLEMDGKIPIAELLARYKKDYENDENMGSDSESSFASDSEEFSDSPTQKESEQNNDSRDVQPIACPEMEEKEADAVVNSGEEKESENRLADAAAAARSAQPTGNTFSTTKVRTKFPFLLKYPLREYQHIGLDWLVTMYEKKLNGILADEMGLGKTIMTIALLAHLACEKGIWGPHLIVVPTSVMLNWETEFLKWCPAFKILTYFGSAKERKIKRQGWLKPNSFHVCITTYRLVIQDSKVFKRKKWRYLILDEAHLIKNWKSQRWQTLLNFNSKRRILLTGTPLQNDLMELWSLMHFLMPHIFQSHQEFKDWFCNPISGIVEGQEKVNKEVVDRLHNVLRPFLLRRLKRDVEKQLPSKHEHVIYCRLSKRQRNLYEDFIASAETQATLASTNFFGMISVIMQLRKVCNHPDLFEGRPIVSSFDMNGVDVCLSSSICSMFTHGPFSAVDLIALGFLFTHLDYSMASWESDDIQAIATPPGLFQGLGNLETGSGLKNQKKLHGSSIFEEIQRELMADRLKEKKERAATLAWWNSLKCKRKPIYSTGLREVVTVKHPVRDIHSQKINPFSYCSSLANIVLSPVARFQQMVDQVESFMFTIPAARAPPPVCWCSKSGASVFFHPTFKERCTEVLSPRLTPFRPAIVRRQLYFPDRRLIQFDCGKLQELAVLLRRLKSEGHRALIFTQMTKMLDVLETFINLYGYTYMRLDGSTLPEERQTLMQRFNTNPKIFLFILSTRSGGVGINLVGADTVIFYDSDWNPAMDQQAQDRCHRIGQTREVHIYRLISESTIEENILKKANQKRALDDLVIQSGGYNTEFFKKLDPIELFSGHRTLSSKNAQSEKSSDGTEVPLSNDDVEAALKNAEDEADYMALKKVEQEEAVDNQEFTEEAIGRMEDDEFGIEEEIKADDTDNVGLTTSNKEKAAVSNGSDPTEDRAITLAGKDEDVDVLDDVKHMAEAAAAEGQAILSFDSQLRPIDRYAVRFLELWDPIIDKTAVELQDQFEEREWELDHIEKLKEDMEAEMDDDEEPLVYERWDADFATKAYRQQVEALTQIQLMEEMESKAREKELAEYENSIGNDVAAPSKPKSKKKTKKTKFKSLKKGGLGSETKSMKEESPIDLMSTDDEIICQEDVTTPDFVSPHSAQFRKRKQAPDDEESKQMKKSKKFKKASEVSPLSLDLSLPGMQQDESIDLKHRERSTVDLELKPLHKSKMGGRVSIAMMPVKRIFTLKPEKLKKKGNLSSKDYFPSADQWLPQEDAILCAAVYEYGPHWRLVSDILYGITGGGLYRGRFRHPVHCSERFRELIQRYVFSASDVINSERANNISSGKGLLKVTENKIIVAQRSISVKHFLVTFLRKHPNIVRCCLGAARSRSSSQSGFNPLLLTSTANHFSQNSVRPPQGKLAFTNLSQCNKLVGAALSENSGAQTDNSVSISKQREEAPVPAEELDITLELQAAKDDNDISFPPLVHLKILDPDSSPSLKTPTPEHICLKSSQYVAESRFRETSNTCFEACLDWPSITLPPGEPRCKTPAKPQSQGKQKLAPDSSKASKSRSRKVSVEHSDLCPPTEQIFQPASFSNDAIAAMDTSSSLEEACLHHDFETSSLFNTNGEFKAEHTDFVPHSYIPGLLSGLDDCSTSSPTFPEFTDIVLETWTAHVMHLPPSRSDEEWLSPLGCMFQQFASMEKMVRSKKMNEA

>IbDEAH22

MKRSLDYYELSDDDRDEEHSFQLSRVLKTDSAPPPPPIESFAYSKTDTSSKRSNSNVIEIGDSSSEEIEDKVENLDDDDDDEELKAVNKIRSRRRAGTGVELGIEDDEEEQEEEKDEIDVVAKALHKCGKISSDLKRELYGSAGAACDRFSEVEEASSLRMVTQDDVNEACAAGDSDFKPVLKPYQLVGVNFLFLLYRKKIGGAILADEMGLGKTIQAITYLTLLKHLEDDPGPHLIVCPASLLENWERELKKWCPDFTVLQYHGASRSAYSKDLSYQAKAGLPPKFNVILVCYSLFERHSAQQKDDRKVLKRWRWSCVLMDEAHALKDKNSYRWKNLMSVARNANQRLMLTGTPLQNDLHLILHMKFKPMHNVSMKSEEVGAQEVKDTLPSWGSEIVNAQFVHLLYGYC

>IbDEAH23

MRLRLSALSAFEFEAPTLSIPVRQPIVQDYRGQVRASMEAMLTLCASATTDAAAPESDDALCSSRLSPHLSLAASQLSTAAILTVFSLHCVIFFSYGNNNLQPSRGEQNCCLRIDFGFLVTDRNRPKTYGRLSVGHGTTFGRFRSVMKVEFHRRLFGYSVGYVTDRPFAHTYCQMLKGSKAKSNSKLGKSHKKKLEKLKEEEKKKHTLDLHYRKYMIGDNAYSLMLSSSSLGQAETTREKRRREVQYSKAGLEVPSDDQPPKKKTANSILCDAEQESDGMQCTFVVNDCPLQSPVSKDVSDTSSYLGTSHWQNKPHAFDGDSFTPIRHATDETNEPLMPKYMQNPLSTSSCHDEEKTRIMVGVDKNQKAKLADCHPARSFIAPTVVHVSRPTEVEDKRKDLPIVMMEQEIMEAINENTCVIICGETGCGKTTQVPQFLYEAGFGSQSDDGRGGIIGVTQPRRVAVLATAKRVAFELGFHIGKEVGFQVRHDRKVGDNCSIKFMTDGILLREVQNDFLLRRYSVIILDEAHERSLNTDILVGMLSRVIRERQREYAEQHKKVMSGGIVSCKEKIYPLKLVLMSATLRVEDFVSGGRIFHDPPPVIEVPTRQYPVTTHFSKRTEIVDYVGQAYKKVLSIHKRLPAGGILVFVTGQREVEFLCQKLRKASKEIVEKNCKEKNEALSMSALKPTEEKDMQEINEAFEVNENSSHEITDRFNSYDEEHGDTYEDESDMSYDSEDDSDLEVSFDGEDLLLLKLWLGKKAFASGSTGREVAPVTAEGESNASSSQLSNSMVAPSIHRAGPMCVLPLYAMLPASAQLRVFEEVKEGERLIVVATNVAETSLTIPGIKYVVDTGREKVKRYNSSNGMESYEVQWISKASAAQRAGRAGRTGPGHCYRLYSSAVFNDIFSPFSDAEILKVPVDGVVLLMKSMHIGKVIAVLIVFPCALLMDPFSSTCNFRLQISPFPTPPEPTALVEAERCLKVLEALDVKGRLTPLGKVMSQFPMSPRHSRMLLTVIQMMQKVKDYTRANTVLAYAVAAAAALSLSNPFVMEFEQTQSDPAGLKQECERSDEKEEKLRKKKLKEAARVSRAKFLNPTSDALTIACALQCFELSGNPTIFCIENALHLKTMEEMSKLRKQLLRLVFNSKCEVQEDFSWSYGTIEDVEAAWRVCSNKHSLQSNEEEILGQAIYAGWADRVAKRTKRVSGPSERDRKVNAVSYQACMVNETVFLNRRSSVSKSAPEFLVYSELLHSKRPYIHGATSVKANWLVKYGQSLCSFSAPLSDPKPYYCPLADRVFCWVDASFGPYLWDLPRHSLPIEDNMKRVAVFAYALLEGQVLPCLKAVRKFMAASPTSILRPEALGNKRVGNLLNRMNSRGGTIDCCVMLKKLWEMNPRELFPEIMDWFQEGFHDQFEALWTEMLFEIHLNPKDHKETESLKPLHWLKISRAVQGSLWAETQKSETAKAPELDISELESLFSAAVPNSDKASSGRKGNSRTSLGAKAEKVQLLIVLSEILKVPRAFVIQGSLYFLRFKPQRS

>IbDEAH25

MDEEKPVFPAFPYKPYPIQLDFMNALYRSLENGGIAMLESPTGTGKTLSIICSALQWLVDRKQLQNDKPENQSGPDDEPDWMRNFVLNTETKASEKKEKRTSFRKNNFNRKEKQEAFRGIETKETKVLKSNNGSEGMDDEEFLMEEYESEGENGGKLKRKSGEDSVSSSSEDDDDDKEEQRLKVYFCSRTHSQLSQFIKELRKTKFASELTSVCLGSRKNFCINEDVLKLGTSTRINERCLELQKSRKKECCKVTKTKNLGLKGRVRRTKASSGCPMLRSRKVEKEFRSEIAQQGPLDIEDLVHIGRELGTCPYYGSRNIVPKADLIVLPYQSLLSKSSRESLGLSLKDNVVIIDEAHNLADSLISMYDAKVTLSQLDCLHSHLESYLKRFQNLLGPGNRRYIQTLMVLTRAFLQILCHGKNASNAEPFCPAEGTEGSFDFSMAINEFLFSLNIDNINLVKLVYYIQESNIIHKVCGYGDKLAAEKVSASKDSDKSSDGSSLSGFQSLLDMLLSLTNKDSDGRVIVSRTRPSSEGQKGGYLKYVMLSGEKIFSEVLDQARAVILAGGTLQPIEETKERLFPSLQPDKLHFFSCGHIIPSENILPIAVSHGPSGHPFDFSYNTRTSSIMIEELGLLLCNLVTLIPEGIVVFFSSFDYEALVYNSWKESGILARIMKKKRIYREPRKSTEVKLVLREYNDTIEELSHSDPKSHNGAILLAIVGGKISEGINFSDGMGRCIIMVGLPYPSPSDIELIERVKHIESIGCASISKTPKLSAPTQCFNGDAQAGLNILRSCKHRGKQYYENLCIKALNQSIGRAIRHINDYAAILLVDGRFSSNPTERRSSHLTSKLPQWINARLVSSTKNYGEVHRLLHQFFKFHKDKVK

>IbDEAH38

MLRAQNTETVLQRSTGGRQMDDEDEALSASEPSDSSDEFISEPDNDDDDIDYNCNSDDEDDGHGAQTSNLRCSPEDRKSENVAALVRGNLEVRRQSILPRVYSVTDAAVNVRKPFKPPSSNGYSSNNEHLARRLWARKRFVPWGSNRPALVAITNRVNALETTHKDVPEEEICLPPDVEPLLLWQPEGFGEEGCNSKPIAVEPLLVKYLRPHQREGVQFMFDCVSGLLSTSNINGCILADDMGLGKTLQSITLLYTLLRQGFDGKAMVRKSIIVTPTSLVSNWEAEIKKWVGERVKLVALCESTREDAISGINNFTSPHSDLQVLIVSYETFRMHSSKFSNDISCDLLICDEAHRIHMRSLTTDFCIRPWLLCHASAAFCCQGLQCNILFTFIFQNDLEEFYSMVNFTNPGILGDAAYFRRYYEFILRRTNALLSNHLPPKIIEVVCCKLMPLQVELYNHFIHSKNVKRAITEETKQSKILAYITALKKLCNHPKLIYETIKSGSPGTKGFEDCIHLFPQEMFSGRSGSWTGGAGLWVELSGKMHVLARLLAQLRQSTDDRVVLVSNYTQTLDLFAQLCRERRYPFLRLDGSTSISKRQKLVNCFNDPSKDEFAFLLSSKAGGCGLNLIGGNRLVLFDPDWNPANDKQAAARIWRDGQKKRVYVYRFLSTGTIEEKRDFRKLFNKEQADSEIQGNSLSTEDLRDLFTFHDSVRSEIHEKISCNRCQEYEVMLDDTCEAKSTDEQDKSNQEDIGGFAGLAGCLNKLKSSEKQIGTPKEEDLANWGHHFFPTSVPDTIFQAAAGDEVSFVFTCQVEGKLVPIESSVKQKPKPQGGQTNISTLKSNLVQRPAALSPRLAVSSMKEPQEAPPQSPSSGVSSKMSTLPAFFKPVQKQRTNSIKLTRPLDHTQMKSNRVISSRNQLPQKRTSPDAMGDDDFV

>IbDEAH33

MLCGGSDSVIIPTGVGGFIACRALSQRNNDPTRASLPLDSVMTAMGLSSWVKELEFCFWKNLSMRRKEVQKSMLNISVEEPHLEVTGVVLCIERAIANSGVCRDDVSYINAHATSTQAGDLTEFQALLRCFGQDPEDLNKEIFETIYYHALKASSDLAAKEVRDLEYSLQVKFARGTLQPDIWVVTPSNQWDWVALRAKIEKNEVRNSLLAAPMPTASTSQILGNNECFEPHTSDIYSRSSKVSPNPGLGSCVLDCSRGVFVHNCWLMHKLQQLTKIRNVLVLRMWMELLLQMEVPRKPPSQKRATMKPIKPEAVIVISPDAKEEVKEKNSLRRKAAAEDSSRKTYTATLTARSKAACGLNIKLPKEKIEDIDAGDVYNELAVLVCMKSMYFGMISYDSHQAYFFNAIEYKILADAMGLGKTVMTIALILARLGKGIPDNQELAEDMAITQHSRNRRIKGGTLIVCPMALLGQWSNDPKVIAEPDVVLTTYGLLTAAYKADGGSSIFHKVDWHRIVLDEAHTIKSWRTMSARAAFTLSVHCSWALTGTPLQNNVQDLYSLLCFLHVEPWCNWAWWNELIQRPYENGDQRALKLIKAILRQLMLRRTKETKDKEGRLLEGWKNGKWKTFLLKSFLSFPNSSPFSNFMYKFGELLFGLLQMEEWEKTRVNNDIVWRGLAKNLAVRIDLQLRETLLGDKANLRLSIVLMVGQFQGSFPDFNERNLEFLTSETDLEVLRALEAFEVKIINHEHATHRRVVKQKRLPKSKIEKLLDVFPQVMFASGVLDKYKNAVVEIADSLQLSRNVLIALLALQSWKPFMQRWIKAHIMLLDTKVLSAVLDKTSNAAKEILKCLIEIAERSPPRSAENIALAIGALCSALPLSEHAIKSSASKFLLSRLFQHEHEYRQWAAAISLGLISSFLHVTDRKQKVEYINALLEVLSVSKSTPVKGACGVGLGFSCQDLLTKSGGEDNFHLTDDYPPNQLPVDIRGDPAEADKLLKIQRELDETKIILLSLNRLPMSNDLFFCLQHETIDSVRERGEKFDSLVQKSSDLSAASQVNEFLAGGTDAPDVDEENAVSRPSMSYDDMWAKTLLETSEMEFFMLVFIFSMEKIEDIDAGDVYNESAVVEYAEDIYKFYKEAEANHLCRRHLQSFYYSSPSIFYFLQGVGGFIACRALSQRNNDPTRDSLPLDSDRDGFVMGKELEFCFWKNLSMRRKEVQQSMLNFSVEEPHPEVTGVVLCIERAIANSGVCRDDVSYINAHATSTQAGDLTESQSSKLSSVVLARTQRPKPFHFAIVDEVDSVLIDEGRNPLLISGQASKDAARYPVAARLAELLMRDLHYNIEFKDRSVELTEEGIFLAEMALETNDLWDENDPWARQHLTPGFCKISNIWGGFGVDFPQLTGRVENKRRWSDGIHQAVEAKEGLEIKADSVVVAQITYQSLFKLYPRLSGMTGTAKTEHPSKWFLHKLLRDFTGFAGKILNDFFSEITEEALLDSLVQLHDMGSLQVAFLEFPEMRQNWSIEDSKYDDSLIPCVMENRAAECRRIGLFQKCDFAVFYPLCGSVNLLVLGDGFPENIVYMNHEILFKYSYEVKVLRVSDGTQRVCRDFREREYVKRFDSVKMGGEDEFGHAYVDICETLIAGRRVGKMIAGKEGVQVGCGSSGVDVDLGSMFALIACLSAGFLLENLLERDENLNLFYDTLLVLQVMKRVVILSGGTDREREEIVFVSEQCLFQILSLVANPVVNSINAVNPDFGQSTAPAKYFGQSRLLTHRLPDEFLLPARRWYLRILTPKETHTALVSQSGPLGSWVLDSQKAEHIWFNVLSHFHQLYLVMDPYRFLRCWMTARAGKTMGYESKATHTAYPFRSLSQKYTISPSLTHCLKTPQQLEPNLKVASQECPLLSFSGDAILHSCYLINRDAIICFTPSKIPYNPLLPQEPLHKLPLKLSSLWADRMPLPPSAIPPNPSQTHTLLSPFQLFTLSSLGQHRHQPLPQKHLTQARFISLSCSSSMCSPGFQPSHHASVVFFLIPLATSYMVDEMAAFTFLELDCSISPPKNSLLSSCRWVYNANVGPRMDVHCARGSLAIRVSKKESMPHCLLFGDLCLSIWWTNKQSMGENQRKDAEEVEISKIDKDVVEGIPDGQVKCIDLKEHRDCITGFAIGGTDTVPATQVYIINSSFNQI

>IbDEAH35

MAKPSRAKSSESESLSDGSDDERPNDQVNEEEDEEELEAVARTAEDYDEEEEDDNDEATAAGDEEVEEENAAANEIGKREKARLKEMQKLKKQKIQDILDAQNAAIDADMNNKGKGRLKYLLQQTEIFSHFASGDQSTSQKTKGRGRHASRVTEEEEDEEYLKEEEDGLGNTRLVAQPSCIQGKMRDYQLAGLNWLIRLYENGINGILADEMGLGKTLQTISLLGYLHEFRGITGPHMVVAPKSTLGNWMNEIKRFCPVLRAVKFLGNPDERRYIREELLVAGKFDVCVTSFEMAIKEKTALRRFSWRYIIIDEAHRIKNENSLLSKTMRLYNTNYRLLITGTPLQNNLHELWALLNFLLPEIFSSAETFDEWFQISGDNDQQEVVQQLHKVLRPFLLRRLKSDVEKGLPPKKETILKVGMSQMQKQYYKALLQKDLEVVNSGGERKRLLNIAMQLRKCCNHPYLFQGAEPGPPYTTGEHLIENADCRKNGSSGQAASKAKGAWFKGVNIFTVASDFVIKMTRLLDILEDYLMYRGHLYCRIDGNTGGEDRDASIEAFNKPGSEKFAFLLSTRAGGLGINLATADIVILYDSDWNPQVDLQAQDRAHRIGQKKEVQVFRFCTEYTIEEKVIERAYKKLALDALVIQQGRLAEQKTVNKDELLQMVRFGAEMVFSSKDSTITDEDIDRIIAKGEEATAELDAKMKKFTEDAIKFKMDDTADLYDFDDEKDENKVDFKKIVSENWIEPPKRERKRNYSESDYFKQTMRQSGPTKPKEPRIPRMPQLHDFQFFNTQRLSELYEKEVRYLMQTHQKNQLKDTIEMEESEDVGEPLTAEEQEEKERLLEEASQYNGFSTWSRRDFNTFIRACEKYGRNDIKGIAIEMEGKTEEEVERYAKVFKERYKELNDYDRIIKNIERGEARISRRDEIMKAIGKKLDRYKNPWLELKIQYGQNKGKLYNEECDRFMICMVHKLGYGNWDELKAAFRTSPLFRFDWFVKSRTTQELARRCDTLIRLIERENQEFDERERQARKEKKLAKNATPSKRALARQAAESPPQKKKKQLTMDDYAGSGKKRK

>IbDEAH42

MLKGSKAKSNSKLGKSHKKKLEKLKEEEKKAILLSESLETMAKYKIGDNAYSLMWSSSSLGQAETTREKRRREVQYSKAGLEVPSDDRPSKKKSILCDAEQESDGMQCTFVVNDCPLQSPVSKDVSDTSSYLGTSHRQNKPHAFDGDSFTPVRHATDETNEPLMPKYMQNPLSTSSCHDEEKTGIMVGVDENQKAKLADCHPPRKDLPIVMMEQEIMEAINENTCVIICGETGCGKTTQVPQFLYEAGFGSQSDDGRGGIIGVTQPRRVAVLATAKRVAFELGFHIGKEVGFQVRHDRKVGDNCSIKFMTDGILLREVQNDFLLRRYSVIILDEAHERSLNTDILVGMLSRVIRERQREYAEQHKKVMSGGIVSCKEKIYPLKLVLMSATLRVEDFVSGGRIFHDPPPVIEVPTRQYPVTTHFSKRTEIVDYREVEFLCQKLCKASKEIVEKNCKEKNEALSVTALKPTEEKDMQEINEAFEVNENSSHEITDRFNSYDEEHGDTYEDESDMSYDSEDDSDLEVSSDGEDLLNQHSLDSDGKLAVPLGEDGNLTSLKAAFEALAGKKAFASGSTGREVAPVTAEGESNASSSQLSNNLVVPSIHRAGPMCVLPLYAMLPASAQLRVFEEVKEGERLIVVATNVAETSLTIPGIKYVVDTGREKGKRYNSSNGMESYEVQWISKASAAQRAGRAGRTGPGHCYRLYSSAVFNDIFSPFSDAEILKVPVDGVVLLMKSMHIGKVANFPFPTPPEPTALVEAERCLKVLEAFDVKGRLTPLGKVMSQFPMSPRHSRMLLTVIQMMQKVKDYTRANTVLAYAVAAAAALSLSNPFVMEFEQTQSDPDGLKQECERSDEKEEKLRKKKLKEAARVSRAKFLNPTSDALTIACALQCFELSGNSAVFCIENALHLKTMEEMSKLRKQLLRLVFNSKCEVQEDFSWSYGTIEDVEAAWRVCSNKHALQSNEEEILGQAIYAGWADRVAKRTKRVSGPSERDRKVNALSYLACMVNETVFLNRRSSVSKSAPEFLVYSELLHSKRPYIHGATSVKANWLVKYGQSLCSFSAPLSDPKPYYCPLADRVFCWVDATFGPHLWELPRHSLPIEDNMKRVAVFAYALLEGQVLPCLKAVRKFMAASPASILRPEALGNKRVGNLLNRMNSRGGTIDSCVMLKKLWEMNPRELFPEIMDWFQEGFHDQFEALWTEMLFEIHLNPKDRTSVSHSGMKGKALFSRAISCINSQTKKLKPLHWLKISRAVQGSLWTETQKSETAKAPEIDISELESLFLAAVPNSDKRKGNSRTFWGQNLRK

>IbDEAH44

MHSPGVLDTARRNRPPENLSYTESGHCYRPRQPYQHQWNSQFNNRRDRLPGPPKTQPRFVIQFRSSSRTLKRSELDEIIEKLPSPPQSSYVFDRGSVLGTLFYEQWSEAVEVVVKLWSMRLDGGLPMTPSLVENVEVSLKKEELKDRLRGVFLEKLEGLMEGELMQKCRQKLELLEAEIEKGLDSKEDSGVQERGELCCASIGGGKYSGEDVHGDSATVFNFETEFNWDRILHLIKRECRRLEDGLPIFALREEILKQIDSEQATVLIGETGSGKSTQLVQFLADSGIAGNGAVVCTQPRKLAAISLADRVKEESVGCYKEKSIACYPSYSSIHQFESKVIFMTDHCLLQHYMRDKSLCKISCIIIDEAHERSLNTDLLLALLRNLLHQRHDLRLIIMSATADADQLTDYFFGCRTFYVSGRTFPVDIEYVPSECEGSFVSVSGMVPSYVSDVVRTVTDIHKAEGEGTILAFLTSQMEVEWAIETFQAPSAIALPLHGKLSYEDQRRVFLNFPGKRKIIFTTNVAETSLTIPGVKYVVDSGRVKESRFEPGTGMNVLKVCPVSKSSANQRAGRAGRTEPGRCYRLYSKNDFENMSCHKEPEIRKVHLGIAVLRILALGIKDVQSFDFVDAPSPKAIEMAIRNLIHLGAIACIDDGYELTADGHYLVKLGIEPRLGKIILSCFHRCLGKEGLVLAAVMANSNSIFCRVGAEVDKLKSDCLKVQFSHPDGDLFTLLSVYKEWDAVPQEKKNAWCWNNSINAKTMRRCQETVEELQVCLQNELSIVVPTYWHWDPHMHTEHDETLKHIILSSLSENVAMFSGYDQLGYEVALTGKHVKLHPSCSLLNFCQQPTWVVFGDILASAEEYLVCVTAFDFKNLATSFPPPLFDLSKMDAHKLQKKTLTGFGSMLLKRLRGKSNCHINRFVSRMRTLCMDERIGIEVNVDQNEIAMYASTRDMERVFEDLNEAVEYEYKLLKNECLEKCLYYRGSASSAPTALFGAGAEIKHLELEKRCLTVDVFLSKGNSFDDKELLMFLERNAGDICVVHRSSGVGQDSGEMERWGRVTFVTPNAAERASALNSVPLSGGIVKVVPSKTMYDGDQHMITSPLLKAKVHWPRKESKGIAIVKCHPKDVAFMVDDFSSVLIGGRRVQCKLSIEHPDSIEIKKFDSELSEAEIFEVLSPVTDREILDLFLLRGNTVEGPPLVACEEALLREISSFMPKRNPYGNSTRVQVFHPEPKDYRMKAAITFDGSLYLEAARALEEMNGKVLPGCLSWQKIQCQQLFSSSVSCLAAVYHVIKNQLDTLLASFRHRKGVECNLHRNENGSYRVTISAGATKLVAEVRKPFEQLMKGKIIDHGGITPTVLQHLFSREGIILMKSIQQETGTYILFDRHTHTLRIFGSSNKIDMAEKKFVDLLLSLHESKQLEVHLRGEALPPNLMKRVVQRFGADLNGIKEMFPEGNFSLNVKQHCISISGPKEVKQKVEDVIYEMAQASSLQNQRSDDEADCPICLCEVEESFKLENCFHVFCRSCLVEQCESAIRSREGFPMCCMHKGCKAPILIADLKSLLSIEKLEELFRASLAAFVAASGGSYRFCPSPDCPLVYRAADPGTTGEPFICVACYAETCTSCHLECHPFISCVKYREIKDDPDYNSLKAWKKGKENVKNCPDCSLTIEKVDGCNHIECKCGKHVCWVCLEFFATSDDCYDHLRYADGVDKLFMCFGTLGSIGDGLQVPLMMYVLSDVINEYGSLDSAVSIQTVNKYSLRLLYVAILVGLSAFVEGLCWARTAERQTSRLRLEYLKAVLKQEVGFFDTQAAESSTTFQVISTISADSNTIQITIGEKIPDCLAYLSSFFFCFIFAFVLSWKLTLAAIPFTMMFIAPGLGFGTMMMKVAMQGIESYGVAGGIAEQAISSIRTVYSYAAEHQTLEKFSQALQKVIELGIKQGFARGLMMGSFGVVYISWGFQAWLGSVLVSKKGEKGGDIFVAGFNVLMAGLNILTALPNLTAITEAKAASIRISEMIDRDPAIDTEDKKGKALSYVRGEIQFKGVYFSYPSRPDTAVLQGLDATIPAGKTVGIVGGSGSGKSTVISLLQRFYDPIEGEIYLDGYKIKRLHLKWLRSQMGLVNQEPILFATSIKDNILFGKEDASMEDVERAAKAANAHDFIIKLPDAYETNVGQFGFQLSGGQKQRIAIARALIRDPRILLLDEATSALDAESERIVQEAIDHASIGRTAIVIAHRLSTIRMAKLIMVLQQGRVVESGSHAHLMEMNDGEGGEYYNMVRMQQQSGIQHEATPSSHRQKIDKKRRHKMSVPSSPLSVRSSAASTPVMYPFSPALSMSPAIPHSGIYSAPYSVQYEDSFDSDDEDRNKPAYPAPSQWRLLKMNAPEWPRALLGCIGAIGSGAVQPINAYCVGGLIAVYFRADKSTIQSHARIYTYVFIGLGVFNMFTNVLQHYNFAVMGEKLTKRVREKLLEKLMTFEIGWRPDVTASSSHLRGHLCLHFRAFPIVERLALVMMAAQPLLIGSFYARSVLMKSMSAKAQKAQREGSQLASEAVVNHRTITAFSSQKRIVGLFKATLEEPRKESIRQSWFAGMGLFSSQFLATASTALAYWYGGKLLTEGQISPEKLFRAFLALLFTAYTIAEAGSMTKDISRGSDAVRSVFAILDRESEIKPDSSVAVDAKKAEIMVALVGQSGSGKSSIIGLIERFYDPLKGAVYIDGRDIKDYKLRKLRSHIALVSQEPTLFAGTIYENIAYGKKNAKEAEIRKAAILANAHEFISGMKDGYETYCGERGAQLSGGQKQRIAIARAILKNPKILLLDEATSALDTISERLVQEALEKMMAGRTCIVVAHRLSTIQKATSIAVIKDGIVEEQGSHSELLSLAGAYYSLVKLQGSSTPYRQT

>IbDEAH51

MKRRYKRSRPRKKAEKGAQRVEFSDDDNHFVVSSDSDYHTSEDDDIIDISEENEMGGFPFNRLVNDEVGVSDDDALYKVPKRRRKVGSTRRGIEMDTGFKEEKQQNEEVVDGSKFGVGGPHTTSPEFIALINRINERKKKRIKKRKDRPILMWEVWDDENDQWLVENFTTDVEIDNRNEVMSETAEAPSELIMPLLRYQKEWLAWALKQEESASEGGILADEMGMGKTVQAIALVLAKRELKRAIGEHCLPSSSPSTSQGLPAMKGTLVICPVVAVGQWVSEIERFTLKGSNKVLVYHGKNREKCLDRLSEYDFVITTYSIVEFDYRKYVMPPKQRCEWCGKALYEKKMAQHRKYYCGPDATRTAKQSKQQKKKPKYGVKLSMEKTESNEGKAQQNNGSEGNPKKKGRKKGVKSYTIDNECSNDGPFENIANEEQVISGKSILHSVKWDRIILDEAHYIKERRSNTTKAILALESSYKWALSGTPLQNRVGELYSLVRFLQIIPYSYYFCKDCDCRALDYCSSTECPHCPHKSVRHFCWWNRYIASPIQAEGHYGNGRDAMILLKNKILKSVLLRRTKKGRAADLALPPRIVTLRRDSLDVKEEDYYTSLYNESQAQFNTYCLILNFFYSLNLAPDCLSLSLCLPLWTLHLLAMDLCHITSAIDMSGKELCRITTPTFLTSSPPATVLEMVSPDFGLPVIYVQAVDHPYLVEYSVSALARSENAVDATNVGQPCGICHDSVEEPVVTSCAHTFCRTCLEEFSSGMAKVSCPTCSKPLTTGLLNGSGNRDSKNRTTIKGFRASSILNRIVLDEFQTSTKIDALREEIRFMVERDGSAKGIVFSQFTEFLDLIHYSLIKVRFVPGTIPSLTLKESAITKFTEDPECRIFLMSLKAGGVALNLTVASHVFLMDPWWNPAVERQAQDRIHRIGQYKPIRTIGGCSDALGKLTEGDLKFLFVT

>IbDEAH50

MSDEQDPVEQFMVLDQWPFMPFNGDGEDDVTGGSELDGSSSPSSSSSQVGGELLMMGFVIVNIVGLQYYSGTINGRELVSLVRDPLNTHDPNAIKVLNTRSAQVGYIERPAAQVFAPLIDSSLITAEGIVPKVSRHGNRYKIPCQVHIFARIAAFESVKSAISDGGLSLISEGSAAFALSEAAVVKDQVVAGDSNSVDAIFKLLDEKILKKEALGTLEPPRSVVTSELLSHQKEGLWWLVQQENAHELPPFWMEKEGVFVNALTNYSTDKRPDPIRGGIFADDMVEEREDIDERELMDGILAASVGRNSKRGRGSTRANNSRKKLKSEGARTKEMKGEPLEEHTRPGSLKVYMYYGRQRTTDANVLQKYDIVLTTYNILANEEQLLESPIKKIEWWRVILDEAHIIKNVNAQQTRAVNNLNAKRRWVVTGTPIQNNSMDLFSLMAFLRVEPLSIKGYWQSLIQRPLVLGDETGIMRLQRLKDLETGKLSILADSFGNFHEMLDELLVLQKVLMETMSLRRTKEKTLVDLPSKTLETVYVELSGEEREIYDHMELRAKAIVTEFIYAEGSLKSYLTVLSALVRPTNLYRCSHVPPNLGDILPVTKIGDVNSNPKLLDKMLMALQDDDGIDCPVCISPPKNTVITSCGHIFCKICILKTMQRMKSCCPMCRHPLTEADLFFAPSETSDSCGTKADNEISRVLAVPHYAAFARSATESSGFQDAAPRWHDELGPTRSSDQGVRCPGPRWTYHLTCEPESFGGRDKPHHGLQGVPVRAVVEPCCRGTGHGPCPQDWADRGGEDRADDRTQHHRGEDSAAAGEEETVRQQNLREEELAGSEGR

>IbDEAH54

MAEEEGEGKEKAEQTQTQQEQQEDEDIEKASSASDSFIDDGDDESEAAAGEEEDLLPEEPLTEQEIEELIAELLEVESKAAEAQEALEEESLAKVEGDVRAELAQTLSADDLESAVAEEMRNFKQEWESQLDELEAESAQLMEQLDGAGIELPSLYKWIESQAPNSCSTEAWKRRTQWVGSQVTSDFSEAVVDAEKYLDIHRPVRRKHGKILEEGASGFLGRKLAENDGSEGLNTNVDWTSFNKMCSDKSSLDNVSFGSKQWASVYLASTPQQAAELGLKFPGVDEVEEIDDIEDEPVDDFVVDDLSEEQKRKFRKVKEEDDVKIDKKLQRHLKQRHRNKRKLETMDQVSECYENGSSYQDLENQACGSSVFEVDKETCNGDSSIAEARGSKRQLVTDELDAENRKRQVITIDSDAEDLTADRSSPPCGVSDMGDQCNSQGYKTDILHSNSLPVRNDNENFRCTACDEVANEVHRHPLLDVIVCMDCKTLMVAKMKDVDCSECYCRWCGRSSDLLSSLEKAIESQGLAADDSSSDSDTDNSDAESNIHAGTKRKRKKKIRRILDDTELGEETKRKIAIEKERQERLKSLEAQFSSKARMMSFGGSSKKSYENGSVEMLGDAKTGYVVNVVRDEGEEAVRIPPSMSAKLKSHQVAGIRFMWENIIQSIKNVKSGDKGLGCILAHTMGLGKTFQVIAFLYAAMRCVDLGLRTALIVTPVSVLHNWRIEFMKWRPTELKPLRVFMLEDVPRERRAELLQKWRSKGGVFLIGYTAFRNLSLGKHIKDRQIARDICQSLQDGPDILVCDEAHIIKNTRADVTQALKQVKCQRRIALTGSPLQNNLMEYYCMVDFVREGFLGSSHEFRNRFQNPIENGQHTNSTAEDVKIMNQRSHILYEQLKGFVQRMDMNVVKKDLPPKIVFVISVKLSPLQRQLYKRFLDVHGFTKDKVTGEKIRKRSFFAGYQALAQLGNIAKARLCKFEFENSNNQISLLLSIYRSGKMNEKYIWNHPGILPLMKENRGTAKREDAVENFIVEDCSSDENAQTIICSEMFHIFAEKPNNNSELPPRKDANGFLHGDWWSDLLKENSYKEVDYSGKMVLLLEILTMCANFGDKVLVFSQNLSTLDLIELYLSKLPRPGKKGKLWKQGKDWFRLDGRTESSLRQKIVERFNEPLNRRVKCTLISTKAGSLGINLHAANRVIIVDGSWNPTYDLQAIYRVWRYGQTKPVYAYRLLAHGTMEEKIYKRQVTKEGLAARVVDRQQVHRTISREEMLHLFEFGDDEGADVIPDLGELNTTDHVGSFLRQKLPLPNGGVSSDKFMQTLIDRHHPSWIAHYHEHETLLQENEEEKLSKEEQEMAWEVYRRSLEWEEVQRVSPDEPTFERQQPSQNGSATDRKPAISNKPPPVRENRLTIKLAQIVETARNHAKVRKCTNLSHMLTLRSQGVKTGCTTVCGECAQEISWDKLKP

193 IbDExDH

>IbDExDH4

MPSVIVAEAMASEASKDLKKKGKKTPKPAAETLDSEKKKSRRSKVDLESDSEGSSAEKKRSKKKEKKRKAAEIDSGDEQRSETSSELVEPVNFKAEKKKKAKLMEAVGSYEEEEEEKEEDPNAVTNFRISKPLREALKAKGIEALFKIQAMTFDTILDGTDLVGRARTGQGKTLAFVLPILESLTNGPAKATRKTGYGRPPSVLVLLPTRELALQVFTDFEMYGGALGLTSCCLYGNTPYAQQENKLRRGVDIVVGTPGRVKDHIMRGNMDLTSLKFRVLDEVDEMLRIGFVEDVEFILGKVEDLKQVQTLLFSATLQDWVKHIASKFLKPDKKTVDIVGNEKMKASTSVRHIIIPCSISARSQLIPDVIRCYSSGGRTIIFTETKGYASELAGLLPGARALHGDIQQSQREVTLAGFRSGKFLTLVATNVAARGLDIDNVQLIIQCEPPRDVEAYIHRSGRTGRAGNTGVAVMLYDPKKSNISRIERESGVKFEHIAAPQPADIAKAIGEEAAVQIAGISDSVIPAFKAAAENLLNTSDLSPADLLAKALAKAAPSALLFRDILRSRARSLLTSMENCVTLQLESGKPVYSPSFVYNVLKRFLSEETAESIKGLTLTADGTGAVFDVSTEDVDTFIEGAKKAYDISLEVVKTLPPLQEREQSRGGGRFGGGSRFGGGGRGFSGGRGGRFSGGRGGFSDRRRGRW

>IbDExDH1

MADNQENSALFPIFILSVIGLPLVPYTVLKIFRAATRSTRNIHCECSVCTRSGKYRKSNSQRISSFLSCSNVTLILLWLIVGLVAYSVKQSRSEIQVFEPFSILGLEPGASDSAIKKAYRRLSIQYHPDKNPDPDANKYFVEYISKAYQALTDPISRENFEKYGHPDGRQGFQVGIALPEFLLAGASGGVLLIWILGGMILLPLIIGVIYLSRSSQYSGNVRRETLSTYFNSVKPSLAPSKIMEVFIKAAEYMEIPVSRSDDEPLQKLFSVVKGELNLDGKNAKQEQAKYWKQHPSLIKTELLIQAHLTRKAETLSPDLQRDYKHVLQLAPRLLEELFVMANLPRTSKGHGWLRPAIGVVELSQCIVQAIPLSARKATRGSSEGVAPFLQLPHFSDTVIEKIAQKARTFQDFRDMTSQERAELLAEVAGLSAAEAQDIEKVLELMPRVTVDVSCETEGEEGIQEGDIVTVQAWVMLKRANGLIGALPHNPYYPFPKEENFWFLLADANSNNVWLSQKVSFTDEAGAVTAASKIVEDKMEALGADAKEISAAVKEATERVKSGSRLVMGKIQAPAEGNYNLTSYLLCDSWLGCDNTVSVKVKVLRRNRTGTRVGRVAEGEQNIPDDIEDEDATDEDEYDEEEVESEYSEEDEEEEDDTQVTDKKRKEFDRQVTDKKAKKEVERQVTGKKGKEEVDRQITGKKGKANGTTRRKNR

>IbDExDH6

MALKNLLSSWGKSLKDFPEMSIPDESSTGLSENMLIAKELAYDKESLKTEHETLVTQLTDEQKNVYDSVMNEIDSNGGGLFFVYGYWRNARRSCGERLSSKNGAFQICYTTFFERNSTCNISQGSDLAELIIRSKLIIWDEAPMTHKYCFEALDKIMRDILRFAIPGSAEKTFGGKTVVLGGDFRQILPVIPKATKREVVGATINSSFLWTNCKVGAPVMLLRNIDHSLGLCNGTPLIITRLADHIIEARIVNGTHEGTKVLIPRTSLSPSDTRLPFKFQRKQFPLMLAYAMTINKKPMPNANSRWVLAVDEDEQDCVATSNVVYKEVFNNV

>IbDExDH5

MNRENSANEVDALILAEIPDPNADTEYHDTVSKFMLHGPCRELRKNSPCMVDEGVAMGAQSVRMESNLITVNKGNDNKVITEWMSSSTNALNGDVVDEINMYYDCRYISACEVTWWRLFGYLIHYRTPLVERLNFHLGNQQNVYYGEDQPLNKIVENNYKVKPPALGVILDDLDELDDFWSILDACRVNGSLGEAMKMGKHLLKSQRVVFSMVLVIWTNKGKSGAKKQEVEILAQLVHSSVFWPYLFVGMSKSSEFYVIGKLT

>IbDExDH3

MDAAEAPLSTSPSQSSFSQQRHFYHAVDRLKFKMETLVDLLGMAGRLPCLPMVVCCSTRDELDAVYSALSTLSYISISAVYSDLSEADRAQVFATFRQAVMRWNRQPMAQPQDAGNGEKEEKSHMIVVTDACLPLVNSGELPFNARVLINYELPTKKETYMRRMATSLAADGIVINMVVGGEVVALKNIDENSGFLIAEMPINSAPQCSGHVKKTDFGGDSMHARK

>IbDExDH2

MELDDWEPSAEELDSLERDAMRQIAERNSSSYAATTSVQHTPASPYRPERSSASVKNAQSPPRRILPSFGANKATGECPVENLPRQQQQQQKRSVKLFLHASGDIAAKFVYDQMHCEKYLKPVGMQKKGIPESFPIIFFMIVDVSSVIIVISRKNSKWNFLLKCGGHELYTLYSLLFVSALPVQNLDPLVQRAIVAANEVCDIQDLYESIPDSIKTKLMPFQRDGVRTNNIDPLRFALQHGARVLLADEMGLGKTIQAIAVSSCVRESWPVLVLTPSSLRLHWASMIQQWLDISPSDILVVLSQLSGSNRAGFKIVPSNAKRSIQLDGIFNIISYDTVPKLQDTLMASEFKVVIADESHFLKNAQAKRTNASLPILQKAKYAILLSGTPALSRPIELFKQLEALYPDVYKNVHEYGNRYCKGGIFGVYQGASNHEELHNLMKATVMIRRLKKDVLSELPVKRRQQVFLNLEEKEMKCINALFCELEIIKSKIKSSQSKDEAESLKLTMNNLINKIYVASAKGKIPSVLDYLGTVIEADCKFLIFAHHAEMIDAIHQYLLKKKVGCIRIDGGTPAASRQALVTDFQEKASIKAAVLSIKAGGVGLTLTAASTVIFAELCWTPGDLIQAEDRAHRIGQVSSVNIYYLLANDTVDDIIWDVVKNKLENLGQVSPLSFTKPILILIFLSLKVFHDGNSTSCFLTLRSHHDFQMLDGQEKSLEVLEDNSSKSPMKQKTLDSFMKRCNTSSEHEPMPKYSRH

>IbDExDH7

MSSNGDPIATIVESTFPMFQNGLCDNSFLESRTILAPTLDVVNAVNDYMNVPTPEFLNGLKASGIPNHSLTLKVGSPVMLLRNIDHSLNLYNNGTRLVVGRLSEHAIEAKIISSSLDEYDSVRSEVAI

>IbDExDH9

MSTLTQGELLPGVAGGRLEGRLVPNLADWAELAVDCVEFDRVNSPNSTEFAELTRRSLSLVRSLPAFLGSGKLQTEIDGIGGCPPPPRLTVATDSCSWLRAGALHLAGTKPVELRAGWMRRPRSRPIVASLGGLFGGIFKSTDSGESTRQLYASTLALINASDSQMSSLSDSQLRDMTATLQQRARSGESLDSLLPEAFSVVREASKRVLGLRPFDVQLIGGMVLHKGEIAEMRTGEGKTLVAILPAYLNALTGKGVHVVTVNDYLARRDCEWVGQVPRFLGLKVGLIQQNMTSEQRRENYLCDITYTVDELVLRRFNYCVIDEVDSILIDEARTPLIISGPAEKPSDRYYKAAKIAAAFEQDIHYTVDEKQKNVLLMEQGYADAEEILDVKDLYDPREQWASYLLNAIKAKELFLRDVNYIIRGKEVLIVDEFTGRVMQGRRWSDGLHQAVEAKEGLPIQNETVTLASISYQNFFLQFPKLCGMTGTAATESSEFESIYNLKVTIVPTNKPMIRKDESDVVFRATTGKWRAVVVEISRMHKIGRPVLVGTTSVEQSDALSEQLREAGIPHEVLNAKPENVEREAEIVAQSGRLGAVTIATNMAGRGTDIILGGNAEFMARLKLREMLMPRVVKPAEGVFVSVKKPPPKKTWTVKDSLFPCELSKESSKLAEEAVQLAVKTWGLRSLTELEAEERLSYSCEKGPVKDEVISNLRNSFLDIMGEYKYYTEEEKRKVISAGGLHVIGTERHESRRIDNQLRGRSGRQGDPGSSRFFLCLEDNIFRIFGGDRIQGLMRAFRVEDLPIESKMLTKALDEAQRKVENYFFDIRKQLFEYDEVLNSQRDRIYTERRMALEADDLQPLLIEYAELTMDDILEANVSPDSPRDTWDFEKLIAKVQQYCYLLSDLTPTLLADKCSNYEEMREYLRLRGREAYQQKRDIVEKQAPGLMKEAEKFLVLNNIDRLWKEHLQALKFVQQAVGLRGYAQKDPLIEYKLEGYNLFIEMMAQIRRNVIYSVYQFQPVLVKEQDQKRQNTENVNANGRGGNEKKSRDEVANNLTA

>IbDExDH21

MDCKHRRWDNRVVNNGSVCEIDIPPRFLLNCGHDPIATIVESTFPSARYGMIDELDLEGRAILSPTLDVVDQINQYMCNMNTAEGRTYLSSDSLCKAESDGENLSQVHTPEFLNSLRLSGLPNHSLILKVGAPVMLLRNIDHSLGLCNGTRLIVTRLTDHIVEAKIVNGTHQRTKVLIARISLTPSDTRLPFKFQRKQFPLMLAYAMTINKSQGQTLTHVGLLLKKSVFNHGQLYVAFSRVTHPDGLKVLALDEYGQTSFTTTNVVYKEVFNNV

>IbDExDH19

MLLLCYGSLSSFDLFHSKHAFTAYMLHRAMKFLPVNIDPIPEEIRQDFGLPSLYDAFSGIHQPKSLLDAELARKRLIFDEFFYLQLGRLFQMLQGLNTKLEKDALLNKCGKPELNTVDIDDWSFLSRKFLETLPYTLTSSQLVAISEIMWDLKRPVPMNRLLQGDVGCGKTVVAFLACMEVISSGYQAAFMVPTELLAVQHYEHLLKLLENLEEVVCKPSVALLTGSTPSKQSRLIRMGLQTGDISLVIGTHSLIAENVEFSALRIAVVDEQHRFGVVQRGLFNGKVGQENNVKQKDQNCLRNYVGKHHLVFKIVDNMIRVYSLQLFFNSTSSKKISTGQDDSFKGSVLMAPHVLAMSATPIPRSLALALYGDMSLTQGYDQTS

>IbDExDH22

MAAVASGSVVPANRRRVPMEEERMTFETSEGVEPISTFEQMGVKDDVLRGIYAYGFEKPSAIQQRAVLPILSGRDVIAQAQSGTGKTSMIALTVCQMVDTKSSEYELSSHLVIVNGVHACIGGKSVGEDIRKLEHGVHVVSGTPGRVCDMIKRRTLRTRAIKLLILDESDEMLSRGFKDQIYDVYRYLPPELQVVLISATLPNEILEMTSKFMTDPVRILGIKQFFVAVEKEEWKFDTLCDLYDTLTITQAVIFCNTKRKVDWLTSKMRENNFTVSSMHGDMPQKERDAIMGEFRSGTTRVLITTDVWARGLDVQQASSFSLLFKVSLVINYDLPNNRELYIHRIGRSGRFGRKGVAINFVKSDDIKILRDIEQYYSTQIDEMPMNVADLI

>IbDExDH10

MMHKHCFEALDRTMRDLLRFVNPYSGSKTFGGKTVVLGGDFHQILPVIPNGTRQDIVASTINSSYLWNNCKVLRLTKNLRLNSVEAEVDIRALEDFSNWIASIGDDKIGGLNDGYVELEIPSHMLLTSTGDHIATIVKSTFPMTYFSCDTVCKADSNNGILDDVHTPEFFNDIRALGVPNHSLTLKISSLIMLLRNIDHSLGLCNETRLVITKLADRVIEAKIMYGANEGTKVLIPHMSMTPSYPRLPFKFQRRQFPLMLSYAMTINKSQGQVLSHVRLLLKKPVAVHGQLYVAASRINNPDGMFQKEKRRLGVHQGLRERKRFSMVVDEDDEEGFEDDGIIKFDNLDLCDEEDEFDMED

>IbDExDH13

MGEQHEELHLEIVKDIKHALDEHNVTYNLPTVSEVPALIVGDIDPNMGSRDILVETKSGGLKRINELNPAYLPLQYPILFPYGEDGYREDIQFNEVRMQKSGGRCRVSQREFFAYRIHERFNEMSTILYARRLFQQFLVDAYTMVESSRLMYIRNNQKSLRCEAYKGLSDALTRGEVDTSKQGKRIILPSSFTCGARYMIQNYQDAMTICRHKGYPNLFITFTCNPKWPEIQRYMKKCNLNEDRPDIVCRVFKMKLDCLVKEIRSSTLFGTVPAVVYTIEFQKRGLPHAHIIVFLERLSTPFTAESKDKFISAEIPDKEVDMEYYKAIEEFMIHGPCGLHRPKSPCMCNQSRAIKYLFKYVNKGNDRVTAKFYKSTSDEQQNDTVDEIAMYYDCRYVSACEATWRLLSYDVQFRYPPVERLSIHLPECQSVVFEDDDRIDNVLNRPTVNQSMFTAWFEANKKFDSAEQLPYIDMPTKFVWKKDIREWHPRQGFSIGRIFYVPPGSGEIYYLRCLLNVVRGPTNWEDIKSYKGVIYPTFRDACYARSLLDDDKEYIDAIVEASQWPSAQSTLLQKTPLTSLDTDLKEMSPPRWRCRCQMLSFERVAVAAAGRCGGGGGGAVLRKKEGRAVLRKKEGHVSYNKSLKDYPHMPISNYDDAVRCDNRLLWDELDYDLNGVDHQKGGLYFVYGYGATGKTFVWRALSAKIRSRGDIVINVASSGIASLLLPGGRTTHSRFAIPIVVTEDSTCNITQGNNLAELIIQCKLIIWDEAPMMHKHCFEALDRTLRDLLRFTDSNAGNQTFGGKTVVLGGDFRQILPVIPKGTRQDIVSASIISSYLWDSCQVLRLTKNLRLSNKESTVDLKTVQEFASWLAAIGDGTMGGPNDGYSNVLIPDEMLLPANGNDIATIVESTFPLFKEGDCQLQYISNRAILAPTLDVLNAINEYIDLHVAEKKTYLSCDTVCKSIRQWYPHDMHTPEFLNGLKASGIPNHSLTLKVGSPVMLLRNIDHSMGLCNGTRLIITRLSDHVVEAKIVSGNNAAVEENAEMICSLRAAVIPEALPERKQPLLERKQLSVRGG

>IbDExDH14

MFSFTSLGAKVDKSINIGNCPPIFRINGQNYHLMGGLHRVGKSPKFAQLYIHDTENEVENRINAFRMDIVKYTDAMYIYQTRCKGVSISSGEYFAYRIHDRLSELSTMLSAPKHIPTSSVDAYRWRLSEQSKKRGLPHAHILIFLERLTEGFSANHMDEFISAEIPNKERDIEYFKAVGEFMMHGPCGQERPKSPCMVPKKFAEVSTLDHVGYPIYRRCDNGEPVERNGINKYLFKYVNKGNDKVTAEFYKSTNSENENAAVDEITMHPPVERLSFHLPDCQTVVFEDDDRIEHVLNRPPRFQDIRTVQGVVYQSFRDACYARGLLDDDREYIDAINRDRIIGLLAQDVQFNRRIVLKDMGLCLSNDEKKNLGLLELERLLQSYNKCLKDYPQMPSPNYDDALLTNNNLLFDELNYDRQAMRDECDKMERQLTDEQLVVYDTILRDIQVKHGGLFFVYGYGGDTVARLGISYTQALRLNLQPQNQINIQKCTYGTNPPRVSILG

>IbDExDH15

MPSLLKQLFSEKDEVSRQFQACVRTYNNTFAFTSLGIHNYDKKLTRRNKGIYTFKVQGQMYHFINDLVPGDQLPKNLQLYFFDTDHEVENRIKGADRMEASVVENLIDVLGRNPYSQFFRNLKDMSSLDDCNIVIRSNASLDQRVYNMPTSSQVAAIWVDEQDGSGQNNRNIRVLGKTGQSHSVKYYYGCYNPLQYPLLFPHGESGWHEGISKVPKASQNSSSICEGIIQPVGFSSIQALLHRENENFVGQSKRNTVSCREYYAYRFQIRPNDQSMLLHSGRLFQQFIVDTYIKIETQRLEYFRTQQRDVRTESMQGLVDSIACGETNASNVGRRVILPVSFIGGPRDMRKRYMNAMTLVQLYGKPDIFLTITCNPNWPEIKNSLEYCDESQNRPDLISRVFRAKLEELKVDLIKKLLELDVDAIVGAEIPDPVQNPSLHSLVAKHMIHGPCGSLNPQNPCMVENSGVSLCKSRYPKRFSDATVLGENSYPTYKRRDNSRLVTVAINLSSGEGVIDFDEIKEYQNARWVSPPEAAWRVYGFTLAEIKPCVVDLQIHLENYQYMRAPKSYEHLKMVGESMTTTFREAAEKLGLLQSDQVIDQCLQEAMCWQMPTSFRSIGPDLIFKWWLILDLNYCALHDCYISTYKSTDVTKSSVQKNTFDGSRKDINAAIDQICEFTGTKIIFWDLRDPFIDNLYKPTAAQSRLPVESSSTALCGGDPHSLPFESPLSECDSAPMSMDASSELPRLRPRRSLPGECSIAECGNGEIDGDSSESERKRVEWKKLGVK

>IbDExDH23

MADQKKKQQHLSLNQRHNRVLLDFSASHSKPPPSSRFHQEKEKIGAQGHSGDEKQNYDVPSFRFDFDSPSDSHHSLEEKEITAGKNLQDENEDIPNFGIGFDSPTASPPLEEPTEESESLEDNFAPQCFEVAALDTRPKSCFEEELTDHSKSGIEDTLTEDFSHISEAAHIDSNLPGEKEKVVKRKIKGRRRLCKISDDIDRKEKNLDANEGANGLEISDFYSPPQVKNAVESDYAGGGSEIRDILNDLSSRLEILSIDRKGEQSPLDIGSSNDLEIQSLGGRDCRIRSLTGRPNKNFAQEPRDCDDGDDFVSVEEHSFTLDGPNYSYKLPENVAKILYPHQCDGLKWLWSIHSQGKGGILGDDMGLGKTMQICGFLAGLFHSKLIKRAMIVAPKTLMPHWIKELSVVGLSEQIREYFGTSMKARDYELQYILQDKGVLLTTYDIVRNNVKSLCGDYYRYGDGSEDDIIWDYMILDEGHLIKNPSTQRAKSLHEIPCAHRIIISGTPIQNNLKELWALFNFCSPGLLGDNKWFKEKYEHYILRGNEKNATDREKRIGSAVAKELRERIQPYFFRRLKSEVFSEDASTSTKLSKKNEMCVWLKLTSCQRQLYVAFLKSEIVLSACDNSPLAALTILKKICDHPLLLTKRAAEEVLEGMESMSVPGQEDHAMAERLVMQMADAAEKFDIEENQNISCKITFIMQLLEFLIPNGHHVLIFSQTRRMLDQIQASLNCSGFKFLRIDGTTKAADRLKIVNDFQEGCGAPIFLLTSQVGGLGLTLTKADRVIVVDPAWNPSTDNQSVDRAYRIGQKKDVIVYRLMTCGTVEEKIYRKQVFKGGLFKTATEHREQVRYFSKGDLQELFSIPQQGFDVSLTQQQLDEEHDHHHIMEGSAKAEMEFLETLDIAGVSQHSLLFSKTAPVTADLDDEDVRSVRGAAAYVGNAPSRSSVEPAVIGGAQFAFNPKDVKSQAPAPKKSYNAVSHPTESEIRDRIKRLSYIFAEKGMRLPDRGEKLQKQIAELYAELDLVQIKERRMKSSTWMIYLGSLRGWLMCKYSLLSCKI

>IbDExDH18

MFLSATLQLRQTLNPFAQIRPHPQQEHAQIDFDECIFIPRRTRSNPSWIPSPPSPSSRSRKCRCPTSSALGKMPGKCIGVTKRNKRGVKPVADEASSWPEKFKKIGKLNGEEERVENENSIQDENVIGPKHNRLSSIQTPNQLFGVFLSSLVTPLIVLTEGPSRLSNNLIDKNRTPLQDIPVTLNMNQERDSSFQSTSSTITNAMRSSTMEFRNLNNDFNKAMEEQDLTNNESIVTYADFEDPCDSCQHCGALFWYEEHVGAQNSNGFTKYSLCCTNVNQGGGPPVYRLNGQNFHLIGSLQPVDGATPKFAQLYIHDTENEVNNRLTSIRNDIVSSSIEMEVVNDIKAALDENNVLVKSFRMARSEIQKNPRTEIRMRLIGKRSKDARTYNLPTVSEVAALIVGDLDPDMGIRDIIVELDGYTMVEAGRLLFIRNNQKALRCEVYKGLSDALFRGDIDPSTQGNHVVEGSILASPNAGTKQNEGEKQSLKQPLPDENDRFRSWMLAPRQARRRQALVDQMATSRTTVNFEEEEFLKRTRRTCINTIMTPIWTVDQPIKERTPKQKIGLPLNSSTPCVDQSEFVLIFSHENSNLSQRTNLLQITIAAPSPHSAYRRHRCASRRPPPPPSTASDSRPAAVTPSRRCSPLAAASPLLSSPPDATAAPPRRWTQLPHHLAAGRPPRSSPLLLAGVSVTREELRSLCTRQEDGQGATVLNQGIEINTVTGNIEGEAGAEVKEDMSVIATVDAPVG

>IbDExDH16

MDLLSSSFYVRLLLAVFHTLQNKMSMLYVLAKDISPQSYKKAIRLRLIRTYTVTEGRNKSAVKSQECVFHDIEGTYVHASIHADFIDQFSHLLKEGKVYAVKNFVAVSYYYQYKTTQHKYMMRFNQYTTIERHRRKGFPSLLFRIKPIEELLAGKVEEKLLIDVIGRVVEFYSPKDKVIAGFPIQLVDFLIEDSKIKCTLWDEHVPAVMPFFNNHVDGHLIVLLQLCRAKTVDSEVRISSSYTAKKFYSIWIAKSLQISEIGQYEICMKGLRKAIIMSQTNWAEGNVRYKVIVRVIDKTSDAPFVLWDKDCFELLGITGYDLKTKYSMAKMQVPIEFEQLRNKSMVFRINLKNEHIRNPAKPISVLSESESGDEAISPIVQANLKLKGVANDQDEVDTVFKRCLLDQVLIKSEFEEV

>IbDExDH8

MAGETINEVELMMPELRGPGGDMAAQMRLLWDLVKAPLIVPVLRLAVYVCLTMSMMLFVERLYMGIVIILVKIFCGKPEKRYKWEPMREDYEIGTSVFPSVLIQIPMFNEKEVYKISIGAVCNFAWPSDRLVVQVLDDSTDHNIKEMVEKECLRWASKGINITYQTRVTRGGYKAGALKEGLTHDYVQDCEYVAIFDADFRPEPDFLLRSIPFLIHNPEIALIQARCRFVNADECLLTRMQEMSLDYHFKVEQEVGSSTHAFFGFNGTGGIWRIAAINEAGGWKDRTTVEDMDLAVRAGLKGWKFLYLGDLHTEFGIISSKYSRSNPVKGGGMPGLLVQNSKHYTTKTYLVIYLLTQVFGAGDVKSELPSTFKAFRFQQHRWSCGPANLFRKMFIEIVRNKRVNVWKKVYVIYSFFLVRKITAHMVTFFFYCVVLPLTILVPEVEVPKWGAIYIPCIITILNSVGTPRANEWVVTEKLGDAINNNNKSNSKPAPKKSKSIFRDRSFQINLVYFEILEASAMANRTEAGEVVISSIRTGMKREFAMMMKAQAECGISIGLRALPVKRRKKEANVVTADQSLSHSNEKLDKTEELRSKDEGEKVEKLPLTSEWEEPKSDVVDGASEDEKNGTAVDSGIGEQETACVMEREELNSLGGSQKEKLINGDVAAKPVDVDNSGNGEENNGLFQKELLINGDVAEKPKEVDKSDNDKQSDSLVNCGGGVEKERELNGDVTEKMMDVDKTDNEEQLDSLMEPAMEELPMSGDCKTKSEAIKADCAYGSALATVPESVENSKVSTSSEKPLRRFTRSCLKPKQQVMSASPTEDTKAEDALESDEASAIGTTSKLEMKMSKKVALVKIPTKLKGLLATGLLEGLPVRYVRVTKARGRPEKGLQGVIQGSGILCFCQNCGGTKVVTPNQFEMHAGSSNKRPPEYIYLQNGKTLRDVLVACKDAPADALEAAIRNATGAGDARKSTFCLNCKASLPEASFGRPRLQCDSCMTSKKSQTTPSQVGDANCRDGQLEFIFLLNYYWADDLYKLGLPDLRGLQWSPSSNSVLKSTERMSSGTCPPSKVHGRLTRKDLRMHKLVFEGDVLPDGTALAYYVRGKKLLEGYKKGGAIFCYCCQSEVSPSQFEAHAGCASRRKPYSHIYTSNGVSLHELSIKLSMERRSSSDENDDLCSICADGGDLLCCDNCPRAFHTECVSLPNIPRGTWYCKYYENMFLKEKFDRSANAIAAGRVAGIDALEQITKRSIRIVDTLHAEVGVCVLCRSHDFSTSGFGPQTVIICDQELPKDKWFCCKECNSIHYALQKLVSDGEQSLPDSLMGIIKEKIKAKNLEDNSINDVKWRLLSGKNSSEETRVWLSGAVSIFHDSFDPIADSSTSRLDLIPTMVYGRNFKDQDFGGMLCAILMVNSLVVSAGVIRIFGKEVAELPLVATSLDCQGKGYFQSLFYSIENLLKSLGVKYLVLPAAEEAESIWTKKFGFQHITPEELKHYKDNYQLMIFQGTAMLQKQVSES

>IbDExDH11

MDKFISAEIPDKEVDMEYYKAIEEFMIHGPCGLHRPKSPCMVNNKCSKHFPKKFVNVSSWDDDGYPIYKRSESSRIVEKNGVQLDSRYVYVNKGNDRVTAEFYKSTSDEQQNDTVDEIAMYYDCASRAQATWRLLSFDVQFRYPPVERLSFHLPECQSVIFEDDDRIDNVLNRPTVNQRSGEIYYLRCLLNVVRGPTNWEDIKSYKGVIYPTFRDACYARGLLDDDKEYIDAIVEASQWSTAQSMRKLFVILLTSNLVNRPENVWNVVWEHLAEDVQFNKRKVLQQEDLCLSDDEKKNLSLIEIERLLQSYNKSLKDYPHMPIPNYDDVVRCDNRLLWDELDYDREALLKESQIMESQLTEEQKLVYETVVNDVDHQKGGLYFVYGYGGTAKTFVWRALSAKIRSRGDIVINVASSGIASLLLPGGRIAHSRFAIPIAVTEDSTCNITQDRTLRDLLRFTDSNAGTRTFGGKTVVLGGDFRQILPVIPKGTRQDIVSTSINSSYLWDSCQVLRLTKNLRLSNKESTVDLKTVQEFASWLAAIRDGTMGGPNDGYSNVLIPDEMLLPANGNDIATIVESTFPLFKEGGCQLQYISNRAILAPTLDVVNAINEYMTDLHVAESKTYLSCDTLCKSDAGNGILADMHTPEFLNGLKASGIPNHSLTLKVGSPVMLLRNIDHSMGLCNGTRLIITRLSDHVVEAKIVSGNNAGQIVLIPRMSMTPTDTRLPFKFQRRQFPLMLSYAMTINKSQGQTLTHVGLLLRKPVFVHGQLYAASRISNPKEAFSSSRTVWSEAFAILVAAF

>IbDExDH20

MLSDSDKDRRNTLLRYFSFYHPGAWLLLSVVLLQSLLLFSARSLPFSSIASYFQHSYPRHNASSTAAMTTFSAAPYASEVNATNDGVECPFGKVYIYDLPRVFNADLVENCGNWTRGTRAARTTEAESATAFYIPFYAGLAVDRTAGIISSRWGASHGISAGRKAHPWDYYDVAVPYPTGFHPTTAGDITRWQQFLRTRHRNTLFCFAGAPRRLIKNDFRALLLTQCNDSGACRAVDCGGTKCSNGTSEILETFLDSDFCLQPRGDSLTRRSTFDCMVAGSIPVFFWKRSAYYQYRWFFPPETESYSVFIHRDEVKNGTSIKSVLEKISKEKGELVKVVSQSDGEHGLSRGICIQRLVLEPTWSKLIRRIQNQTTEALDFEKKEKKSEEDKGKSEYDSERPSKKRKALEIDSIEEVRSATRPMNFKVDKKKKKTKFTEVVDNGVETEENPSSISNFRISDPLREALKVKGIEYLFPIQATTFDIILDGSDLVGRARTGQGKTLAFILPILESLRNGPATATRKTGYGRAPSVLVFADFEFYGGALGLTSCCLYGSSPYAPQQTKLKRGVDIVVGAPGRIKDLIQKGNIDLASLKFRVLDEVDEMLRIGFVEDVEFILGKVEDAGQVQTLLFSATLPDWVKHISAKFLKPDKRTIDIVGNEKMKASTSVRHIIIPCSTSARSQLIPDIIRCYSSGGRTIIFTETKDYASELAGSLPGARPLHGDIQQAQREVTLSGFRSGKFMTLVATNVAARGLDIDVMLVIQCEPPRDVEDYIHRSGRTGRAGNSGVAVMFYDPKKSNISKIEKESGVKFEHIAAPQPADIAKIVGKEAAEEIAGISDSVIPAFKAAAENLLQTSHLSAAELLAKALAKASGYTEMKRRSLLTSMENYVTLHLVSGRPVYSPSFVYNALKRFLHEAMAESIKGLTLTADGRGRVFDVSAEDVETFIAGARNEFDISLEVVKALPQLQERDKSRGGRFGGGRGGGGGGGGGFSDRRGGGGGRFSGGGGRGGRGGFSDRQNDRIGRGKGRSKWNTLARLLDNCLKMNELKRIHTHIITSPFLLTTDRYFLISRLIFFCCVSESGSLSYAANAFRLVSRPSLFMYNAMIRAHASKNTDPTSCQALVLYKRLLFDEFTPDFITLPFVLKECVNRVDAFAGRSVHGHAVKFGLHSDVFIQNSLISLYSGCGLLDNARRVFDEMPNRDLVSWNSMVVGCLRNGELDSALELFRNMKKRNIITWNSIITGFAQGGRGKEALGLFHEMQISGGDDVISPDKITIANVISACASLGAIDHGRWVHSFLKRSKIECDTVIATALVDMYGKCGCVDKALEVFTAMPKKDVLAWTSMISVFSLHGNANQAFELFLEMEAAAVKPNSVTFTALLSVCAHSGLVEKGRWFFNAMRSVYSLEPQLQHYACMVDLLGRAGLFDEAEVLIRSMPMEPDVFVWGALLGGCQMHRNFQLGEKVAQYLIALEPQNHAFYVNLCDIYAKAGKFDQVKKLRAFMNSKGIAKTAPGCSMIEIDGIVHEFSVRGSPQDLLQEIKPLLDALSCEMKRESHISCIQHFGKS

>IbDExDH25

MDRIEAAKELPFRVGFTGHSGHLTIEPLPPVERPKPLDSLPFVLPPAFPDETPETIQQYIKDKYLLPQLDADEFTPEKMGRQWEFDWFDRAKILPEPSLPRTIIVPKWEPPFRRPKKPTRWTMGARVCGDVSELTAGAEDLGALPQITGPPKDFVRGSVNNRPFRPGGLDGSSSLGRALPDGASSGKWVHEVLDGGYAQTIPPGFKKGLDLGDLEMHSSSWNVYEDQSAVNSTSNKKVNDFSVQFDELFKKAWEEDMTEYVEDALPAELECLHLLDLIKSGKICYIHIAWPFWQWGLMPLLWLGHSSELQAEVKEEFSVKLESLEIDTEVNKPEVAGEVTVTEKSVLDEILAAASGVSALRLDGERDGSGHEQTEAWAVTGDNKEVVERFNDLIPDMALTFPFQLDPFQRRGESVFVAAHTSAGKTVVAEYAFALASKHCTRAVYTAPIKTISNQKYRDFCGKFDVGLLTGDVSLRPEASCLIMTTEILRSMLYRGADIIRDIEWVIFDEVHYVNDVERGVVWEEVIIMLPRHINFVLLSATVPNTIEFADWIGRTKQKKIRVTGTTKRPVPLEHCLFYSGELYKVCENEQFVPQGFKAAKDVFKKKNTSSAIGGTGAFHGSSSAANDRSRGQRRDNFSHGKHPKGPQTSGNVGSSWGNKNSVGQNVMGLRRSEASLWLSLINKLSKKSLLPVVIFCFSKTRCDKSADNLPGTDLTTSSEKSEIRIFCDKAFSRLKGSDRNLPQIVRIQSLLRRGIGVHHAGLLPIVKEVVEMLFCRGVVKVLFSTETFAMGVNAPARTVAFDHLRKFDGKEFRQLLPGEYTQMAGRAGRRGLDKIGTVIVLCRDEIAEERDLKNVMVGSATKLESQFRLTYIMILHLLRVEELKVEDMLKRSFAEFHAQKKLPERQQLLMRKRAQPKKSVECIKGEPAIEEYFEMYSEAEMYSKQISESVMQSPVAQQYLIAGRVVVVRSQQGQDHLLGVVVKTPASNNKQYIVLVLTPDLPSTLQSQSGTGKPNDKKAPDFQILIPKSKRGLDDEYCPSVTSRKGSGIINIKLPHHGNAAGVNYELLGLKSEGNKYPPALDPVKELKLKDVDLVEAYYKWSNLLQKMAQNKCHGCVKLDENIKLAGEMKRHKEELERLEFQMSDEALQQMPDFLGRIDVLKEIGCIDADLVVQIKGRVACEMNSGEELICTECLFENQMDDLEPEEAVAIMSAFVFQQKESKAPSLTPKLAQARKRLYDTAIRLGELQAQFKLQIEPREYAEENLKFGLVEVVYEWAKGTPFADICELTDVPEGVIVRTIVRLDETCREFKNAAAIMGNSALYKKMETASNVIKRDIVFAASLYITGV

>IbDExDH30

MERLKGVKKTRIGRRSKQLERYEQKMKEERQKRIRERQKEFFSEVEVHRERLEDVFKMKRERWKGINRYVKEFHKRKERIHREKIDRIQREKINLLKINDVEGYLRMVQDAKSDRVKQLLKETEKYLQKLGSKLKEAKGNTGVVEEDEIAFDEEDETDQAKHYLESNEKYYMMAHSVKENIAGQPTCLVGGKLREYQMNGLRWLVSLYNNHLNGILADEMGLGKTVQALDISLKKRILSRALCTGGLNSGPFLVVVPSSVLPGWESEINFWAPSIHKIVYSGPPEERRRLFKERIVHQKFNVLLTTYEYLMNKHDRPKLSKIQWHYVIIDEGHRIKNASCKLNADLKHYRSNHRLLLTGTPLQNNLEELWALLNFLLPNIFNSSEDFSQWFNKPFEGNGDNSADEALLSEEENLLIINRLHQVLRPFVLRRLKHKATSRRRLGVRSVETERLMCSPRLTGEVAGICLTPGVEGVVAQSSSSSGVVLLFRAKPTPLCGNIMEQNRSKIRMFALVENQLPEKIERLIRCEASAYQKLLMKRVEENLGAIGTSKVRSVHNSVMELRNICNHPYLSQLHVEEVHEFIPKHYLPYVVRLCGKLEVLDRLLPKLKATDHRVLLFSTMTRLLDVMEDYLYWKQYKYLRLDGHTSGGERGALIEKFNDPNSPYFIFLLSIRAGGVGVNLQAADTVDLQAQARAHRIGQKKDVLVLRLETVQTVEEQVRAAAEHKLGVANQSITAGFFDNNTSAEDRREYLESLLRECKKEEAAPVLNDDALNYLIARSESEIDVFESVDKRRQEEEMVTWKKLCSEKGAENSESVPPLPSRLLTEEDLKSFYEAMKISDTPTVIPSTAVKRKGQYVGGLDTQHYGRGKRAREVRSYEEQWTEEEFEKMCLAESPESPVAKDEMSEKKLPTSSSSTMAIAEEQVLPSLQSSQQSEQTPVPIVQHSTEVTPPSKRGRGRPKRTVPVANVSPSPITLPATKESDEVITGSNIQNVSSCPVTLAPRPESMPGSSVEAKPPPGHPTSGRGRGRGQGRGRKSQIGEAPRRRGKKQNTIIQSVPVCPGNPDTITQTVNASSSSRTNDQDPVPHSTTEVETGKGQSSVSPAPPASVSKEPTIVSALPVVAPSSTSKELNSVSPIPLALASTGKDENSVSPAPIIASSPAASNSRTGSERAPITPSPSLLGSVSSDNSSTMGVVGKQDSGNQLALTSGPAAPMPITNTGLHASAPTPAPKQGRGRGRKPQTGGEAPRRRGKRQDLATNVGSPPPRSNQETEASLQTNLLNPVTSRITEDHGSKEVEEQRAVKSDPSQTHDAKVNDDMSTRKELHSETQVLERETDENLRGEENKSESRNDISGNVSKVASESCSNAVQSAEIKHDSVLMKDGVPQQIESQNSLRVELGSSKSGTETDNSKINVGKKAIQTASILESTAVVGKPAKEKCEGHSGNKDDEKEEQRLTTVVGHLASSQSNAEPATMHTHKTEVDTIAVVKKDVSSKPKSVETKLSKSRKIKKGSRTSFCVSANPSIEVNKVESLTETLVTASGSESIIPDDLDQKGDSEMPVGKTSATTGALGLKPESIVCSSLQNKSSASKTPGEVDPVLGCNLDMLDGDKSDTSKIESGNHSANNSIDSKPEVTVDENNPDKVDRKELDQEDPVLEVAPISKPKGTDNLDGMTSSILPSSEADVLVSVSPQTELVIQSSVQSDVGQGMGSLTNDSLAQVCKVPETNPLADSSGIEKEQTPTMQTNELQSERQTTVAKSLSDKSDNDHDVQAPEIKNGVSEDGANPETNESQPSPTLHTSESHSEMQVTVAGCPVENSERDHTAQAQEIENNVDMESQPCQNLQSSACQSRPQTSVAESPIDKSDKDHCAQAQETQNDVCEDAMTQERNVSQIGTDDKENISNADDALLQLPVVCESETKSAVESFEVSLNPCNIEKNTSLAVQASESLEPARIEPQCESDKDLVAEIEERKSSDVANSNQVMKENIERIPNETWNEFLMRGATTDNVTVEPPVESQNCQGSKMAEHEVISELSETQYNPASATSENEAEVHCSTENQSKINTDVILGDIKCVEPKSSSVDDHAVDEPESKKSDANKENQMKINTEVISGDIQGVEPKFSSVEGLAIDVPQSQISNANEGISKPEVNEGEHATAVDASNNDSEIECISDDLGTVTSKEATSIGVVGAGEKSEVDEDTSYQFPSESKCSAAPWASEEDSEAQQISENLEIVEVRVVTAAEKSETPCGEDSAVQVQENQRSDKTNDLSNSEISNPEQYIAAEASRTKQIAENVETESVRQATSVDSGFVAAAGNSENPCGVDVANEVQDNQISDGNKNVSKIEISEPSIAAGDSEIVFEMKQISESVATEAVREGTSVEVTAEEKSENSCQEDLAPKVQVEQDIDGNEHISDPEAVESSMSTVAEASEVKTEMKYTSEKLEAETAKETTSVDFAFIAAENSENQIGEDILVEVQVVNKNKDNLNPTEPEPCSATQASEVESKMKQILETVETETILDDTSVEDVTLAAAENSQMISENMGIETVKQSTSVDYAVVKEAGSFEDPCGEVLRNQVSDENKGTSNTGIAEPEPSTTFGASETHSEMKQIFEIVKTETVKEDTSVEVGALAAEENSEMISHNTEKEIAGEVSSVDDRALAGTEISENPSQKDFMVEVQENKKSDGKGHEPTTVASCNEVEIKQIPQNSEMVTAKAVTLLEAEAAEMAVETSVTVTFKETVSDISLATDLKGDISFEKPVDGSHGIHLIPCINDDPILESDVGDCSTAADEGLPSAVEGDDKDLSHQ

>IbDExDH32

MPIPNYDDVVRCQNRLLWDELDYEREELLKESQIMESKLTEEQKLVYETVLNDVDHQKGGLYFVYGYGGSGKTFVWRALSAKIRSRGDIVINVASSGIASLLLPGGRTAHSRFAIPIAVTEDSTCNITQGSNLAELIIQCKLIIWDEAPMMHKHCFEAVDRTQRDLMRFTDSDAGNRTFGGKTVVLGGDFRQILPVIPKGTRQDIVSASINSSYLWESCQVLRLTKNLRLSNKESTADLKTIQEFASWLAAIGDGTMGGPNDGYSNVLIPDEMLLPANGNDIATIIRCRVMVSSDMHTPEFLNGLKASGIPNHSLTLKVGSPVMLLRNIDHSMGLCNGTRLIITRLADHVVEAKIVSGNHEGQIVLIPRMSMTPTDTRLPFKFQRRQFPLMLSYAMTINKSQGQTLTHVGLLLRKPVFVHGQLYVAASRISNPKGLRLLIANEDTDSSHYTTNVVYHEVFNNL

>IbDExDH34

MKQETPVEALADENTGKGDGENCEHSFILTDDTGLVCRICGLVQKSIETIIDYPFAKEPKRQRTYPRQGARGSGNADENLPEAIHSYQELPVVEIYVHPRHRAIMKPHQVEGFNFLARNLVTENPGGCIMAHAPGSGKTFMIISFLQSFMAKYPEARPLVVLPRGILGTWKKEFQNWQIEEDFPLYDFYSVRADNRSQQLGVLRQWAEKRSIMFLGYKQFSSIMCDLCRNKASASCQEILLKCPSILILDEGHTSRNQDTDIMSALAKVKTPRKVVLSGTLYQNHVNEVFNILNLVRPQFLKLERSRAIMRRILSTASIPAGRRHNKQQSSDSFFFDIVEKTLLNDSDFKRKASLVCDLREMTKDVLQYYKGDSLDDLPGLMDFTVFLKLHPKQKRSVDKLKMEGSKFKISSEGSAICVHHGLKDLSRVDDQRIDNTLADLDIGEGVKAKFFLNLLNLCEAMVLARKSFRSPGFRLSNTESSMEQFNTSPQARVFFGSIKACGEGISLVGASRIVILDVHLNPSVTRQAIGRAFRPGQVKMVYVYRLVASGSPEEEDHSTCFKKESISKMWFEWNEYNGDPDFNMQPVDPQNCGDMLLEAKRINEDIVALYRR

>IbDExDH24

MNKATLHRSIRLLSKVDKSINIGNAPPIFRIHGQNFHLIGGLIPQEGNRPKFAQLYIHDTDNEVENRISAFSMGEQNAKLHLEIVKHIKHELDEHNVLVKSFRCAKGHITYQRVEVRSIVGDIDPNMGSRDILVETKAGGLKRINELNPAYLPLQYPILFPYGEDGYREDINFNELIQQRSGGHYRVSQREFFAYRIHERFNEMSTILYARRLFQQFLVDAYTMIYDPKLPDAMAICRHKGYPNLLITFTCNPKWRDSRYMKNAISTLEDRRHTVVFKMKSDLSQRNTFGHTIRYRDCRDNEPFTAASMDKFISAEIPDKEVDMEYYKAIEEFMIHGPCGLHRPKSPCMNRGRMGALDKRYVVPHNRVTAEFYGSTSDEQQNDTIDEIAMYYDCRYVSACEATWRLFSYDVQFRFPPVERLSFHLPECQSVVFEDDDRIDNVLNRPTKDIESRIQGKGVRGPTNWEDIKSYKGVIYPTFRDACYARGLLDDDKEYIDAIVEASYWSTAFSMRKLFVILLTSNLVNRPENVWDAVWQHLAEDVQFNKRKVLQQEDYPHMPIPNYDDVLRCQNRLLWDELDYDREKLLKESQIMESKLTEEQKLVCETVVNDVDHQKGGLYFVYGYGGTGKTFVWRALSAKIRPRVILSSTRCFKWNCISASCKLIIWDEAPMMHKHCFEAVDRTLRDIMRFTDSDAGNRTFGGKTVVLGGDFRQILPVIPKGTRQDIVSASINSSYLWESCQVLRLTKNLRLSSKESTANLKTIQEFASWLAAIGDGTMGGPNDGYSNVLIPDEMLLPANGNDIATIVESTFPSFKGGGCQLQYISNRAILAPTLDVVNEINEYMTNLHVAESKTYLSCDTVCRSDAGNGILADMHTPEFLNGLKASGIPNHSLTLKVDLAMLLRNIDHSMGLCNGTRLIITRLADHVVEAKIVSGNHEGQIVLIPRMSMTPTDTRLPFKFQRRQFPLMLSYAMTINKSQGQTLTHVGLLLRKPVFVHGQLYVAASRISNPKGLRFLIANEDTDSNEINPFKSRPTHIQRQFSQFFYLHAPLADEINSGVKFGSKDQGLSFTRGSVGATSQNEDLYVQLSYHYLHSPDTLLSRSIVTFEDRGGGSVHSKDKVVTTTATCRSEKK

>IbDExDH31

MFAHGFQGSATSDKVDAWKWKMTTLLRDNDNQEIVSREKKDRRDYEQIAALASGMGLYSHMYAKVVVVSKVPLPNYRFDLDDKRPQREVILPPGLPRRIDDHLKNYLSCKPSTAMFSRSSSNCSIATDEGLFELPEPLPHNTAAMENIIRRRSMQMHTEQQSWQESAEGRKMLEFRCSLPAYKEKDAILSAISQNQVVIISGETGCGKTTQIPQFILESEIESMRGAACSIICTQPRRISAMSVAERVAVERGEKLGETVGYKVRLEGVKGRDTHLLFCTTGILLRRLLVDRNLKGVTHVIVDEIHERGMNEDFLLIVLKDLLPRRPELRLILMSATLDAELFSSYFGGAPAVHIPGFTYPVQTHFLENILEMTGYRLTLYNQVDDYGQEKAWKVNKQAPRKRKSQLVSAVEDALGAADFKDYSPQTRESLSCWNPDCIGFNLIEYLLCHICENEGPGAVLVFMTGWDDISSLKEKLQCHPTLGDASRVLLLACHGSMASSEQRLIFDEPEGGVRKIVLATNIAETSITINDVVFVIDCGKAKETSYDALNNTPCLLPSWISKVSAQQRRGRAGRVRPGICYHLYPRCVFDALADYQLPEILRTPLQSLCLQIKSLKLGSISEFLSRALQSPELLAVQNAIEYLKIIGALDENENLTVLGRYLTMLPMEPKLGKMIILGAIFNCLDPVLTIVAGLSVRDPFLAPLDKKDLAEAAKKAQFSRDYSDHLALVRAYEGWRDAEKDLAGYEYCWKNFLSAQSMKAIDSLRREFYSLLKDAGLVDSNAAIYNAWSYDHCLLRAVICYGLYPGICSIVHNEKSFSLKTMEDGQVFLYSNSVNARDSKIPFPWLAFNEKIKVNSVFLRDSTAVSDSVLLLFGGSILKGDMLLYPRMDLHAYHELLSAIRLLLSGDQCSGKFVFNRQFLQQQSKPFVAASSPAAALISRTESGPGGDNSKSRLQTILSRAGYAAPVYETKQLGNNQFRATVEFNGTQIIGRPCNNKKQAEKDAAAEALDWLLGENRATNGNVDQISMFLKKSKTDHS

>IbDExDH33

MEHEADSWAVGDDGSEHNNNGDDSDHLDGIWKEMAFSMESSKETPVEALADENTGKGDGENCEHSFILTDDTGLVCRICGLIQKSIETIIDYPFAKEPKRQRTYPRQGARGSGNADENLPEAIHSYQELPVVEIYVHPRHRAIMKPHQVEGFNFLARNLVTENPGGCIMAHAPGSGKTFMIISFLQSFMAKYPEARPLVVLPRGILGTWKKEFQNWQIEEDFPLYDFYSVRADNRSQQLGVLRQWAEKRSIMFLGYKQFSSIVCDLCRNKASASCQEILLKCPSILILDEGHTSRNQDTDIMSALAKVKTPRKVVLSGTLYQNHVNEVFNILNLLEEGTISSKVQIQFFFDIVEKTLLNDSDFKRKASLVCDLREMTKDVLQYYKGDSLDDLPGLMDFTVFLKLHPKQKRSVDKLKMEGSKFKISSEGSAICVHHGLKDLSRVDDQRIDNTLADLDIGEGVKAKFFLNLLNLCEAMGEKLLVFSQYLLPMKFLERLTINAKRYSTGKEIFSITGDSDSATRESSMEQFNTSPKARVFFGSIKACGEGISLVGASRIVILDVHLNPSVTRQAIGRAFRPGQVKMVYVYRLVASGSPEEEDHSTCFKKESISKMWFEWNEYNGDPDFNMQPVDPQNCGDMLLEAKRINEDIVALYRR

>IbDExDH28

MFCFTSMGGRIDNNVNKGGRPLIFRLNGQNYHLMGSLLPEEGSAPHFAQLYIYDTANELQNRINAVRGCGEQTDIHVEIVKVIKDELDKHNVLVKSFRIAKIEIERNPRVEYEERLRSLDSIISGEIPDKSSDPAYFNAVEEFMIHGPCGASRKSSPCMVNGSCSKHFPKKFVNSSTFDEDGYPIYTRRDNGRTIMKNGITLDNRYVVAHNRHLLLKYKAHINVEWCNQSRSIKYLFKYVNKGHDRVTAKFYKTSNEEESTKVIDEINMFYDCRYISPCEASWRLFSFDIQLRSPSVERLRFHLPNQQSVIFEEDDVTSTGTRTTEGYPCRNPIKSRKSPL

>IbDExDH27

MAASRTGGRRTAVARGEGEKRREAKVRGSYNNFSSSLSIGAMFWMEERQNKCVTRGTPKFSICCNNGKITLPSMLMPPKGIFDLFFGSTNKSKQFLNNIGTYNNMFCFTSMGGRIDKTVNLGKSPHFAHLYIYDTHNEITNRLNAVRPDSENKDIDSEIVKDIKNDLDKHNVLVKSFRMARDEYEKNPCAEVKIKLLGKRNKDARTYNLPQVSEVAALVVGDLDTNMGPRDIMVETKAGTLQRISELNPSYLPLQYPLLFPYGEDGYSEDIPFSIIKWEVDPNAQGRRIILPSSFVGGARYMIQNYQDAMTICKWIGYPNLFITFTCNPKWPEIERFLKEKNCRAEDRPDIICRVFKMKLDGLIADCRKNKLFGSVNGVIYTIEFQKRGLPHAHILLFLEKKREGTITSNLDAIISAEIPHKEMDTEYYTAHFPKKFVETSTLDDDGYPIYKRRDTGRIVVKNGIPLDNRYVVPHNRELLMRYMAHINVEWCNQSRSIKYLFKYINKGHDRVTVEFCKSNTDKDNGKVVDEINMYYDCRYISPCEAAYDNPVENVVNRPTIAHNLCGRKILGNGVQGKEDLQLVGFFIFLLFGSFKDACYARGLIDDDKEYVDAIIEARRSIPRANKFEYARRRLCYDRDAQKVENEVLVSQLTGEQRTIYNEVIQNVDNNTRGLFFVYGYGGTGKTFLWRALSSYLRAKGDIVLNVASSGIASLLLRRVRTTHSRFAIPISINEDSTCNISQGSPLAELIVYCKLIIWDEATMMHKYCFEALDKTMRDLGEKQLFLVEISGKSYQLFQRLECTDEEDDLIIPNHILLQYVDNPIAAIVESTFPNYETACTESQFLLSRAILAPTLDVVDNINDYMNEMNKGDSKTYLSCETVCKADDSGDTLGDLHTPEFPNGLRYVLEYQTIL

>IbDExDH26

MRFKTCEHKYILKANNDTVISFSDQDDSIFPKNMFQFKDFGSLKAPGGVDCNQLIDLIGRVVFIHQPKDIQVKQNSQRLIDFVIEDCRVRVTDDGEIRISSSDTATRVLFNYKCPELFPFKDRQIGYPHLFITFTCNPKWPEIEWYVAHCGLKADDRPDIICRVFKMKLDAVIEDIKTQKLFGDICGDQTLDEIVKNQIVSERFTAWFEANEIMKMRSLTYVEFPAKVVWDVVWEFLAEDAQFHHRRHMDNPIGEIVIFVGKSLRDFPEMPLPDETNIGFMENMLIAKELAYDKESLKTKHETLVTQLSDEQKNVYYSVMNDIDCNGGGLFFVYGYGGTSKTFVWRTLSSMIRSRGDIVLNVASSGIASLLLPGGRTLQSIFAIPLSLNEDSTCNISQDSDLAELIIRRKLIIWDEAPMTHKHCFEALDKTMRDLLRFAIPGSVEKTFGGKTVVLGGDFRQILPVIPKATRPIVVEATINSSYLWTNCKVLRLTKNLRLRSLASEEDRFLLNCGHDPIATIVESTFPIAGYGMIDELHLEGRAILSPTLDVVDQINQYMCNMNTAEGRTYLSCDSLCKAESDGENLSQVHTPEFLNSLRLSGLPNHSLILKVGAPVMLLRNIDDSLGLCNGTRLIVTRLTDHIVEAKIVNGTHQGTKVLIARMSLTPSDTRLPFKFERKQFPLMLAYAMTINKKGVKTCNGIYRHALREEQAWQLDSYTFTSYSNHYMPITFDGDGATTWIWNLYSWSSPRGRLPGSDDGKDGRWRQGEWGVSVWRRIRRWENERPSEIVETRVVCPERLGWLAVGGGRWPRSRGYMAACGTVWKTRDKLRQRGAGNLKLRISGLKGNCRCAEQRSFHPFNTTAVLRITT

>IbDExDH29

MPPMYILATDISPMRTTCALKVRCIRTYEVKERRSTEVVKCRECVFHDSEGNVLHAHIPREHVVKFQNQHMFRLRSFKSLKESEKIDDKELIDVIGRIVEIYSPIEKIVGGRPSRLIDFLIEDESGDEMKCTVWDDHVDKIDSCMSAANTFDTFNTGSMIMTTISEYGDYWVVAKIIGIEGALDWCYNSCKTQGCSKKLTLNTKGLYDCYKCNKTWAEGILRYRIKVRVIDRNGNAPFLLWDRECKELIGMSAVELRDKYPQDNQLLPNEILSLCSMALIWKIAAVRKEQFDNLHNAFAVMKTSDNRTDKGTTCNTNNKQLTGTPKNRFNVTLGSKNSLPLSDITNCNGTIRSKKIKGSLTLTGVSTIENNVNAHKRSERSQSYSLGISRPNITQESDPQVVICRDLSQDFDEVMESEVQSVQYDDCGDPSYSCEHCSATFWYNERLSQSRLKSLPKYSVCCAHGKINLPQMSTPPKQLYDLFFEHGEKMTCFLKNIRKNNDKDSIHVEVVLSLILKKFWDENNVLVQSFRNAKTHIQANPRVEIKMRLIGKRNKDARTYNLPTASEVAALIVGDLEPTMGQRDILGETRSGLLKRISELHPSYFPLQYPLLFPYGEDGYREDIQRCKDSNNKSNSRVRITAREFFSLRMHERPGELSTLLFSKRLFQQFLVDAYTMVETGRLILALTLGYREIPACELIYIRNNQKSLRCEAYKGLSDALTRGEIDPSTQGKRIILPSSFTGGARYMVQNYQDAMARCHWIGYPNLFITFTCNPKWPEIERYLGKRDLKAEDRPDIVCQIFKMKLDDLIKDLRTGELFGTIRAVIYTIEFQKRGLRHAHILLFLANNDRNAGCNFIDKMIAAEILDKEVDQEYYKCVEEFMVHGPCENVRKQSPCMVNGRCSKHFPKKFVDSSTFDEDGYPIYRRRDDGRFVMKSGIQLDNRFVVPHNRYLLLKYRAHMNVEWCNQSRSIKYLFKYVNKGNDMVTTEFYKSTSDSNGNEVIDEINMYYDCRYISACEATWLFSFEVQFRTPAVERLSFHLPDCQTIIFEDDDPVDNVLMRETIGQSMFNGWFEANKRFPEAKLLTYIEMPTKFVWKKDIREWSPRKKGFVIGCIFYVPPGTGELYYLRCLLNIVRGPTRFEEIWFFNGIQYTSFRDACYARGLLNDDKEYIDAIKEASDWSSAHSMRKLFVTLLTSNSFNRPEIVWKEVLDYLSEDVQYNQRIFLSIPDLVLTNEEKENLTLIELEKLLQVYNKSLKDFPPMPTPRAASTRLNGNRLLFEELSYDRAVLADESDRFVSQLTEEQRGVYDTIIGDVSSNKGEIVINVASSGIASLLLPGGRTAHSRFYQLYRRALGASNEDCPEVVIPNEMLLSSNGDPIATIVESTFPMFRNGSCDNSFLESRAILAPTLDVVKAVNDYMSSMHEAESRTYLSCDSVCRTDNGSRVLVDVHTPEFLNGLKASGIPNHSLTLKVGSPVMLLRNIDHSLGLCNGTRLVVTRLSEHVIEAKIMSGSHSSTRVLVPRLSMTPSDPRLPFKFSRRQFPLMLSYAMTINKSQGQTLSHVGLLLKKPVFVHGQLYVAASRVSNPTGLKILLCSELESCCKKTTNVVYKEVFNNL

>IbDExDH38

MLQAPSFNHLPSLQRYAATLNRSYISRFKLFRASIAELLFVDSFLLTVKAHCPRSQAPASSSIFSSSQPLLHRPPSSVHHSSGTPISNFSTYSNLTVVLLWIVMGVLVYYIKNISSEIEIFEPFSILGLESGASDSEIKKAYRRLSIQYHPDKNPDPEAHTYFVDYISKAYQALTDPISLENFEKYGHPDGRQGLQMGIALPQFLLNIDGASGGILLLGIVGVCIILPLTIAVIYLSRSAKYTGNYVMHSTLAAYYHFMKPSLAPSKVMDVFIKASEYMEIPVRRIDEEPLQKLFVLVRSELNLDLKNIRQEQAKFWKQHPALVKTELLIQAQLTRETAALSPNLQRDFRRVLELAPRLLEELMKDCGKILICSSFPIISFNASKKLVSILESNVYLELMEFCFRDFFPLAPTLISSFTLNEMAIIPRTPLGHGWLRPAIGVVELSQSIIQAVPLGARKATGGSSEGYASFLQLPHFSEAVIKKIARKKVRTFQDFRDLDAEERMELLTQVAGFSKAEYRDVEMVLEMIPSISIDISCETEGEEGIQEGDIVTMHAWVTLKRGNGLIRALPHSPYFPFDKEENFWLLLADSFSNDVWLSQKVSFMDEATAITAASKAIQESKEGSGASAREINGAVREAIEKVKNGSRLVMGKFQAPPEGNYNLSSFCLCDSWIGVDAKSNLKLKVLKRSRAGTRGGVTADETPAPEEGIEEEEEDDDGYDDYESEYSEDEEDVKDPKGGKEVVANGGTTQKGSSSSSDDDDSETEAD

>IbDExDH36

MEEATEQKKKTEKNRVPVLPWMRNPVDISAIDECPLNNLPFLDPRLEAALKNTGITSLFPVQVAVWQETIGPGSFERDLCINSPTGSGKTLSYALPIVQMLSNRTVKCLRALVVLPTRDLALQVKEVFETLAPAVNLRVGLAVGQSSIADEISQLIKKPKLESGMCYDPEDFSRELQSAVDILVATPGRLMDHINNTKGFTLEHLCYLVVDETDRLLREAYQSWLPTVLQLTRPCDMLFPSDSFSPSAFSSLKTIRRFGVERGFKGKSCPRLVKMVLSATLTQDPGKLAQLDLHHPLFLTTGEMRYKLPEQLKSFKVICESKLKPLYLIALLQALKGEKSIVFTSKTLRAFRDGEVQVLISSDAMTRGMDVEGVKKFKKMLQKVDNNSCTAHPIESQAVESLRPIYKSALEKLKESVESEKFKKRHTSLKASNASKRKGEQRLE

>IbDExDH41

MKQGCGICKNLDTLIMRNLDEELSPTPSVQSSLPNTVRMLIPFKNSEVSGEELKNSKRKTKLMWSPVALEAQSFPEAIAEFKRVSLSDRRPALDLHVKVRKHLLYLGWIVESVYDRNMTRFRYLSPSGTVLMSLSQICKELETMSKTVSDSSTSCSSIVKQESGRELYDPPSAEPIIDSVYCPQAVFEYVRVVAPKRLSKKRRPGVAILADKAKKHLVFSDWKIYSQRKGDRRELRYMSPAGKVFYSLITACECYIKENALSLSDAHLTGQRQIRQVDLEDEDHVCNEQLLLESGEKLPTIKSHKKRKLNLYTSRAFIRSGDVADSHSSTRVLRSSKRARKIASSSHQTPRSILSWLIDNNVILPRQNCCQKVYGLSTFAAHAGSTTHCSSENIILEDGRSILDCQSEMKWKISARKTELHIKKRRHNMSKNDYICSICHDGGELILCDRCPSAFHTGCLGLKELPDGDWFCPLCCCRICSQSRFDKNKDQFADNNALYCSQCEHQYHVACIRNNGLLKHPEGYWFCNNRCEQIFVDLHKLLGKPIQLGTDNLTWTLLKYKKPDSSHPDVVDDEHALENYSKLNVAIDVMHECFEPVKESRTGSDLMEDIIFSRWSELNRLNFQGFYTVILERNEELITVATLRYSMMLSFLNLAALFYNPSRFPLTNLNYLNCHLKGSWRQGITPLPPFFQVLLRASRASGISCSLGFCLYTLTPKLSELGVERLVLPAATSVLNTWISSFGFSVMEESTRLDLSGFNILNFHGTVMCEKFLARSSPSMELIVSTAAAEPNQHIFGAVANKGNNAELDGTTSAVSEVLQAEQIDGSDIIDKGPAETPGGTGTNDHPAPLVLVLKETPAPRLPVQNTLDCLREKAPDQREAGNNVVGMFKCYERRRRYDAVEANLFTKAVTDG

>IbDExDH40

MPALNHSNIYSKTTVLRRLAQKRDQENASSLHLSNNHSQKHGNGSQFQNSYVYGVFENSINSPLSIITHSGQLIKSSISCNKNQSQSVISNEAGTTSVQRLQVRAPLNDVTNVRLLKYNVLTKVGKNEVICPNDLQKAIRLRATESTPNLQTLSNGSTAEADTNLPIRRSQRLTFQKGKAVVNSQQLIIDNIQNDNVVSVSALTVAYHDIGDPSNICQNCNAIFWFEERMNKAARCGTAKYSGCCGHGKIVLPKMMRPPQKLFDLFFSQGDKQKEFLRYIRRYNNMFAFTSLGAKVDKSINIGNAPPIFRIHGQNFHLIGGLIPQEGNRPKFAQLYIHDTDNEVENRISAFSMGEQNAKLHLEIVKDIKHELDEHNVLVKSFRCAKGNIDSNPGVDFKMRLIGKRNSDARTYNLPTVSEVAALIVGDIDPNMGSRDILVENKAGGLKRISELNLAPTTSKRGLPHAHIIVFLERQSEPFTAASMDKFISAEIPDKEVDMEYYKAIEEFMIHGPCGLHRPKSPCMVNNKCSKHFPKKFVNVSSWDDDGYPIYKRTESSRTCGKNGVRLDSRYVVPHNRYLLLKYRAHINVEWCNQSRAIKYLFKPENVWDAVWQHLPEDVQFNKRKVLQQEDLCLSDDEKKNLALIEIERLLQSYNKSLKDYPHMPIPNYGDVLRCQNRLLWDELDYDREELLKESQIMESKLTEEQKLVYETVVNDVDHQKGGLYFVYGYGGTGKTFVWRALIAKIREVILSSTLLQVELHLCCFQVVVQHTRDSQYQLQSQRTRPAISHKAVDRTLRDIMRFTDSDAGNRTFGGKTVVLGGDFRQILPVIPKGTRQDIVSSINSSYLWESCQVLRLTKNLRLSSKESTDLNTIQEFASWLAAIGDGTMGGPNDGYSNVLIPDEMLLPANGNDIATIVESTFPSFKGGGCQLQYISNRAILAPTLDVVNEINEYMTNLHVAESKTYLDSRLVCRSDVGQWYPHMHTRSSSMA

>IbDExDH43

MFWMEERQNKCVTRGTPKFSICCNNGKITLPSMLVPPKGILDLFFGSTNKSKQFLNNIRTYNNMFCFTSMGGRIDNNVNSGGAPPVFRMKGQNFHLMGSLLPMEGKSPHFAQLYIYDTQHEINNRVNAVRRWYGRDEFEKNPCRGGQNKVTWKRNKDARTYNLPQVSEVAALVVGDLDTNMGQRDIMVETKAGTLQRISELNPSYLPLQYVAFPLAEDRPDIICRVFKMKLDALIADCRKNKLFGSVNGVIYTIEFQKRGLPHAHILLFLEKKREEEFMMHGPCGKARMNSPCMRRDTGRIVVRNGIPLDNRYVVPHNRELLLKYRAHINVEWCNQSRSIKYLFKYINKGHDRVTAEFCKSNTDKDNGKVVDEINMYYDCRYISPCEAAWRLFGFDIQLRAPPVERLSFHLPGQQSVIFADDDPVENVVNRPTIAHSMFMEWFEANRTYPEARELTYAEMPTRFVWKKDIRKWSPRKRGFAIGRVFYVPPGTGDIYYLRCLLNKVRGPMSFLDLMKVDGVQFGSFKDACYARGLIDDDKEYVDAIIEASDWATGIHLRRLFVTLLMSNSIARPDAVWDVVWHLLSEDAQFHTRRNLNMPRMLSPLGKSLKDFPNMPLASEECYNLASNKFIQEEWCYDRDAQKVENEVLVSQLTDEQRTIYNEVIQNVDNNTGGLFFVYGYGGTGKTFLWRALSSYLRANGDIVLNVASSGIASLLLPGGRTAHSRFAIPISINEDSTCNISQGSPLAELIVYCKLIIWDEAPMMHKYCFEALDKTMRDLLRFKNENSYDIPFGGKTVVLGGDFRQILPVIPKGTRQDIQFKHLMNDKRTEQFAKWIADIGDGVSSTCTDEEDDLIIPDHILLQYEENPIAAIVESTFPNYEIACTDSEFLLSRAILAPTLDVVDSINDFMNEMNKGDSKTYLSCDTVCKADDPGDTLGDLHTEFLNGLRLIVTRLADHVLEAKIMSGTHKGMKVLIPRMSLTPSDTRLPFKFQRKQFPLMLSYAMTINKSQGQTLSHVGLF

>IbDExDH37

MLIVEELSYDKESLKTEHETLVTQLTDEQKNVYDSVMNDIDSNRGGLFLVYGYGRTGKTFVWRTLSSKIRSRGDIVLNVTSSGIASLLLPGGRTAHSRFAIPLSLNKDSTCNISQGSDLAELIIRSKLIIWDEAPMTHKYCFEALDKTMRDILRFAIPGSVEKTFGGKTVVLDDDFRQIPPLFKSNKTEVVEQLLILHTVDKLQDIGDGIAGVVNNGSSYIDIPSRFLLKCGHDHSNYKGRTYLSCDSLCKAESSDENLSEFGARFSGSGGRACVLGVNAGSHRGFVVGGGESRLNLDDRQRREKVLFVCFRGTLGTGGGAFVWFVATGEIQGFNYDDLVREAENRWLKPAEVLSILQNHENLKITHVPPEKPPSGSLFLFNKRVLRFFRKDGHRWRRKKDGRAGGEAHERLKVGNVEALNCYYAHGEQNPKFQRRSYWMLDPAYEHIVFVHYRDISQGRQKALSMPQLSPVSFTINQSPSPYSVQHTIYTAARGGYFEAYSSSGSTEISSFAATKSNGTNHSSGTVEEDSSPDAHEISEALRRIEEELYMNDGIAPVYNTIENSAHADNIRDDHHNSINQIPEDSNCDLLQRRSGDLTEYRDQFLGHEVNLWNMGNGYGSSVDGKNILLARDAVETPESVSWQLNFNGDFRYPTFWPEINTDGSNPDQSPTLFDQDQNGISIAQKQKFRIADLSPNWGYADEATKVIIIGSFLCDPSESEWMCMFGDIEVPVQIIQEGVLCCHAPPHLPDKVTLCISMGNQEPCSEGREFEYRVKSTGTAESCLPETQCTSKNTEELLLLVRFAKMLLSDGSRCSTDSPESGTKFLEKVKASEELWGEMIDTLLVGNSTSSLTVDWLLQELLKDKLQQWLSSKLQGQTNLSDSDCVLSRKEQGVIHMIAGLGFEWGLQPILDAGVTVNFRDINGWTALHWAARFGREKMVAALIASGASAGAVTDATKQDPIGQSPADIAAVCGHKGLAGATTNEEDQLSLNDTLAAARNATEAAARIQAAFRAHSFRKRQQRSEAAAAAAAATGDEYSILLNDIQGLSAASKLAFRNSRDYNSAALVIQKKYRGWKGRKDFLAIRQKVVKIQAHVRGYQVRKEYRVCWATGILEKVVLRWRRRGVGLRGFHLEIDESDGEAILRVFRKQKVDAAIDEAVSRVLSMVESQEARNQYRRILEKYRQAKAKLQSAENDARTSHDSMSNMENDDLYSSFY

>IbDExDH44

MPALSATRAFLHFGDSLSLRNLSGLTRIPPLNGRVRFLSDFGPLTLASLGLKNDVETISRNAKNKLQEGVSTIEVPKNNTKKKVNQKRNNGVKETRSIDIGAAPFAAKSFSELGLPSLLIERLEKEGFTVPTDVQAAAIPTILKNHDVVIHSGQGQNRTDIEAVIVAPSRELGMQIVRETEKLLGPDRRLVQQLVGGANRSRQEEALKKNKPAIVVGTPGRIAEISAAGKLHTHGCRYLVLDEVDELLAFNFREDMQRILDHVGRRSGAGLRASSSSVKRAERQTILVSATVPFSVIRAARSWGCDPLLVQANMVTPLQSLPPGSVDFSGTPTSASLNSNSQAQPAVQSLPPNLNHYYCVTRIQHKVEVLRRCIHALDAKSVIAFMNHTKQLKDAVYKLEARGMKAAELHGDLSKLARSTILKDFRNGEVRVLLTSELSARGLDVPECDLVVNLELPTDSIHYAHRAGRTGRLGRKGNVVTICEEAEVFVVRKLQKQLSIPIQSCEFAEGKLVITKDE

>IbDExDH45

MPALSATRAFLHFGDSLSLRNLSGLTRIPPLNGRVRNAKNKLQEGVSTIEVPKNNTKKKVNQKRNNGVKETRSIDIGAAPFAAKSFSELGLPSLLIERLEKEGFTVPTDVQAAAIPTILKNHDVVIQSYTGSGKTLAYLLPILSHVFPLNGVNSSSGQGQNRTDIEAVIVAPSRELGMQIVRETEKLLGPDRRLVQQLVGGANRSRQEEALKKNKPAIVVGTPGRIAEISAAGKLHTHGCRYLVLDEVDELLAFNFREDMQRILDHVGRRSGAGLRASSSSVKRAERQTILVSATVPFSVIRAARSWGCDPLLVQANMVTPLQSLPPGSVDFSGTPTSASLNSNSQAQPAVQSLPPNLNHYYCVTRIQHKVEVLRRCIHALDAKSVIAFMNHTKQLKDAVYKLEARGMKAAELHGDLSKLARSTILKDFRNGEVRVLLTSELSARGLDVPECDLVVNLELPTDSIHYAHRAGRTGRLGRKGNVVTICEEAEVFVVRKLQKQLSIPIQSCEFAEGKLVITKDE

>IbDExDH42

MARIRSNLTQLVEVKMNLIGRRSKDGRTYNLPTANEVDALIVGDLDPSMGDLDILIRADGLKRINQLNPAYLPLQYPLLFPYGEDGYREDISFSDAWHQRHHGGRKRISPKEANSEAEIDAIISAEIPDPDDDTEYHDVVGEFMLHGPCGQLRKNSPCMINACEATWRLFGYAIHYRTPPVERLNFHLEHQQNVVYGEDQTLDEIVENQTLSRANSRLEYIDGITDSSHWASASALRRLFATLLSSSTISRPEVVWDAVWEFLAEDAQFHHRRLMNNPDSDKKQFALVELEKLLSLWGKSLRDFPEMPLPDETNIGFNENMLIAEELAYDKESLKIEHETLVTQLTDEQKNVYDSVMNDIDCNGGGLFFVYGYGGTCKTFGSARKTFGGKTIVLGGDFRQILPVIPKATRPIVVGATINSSYLWTNCKVLRLTKNLRLRSLASEEDKLTADWFSKWIAKHRRWDNGVVNNGLSEIDIPPRFLLNCGHDPIATIVESTFPSARYGMIDELDLQGRAILSPTLDVVDQINQYMCNMNTAEGRTYLSCDSLCKAESDGENLSQVHTPKFLNSLRLSGLPNHSLILKVGAPVMLLRNIDHSLGLCNGTRLIVTRLTDHIVEAK

>IbDExDH39

MASLAERLHVRWERRPVYNLDDSDDETDIRRGKSGPSQETIERFVRHDAKVDSCQACGGDENLLNCEACTYVYHPKCLLQPLEATFPSSFRCPESVSPLNDIDKILDCETQPTIGDDSDASKLGSKQIFVKQYGVKWKGFSYHRCACVPEKEFVKAYKTLPCLKTEVNNFHRQMSCILLVLGLFFCFIPLSCCDPGRSCPKTWNSSLLNYTYQFSMAQHQLRGVLNLVDGCSFRVSQFDMLEGSDVRWWGAVGEEFENLTKGFVISDQILNKTYKNESFVVSLMKNMTWDHIKVVSVWDLPTTSGFGHVVLGNSTNSTNYLAPSSVYVNGSEVQAKGQPTMLENCRILSENYRIRWTLNEDEDTIDIGLEAAIPFVNYMAFGWANPNASSKFMSGSDVTITGFREDGMPFADDFFISKYSECMIKKDGSAEGVCPDTVYEPSDQVLVNNTRLVYGHRRDGVSFIRYNRPLNSVDKKYDIRLYPKDKMTVIWALGLISPPDSLRPFYLPQNHGGTYGHLTLNISEHVNDCLGPLDADDKQDQDLVIADKKEPLIVTAGPALYYPNPPNPSRVLYINKKEAPILRVERGVPIIFSLQAGHDVAFYITSDPIGGNATLRNASETIYFGGPEAEGVQASPTEMTWAPNRNTPNLVYYQSVYTQKMGWKVEVVDGGLPDMYNSSVILDDEQVTLFWTLSENSISMAARGEKKSGYLAIGFGRGMVNSYAYVGWVDDDGKGRVSTYWIDGRDSSSIHPTYENLTNVRCKSENGIITMEFTRPFLPSCIKDDRPECKNIIEPTTPLRVVWAMGAQWSEGHLSVRNMHSITSSRPVLVLLMRGSAEAEEDLRPVLAVHGFMMFLAWGILLPGGILAARYLKHVKGDGWFQIHVYLQYSGLAIVFLGFLFAVAELRGFAFNSLHVKFGLLAIMLVIAQPLNAYLRPKKPSPGEEVSSKRVLWEYVHVVTGRCAITESKQGCRQRLASF

>IbDExDH35

MQQRIHAQDNKPKKNNLFPVNHVAIETPMQKTTTSQCGALTQGHSMIRTCHLGTTSSILQNTTQGNNTGPDSVGVVTQIDDTNMQRRAPGPTRNLSQEFDQVVDRNEIHGDYVDLGDPIHYCENCSALFWFEERVNKRNVRGSPKYSTCCNQGKITLPPINQPPKQLLDLFFKFSEKRTHFLENIRSYNSMFCFTSMGGKIDNSINQGSAAPVFRMYGQNFHLIGSLLPPDGIKAKFAQLYIHDTENEINNMICSVRKQDEKHTIHQDIVADIKDVLDNHNVLVKSFRNAKDVIETNPRVEIKMRLIGKRAKDARTYNLPSTSEVAALIVGDLDLSIGNRDILVESKSGRLKRISELHPSYLPLQYPILFPYGEDGYREDIQFVRTSNNLNGGRQRVTAREYFSFKIHERQYEISTILFSRRLFQQFLVDAYTMVESGRLIYIRTNQKALRCEAYKGLSDALTRGEVDPSTQGKRIILPSSFTGGARYMIQNYQDAMAICRWIVIYTMEFQKRGLPHAHILLFLAKNEINSSPNYMDDIISAEIPCKDSDKEYYDVVEEFMIHGPCGALRKKSPCMVNGRCSKHFPKKFSSASSFDADGYPVYRRRDSGRTVTKNGIELDNRYVVPHERALLLKYRAHMNVEWCNQSRSIKYLFKYVNKGNDRVTAEFYKSPVDANGNEVVDEINMYYDCRDACYARGLLDDDKEYIDAFEEASHWSSGQSMRKLFVTLLTTNSMNRPEVVWGLVWQHLAEDANISQRRLLQNNEFPPMPLPNFDSSINSGNILLYEELDYDRHALAEESVLLASKLTNEQKVVYDSVIEDAMTNKGGMFFVYGYGGTGKTFVWKALSATLRSKGEIVLNVASSGIASLLLPGGRTAHSRFAIPISITEDSTCNIRPGTDLAELIIRARLIIWDEAPMMHKYCFEALDRTMRDLLRFVNPRSAYQTFGGKTVVLGGDFRQILPVVPKGTRQDIVAATINSSYLWDNCKVLKLTKNLRLNTIGDAAEFEKLDVFAKWIASIGDGTIGEQEDGFPEIDIPSNMLLSSKNDPIATIVQSTFPMFSNEIVDHTFLESRAILAPTLDVVNAVNQYMSDMHDAESRTYLSCDAVCNSESTNGILADVHTPEFLNGIRASGIPNHSLTLKVGSPIMLLRNIDHSLGLCNGTRLIVTFRTCNRSKDFHR

>IbDExDH64

MPPVNLTANAIQAINAGDVNSKPLVQVLDIKLIGSTQERYRLLLSDSVYTQHAMLATQLNDRVKTGRVRTGSVVQLIDYICSNVQNRKIVVILNMETIIPDCETIGNPQSHLETDLGNQKPTPNRTSGPALFNGNSNMAAQSSRQTSFANSNSLSSQSVANNFQSNRPIIQPAYQPPPNYKGHGAIMKNEAPARIIPIAALNPYQGRWAIKARVTAKGDIRRYNNARGDGKVFSFDLLDSEGGEIRVTCFNAVLDRFYDKIEVGKVYMISKGSLKPAQKNFNHLKNEWEILLETSSTVDQCPDEDPSIPRQQFSFRPISEIESVENNSILDVIGIVISVNPSVPILRKNGMETQRRILNLKDQSGRSVELTLWGDFCNREGQQLQEMVDSGFFPVLAVKAGKVNDFSGKSIGTISATQLFINPDFPEAFGLRQWFDQGGKDVASHSISRELMPSISKNEIRKTISQIKDEGLGRSDKPDWVTVKATLTFIKTDTFCYTACPLMIGDRQCNKKVTKSGNSRWICDRCNQEFEECDYRYLIQGQIQDHTGLTWVTAFQESGEELLGCSAKELYMMKLDEEDDTRFSEIIKKCIFTQFLFRLKIKEESYGDEQRVKITVVKAERVDHSRESRYLLDLL

>IbDExDH61

MSHLGGGAEAHARFKQYEYRANSSLVLTTDSRPRDTHEPTGEPESLWGKIDPKGFGDRVFKGKPAELEEKFKKAKKKKEREPLSSEPIPTRQSKRRRLQEESVLTATEEGVYQPKTKETMAAYELMLNTIQQQLGGQPLNIVSGAADEILAVLKNDNIKNPEKKKEIEKLLNPIPNNVFDDLVSVGKRITDYQDGSDALVSAAANGDDALDDDVGVAVEFEENEEEEEESDLDMVPEDEEEDDGLEENGAGAMQMGGGIDDDEMLEADEGMSLNVQDIDAYWLQRKISQAYEQQIDPQQSQKVAEDVLKILAEGDDREVENKLKVVWCTRLARAEDQDMKKKIEEEMMELGPDHTAILDQLHATRASAKERQKNLEKSIREEARRLKDETSGDGDRGRRMISDRDVESGWLQGQRQLLDLDNLAFHQGGLLMANKKCELPLGSYRNHKKGYEEVHVPALKPKPLAEDEKFVKISSMPEWAQPAFRGMTQLNRVQSKVYETALFTPENILLCAPTGAGKTNVAMLTILQQIALNRNEDGSFNHNKYKIVYVAPMKALVAEVVNNLSNRLQEYGVNVKELSGDQTLTRQQIEETQIIVTTPEKWDIITRKSGDRTYTQLVKLLIIDEIHLLHDNRGPVLESIVARTVRQIETTKEHIRLVGLSATLPNYDDVAVFLRVDLQKGLFHFDNSYRPVPLAQQYIGVTVKKPLQRFQLMNDVCYEKVIAVAGKHQVLIFVHSRKETAKTARAIRDTALGNDTLGKFLKEDSASREILQSHTELVKSNDLKDLLPYGFAIHHAGLVRADRQIVEELFADGHVQVLVSTATLAWGVNLPAHTVIIKGTQIYNPEKGAWTELSPLDVMQMLGRAGRPQFDTYGEGIIITGHSELQYYLSLMNQQLPIESQFISQLADQLNAEIVLGTVQNAKEACKWLLYTYLCIRMVRNPSLYGLAADALKNDITLEERRADLVHSAATLLDKNNLIKYDRKSGYFQVTDLGRIASYYYITHGTISTYNEHLKPTMGDIELCRLFSLSEEFKYVTVRQDEKIELAKLLERVPIPVKESIEEPSAKINVLLQSYISQLKLEGLSLTSDMVFITQSAARLMRALFEIVLKRGWAQLAEKALKWCKMISKRMWSVQTPLRQFHGIPNEILMKLEKKDLAWERYYDLSSQELGELIRYPKMGRTLHKYIHQFPKLNLSAHVQPITRSVLRVELTITPDFIWDDKVHGFVEPFWVIVEDCDGEYILHHEYFMLKKQYSDEDHTLNFTVQIYEPLPPQYFIQVVSDKWLGSQTVLPVSFRHLILPEKYPPPTELLDLQPLPVTALRNPAYETLYQEFKHFNPVQTQVFTVLYNSDDNVLVAAPTGSGKTICAEFAILRNHQKGSDSVMRAVYIAPIEALAKERYSDWKKKFGEGLAHLRNGMLYLVVGNSGKHVQQVSLFIIDELHLIGGQGGPVLEVIVSRMRYIASQLDKKIRIVALSTSLANAKDLGEWIGATSHGLFNFPPGVRPVPLEIHIQGVDIANFEARMQAMTKPTYTAIVQHAKNGKPAIVYVPTRKHARLTAVDLMTYSSVDSGERPMFLSQSPNEGLTSTDQDIVKTLFENGWIQVCVMSSSMCWGVPLSAHLVVVMGTQYYDGRENAHTDYPVTDLLQMMGHASRPLLDNSGKCVILCHAPRKDYYKKFLFEAFPVESHLQHYLHDNLNAEVVSGVIQNKQDAVDYLTWTFMYRRLTQNPNYYNLQGVSHRHLSDHLSELVENTLSDLEASKCVASTNSFQFGPGEEELIRRLINHQRFSFENPKYSDPHVKANALLQAHFARQVVGGNLASDQQEVLLSANRLLQALVDVISSNGWLNLALLTMEVSQMVTQAMWERDSMLLQLPHFTKDLAKKCQENPDKCIETVFDLLEMEDDERRELLQMSDSQLMDIARFCNRFPNIDLTYEVVDGESVSAGGDDISVQVTLERDLEGRTEVGPVFAPRYPKTKDEGWWLVVGDPKSNQLLAIKRVSLQRKSRVKLDFAAPAEPGNKTYTLYFMCDSYLGCDQEYTFTVDVKEAGAVDD

>IbDExDH48

MARAPTRTLLYLYSYSKRNFFRATVLPCSNLHARTPYSRKFQDYPFHEPKLQPQPQFLHTHFAQFTKFGFLGFSGRHFSGLVGNDGSCSSPGESVEGEAGNLELGKEENEDGKVGCEKRLDFGHIVGRDPVEIYRELRDASKGEKQTRGDWDSCTEIFSCFAKSGWASNQALAVYIGASFFPTAVLKFRKFFFKKCKTDIVKYLVSLGPSHEAEEFLFPMFVEFCFEEFPDEIKRFRSMVESADLTKPHTWFPFARAMKRKIIYHCGPTNSGKTYNALQRFMEAKKGIYCSPLRLLAMEVFDKVNAVGVYCSLLTGQEKKSVPFSSHVACTVEMVSTDELYDVAVIDEIQMMADSCRGYAWTRALLGLKADEIHLCGDPSVLNIVRRICSDTGDELVEQHYERFKPLVVEAKTLLGDLKNVKSGDCVVAFSRREIFEVKLTIEKLTNHRCCVIYGALPPETRRHQASLFNDPDNEYDVLVASDAVGMGLNLNIRRVVFYNLSKYNGDKIVPVPASQVKQIAGRAGRRGSRFPEGLTTTLHLEDLDYLIECLKEPFDEVKKVGLFPFFEQVELFAGQLSNATFSELLEKFGANCRLDGSYFLCQHGHIKKIANMLEKVQGLSLEDRFNFCFAPVNIRDPKAMYHLLRFASSYAQNLPVNIAMGMPKCSARNDSELLDLETRHQVLSTYMWLSNHFEEEKFPYFKKAEAMAAGVAELLGESLTKACWKPESRSGGKPRMQEKDNREKREKSLPGQQEEKKVAAQCS

>IbDExDH46

MRQITSSAVIGSKRSLQTHNKENNSNAGIKNSYITTPNNVKVLQTQEPCISGVFNNSTNSPLSILTKSGNLIKSSINCTDNTTVTSLGLKGFERVKRHKVLLQELTNADPNKRRCHGRVYMAESSNGNNSSGRVPNSVTTTVDWTLKDVTVGSCGESHLNCTVEDNCLVRNLAQDFAMIDREHEQQPNTQEGILQAQSHLNSNETYERQVLGPGKQSVISPTPTIVAYMDIGDPNNICENCNAIYWYEERVNKVMRRTKIFYHVAMGRMYNNMFSFTSLGAKVDKSINIGNCPPIFRINGQNYHLMGGLMPEVGKSPKFAQLYIHDTENEVENRINAFSGGELNDPTHLDIVQDIKLDLDEHNRPKAKDIIVETKSGSLKRINELNPAYLPLQYPLLFPLGEDGYREDIQYGTTSNKPTGARVRVSQREYFAYRIHDRLSELSTMLYAKRLYQQFLVDAYTMVESGRLQYIRNNQKSLRCEAYKGLSDALTRGEVDTNKQGKRIILPSSFTGGARYMIQNYQDAMAICRHKGYPNLFITFTCNPKWPEIQRYMDKCNLNAEDRPDIVSRVFKMKLDALVKEICNGKLFGIVTAVVYTIEFQKRGLPHAHILIFLERLTEGFSANHMDQFISAEIPNKDLDSDYFNAVGEFMMRGPCGHERPKSPCMVNKKCSKHFPKKFAEVSTLDHDGYPIYRRRDNGAIIERNGVKLDSRYCVPHNRYLLLKYRAHINVEWCNQSRSIKYLFKYVNKGNDRVTAEFYKSTTSEKENADVDEITMYYDCRYVSACEAAWRLLSFDVQLRHPPVERLSFHLPDCQSVVFEDDDRIEHVLNRPTVSQSMFTAWFDANKKYEAARELPYIDMPSKFVWKKDIREWHPRKRGFSIGRIFFVPPGSGEVYYLRCLLNVVRGPTSFQDIRTVQGVVYQSFRDACYARGLLDDDREYIDAINEASHWSTAHSMRRLFVILLTANLVNRPENVWNHVWHHLCEDVEYTRRIVLKDMGLCLSDDEKKNLGLLELERLLQSYNKCLKDYRQMPSPNYDDAILTNNNLVFDELNYDRQAMRDECELMESQLTDEQLVVYDTILSDVQAKKGGLFFVYGYGGTGKTFVWKALSSKIRSEAGIVLNVASSGIASLLLPGGRSAHSRFAIPIAVNEDSTCNISQGSPLAELLIPAKLIIWDEAPMMHKHCFEALDKTMRDLLRHIDPNRNGTLGGPNDGHAKVEIPKEMLVPSNGDYIATIVDIVFPMFKQAGSDFQLMENRAILAPTLDVVNAVNEYMTDLHVAESRTYLSCDTVCKSDSTNGILYDMHTPEFLNGLKASGIPNHALTLKIGSPVMLLRNIDHSMGLCNGTRLIITRLSDHVVEAKIVGGHNAGNVVLIPRMSMTPTDTRLPFKFQRRQFPLMLSYAMTINKSQGQTLTHVGLLLKKPVFVHGQLYVAASRISNPPGLKILIPNEDAELGSWGRASAVVHLPTSLTSLQNDSSPRVFFHAVSLGTEPLHCGWYSSYGLIFAGDKSMCQIHSSYSLIQDGNQSVMNRINGDHVGSPLDVSGKSDDEQHDVIVSDAFENRMRRLSSVVGTSSNVQSDASSPESPYRRATSLPYSSGADLSWSAPPRARRGLGHSITAPASTHRNLILEGQEVVFSRSMADKRSLQGTELRLDRLSGVRRLQCSVGTREMFSHSWLWLRDFRHDFSISLVGLTILTASYTPICTYFDAPGVDFYPARYGQKQRLHSLNTGDYSTEKTVKGHYWINFFQLVLNQI

>IbDExDH50

MAMIISPSPRTLSSSSPITQMFQLPRSSSSLSSTRISPPALHSRKSTPISAKASSNSPAVFSEDISDVLGEVSIFRATGEPVKFKQLWDQQEGVAVVALLRHFGCVCCWELASTLRDSKPRFESAGTKLIAVGVGEPKKAQILAERLPFPLDCLYADPDRKGLYYGLGRTFFNPASTKVFSRFKELREANKNYTIAATPDDRSSVLQQGGMLVFKGKQLLYARKDEGTGDHAPLDDVFDACCKLPVTSPLEYSSPHMELVLSFSLSFHVNILKTLSIYKGEYINQAIDCATHFSGTVNSFYNNFRVTLENRLPTPYLFDAQAYAITVEHAPILWMEPAEIKLFVGGVSHNTNEQTLRDYFSKYGELRSCDIIRDRITGLGRGFGFIVFADSSVVEHVLSDTHVILGKKVEVKVAKPKGSREAMLETRSVKKSFCRGIPPDSTDEELVQHFEKFGIVEDAEIRRPRGFGFVTFESEEGAKNALRIPFQYMKNKRVEVKVAEVKAKVGLNNGMRNSLPNMTFPIFPTTNWVNGNPQLANYVPVPHCTAPTINWYSYGASEFCYNSYYPAGYNGGNWQDLNNPNPSPVVTRPPLASSQPVVIRPPPSSSKLVVGPPPASKPVVIRPPPAKVEEQNGNVAVEIDEIQGDEIDDKSLTNGEVQIQDEIDGESLTNGEVETQDESDVAAGDSF

>IbDExDH63

MKFSSKEEHRSVEEDGPYYADEINDDSQDEGKKRDFTKLELKPDHANRPLWACADGRIFLETFSPLYKQAYDFLIAIAEPVCRPESMHEYNLTPHSLYAAVSVGLETETIVSVLSKLSKTKLPKEMIDFIYASTANYGKVKLVLKKNRYFIESPFPEVLKKLLQDEVIGRARISSEGVHGNDGFTVSKSAGEIEGRHDELLNEAELAAAAEEKETHSFEIDPSQVENVKQRCLPNALNYPMLEEYDFRNDTVNPDLDMELKPHAQPRPYQEKSLSKMFGNGRARSGIIVLPCGAGKSLVGVSAACRIKKSCLCLATNAVSVDQWAFQFKLWSTIREEQICRFTSDSKERFRGNAGVVVTTYNMVAFGGKRSEESEKIIEEIRNREWGLLLMDEVHVVPAHMFRKVISLTKSHCKLGLTATLVREDERITDLNFLIGPKLYEANWLDLVKGGFIANVQCAEVWCPMTKEFFAEYLKKENSKKKQALYVMNPNKFRACEFLIRFHEQQRGDKIIVFADNLFALTEYAMKLRKPMIYGATSHVERTKILEAFKTSKAVNTIFLSKVGDNSIDIPEANVIIQISSHAGSRRQEAQRLGRILRAKGRHQDRMAGGKEEYNAFFYSLVSTDTQEMYYSTKRQQFLIDQGYSFKVITNLPPPDSGPELSYHHLDDQLALLGKVLSAGDDAVGLEQLEEDADDIALQKARRSMGSMSVMSGANGMCWEKRARRACAEEQAERSIQETPLIQETLWLRLESTHGCVLSLTITLLNEKLGVSDIVIPRASAHQSSLKYSSRIGYGCDMLVCSMW

>IbDExDH59

MRDLLCFVDPNSSSKTFGGKIVVLGGDFSCKDLKLTKNLCLNAVPHEVDKQKVVDFANWIADIGDGKTSGPNDGYAEVEIPKDMLLPSDEDHISIIVKSTFSMFVNVNSNCTFLEGRAILAPTLDVVNQVNAFTSLRGERHTTEWGSGPAALDMVLGAKKRTRSLMASDWRPMKHDQLLLHDVPVGYNSSLILEQIGNFLGSFVKGDDRFAGAPWLDFYRIRVALPVDKPIKRRMKLLKRDKSWCWIALFVGGSPRKSWPEGGVGNIAAVAVDVVESWAVVAAVSKAVGGGHRGAIMSALERLRVKLGLEGLFYVDNVGLSGGLALFWRKNNTARLLSFSKNHVDVEVSIGGLGIWRMTGFYGFPERSRRAESWDLLRTMAGRSLLPWVVIGDFNDLLLQSEKRGEKKRGGMEERLDKVLAGVDWCNLLPRASVSNILSRNSDHSALFLGVNPSMRAQARPRKIFKFEMAWVHDEGCLRQVEEAWEEGRNAGLLACLHHCGNRLTRWGGTTSISLGRRLPNLGKDSFSLKEDAFWKQRAKHHWLRGADANTKFFHRYASARKRKNSLSRLKDDSDVWVEGDALNTVVLDYFHHIFASNNAAINLESFTASISPGVTPEQNDALLLPFCESKVKAALLSMFPDKAPGPDGMNPGFYQHFWDVVGPDVSAFVLKCLNECSFPEGLNNTNVVLIPKKNTPEKVSDLRPIALCNVVYKIMAKMIANKMKPAGNIISSHECFIPNRLIIDNILIAAEVGHYLNRKRCGVKGWGALKLDMAKAYDRVEWPYLRRMLLALSFSVVWVDLVMLCVTTVSYNFLVNGIDVEGLSLLLQQAKARGDFHGCRVARGAPPVSHLFFADDSLLFFRANAQEAEAIKQCLTVYEHMSGQAVNYHKSSVCFSRNTAGVDRDNVAAILGVTLAPNFVCIAIERAMNRYWWGSGNERGIHWKAWDKLCAPKKFGGLGFKDLRAFNLAFGVRRRVGNGNSTLIWGHPWLPDEPDPMVQTIMPHGLDGSLVSGLLDPTTGTWDHSIIHDIFHPNDVSRILKVPVAPLYEDSWFWPGDLKGIYTVKEGYKRVVGDFQPTTGIYDKWLHLWRIKCPAKWKIFIWKALSNILPTTTNLIIKRVEISPACPIGFISSLEKDAMSGVEASSRRSYHHHSNAATVGAVLQTQDGTFIAGFNGRLPPCFSPLRAESLACKEALSWLKDRGLTSVHIYTDCSTPGEFVNYELTVSLLPLPRRGSHQSRRATFVCDFSVFTTSIAKHRAPGRTALSPQFYLSRRKRWSLNSTEEGRGEVDYEVLTALTSRYNDIVILDTAESRVLLLDSSGNVHSILYKAKKWTNAYWDDFATFPAIVPKGPIAILGLGGGTAAHLMLELWPSVQLEGWEVDDIVLPFHCHCSVAFVCEKAREYLGLSDLEEHNEVGGALKVHIGDALSPSVAIPGGYAGIIVDLFSDAKVLPQLQEINWKKLPRTAGENYLALTGPLPDLAVWSACLPEELSSIVKQWKACQFS

>IbDExDH54

MSMLYVLAKDISPQSYKKAIRLRLIRTYIVSEGRNKSAVKSQECVFHDIEGSYVHASIHADFIDQFSHLLKEGKVYAVKNFVAVSYYYQYKTTQHKYMMRFNQYTTIERHRRKGFPSLLFRIKPIEELLAGKVEEKLLIDVIGRVVEFYSPKDKVIAGFPTRLVDFLIEDSNLKANCSPLRSISMVSYGHSANNSQSPMVVSTIRDLYERAEEGYYYVPETYYFCDTYKTNWAEGNVRYKVIVRVLDKTSDAPFVLWDKDCFELQGITAYDLKIKYSMSKMQVPIEFEQLRNKSMVFRINLKNEHIRNPAKPISVLSVSHNEELEAQYCPSMLDDQDELSRMIEEDADDLESEESESGDEAISPIVQANLKLKGVANDQDEVDTGAIKRCLLDQFSSSQNLKKCRPLVIKEEKTL

>IbDExDH47

MARAPARTLLYLYSYSKRNFFRATVFPCSNRFLHARTPEKFQDYPFHEPKLQPQPQFLHTHFAQFTKFGFLGFSGRHFSGLVGNDGSCSSPGESVDGEAGNLELGKEENEDGKVGCEKRLDFGHIVGRDPVEIYRELRDASKGEKQTRGDWDSCTEIFSCFAKSGWASNQALAVYIGASFFPTAVLKFRKFFFKKCKTDIVKYLVSLGPSHEAEEFLFPMFVEFCFEEFPDEIKRFRSMVESADLTKPHTWFPFARAMKRKIIYHCGPTNSGKTYNALQRFMEAKKGIYCSPLRLLAMEVFDKVNAVGVYCSLLTGQEKKSVPFSSHVACTVEMVSTDELYDVAVIDEIQMMADSCRGYAWTRALLGLKADEIHLCGDPSVLNIVRRICSDTGDELVEQHYERFKPLVVEAKTLLGDLKNVKSGDSLPPETRRHQASLFNDPDNEYDVLVASDAVGMGLNLNIRRVVFYNLSKYNGDKIVPCSSAQVKQIAGRAVKKVGLFPFFEQVELFAGQLSNATFPELLEKFGANCRLDGSYFLCQHGHIKKIANMLEKVQGLSLEDRFNFCFAPVNIRDPKAMYHLLRFASSYAQNLPVNIAMGMPKCSARNDSELLDLETRHQVLSTYMWLSNHFEEEKFPYFKKAEAMAAGVAELLGESLTKACWKPESRSGGKPRMQEKDNRKSARNHYQDSKRRTRLLHNAPNEVAGQKEMDQDCAFLTQRLNGMIYSKMGSHVRFLAKTYAFSTQMEQNTILAILHQFVECWLLPPASRCSL

>IbDExDH52

MDAYSLKSVQELPPMFRSVFSFRYFNSLQSECFPACFLSDVNMVISAPTGSGKTVLFELCILRLLSRFISTEGKFIHVKGSLKTVYVAPSKALVQEKLRDWNQKLGSWGINCLELTGDNEYYNNKDIQDADIILTTPEKFDAVTRYRIKDGGLSFFSDIALVLIDEVHLLNDPRGAALEAVVSRIKMLSCISEMKLSPLAHVRFLAVSATIPNINDLAEWLMVPNQGIKKFGEEMRPVKLTTKVFGYTPAKNDFLFEKIFSCNIQEANLHLSSAQPEKEHKRLHNDSLGYHNGGLSMKDRNLIEGLFLNSDLQVLCTTNTLAQGINLPAHTVIIKSTQHFNQEKGLYMEYDRSMILQMCGRAGRPQFDDAGVVVIMTRKETVHLYENLLNGCELVESQYAVLNITSMHYGALNCTDCSTDSVRYYKGNRMDEMLIFNPENYAMTKLLASNNIERHLQEICVKKVNELSRYQMIWTDEDGFLLKPLEPAKLMTKYYLKFDTMKHIMQASANCSIEDALHIICHAEEIAWIQLRRNEKKLLNDINNDKNNRLRFHILGDKNKRKKRIQTREEKIFILANDCLTGDPLVHELSMTQDMNSICTNGCRIAKCMKDPYLLKQLPGIGMVTAKALNSMGVKSFEALADADPRKIEMVTGRKYPFGNHIKGSLLSLPPKIEMEVKETESQRQGKSKVVITLTRMSQPALMTKGYYADLVVGLGEGNLILFHEKIRVHDFPSPYSATVIVPNPQKRKLTVKADLIFDEFIGVDIHQKVTVMKEMNQDISNKHKTRQNPSFQLKEAYIVEDNKDSTSEAPIEEPLNVDESEGFFDMPSFTLIDEEPDKEVVTAVGTEDDECKVITERTIFDHIREKAKSLPPLGMLGDPCFTSLETLAQIRKRTRERLLAVENPIEVLDEGINKVARHSMTIQSARGHQLEKSFSDKDATSESHYISKDNNPTNDTGKHPSEPNSVQLVDLTKKPEFGQMQGSPILGRLKTTGSKLYTLGNQNWFSPQSSHTNSDFEGFKARKSEVIVLDPEPVERKQHEAIGVTKLRDNVCSGSAFSRSDITSQSSVVSSSPPCTSLPRNEVGDLRNSSETKCKQHMSTPSLLERQLSFPAVVQDTREMNCFLGFKSVFTFL

>IbDExDH53

METRRQEAHSGEYRRPTSEWCQLKPETGQLLSPFIRTSHSKFVMPLFLNKDFTCNISQGSDHVELIIRSKLIIWDRAPMTHKHCSEALDKTMSDLLRFIIPGSAEKTFSGKTGLVVYNGLPEIDISAQVLLKLGPNPITNIVEITFSSVRYVMLDESHIEDPSDPLSDFRCR

>IbDExDH56

MENPTLPVHNENENVVENNNGGNDNNIAESQTPPVNVFSQDGGSTGGYIPTMRVPTIPSGSVERPEKFTGIGSNFKRWQHKMLFYLATIGLSRFLPRGFQGLDHKYKSEDAAAKKFVVGRFLDFKMVDSKTLLSQVEDLQLIFDEIRDEGMVLSEAFRVASIIHLLPPGWKDFKNYLKHKRKEMNLEDLIQRLRIEEGNMRSDGKTMMTPQTVGFRIGDAVEVTSNDEGFVGSYYEATIVGHTEGKRVGYGVGAFSYLPMHSGILVDSPAFTPVNPIVNVIGSERLYGGFMMCSPRKPINMSTNVNVHPTSFEDNYVATRGTPLSATTNVTSNPNSYALNVEIPHVTPLSVITDVNMRDIPSPFTQCSFNISNMSTSTSSSSQPLADVSNVERALDPHCNRALLSTLRNSSKVSSRTDVKNLNSDYEQVPSHNKAYDSYFDLGDASHSCNYCGALFWRQERSNQNVTRGLPKYTKCCYEGKIQLPRMKDPPSVLQNLYFGHTEQSTHLLNNIRRYNNMFCFTSLGGRVDRSINTGKAPPVFRISGQNYHCIGSLVPGEGSTPKFAQLYIYDTDNEINNRINSVRMARTEIQSNPVVEVKMNLIGRRSKDGRTYNLPTANEVAALIVGDLDPSMGDLDILIQSRIGQLKRINQLNPAYLPLQYPLLFPYGEDGYREDISFSDAWHQRHHGGRKRISPKETHQKALRCEAYQGLSDALTRGELDPTDRGKRIILQSSFTGGARYMIQNYQDAMAICRQIGYPHLFITFTCNPKWPEIERYVAHCGLKPEDRPDIICRVFKMKLDAMIEDIKTQKLFGDICGGLIPEIDAIISAEIPDPDVDTEYHDVVGEFMLHGPCGQLRKNSTCMINACEATWRLFGYAIHYRTPPVERLNFHLEHQQNVVYGEDQTLDEIVENQTVKQSQFTAWFEANKKYEDARSLTYAKFPNDDKEYIDDITDSRLLRLLQVQSVGQRSYGMLFGNFLQKMHSFIIDSLRDFPQMPLLDETNIGFMKNMLIAEELAYDKESLKIEHETLITQLIEEQKNVYDSVMNDIDCNRGGLFFVYGYGGTGKTFVWRTLSSMIRSRGDIVLNVASSGIASLLLPGGRTAHSRFAIPLSLNEDSTCNISQGSDLAELIIRTKLIIWDEAPMTHKYCFEALDKTMRDLLRFTIPDSAQKTFGGKTVVLGGDFRQILPVIPKATRPIVVGATINSSYLWTNCKVLRLTKNLRLRTLASEEDKQTVDWFSKWIANIGDGITGVVNNGLSEIDIPPRFLLNCGHDPIATIVESTFPSARYDMIDELDLEGRAILSPTLDVVDQINQYMCNMNTAEGRTYLSSDSLCKVESDGENLSQVHIPEFLNSLRLSGLPNHSLILKVGAPVMLLRNIDHSLGLCNGTRLIVTRLTDHIVEAKIVNGTHQRTKVLIARMSLTPSDTRLPFKFQRKQFPLMLAYAMTINKVRVKH

>IbDExDH60

MATLRWEVMSLSLSGGDLEIDSLCFNRFRWEANEAARCDWTDRRHSHRTSRSSPVAGKPAPSPPFVLPPSPSSATVAAGRRRRRKKPDGAGDRQQSPPLRRRDRRKDRNGRLNSDQDADRRSLMASQPSDSKKKKQKKPKKRVREDSELLEHLDSLPWNSSLPETDDAFSHLFGDDELGGGFLSLEEVDETTYGLEISKSSGGNEQPKSKGKPKTKKQKISESHDDSNGEEGDDKADEEIQQKKRKEFPTTKNDDEEDSVDETEFYAWNEMRLHPLLMKSIYRLNFKEPTPIQRACIPAAAHQGKDVVGAAETGSGKTLAFGLPILQRLLEEKEKADRLNAEDGEVDEKIAPRGLLRALIVTPTRELALQVTDHIREVAKHTNIRVIAIVGGMSTEKQERLLKRRPEIVVGTPGRLWELMSGGEVHLVEEGMPGKKY

>IbDExDH49

METGDGDKNKPTVDSPTSVLEDEEEAFIDAKNGDSSNVSQSMLKEEEILSENPLKEEEEENDPNKTEELNETQFSRLDELLTQTQLYSEFLFEKMDDITVKKAKRAVAAMLTRSKEGDSSDYSSLTEEERAEKEQSELVPLLTGGKLKSYQIKGVKWMISLWQNGLNGILADQMGLGKTIQTIAFLSHLKGNGLNGPYLVIAPLSTLSNWLNEINRFVPSINAIIYHGDKKERDELRRKHMPRTIGAKFPIVITSYEVALSDARKHLRHYGWKYLVVDEGHRLKNSKCKLFKELKLLPVENKLLLTGTPLQNNLAELWSLLNFILPDIFSSHDEFESWFNFCGKGNNEDSNEDLAEKRKAQIVAKLHGILRPFLLRRIKADVEQMLPRKKEIILYATLTEHQKNFQEHLINKSLEGHLIKNVSTMRGFKGKLNNLMIQLRKNCNHPDLLESQFDGSFLFPPVEQIVTQCGKFQLLDKLVGKLLARKHKVLIFSQWTKMLDIIDYYFSEKNLEVCRIDGSVKLDERRRQINEFNDVNSTYRIFLLSTRAGGLGINLTAADTCILYDSDWMDLQAMDRCHRIGQTKPVHVYRLATAQSVEGRILKRAFSKLKLEHVVIEKGQFQQERSNPNCTDILQEEDLLALLREEENAEDKLVQTDISNEDLERVMDRSDLVVVHSKENGKPQQACSNALPLKGPGWEVVVPTATGGMLSGLNN

>IbDExDH57

MKDPPHVLQRLYFGDTERSRHFLSNIRRYNNMFCFTSLGGRIDRSLNTGNAPPVFRISGQNYHCIGSLVPIDGSMPKFAQLYIYDTDNEIKNRINSVRGESNLGDINEEIVVELKNMLDQNNVLVNLFEWQARSNLIPVIEVKMNLIGKRNKDGRTYNLPTAHEVAALIVGDIDPSMGDLDILIETRTKQLKRINQLNPAYLPLQYPLLFPYGEDGYREDIIFSDAWRQCHPRRRIRISPKEYFSFYIHEKVNEKHTLLYSRRLFQQFLVDPYTMIESARLIYIRTHQKALRCEAYQGLSDALTRGELDPAARGKRIIPPSSFTGGARYMIQNYQDAMAICKHIDDRPDTICRVFKMKLDAMIEDIKTDKLFGDICGVIYTIEFQKTGLPHAHILLFAKRMNRPNSANEIDALISAEIPDPDVYAKYHHAVSEFMLHGPCGELRKNSPCMVDANALNGDVVDEINMYYDCRYISACEATWQLFGYWIHYRTPPVERLNFHLEHQQNVLYGEDQPLDEIVENQTVKQSQFKAWFEANKKYEEARSLTYAEFPSKFVWKQDLREWQPRKRDDDKEYIDGIRFEFGIRRMHRCRRVMRIVCFGGIGEVTIFVGEKPKGLSKMPIPDESSMCLSENILIAEELAYDKESLKPEHETLVTQLADEQKNVYESVMNDIDSNGGGLFFVYGYGGTGKTFLWRTLSSKIRSRGDIVLNVVSSGIASLLLPGGRTAHSRFVIPLSLNEDSTCNISQGSDIVELIIRSKLIIWDEAPMMHKYCFEALDTTMRDLLRFTIPGSAEKTFGGKPVVLGGDFRQILPVIPKATRPLEEDRHMVDWFSKWIADIGDGIAGVVNGGLSEIDIPPQFLLKCGHDPIATIVESTFPSSRYGTLDESQLEGRAILSPTLDVVDQINQYMCGMNTAEGRTYLSCDSLCKAESSGENLSEVHTPEFLNSLRLSGLPNHSLTLKVGAPVMLLRNIDYSLGLCNGTRLIVTRLTDHIVEARVVNGTHKGTKVLIPRMSLTPSDTRLPFKFKRKQFPLMLAYAMTINKSQGQTLTHVGLLLKKPVFNHGQLYVAFSRVTHPDGLKVIALDEDEQDCVATTNVVYKEVFNNV

>IbDExDH58

MDTRLTRRASQLRTVDLATIGRIDLVNTSSRKPLNNITNGTTQRTFQIEGSYTTSTMCQNDINHSSRSTLNCSRTCISTQLTHITKASSSGGHLVHNDYDERITTCEETARDLLLEFDNVDQPQNMEQSCIIQPVAYKDIGDPSYNCEYCGAMFWFEERMNKAVVRGTPRPDGGENHIHTEIVADIQRDLDINNVLVKSFRMARDELDNKPRVEVKIKLLGKRNKDARTYNLPIVSEVAALIVGDLDPSMGERDILVESHGGGLKRINELNPAYLPLQYPLLFPYGEDGYRDDIPFNSSRGNGNRCRQRITPREYFAFRLHERLMETSTLLYSRRLLQQFIVDGYTMVECGRLKYIRTHQKSLRCETYNCLTDALTHGEVNPAAQGRRVILPSSFTGGARYMIQNYQDAMAICRWIGYPNLFITFTCNPKCSQQYKQHEYILGLKFCKLAAIVIKPQFRCIIGANLPFTVLARQSWIKTITGSGSVELKYGSMAST

>IbDExDH62

MSHLGGGAEAHARFKQYEYRANSSLVLTTDSRPRDTHEPTGEPESLWGKIDPKGFGDRVFKGKPAELEEKFKKAKKKKEREPLSSEPIPTRQSKGGFRRKVCLPTEEGVYQPKTKETMAAYELMLNTIQQQLGGQPLNILSSLRRMRRRRRKVILIWCRRMKRRMMDWKRMDAGAMQMGGGIDDDEMLEADEGMSLNVQDIDAYWLQRKISQAYEQQIDPQQSRKVAEGVLKILARFGTRLARAEDQDMKKKIEEEMMELGPDHTAILDQLHATRARQRQLLDLDNLAFHQGGLLMANKKCELPLGSYRNHKKGYEEVHVPALKPKPLAEDEKFVKISSMPEWAQPAFRGMTQLNRVQSKVYETALFTPENILLCAPTGAGKTNIVYVAPMKALVAEVVNNLSNRLQEYGVNVKELSGDQTLTRQQIEETQIIVTTPEKWDIITRKSGDRTYTQLVKLLIIDEIHLLHDNRGPVLESIVARTVRQIETTKEHIRLVGLSATLPNYDDVAVFLRVDLQKGLFHFDNSYRPVPLAQQYIGVTVKKPLQRFQLMNDVCYEKVIAVAGKHQVLVSTATLAWGVNLPAHTMLGVLEGLNLTPMGGHNYNRAQLNCMQNAKEACKWLLYTYLCISMVIRNPSLYGLAAADALKNDITLEERLIWRVPIPVKESIEEPSAKINVLLQSYISQLKLEGLSLTSDMVFITQSAALAYRGFEIVLKRVGEKDLAWERYYDLSSQELGELILLCYPKWDRTLHKYIHQFPKLNLSAHVQPITALSLEDTNDREYILHHEYFMLKKQYSDEDHTLNFTVQIYEPLPPQYFIQVEFKHFNPVQTQVFTVLYNSDDNVLVAAPTGSGKTICAEFAILRNHQEGSDRYRVYIAPIEALAKERYSDWKKKFGEGLGMRVVELTGELATDLKLLEKGQVVISTPEKWDALYVRWKQRKHVQQVSLFIIDELHLIGGQGGPVLEVIVSRMRYIASQLDKKIRIVALSTSLANAKDLGEWIGATSWGSPTSPGVRPVPLEIHIQGVDIANFEARMQAMTKPTYTAIVQHAKNGKPAIVYVPTRKHARLTAVDLMTYSSVDSGERDVLITISK

>IbDExDH55

MQNNISFDLRVAEWHSPLSHPVIMGASGSEGFGSIYPTPIAARFPAVDSFPNRRKRLKTNEAPINQIPSLCWREELEERLLKRSSGILDYSDPYSMSNLWGSLECGKYGSVTKEIEELMAQSRRCIDSCYARDPTLPYKFLELEKNHTTEYKGDQSATAVIDLEDEHVARNVPVARFVPPAQLVPSAGPLLILDSDDEDNKKPNCTLQGVLSINTVGGSYLKDHLDSPGTKTPKGSANLAFQTEKIKDKGVYVGVEDDSETEDGNDANFDGLDDIWNEMSFAIECSKDVTVDASSNKDKAEDEDEECEHSFILKEDIGYVCRICGVIKKSIESIIDYQYSKSAKNARTYRYEGRTTKDSGPSENLFEPNKPSHEFELAEISAHPRHKKQMKPHQVEGFNFLLNNLVTDNPGGCIMAHAPGSGKTFMIISFLQSFMAKYPFARPLVVLPRGILGTWKKEFLRWQVEDIPLYDFYSFSIIVCDNEASRGCCLPEILLDSPSILILDEGHTPRNGIDVLTSLEKSKVSKLEDSKAIKRRILASMRMRGRLQLYKIEGDDKKSSSLLQGRLSEELRGLVDFTVILKLHPKAKIEVLWVKELRRKFKISLEGSALYVHPQLKSLSKTSVKERIDEEKIDMIVDNLELREE

>IbDExDH72

MSSSTNGHNDEVVDEINMYYDCRYISACEATWRLFGYAIHYRTPPVERLNFHLEHQQNVVYGEDQTLDEIVENQTVKQSQFTAWFEANKKYEDARSLTYAEFPSKFVWKQDLREWQPRKRGFSIGRLFYVPPGCGELYYLRCLLNLIRGPSSHDDIRTVPGVIHKSYRDACYEYGLLDDDKEYIDGITDSSSWASASALRRLFATLLSSSTISRPEIVWDAVWEFLKMQLHHRRRMNNPDSDKKQFALVELEKLLSLWGKSLRDFPQMPLPDETNIGFMENMLIAEELAYDKESLKIEHETLITQLTEEQKNVYDSVMNDIDCNGGGLFFVYGYGGTGKTFVWRTLSSMIRSRGEIVLNVASSGIASLLLPGGRTAHSRFAIPLSLNEDSTCNISQGSDLAKLIIRTKLIIWDEAPMTHKYCFEALDKTMRDLLRFTIPDSAQKTFGGKTVVLGGDFRQILPVIPKATRPIVVGATINSSYLWTNCKVLRLTKNLRLRTLASEEDKQTVDWFSKWIANIGDGITGVVNNGLSEIDIPPRFLLNCGHDPIATIVESTFPSARYGMIDELDLEGRAILSPTLDVVDQINQYMCNMNTAEGRTYLSSDSLCKANRTVKIYHKYTLEFLNSRDYRTSQSFIDIKVKMGSRSSLAGMEVPIIGSDAIKWIQLSVPSSASTPAEAAAVLPDHLNRDTASYAVFGNPPTYLIWRINKSQANVVEIMQLNDDKEFPNIGLQIVFPDALFPFTLIWVAYLIKLKDVSTYISSSVLPSSEVIECNTQMNPHHGAITAVAATAGSIVVGRNDGSVSCFQLGMVEPSAPG

>IbDExDH76

MSHLGGGAEAHARFNRFEYRANSGLVLTSDSRPRDAHEPSGEVETHPGNIDPKTFGDLVYKGKPAELEKFKKGKKKKERESPSSESIPTGQSKRRRIQEESVLTATEEAAARGQPLNIVNGAADEILGILKNENIKNPDKKKEIEKFLDPIPSNIFDELVSLGKCITDYQDGSAVVSAAANGDEALDDDFGVAVEFEENEEEEGGSDLDMVLEDEEEEDGLEENDAGAMQMGGGIDDDVMQEADEGMALNVQNIDAYWLQRKISQAYKQQIDPQQSLKVAEDVLEILAEGDDREVENKLLLHLGYEMFGLIKYLLRNRLKVVWCTRLARAENQDMKKKIEEEMMDLGPDHTAILDQLHATRASAKERQKNLEKSLREEVLRLKDETSRDGDRGQRLISDRNVESGWMEGHSQLLDLDSLAFHQGGLFDDKQEPKPLSEDETFVKISSMPEWVQPAFRGMTQLNRVQSKVYQTALFTPENILLCAPTGAGKTNVAMLTILQQIALNRNEDGSFNHNKYKIVYVAPMKALVAEVVSNLSNRLQVYGVNVKELSGDQTLTRQQIEETQIIVTTPEKWDIITRKSGDRTYTELVKLLIIDEIHLLHDNRGPVLESIVARTVRQIEMTKEHIRLVGLSATLPNYDDVAVFLRVDLQKGLFHFDNSYRPVPLAQQYIGITVKKPLQRFQLMNDVCYEKVIAVAGKHQVLIFVHSRKETAKTARVIRDTALGNDTLGKFLKEDSASREILQSHTELVKSNDLKDLLPYGFAIHHAGLVRADREIVEQLFAYGHVQVLVSTATLAWGVNLPAHTVIIKGTQIYNPEKGAWTALSPLDVMQMLGRAGRPQFDTYGEGIIITGHSELQYYLSLMNQQLPIESQFISKLADQLNAEIVLGTVQNAKEACKWLLYTYLCIRMVRNPSLYGLAADALKSDITLEERRADLVHSAATLLAKNNLIKYDRKSGYFQVTDLGRIASYYYITHGTISTYNEHLKPTMGEIELCRLFSLSEEFRHVAVRQDEKIELAKLLERVPIPVKESIEEPSAKINVLLQSYISQLKLEGLSLTSDMVFITQSAARLMRALFEIALKRGWAQLALKALKWCKMISKRMWSVQTPLRQFHGIPNEILMKLEKKDLAWERYYDLSSQELGELIHYPKLGRTLHKYIHQFPKLNLAAHVQPITRSVLRVELTITPDFIREDKVHGFVEPFWVIVEDCDGEYILHHEYFMLKKQYSDEDHTLNFTVQIYEPLPPQYFIRVISDRWLGSQTILPVCFHHLILPEKYPPPTELLDLQPLPVTALRNPVAPTGSGKTLCAEFAILRNHQKESASIMRAVYIAPIEALAKERYSDWKRKFGEGLGMRVVELTGETATDLKLLEKGQIVISTPEKWDALSRCWKQRKHVREISLFIIDELHLIGGQGGPILENGLGPPLMDFSTFPPAVRPVPLEIHIKSIDIANFEARMQAMTKPTYTAIVQHAKSGKPAIVYVPTRKHARLTAVDLMTYSSLDSGERPMFLSESANELEPFIEGIKEPMLKETLKYGVGYLHEGLISIDQDVVKTLFENGWIQVCVMSSSMCWGVPLSAHLVVVMGTQYYDGKENAHSDYPVTDLLQMMGHASRPLLDNSGKCVILCHAPQKDYYKKFLFEAFPVESHLQHYLHDNLNAEVVSGVIQNKQDAVDYLTWTFMYRRLTQNPNYYNLQGVSHRHLSDYLSELVDVKTSIQIAFPLHILHIYTHTKK

>IbDExDH67

MYQILPKRFVDSSSFDQDGYPIYRRRDDGRTITKNGIQMDNRYVSATSYLEVIAMTSVRNSAVERPSFHLRITKIVYFQDDEDVETIFESTDLFEDLEFEDIKTYNGKEYNTFKDACYARGLLDDDLEYIDAVKEASEGSAHSKKVVCQPTWQTQWEDFEYVGEVWWYLVKMLNLIGKVLNDQGLSWKSYHAIVSFLLKKVGYFVFRLTDEQGHYKAYIRAKGQIVINVASSGIASLFLPGGRTAHSRFAIPISINEESTYNIHQVPIGLLEKSSCIRATLGKFYCGAKGIKTDIVSCINSSYLWKPLRKLSPNKEFEVMVLIGEDTIVILARLISHEMLLQCNGDLLQLLLILSLSSAVIGCSKINEHVVEGSILADEIGTRVLIARMAIAIDTRLPFKFNRRQFPLMLSYAMTINRVKGKLCPSCSCEGVLVPPDQCCNHKDDKGAFKHVTIRGRLPLVLLQLSLSSLEPPCVFVADFDLSFCFLFLRSLFSSVSPFLLACDGRLFFLRFLGYCGLDYSRQEINCVDLNEVNDGGVNVNNLDVNDGEQNDNVDVGDEKDDDEKECENNDNQDQFERKKRKRISKAHTDFSEVTSKDGSIKLQFRIAKETFSRSHKLPLGGKLFHVRKSIEKLILDCVTRWNSTYEMLVVAIKVKDAFPIFAQREPSYKCCPSLEDWSKIEHVLSILEVRDALHDLFYEYVEMDNMKTRKDVTPNKSELDIYLEEGVYRCQDGEVTSEFDALAWWKSHELKFSILSKLARDVLAIPISTVASEATFSAGTRVLDPYRAKLSSDMVQVFGLWSGLGSSTSWDQ

>IbDExDH73

MYEVPEVRGAQTSKSLELLFHDAGGEVRITSSYDVTQLWFNQTFPEFVDFRSKLAANTSPVKSISTTFVLSHSTGPSEFQSGAIIVSTLSDVFEAIEDNDFYVPAEIMGIEGYENGILHILHEGRYKLLVRVVDKTCDAPFLLWDREVAELVGVPTSLLYQKYSKGTMRGPNSAYNVIRVLRDQLLLDTYCSNLRDHQDKDLMSKMIEEDQDENSESDEETSKDDEVQSPLPIDTGKIKVVDEEDSESVKKCLMDQFSTSKSGNTDSLGKEIVLDCAKQSTMSKEIDRVHFNFLEESKLIEVTPQFYGGPKALVHCSDEDDGRLGIGGKEVNSSLSHIGSSNSFSVTNGNKENWSLSEFGTSNTFSGTYNIARRYLVLTNSTEFNTPLILKDSLHSKSSNVTNDLSTSHNDNLISTIECNKKKCKRHVDYNKSFVVGTRNLLPSFEEHGKITLPRIKLPPQPIQELFFGKGEMSKQFLHNIRTYDNMFFFTSMGGRIDNNVKKGGGPPIFCLNGQNYHLMGNLLPEEGSAPHFAQLYIYDTANELQNRINAVSQVIKEELDKHNVLVKSFRIAKTEIERNLCVEVRIKLLGKSYNLPEVSEVAALIVGDVDTNMGERDILVETHSGQLQRISELNPSYLPLQYPLLFLYGEDGYREDIGFTIKKTAIPGGRQRISPREFFCYRIHSRSSELSTLLHAKSLFQQFLVDGYTMVDPSTQGRRIILPSSFTGGARYMIQNYQDVMAICRWIGYPNLFITFTCNPKWPEVQRFLKHINLKPAGPPDVVCRIFKIKLDALITDCRKNKLFGPVVGDKFQYEERLRSLDSIISAEIPDKSSDPAYFNAIEEFMIHGPCGASRKSSPCMVNGNCSKHFPKKFVNATTFDEDGYPIYTRRDNEWCNQSRSIKYLFKYVNKGHDRVTAEFYKTSDEEESTKVIDEINMFYDCRYISPCEASWRQFSIDIQLRSPSVERLSFHLPNQQSVIFEDDDAVDNIVNRPTIAQSMFMEWFEANKNFVDARKVTYAKMPTKFVWKKDVRKWQPRQRGFAIGRIFYVPHGTGEIFYLRCLLNQVREYVDAIEEAIEWASASNLRRLFVTLLMTNSIGKPEIVWETIWGHLSKDAEYYLRKTLQKLDLVLNNSDKKIFALMEIEKILSAMGKSLSDFAPMSTPNIDMYTVATNRLIQEDLAYDCDSMKFKNQLLVKQLTREQREIYDEIIDDIEKNSGGSKGEIVLNVASSGITSLLLPSGRTAHSRFAIPISIHEDSTCNISQDLQHSAILAPTLDVVDNINQYMNDHNSGEGKTYLSCDSICKSNSNGDMLSDLHTPEFLNGLRCSGVPNHSLTLKVGSPIMLLRNIDHSLGLCNGTRLILTRLADHVLEGQIMCGTNAGTKKRGFVIEEAGVQPWSNVRSSLQILQTWLQFGEMQGNYTQDSYGSSSNHATFQHGEVISNMHCHCGESLNLRTSWTNDNPGRRAGEVGGVVSLGGMILLCVQGQRQSSPDC

>IbDExDH79

MDANKRLSYSNLFNLESLVNFQLPRDEDEDFDYYGNSSQDESRGSQGAIGERSNGMLSGRELKKKKRRTAYSSEEDENRNYSAYISEEKYRAMLGEHIHKYKRRLGTSPANIASTRTVMPSAKNSLGLKDPELKSDQRGGLLKHDSASDFLSKNNSQKLGNYLQSDVPKFVVDRSNYEPAFLDIGGGKTYSIPPPYEKLSATLNLPTVADIQVDEIYLQGTLDLETLAAMMASDNRLGPRSRGGMGDPIQQYESLQARLKSQLTSNSVPKFSLQVSEAALEASSIPGGAAGSIRRSILSEGGVLQVFYVKVLEKGDTYEIIERSLPKKPKVEKDPAVVEREEMEKIGKYWVNMVRKDIPKHHRLFTNFYKKQFTDAKRFSETCQREASATNVSAFVKLKVGRSLKVMRGAGIRTRKLTRDMLVFWKRVDKEMAEVRKREEKEAAEALKREQELREARRQQQRLNFLLSQTELYSHFMQNKSSSQPTEALNIDSGRADDQEMLLSSAEAQPGEEEDPEEAELRMEALKAAQDAVSKQKRMTSAFDNECLKLRLASETENSLPDASVTGSSNIDLLHPSTMPVASTVQTPELFKGTLKEYQLKGLQWLVNCYEQGLNGILADEMGLGKTIQAMAFLAHLAEEKNIWGPFLVVAPASVLNNWADEIGRFCPDLKTLPYWGGLQERTVLRKNINPKRLYRRSLCNHDVRMVLDTWVIVKSKLLISHHVQIELFLVRDAGFHILITSYQLLVSDEKYFRRVKWQYMVLDEAQAIKSANSIRWKTLLSFNCRNRLLLTGTPIQNNMAELWALLHFIMPTLFDSHEQFNEWFSKGIENHAEHGGTLNEHQLNRLHAILKPFMLRLVKKDVVSELTGKTEITVHCKLSSRQQAFYQAIKNKISLAELFDSSRGHLNEKKIMNLMNIVIQLRKVCNHPELFERNEGSSYFYFGQIQNSLLPPPFGELEDVYYSGGRSPVTYQGHVIIPPIVKSLNAKTGLPRNPELKFDMFYLGTRCDRLYLDEMLNLLMESEDGDLNYNHIGRDKVRAVTRMLLLPSKSDTNLFKWRSETGRGDAPFEALVMPHQDRLLSNIDLLHSIYSFIPRARSPPIHANCSDRNFAYKMVEELHNPWIKRLFVGFARTSDHNGPRKPASPHPLIQEIDSELPVSQPALQLTYKIFGSCPPMQPFDPAKMLTDSGKLQTLDILLKRLRAENHRVLLFAQMTKMLNILEDYMNYRKYRYLRLDGSSTIMDRRDMVRDFQHRSDIFVFLLSTRAGGLGINLTAADTVIFYESDWNPTLDLQAMDRAHRLGQTKEVTVYRLICKETVEEKILLRASQKNTVQQLVMTGEHVQGDLMAPEDVVSLLLDDAQLEQKLKEIPLQAKERQKKKGGTKGIRVDADGGVTLEDFADNANGFESTADPADKGKSSKKRKSTADKPAPSKSRPQKVPKNVEYSSPNSIAMDDEMDDLPNDTETRPQRPKRLKRPTKSVNENLEPAFTATPIAGQDGNQKPPLPDPSSGGWRTAVEEESSRQVN

>IbDExDH69

MKAPFPSSWRCPECVSPLNDIEKILDCETRPTIADDSDASKLGSKQIFVKQYLVKWKGFSYLHSSWVPENEFVKAYKTLPRLKTKVNNFHRQMSSMTDTEDYVAIRSEWTTVDRILACRGDGEDKEYYVKWKELQYDECSWELESDICSFQQEIERFNKIQSRRKQKSSPQDTTESKKKQKEFQQYECSPEFLSGGSLHPYQLEGLNFLRFSWSKQTHVILADEMGLGKTIQSIAFLASLFEENVSPHLVVAPLSTLRNWEREFALWAPQMNVLEVWTGEWRRLWGWLRSSRSGHQSLQDMAVTLGVVFHVLADGNGVCGGKEWDVHCFLCRVLILTRGLLEMNGHEISFHKDYEFYFPKNHKKSKKKKSGQVLGESKQDRIKFDVLLTSYEMINLDTASLKPIKWESMVVDEGHRLKNKDSKLFSSLQQYSSRHRVLLTGTPLQNNLDELFMLMHFLDAGKFGSLEEFQEEFKDISQEEQISRLHRMLAPHLLRRVKKDVMKELPPKKELILRVELSTMQKEYYKAILTRNYQILTRKGGAQISLINVVMELRKLCCHPFMLEGVEPEDNKEFHKQLLESSGKLQLLDKMMVKLKEQGHRVLIYTQFQHMLDLLEDYCNFRKWQYERIDGKVGGAERQIRIDRFNAKNSSRFCFLLSTRAGGFRN

>IbDExDH65

MGENKENDAYEEELLDYEEEDEKAPDSVAAKVNGESAKKGYIGIHSSGFRDFLLKPELLRAIVDSGFEHPSEGEFVNLLSELLWALYYLQLFFTILCLLFSVQHECIPQAILGMDVICQAKSGMGKTAVFVLSTLQQIEPIAGQVAALICHEFERFSTYLPDIKVAVFYGGVNITIHKDLLKNECPHIVVGTPGRILALAREKHLSLMNVRHFILDECDKMLDSLDMRRDVQEIFKMTPHDKQVMMFSATLSKEIRPVCKKFMQDPMEIYVDDEAKLTLHGLVQHYIKLTEMEKNRKLNDLLDALDFNQVVIFVKSVSRAAELNKLLVECNFPSICIHSGMSQEERLTRYKGFKEGHKRILVATDLVGRGIDIERVNIVINYDMPDSADTYLHRVGRAGRFGTKGLAITFVSSSSDSGVLNQVQERFEVDIKELPEQIDTSTYSMISSFRLFSAVLEKLAGILSNSAPRDTGGLWQLSALIILSLLYPLMEHSRFSRVY

>IbDExDH68

MIREDILSSFIPIKPSCASAHLSSRRHHHGKVEWTALRRVIPVGKALGSTDRQCYSLLRAAANRFSGRSAAMGMYCLANQLKAWNRRSAIKIRVMRTYCVMERRGSTQVKSREVIFHDEEGTVMHAHIPNDILPKFLNSFVEGSVYCVKNLFVVANWHTYKTSMHEYMLQFNGETIMKEYRSASFPRHMYRIRSFQSLRNNPSINDKELFDLIGRVVQIHAPQQKTINGNDARLIDFVIEDSQGSRLTCTFWDDHVSKIEPFYESPGNEPLYVLIQSCRLKFGVRDGDVKICSSYDVTQIHFNIDCPEMQQFKESMTELSQLTPMRSIASMSSMSFTNTHDDSSTQSLELITINELYDTEDFGDFWIAARVNGVERPSDWFYISCPKKGWNKALKLSEGVNKCFKCNEIPDGSVRKYKLRVRVVDMKGTASFLIWDRECVDLLGIPAEDLYERNLNNLGNIKEILELKGRTMLFKISAKKEHYVRRNIPFPVVKIKTDQLLLQQLCPDLLALDENDFNSDGQISEGDDKFLEGFESDEGESPIALLPPTSSTDCTTDGPVKRCLLDSFSSTKGGKKVKQSHDCREEVPTVKRGEEECRPLPPPDLLLFCSSPPYKSKGRGLGESHASRRWLDQIDSVAAARGGGADSEGGEEESPVIAAPDLLAALLSPPYKSKGRGGGIAVYARWFVGLIPSAAPREEVAGRKVGEEHSLAIANTAGPAGCFAPVGLLLRRSATNFIRL

>IbDExDH70

MARAHIENNPATEIKIKLIGKRSKDTRTYNLPQVQEVAALIVGDIDPNMRECDILVETNFGNLQRINELNPSYLPLQYPLLYSYGEDGYREDIQFADIVGRASTTRNRVTPREYFSFRLQDREVDPTTQGKRIILPSSFTRGARYMIQNYQDAMAICKAMGYPSLFITFTCNPKWPEIKRAKRVRVSAWAGVWKVRRRCEEESGQPGGGRHAPAHAAVSGDISGRRSQFRRRLVMLFPESKMAQERNITVISTPSKLIDIVLASDGYNFPEWRFLVKVNLDGMGKGDHLEEECPTDDSQGEWKTIDKALLSRIINCIDRRIILSMQHCQTVKEVWELVDKWFSGTSNLRKLYQLSQEVYRSNQKGRELRDYYYEFKSVCVALCAAMPITNDVNEMKKQHDKLLVFSWIAGLDKEYDVLRSQLLDNKDLESLDQVFSMLQNASGNNGSNEVQERNVLMSQGNLDPSALVSHGEGYSGFRGGGNNRFEGGALESRGGPRIVMTDEEFAKFNQMKISSSSQSPSNHVSPAATFVQTGNSVACVSSTSRQWVIDSEASEHMTGSFDEEDYWLGVQEFPARLRFGHPSLPTLKKMCPSLESLDVLQRKNRHPLGNRDASLLFQMKVSKVFWADAAHTACFFINRMPSSVLRGQVPYSLVYPSKPLFSLPPKVFGCTCFVQDVRPQKGKLDPKSLKCVFLGYSRTQKGYRCYSPDLGRYLVSADVTFFEETVFSGCISGDRSPLIEDDFLTYTITIPVPTVESVSRVVPPATQVYSRKPRDVVAPTVAQVDTPSLPEPLSNGLDLLVALRKGKRSCCHPLPSNVFYGHLSESSRTFISQIDSVSVPKTLCQAMSHDGWKFAMEEEIATLEENQTWELVQLPTGKSAIGCKWVYVVKTNPDGSVARLKARLVAKGYAQTYGVDYTETFSPVAKLTSGEEGKVCNLKKSLYGLRQSPRAWFAWFSGVVTAFGMQRSAYDHTVFYKHSELGSILLIVYVDDIVIIGDDAGIEAQIFLKEVPYKDLGVLKYFLGIEVIRSRRGIFLSQRKYVLDLLEESRLSGAKPRETPMDQSVKLIVGEGESEDPVVSQFMAFPTKSHWEAAVRVVKYLKGALVKGILCASHGHMGVEAFSIDWSPSDRRSTTGYCVFFGGNLVSWKSKKQSVVSRSSAESEYRAMTHVACEVTWLYNILGEIGVEVNKPIPLWCNNKAAIHISNNPVFHERTKHIEYYKAVEEFMMHGPCGSARKNSPCMSRSIKYLSKYVNKGNDRVAVEFYNNTVDESTGKVVDEIKMYYNCRYISPCEAGWRIFAFDIQFRNPSVEHLSFHLPNEQSIIFDDDDDNVNNVVNRPTVSQKMVWDVVWIHLAKDVEYQARKSLGIPDLVLTDTQKKNYALLEIEKLLHACNKLLGDYPPMPIPDGLTNSFFGNRLLYEELAYDQQALKEEHDILVGKLTEEQLVIYSKVMFDVDNNKVKGKIVLNVASSGIASLLLPGGRTAHSRFAIPISINEDSTCNIKQGEEEFEKLDNFARWIANLGDSKLGDNSDGELISAKDATFIRDISIDPPFHTLDFLSLGLA

>IbDExDH77

MSHLGGGAEAHARFNRFEYRANSGLVLTNDSRPRDAHEPSGEVETHPGNIDPKTFGDRVYKGKPAELEKFKKAKKKKERGSPSSESIPTGQSKRRRLQEESVLTATEEGVYQPKTNETRAAYGAMLCVIQQQLGGQPLNIVNGAVDEILGILKNENIKNPDKKKEIEKFLDPIPSNIFDELVSLGKRITDYQDGSDAVVSAAANGDEGLDDGFGVAVEFEENEEEEEGSDLDMVLEDEEEEDGLEENDAGAMQMGGGIDDDVMQEADEGMALNVQDIDAYWLQRKISQAYKQQIDPQQSLKVAEDVLEILAEGDDREVENKLLLHLGYEMFGLIKYLLRNRLKVVWCTRLARAEDQDMKKKIEEEMMDLGPDHTSILDQLHATRASAKERQKNLEKRLREEVLRLKDETSRDGDRGQRLISDRNVESGWMEGHSQLLDLDSLAFHQGGLLMANKKCELPLGSYRNHKKGYEEVCVPALKPKPLSEDETFVKISSMPEWVQPAFRGMTQLNRVQSKVYQTALFTPENILLCAPTGAGKTNVAMLTILQQIALNRNEDGSFNHSKYKIVYVAPMKALVAEVVSNLSNRLQVYGVNVKELSGDQTLTRQQIEETQIIVTTPEKWDIITRKSGDRTYTELVKLLIIDEIHLLHDNRGPVLESIVARTVRQIEMTKEHIRLVGLSATLPNYDDVAVFLRVDLQKGLFHFDNSYRPVPLAQQYIGITVKKPLQRFQLMNDVCYEKVIAVAGKHQVLIFVHSRKETAKTARVIRDTALGNDTLGKFLKEDSASREILQSHTELVKSNDLKDLLPYGFAIHHAGLVRADREIVEQLFAYGHVQVLVSTATLAWGVNLPAHTVIIKGTQIYNPEKGAWTALSPLDVMQMLGRAGRPQFDTYGEGIIITGHSELQYYLSLMNQQLPIESQFISKLADQLNAEIVLGTVQNAKEACKWLLYTYLCIRMVRNPSLYGLAADALKSDITLEERRADLVHSAATLLAKNNLIKYDRKSGYFQVTDLGRIASYYYITHGTISTYNEHLKPTMGEIELCRLFSLSEEFRHVAVRQDEKIELAKLLERVPIPVKESIEEPSAKINVLLQSYISQLKLEGLSLTSDMVFITQSAARLMRALFEIVLKRGWAQLALKALKWCKMISKRMWSVQTPLRQFHGIPNEILMKLEKKDLAWERYYDLSSQELGELIHYPKLGRTLHKYIHQFPKLNLAAHVQPITRSVLRVELTITPDFIWEDKVHGFVEPFWVIVEDCDGEYILHHEYFMLKKQYSDEDHTLNFTVQIYEPLPPQYFIRVISDRWLGSQTILPVCFHHLILPEKYPPPTELLDLQPLPVTALRNPVYEALFQEFKHFNPVQTQVFTVLYNSDDNVLVAAPTGSGKTLCAEFAILRNHQKESASIMRAVYIAPVEALAKERYSDWKRKFGDGLGMRVVELTGETATDLKLLEKGQIVISTPEKWDALSRCWKQRKHVREISLFIIDELHLIGGQGGPILEVIVSRMRYIASQLEKKIRFVALSASLANAKDLGEWIGATSHGLFNFPPAVRPVPLEIHIKSIDIANFEARMQAMTKPTYTAIVQHAKSGKPAIVYVPTRKHARLTAVDLMTYSSLDSGERPMFLSESANELEPFIEGIKEPMLKETLKYGVGYLHEGLISIDQDVVKTLFENGWIQVCVMSSSMCWGVPLSAHLVVVMGTQYYDGKENAHSDYPVTDLLQMMGHASRPLLDNSGKCVILCHAPQKDYYKKFLFEAFPVESHLQHYLHDNLNAEVVSGVIQNKQDAVDYLTWTFMYRRLTQNPNYYNLQGVSHRHLSDYLSELVENTLSDLETSKCVDVYDDLILSQNNLGMIASYYYINYTTIERFSSSLTPKTKLKGLLEILTSASEYQQLPIRPGEEELIRRLINHQRFSFENPKYSDPHVKANALLQAHFSRQVVGGNLAADQQQVLLSANRLLQALVYVVSSNGWLNLSLLTMEVTQMVTQGMWERDSMLLQLPHFTKELVKKCQENPGKSIETIFDLLEMEDDERRELLQISDSQLMDIARFCNRFPNIDLAYEVVDEVGPVFAPRYPKTKDEGWWLVVGDPKTNQLLAINRVSTLQRKSRVKLNFAAPAEAGKKTYTLYFMCDSYMGCDQEYPFTVDVKEAMAADD

>IbDExDH75

MLYRTGIVVNVVSLACALRVEFVKEVIEFTLGVARRPAFIDKIPREYFAYHLHERRMETATLFYSRRLFQQFLVDAYTMIEAEGLYDAMAICRWVGYPDLFITFTSNPRWPEVDRYLSSKGLRPEDRPDIMSRIFKMKLDGLLNDCRKKKLFGSVRAVIYTIEFQKRGLPHAHILLFLERDRAEESVDYLDSIISAEIPDKEVDPDYYAAVGEFMLHGPCGPARTSSPCMVDGKCSKSIKYLFKYVNKGKDRVTAEFYKSSVDEETGTVIDEINMYNDCRYVSPCEASWRLFGYEIQFKNPAVERLSFHLPNQQAFVFEDDEPIDDILNRPTVSHSMFVEWFEANKKFPEARLLTYAEMPTKFVWKKDTRQWHPRKRGLRLGGYSMSHQSPGFYALRKLFVTLLTSNSIAKPELVWHAVWHHLSEDAQVNARKQLRNQDLFLTDAKKKNYALQEIQKLLSIWGKSLQDYPDMPMPDNEVAMLTSNRLIHEERSYDCAEQLLENERLVGLLTDEQSLVYTSIIKDVEENSGGLFFVYGYGGTGKTFLWRALSSHFRSRSEIVLNVASSGIASLLLPGGRTAHSRFAIPIAINEDSTCNIKQGSPLAQLIVECKLIIWDEAPMMHKHCFEALDKTMRDLLRFKNPCSLDMTFGGATGYRELEDFATWIAAVGDGDIGKASDVDFEISIPQKHLLDSGDDPIATIVENTFPMFRTTPNEEHFLENRAILAPTLDVVDNINEYMNNLNEAEGRTYLSCDSVCKSDANIDMLSDLHTPEFLNGIKCSGVPNHSLTLKVGSPGMLLRNIDHSLGLCNGTRLIITRLADSVIEAKIMCGPHVGTKVLVPRMSLTPSDARLPFKFQRKQFPLMLSYAMTINKSQGQTLSHVGLFLRKPVFNHADDQLDFDDEEYGGGQKMQYHGGGTIPALAEEEMMGEDDEYDDLYNDVNVGEGFLQLQRSEAPKPPDTVVNGGSQAPENNMSEPRAGAITSQEMNPGVASSGLRFPEQKSGLTAERGPNQAADAPEKARPPSMTRDPQVGNMGFQGSVPMSHRTGSDPADISGNAVNESMPLQNSAAGGSRGAPLMPAHQMNSNANINMNSSMMNENQMRPAIENGNTMLFVGELHWWTTDADLESVLIQYGKVKEIKFFDERASGKSKGYCQVEFYDPSAAAACKEGMNGHLFNGRACVVAFASPQTIKQMGVSYMNKTQNQVQAQPRRPMNEGLNRGSGTSFPSGDQGRNFGRGGWGRGGQGMPNRGPGGGPARGRGAMGAKNMVGNVPGANANMSGGAYGQGIAGPGFGGPPGLMHPQGMMGPGFDPGYMGRGAGYGGFSGPGFPGMLPPFPAVNPMGLAGVAPHVNPAFFGRGMAANGMGMMGTTGMDGPHPGMWTDTSTGGWGGEEHERRTRESSYGGEDNASEYGYGEASHDKGARSTAASREKERASERDWSGNSEKRHRDDREYERDRYDREHRYRDERDGYRDYRHKERELDYEDDYDRGHSSRSRSRSRAVQEEDHRSRSRDTDYGKRKRLPSE

>IbDExDH78

MATKKERIFHGAIVLFIHKSYRDACYEYGLLNDDKEYIDGITDSSYWASASALRRLFATPLSSSTISRREVVWDAVWEFLAEDAQFHHRRRMNNPDKKQYLSCGVGEIVIFVGEESERLSKMPLPDETNIGFMENMLIAEELAYDKESLKIEHETLVTQLTDEQKNVYDSVMNDIDCNGGGLFFVYGYGGTGKTFVWRTLSSMIRSRGDIVLNVASSSDLAELIIRSKLIIWDEAPMTHKHCFEALDKTMRDLLRFTIPGIRAHQELKATGLASEEDKHTVDWFSKWIANIGDGITGVVNNGLSEIDIPPRFLLNCGHDPIATIVESKFPSARYGMIDELDLEGRAILSPTLDVVDQINQYMCNMNTAEAPHCHKIDGSYCGSKIVNGTHQRTKVLIANVSHPIDHEIALQVPRKQFPLMLAYAMTINKVIIETLTHVGLLLKNGL

>IbDExDH80

MMDLIWRCVEIFQVVLCCGSGKEDGAETEWGQHYSYSQVINNYDSLSEGTVTSAPSTKLPFTTQPNVTPTANENRLRWQCGFQAELEIESATDIDYIKKKQKTLAEFPASLSSTKLSSSSLSSSSVKPPPATPSPAFSQKRSQVSQQPYSSLTSSHKVDIVPQVLKKPLSISTYKDYFRALLYAEDCYLEGNARYDRKNLNLEERNKWDHFEMKNVTLGLHYAATYKRSRLNSLDEDDQKGEKTFVAFEIDKVPEKRPFLLSRDFVSLRPSKKNFLLFEGLVYRVVKSNLLLAEFGEDFHSQHCPDYKYDVKFSFNRVCLKRAHQAIESASGPLFRNFLFPEFLPENRLLSKHSFAYKTLDSEQSSALDKILRLQASPPYLVKGPISVTKTGNLIVAAVVELCRASPLNRVLLCAPSNKTCDVILRALKKQVPEHDMFRANAAFRERDGVPVDILPSCLYEDETECFSCPLLNELCRYKVILSTFMSSYRLHNEGIRAGHFSHIILVDASSATEPETLVPLANFATEGTVTLVTGEPSNHSGWVRSPMARKFGLARSYFERLCGSKLYMSLDPNAITVLRDKYQSC

>IbDExDH66

MSHFCHEVGLRSSIRRLFYVPPGCGELYYLRCLLNLERGPFSHEDIRTVAGVIHNLFRDACYEYGLLDDDKEYIDGIIDSSYWASAYALRRLFVTLLTSSSLRKPKVVWNAVWDFLAKDAQVQRQRVLQNPDKKQFALMELENLLSSWGKSLKDFPEMPMPDESSMVLSENMLIAKELPYDKESLKTSMRHCGIASLLLPGGRTAHSRFAIPLSLNEDSTCNISQGSDLAELIIRSKLIIWDETPMTHKYCFEALDKTMRDILRFAILGSAEKTFGGKIVVLGGDFRHVLPVILKATRPEVVGVTINSSYLWTNCKVLRLTKNLRLRTLASEEDRHTVDWFSKWIAYIGDGIAGVVNNGSSNIDISARFLLKCGHDPIATIVESTFPSSRYGMLDESQLEGRAILSPTLDVVNQINQYMCDMNTAEGRTYLSCDSLCKAESGGENLSEVHTLETRLIITRLADHIVEARIVNGTHEGTKVLILRMSPSPSDTRLPFKFQPKQFSLMLAYAMTINKSQCQTLTHVGLLLKKPVFSHGQLYVALSRVSHPNGLKVLAVDEDGQDCVATSNVVYKEGCRGKVRRSIGTMGLRLCHWEWIESAPKKWAGQETIWPHDPHTGWSYCVTIPSWVVLAKSRDSDPVVSNINTLKGQFYRVVVGLQSPDGITSTRIVLRRFNDFLKFHAALKKIFPKKNLPPAPPKGFSRLKTKVLLEERRSSLEEWMTKLLSDIDLSRSVATASFLELEAAARSSFQDEQQLTSEPRSPVNNTASSLEVHPSAGLSIVAGSSSLASDYGSDTAYEASDVDSTSHGRGNPSELGTEDLSLDEDLTSPIDKFMKYGMSNIDEGLFMGQAILEQLGSFPRHKTHAKEISNVMEESMSNGSAMKSSYISGDTTAHFSEQDHSHIIHHARKFSAESVGSDIASQRGSELANSSFPNSFGDGCAEIPKASKASGNTEILGKDLTLPDHIQLVLPSDQRHKMHRVLTTMQRRLVTAKTDMEDLISRLNQEIAVKDYLSTKVKDLEVELEATKEKSKENLEQAILVERERVTQMQWDMEDLRRKSMEMEHKLNSQQGEINKDSSITNLNQEKDALQQELTATKLQFEELLKRHQELEVKSRADIKVLIKEVKTLRSSQSELKHQLSESFKEKAETERRHAQERQISDRSTAWWKLLHDCEILQQRVEECKINLADEEENLIKKFQSLPDAMHLLTTSDNQINLLLTEVQQLSQGANSVSVDDTEDNVDTDTMEIIEKMRKMLRNVFINNGKLRKQVNSVLRCALETKMSFQSVTKGPQGEGENVQHDELDE

>IbDExDH74

MSSTLLKKLNHELTSYPICFSSSGYKVSLARVLLKLLKSLKGFHWLPCDLSTVVEKVMDNLLQALTLRCFLSGEARTPTEQKPRHERIRNAFQDRVSLGMHLKIFAFTSLSYFLIPCGPRSPNSRFTYLPACFTFWRQCELMQASSSVANIDEWKWKLSLLSRSEDQEVVSRDKRDRRDYEQISNLAKRMGLSSEIYGKVVVISKVPLPNYRPDLDDKRPQREIRSINPVNFTEFIYGCAHVQVVIPLSLQRRVEGLLQEHLDRIHLSSEKSDGMPSESLPADKVENVTMDENPDSFLDDSVMEKVLQRLSLRMRNLQRTWQVVVISGETGCGKTTQLPRYILESEIESGRGAFCSIICKQPRRISAMAVAERVATERGEPLGESHRSSEFQIFFSWLIISNLKISLFSCCPSQKIFDSDRNLDGITHVFVDEIHERGMNEDFLLIVLKDLLPRRRDLRLILMSATLNRFIFGYFGGAPTIHIPGFTYPVRAHFLEDVLETTGYKLTSFNQIDDYGQDKMMLSIDQTLRTTRLIGTDSLASWSPIRSCLRDQLKAHPLLGDPNRVLVLTCHGSMATSEQKLIFERPPQNVRKIVLATNMAEASITINDVVFVVDCGKAKETTYDALNNTPCLLPSWISQASARQRRGRAGRVQSGECYHLYPQCVYEAFAEYQLPELLRTPLNSLCLQIKSLQVGSIAEFLSAALQPPEPLAVQNAIGFLKMIGALDEHENLTYLGKYLSVLPVDPKLGKMLVMGAIFRCFDPILTIVAGLSVRDPFLLPQDKKDLAGTAKSRFSAKDYSDHMALVRAYEGWKDAEREGSAYEYCWRNFLSAQTLQAIHSLRKQFSFILKDAGLLEADNATNNKLSHNQSFVRAVICSGLYPGITSVVHRETSMSFKTMGDGQVLLYANSVNARYQTIPYPWLVFGEKVKVNTVFIRDSTGVSDSILILFGGNLGNGATAGHLKMLDGYLEFFMDPSLAECYMNLKEQLDELVQKKLEDPGKDMHKEGKYLMLAVQELVAGDQCEGRFVFGRESKKPKESSNNDRFTKDGTNPKSLLQTLLMRAGHHPPKYKTKHLKTNEFRALVEFKGMQFVGKPKRNKALAEKDAAIEALAWLTHTSDKKGEEDDNSPPDVTDNMLKLLGKRRRSKRRSS

>IbDExDH88

MPIPYEANMCLMENMLIAEELTYDKESLKIEHEKLFFVYGYGGTCKTLVWRTLSSKIRSNNDVVLNVASSRITSLLLPRGSDLAELIIRSKLIIWDEAPMTHKHCFEVLDKTMRDLLRFAIWGSAQKTCSGKIVVLDGDFKQILPVILEATRPVVVGTTINSSYLWTNCKVLRLTNNLRLRSLASAEDRQTVGGFSKWIANIGDGTAGVVYNGLFQINIAAQFLLKCGPDPIATIVKRTFPSTRYVMLDESHLEGRTILSPTLDVVDQINQYITLTTLLVFATTIMSMLISSLPEEVMHLAVGHQTSKEIWDAVEIAVPSSSQAHALNLLGLNFAHRLPLLLFMLLLLSSVVVAKIIALGRTEVVAHFEVSSSPRVITVVVNAVEVRGAANHATPYISALSTFEEYNGNDTLRVGDGMSLPISRVGYTSFTSPTRDIVTKTFLFGVVVWVSSIRCRCLIHLHLLFFLHERLLLSGIVVLGILISVFRVVSCSLVRQSCAYTHEHNGRVECKHRHVVETGLTLMAHGSVPSRFWDFAFESAVYLINKLPTPTLGHLSLLGHYHGLSLLFRTHAMVTRSMSRAGVGSIALTTEVCPSKPTCYTQAVRFAEWRVAMDLEFNALLQNQSRQLVLYRPGMNVIGWVHLLFLGNWSLREGYCGHFGAKKETFSRINTSMTRCMLVQASMLRKVESCTSKNHTRARFYSRKGRKLHEQNHARASTSVQEAEHLGETRTKRATLVQAVILVGSGFCCCPCALNACFALEKDEGKVFNEMPWGIKVATKVELESFGRPRTGMALAVDATFPRIKIRACHDLHY

>IbDExDH91

MGDLDILIQTRTGQLKRINQLNPAYLSLQYPLLFPYGEDGYREDISFSDAWHQRHHGGRKRISPKEQIGYPHLFITFTCNPKWPEIERYVAHRGLKPEDRPDIICRVFKMKLDAMIEDIKTQKLFGDICGVIYTIEFGKRGLPHAPILLFAKTINRANSAAEIDAIISAEIWKFDTDEESDIGHGEFMSSYTNGHNDEVVDEINIYYDCRYISACEATWRTFGYRDTLSDATAVERLNFHLEHQQNVVYGEDQTLDEIVENQTVKRANSRIWFEANKKSRKCVLSHASSQAGGAYGILFGISSKMQFHHRRRMNNPVETFEMPLPDETNIGFMENMLILKSWYDKESLKIEHETLITQLTDEQKNVYDSVMNDIDCNGGGLFFVYGSEERQDITVVLKRHLVESGSFGGDFRQILPSYPQRERPIVVGATINSSYLWTNWVVNNGLPEIDIPPRFLLNCGHDPIATIVESTFPSARYSMIDELDLEGRAILSPTLDVVDQINQYMCNMNTKVELI

>IbDExDH89

MILKARINCRHHRQLPTLTSGNGNLAYYRAVRNKKLYLEIKGIEETMSKYLTSPKGWAYTGMLLYCVASRYRHVTENLTHFSLFPEIYGKVVVISKVPLRTTHCILMISGLKGRSINPVNFTEFIYGACPGCHPTKFAKESGGLASGTPGRNSSRRSRWFFKGEACECAVCKELGRLVLAFMVAYIKYPQALTVHLDLHPDPFFSTYPTLSVLKQVVVISGETGCGKTTQLPQYILESEIESGRGAFCSIICTQPRRISAMAVAERVATERGEPLGESGMKGKNTHLLFCTSGILLRRLLSDRNLDGITHVFVDEIHERGMNEDFLLIVLKDLLPRRRDLRLILMSATLNADLFSGYFGGAPTIHIPGFTYPVRAHFLEDCQGTDSLASWSPDSVGFNLIEAVLCHICRKERPGAVLVFMTGWEDISCLRDQLKAHPLLGDPIRVLVLTCHGSMATSIVKPLFSSFVRF

>IbDExDH97

MMDSMELGDSPRSNSETTGSDVAIDHHGVFTASASTDENVNMSISAEKLVRSFHTSTKLPSGAPAELPRKLNGSACEACGRPEAEGCVVVCDGCERGFHVGCLGMLVGEAIELEEWICGKCSGCGVRSNRWNLGWRNKRRRVDTDSGLDITGTPRSEGEGEGCKDSLLFRKHIPGDNPFGGNLVGLPVEVTNLQHFNNSFGSQEATDTVKLQFVPPSLGAVDSCLKGLNGENNSTTMGSSTKDLNEMYFLALKEYLKEEHVLVEGWRVEVEHNSATRELCPVYVTPDGKRLGSVSEVASYLKSITTNMPTQCGTGKYDDGLPIQYGDFFVLSVGEVDARHSYHCRNQIWPVGYSSCWHDKVTGSLFMCHVQDGGDSGPLFKVKRSPCSALHIPNPLQVLYQAKHGHSNGQNERSDVLASQSMDYEDLSIMSMLAEPAAPLENDILSCLVHSSDDYLDMQGSHDMLTEKSHSKRNQSLLSDCSPFGHDADIFSVEESSSSLAWRVMSRKILKLFREIYTQTGVVKFFCKHILDKSESFCCDVTNEGAAEKYAPLARFYGLPTIVNIPCIIQADMQLEVVAQELLKWLDQDRFGLDAEFVQEIIEQKQDVRDSFGYVPLNQRSSFSSFITVGNSLLEGRAVEVESEDGRCPSHGLRSSNKVNTMTEEHLSDTRSLPPGIPFSTKMPTLLLGDTIQVLQFFWRFHEVLDLKKLLTFEDIEEELINPRCHGVDLLEKVGGEIHEKPFVIPNNTDGAKMHISSYKSGPEVDLQNLHAFVQMERKSTEEASPMKLEFINSSKCSGSALTKAIISTLHVLLSELQTKVAVVGNLNFDIGDSRKRGRKKDFDYVSIAKRNTLSMLPYNELTWPELARRYALALLSMNGYFESTEITTHDNSAQVIHCLQGDGGALCGSLTGVAGIEVDASRLKREFFGSLDGDERDNLAVDDEDSDRKSLDESVTVSESIPDWIQVLEPVRKLPTNVGARIKKCIYNALERSPPEWARKILEHSISKEVYKGNASGPTKRAVLSVLAQAQDENCRLVLRHAIAADHRKVFCNLLGKRTMNYNTDGGIIGTPGMTSRPLDFRTIDQRLAVNAYGGVHKAFYEDACEVWSNVFIAFKHQPDLLQLAESLANNFKSLYDKKVATLSQKLVKCRKLNSVDASLQKEIENILTCSEIPKAPWEDGVCKVCGIDNDDKSVLLCDACDAEYHTYCLNPPLARIPSGNWYCPSCVTNKQMVSDAYTETAVVCPQKKKDHSKLTLAYIEAVAHLATVLETKEYWEISVAERTNLLKFLCDELLNSALLHGHLEQSAEDSVELQQKWRSLLKELKVLTLRENFLARKADDGSLTVGEHDVEVQESNETLTGNEKCSGYLQTTSHSGNHISVSNDDVPSSGDGQKCVDPNAFFKHLPADCLENSDSVDCQSFEPMDADDAEGVSPITGDGQLNESSLVNTTSSFGQNMDLLSCEETKGLKGDATYEDNFEKHTGRNMNIRNSEGVDGHHASVDKAATDLNAHLFTTSTGTVQAFNLSLKSVRDEILLMQNSITSLQKQLQKVSLRSEFLGFDSAGRLYWVVSMSDAKPCVIVNENVELHQGEKITSGSNASSPPFWCKAYDGNSVQSSWISYGSDSEIDLLLEFLKDDDPLERQLKQSILHWQKQRYNLPQLSELSLPHEVLDANLATRATGLLEAKCGSSIEAEATEFFKKQEKGGIAFVRDGIIYRCKCLEPIFSFERHYKYCRQTLSAPGELRFHGQVTCKQREKPVEVDNTSKQKGMVKPTSKDEYKCEVGSVDQTDGLECPFNLEDICSKFVIKETMKEEAEKIGLLGSSNGLPTFVPSLPPYLTDPASMLLQVQDNTSSLDHNAADTLTSSSKHAPKPTVHDASQNLPTTNGLEERDTLKHDNCLEHPLVSQSALKPLVGEASRILRRLKINLLDMDAALPEQALRPSMSQIERRLAWRAFVKSAETIYQMVQALIVFEEMLKSDYLSNTWGYWSSISGAAKICTLSSLALRIYSLDAAIKYEEEVAAIEKTKVGRKVSKKASSSFAQ

>IbDExDH96

MEIAKKKQRPGQGLIDAVFSWSLEDVMNKNLYRDKVKEIPDTFMSTDHYLKSFINPLLKETHADLYSNMKNSLRNAPSREVLEVKISKDFKAPKDMLYNILLKTTREGDGESKQPYEPEAGDLIAFCDVKPKRVEDLNRPKMSYAIAVVQGRKDDGSARFPILSSKPVTFRKKMERGREGDKLFVVYLTSLTTNIRIWKSLNMDKDSANLKIIRTVLQIDPKDEGDCALCTYGGTQATTLLNAKAAIQSFGLDNSQEEAVLSCVKARKCVHRSSVKLIWGPPGTGKTKTVASLLSVLFNMKCRTLTCAPTNVAVIGVAKRLMELVRGSLQYDSYGLGDIVVFGNGERMKVNDHEDLLDVFLDYRVDALASCLSPLVGWQAGLNWMINLLEEPEEQYQKYLDKIKDENEESDDELETGSSSGELSSTEEKDGLITDKYLNKTKGNNSNMHLKKFIVQTIKENKKKKLKDESSQKKSIDKVAKCHRGKDRIGEVTIWTFEEFVLKKYKSLAEQLDFCMTTLYTHLPTSYIPQEVAKKMVRALNLLQTLGGLLKTVAEIPGGLRDGLKGIKVSLSKWKKRINELQITKSKCVVILKLLRGSIKLPNFSENYQIRNFCLKFAVLLFCTVSSSSKLHSEGMAPIELLVIDEAAQLKECESTIPLQLPGLCNAILIGDEKQLPAMVQSKICEKAGFGRSLFERLVKLGHKKHLLNIQYRMHPSISLFPNRQFYEGKVMNGPNVKSIEYEKRFLKGNMFGPYSFINISQGKEELDEKCSSKNMAEVSAVAEIIAMLYTEFLRSKQKIRVGCISPYKAQVFAIQEKLGKKYSTDVESDFSVNVRSVDGFQGGEEDVIIISTVRSNGNGSVGFLSNFQRTNVALTRAKYCLWVLGNGATLINSGSVWGDLVQYSKARGCYYDACSDKNLEKVIADSSDELTTKLSAMSLSNKPGSSFKASGKVKNFKKNKAWFSSNGGRG

>IbDExDH86

MVMLSSSFYVSLLLAIVHTLQNKMSMLYVLAKDISPQSYKKAIRLRLIRTYIVTEGRNKSVKSQECVFRDIEGTYVHASIHADFIDQFSHLLKEGKVYAGKNFVAVSYYYQYKTTQHKYMMRFNQYTTIERHRRKGFPSLLFRIKPIEELLAGKVEEKLLIDVIGRVVEFYSPKDKVIAGFPTQLVDFLIEDSKGNRIKCTLWDEHVTAVMPFFNNHSDGHLIVFLQLCRAKTVDNEVRISSSYTATKVLFNLDCQEFADFRNSLKANCSPLRSISVVSYGHSANNSQSPMVVSTIRDLYERAESKMQVPVEFEQLRNKSMVFRINLKNEHIRNPAKPISVLSVSHNEELEAQYCPSMLDDQDELSRMIEEDADDLESEESESGDEAISPIVQANLKLKGVANDQDEVDTGAIKRCLLDQFSSSQNLKKCRPLVIKEERTL

>IbDExDH95

MFCFTSMGGKIDNSINQGSAAPVFRMYGHNFHLIGSLLPPDGIKAKFAQLYIHDNENEINNMICSVRKRDEKHTIHQDIVADIKDVLDNHNVLVKSFRNAKDVIETNPRVEIKMRHIGKRAKDARTYNLPSTSEVAALIVGDLDLSIGNRDIVVESKSGRLKRIIDAYTMVESGRLIYIRTNQKTLRCEAYKGLSDALTRGEVDPSTQGKRVILPSSFTGCARYMIQNYQDAMAICRWIGRTVMKNGIQLDNRYVVPHNRALLLKYRAHMNVEWCNQSRDACYARGLLDDDKEYIDAFEEASHWSSAQSMRKLFVTLLTTNSMNRPEVVWGATFVWKALSATLRSKGEIVLNVASSGIASLLLPGGRTAHSRQILPIVPKGTRQDIVAATINSSYLWDNCKVLKLTKNLRLNTIGDAAEFEKLDVFAKWIASIGDGTIGEQEDGFPEIDIPTEHSRTYLSCDTVCNSESTNGILADVHTPEFLNGIRASGIPNHSLTLKVGSPIMLLRNIDHSLGLCNGTRLIVTQLSEHVIEAKISTGDHSGTRVLVPRMTMTPSDPRLPFKFREDNFGDVVIC

>IbDExDH81

MASTLRRPPPVFAPLLRPHPSPSPPSPHTFSTLYIYDLPRVFNADLVENCGELDPWHSRCQVLSNDGFGLPAAGISKILPENLAGSWFWTDQFALELIFHNRMANYKCRTTEPESATAFYIPFYAGLAVGKYLWPIKVNYTNEDRDRDCKMMLKWIQDQPYWNRSNGGDHFITMGRITWDFRRKEGMNWGSSCIFLPGMHNITRLLIEAHPWDYYDVAVPYPTGFHPTTAGDITRWQQFLRTRQRNTLFCFAGAPRRLIKNDFRALLLTQCHHSGACRAVDCGGAKCSNGTSEILETFLDSDFCLQPRGDSLTRRSTFDCMVAGSIPVFFWKRSAYYQYRWFFPPETESYSVFIHRDEVKNGTSIKSVLEKISKEKGELVKVVSQSDGEHGLSRGICIQRLVLEPTWSKLIRRIQNQTTEALDFEKKEKKSEEDKGNSEYDSERPSKKRKALEIDSIEEVRSATRPMNFKVDKKKKKTKFTEVVDNGVETEENPSSISNFRISDPLREALKVKGIEYLFPIQATTFDIILDGSDLVGRARTGQGKTLAFILPILESLRNGPATATRKTGYGRAPSVLVLLPTRELALQVFADFEFYGGALGLTSCCLYGSSPYAPQQTKLKRGVDIVVGAPGRIKDLIQKGNIDLASLKFRVLDEVDEMLRIGFVEDVEFILVLWALKFVGTVDIHFRKVEDAGQVQTLLFSATLPDWVKHISAKFLKPDKRTIDIVGNEKMKASTSVRHIIIPCSTSARSQLIPDIIRCYSSGGRTIIFTETKDYASELAGSLPGARPLHGDIQQAQREVTLSGFRSGKFMTLVATNVAARGLDIDVMLVIQCEPPRDVEDYIHRSGRTGRAGNSGVAVMFYDPKKSNISKIEKESGVKFEHIAAPQPADIAKIVGKEAAEEIAGISDSVIPAFKAAAENLLQTSHLSAAELLAKALAKASGYTEMKRRSLLTSMENYVTLHLVSGRPVYSPSFVYNALKRFLHEAMAESIKGLTLTADGRGAVFDVSAEDVETFIAGTVCVSVFDLKCARNEFDISLEVVKALPQLQERDKSRGGRFGGGRGGGGGGGGGFSDRRGGGGGRFSGGGGRGGRGGFSDRQNDRIGRGKGRSKIGLSQLRRQCFQARLQAKFVYVQRHDQSSCLQNTDPTSCQALVLYKRLLFDEFTPDFITLPFVLKECVNRVDAFAGLSVHGHAVKFGLHSDIFIQNSLISMYSGCGLLDNARRVFDEMPNRDVVSWNSMVVGCLRNGELDLALELFRNMKKRNIITWNSIITGFVQGGRGKEALGLFHEMQISGGDDVISPDKITIANVISACASLGAIDHGRWVHSFLKRSKIECDTVIATALVDMYGKCGCVDKALEVFTAMPKKDVLAWTSMISVFSLHGNANQAFELFSEMEAAAVKPNSVTFTALLSVCAHSGLVEKGRWFFNAMRSVYSLEPQLQHYACMVDLLGRAGLFDEAEVLIRSMPMEPDVFVWGALLGGCQMHRNFQLGEKVAQYLIALEPQNHAFYVNLCDIYAKAGKFDHVKKVRAFMNSKGIAKTAPGCSMIEIDGVVHEFSVRGSPEDLLQEIKPLLDALSYEMKRESHIEVNHDLCLTVYR

>IbDExDH93

MPKRGERRIPDLNEDSTCNISQGSDLAELIIRSKLIIWDEAPMTHKYCFEALDKTMRDILRFAIPGSAEKTFGGKIVVLGGDFRQILPVIPKATRPEVVGATINSSYLWTNCKVLRLTKNLRLRTLASEEDRHTVDWFSKWIADIGDGIAGVLNKVNLISIFRQGGENLSEVHTPEFLNSLIFSGLPNHSLTLKGAPVMLLRNIDHSLGVCNGMRLIITRLADHIVEARIVNGTHEGTKVLIPRMSLSPSDTRLPFKFQRKQFSLRLAYAMTINKSQCQTLTHVGLLLKKPVFSHDQLYVTFSRVSDPNGLKVLAVDEDEQDCVATSNVVYKEVFNNV

>IbDExDH87

MKITLKFKAICKCKDEDCIGKDEYYLAMICAINAEMMKIALKFKAICKNEPLSNDNNTQASLLQSNILFDNAQSSLQSVLGSPAFTPVNPIVNVVGSERLYGGFMMCSPRKPINMSTNVNVHPTPFEDNYVTTRGTPLSATTNVTSNPNSYALNVEIPPVTALSVVTDGGRVDRSINTRNAPPVFRISGQNYHCIRSLVPGEGSTPKFAQLYIYDTDNEINNRINSVRQDNNSGDIREDIVEKFKNMLDQNNVLVKCFRMARTEIQSNPVVEVKMNLIGRRSKDGRTYNLPTANEVAALIVGDLDPSMGDLNILIQSRTGQLKRINQLNPAYLPLQYPLLFPYGEDGYREEILFSDAWHQRHHGGRKRISPKEYFSFYIHERKALRCEAYQGLSDALTRGELDPTGRGKRIILPSSFTGGARSIKYLFKYANKGNDRVTAEFMSSSTNGHNDEVVDEINMYYDCRYISACEATWRLFGYAIHYRTPPGERLNFHLEHQRMLSMVKIRHSMRLWRTKRLSERFTAWFEANKKYEDDVLSLMHSSQGKSLRDFPEMPLPDETNVGFMENILIAEELAYDKESLKIEHETLVDSSLCMVTEEGRHSCGGRVILNDKERGDIVLNVASSGIASLLLGRKGDSRFAIPLSLNEDSASNISQGFGQNHEGSLRFVIPGSAQKTFGGKTVVLGGDFRQILPVIPKATRPIVVGATINSSYLWTNCRFLLKRGHDPIATIVESTFPSARYGMIDELDLEGRAILSPTLDVNIDHSLGLCNGTCLIVTRLTDHIVEAKIVNGTHQRTKVLIAECLTPSIEIALQLQPSPNHWRSSQSVMASHAAVARSSSYASATATRLFNFNTRTFPKTQSSILMQLRSSRKSKNIRSESISSASSGSIAVRGESFRVNANAGGGNGSTAEANGIDGSKPVTFKWPDNKRPRVCILGGGFGGLYTALRLESLVWSDDKKPQVILVDQSERFVFKPMLYELLSGGISRHVEIAPLFSQLLANTSVHFFKDRVKCLYPRDHLLTNVPAPSDPGGTVHLESGLAIEYDWLVLALGAEARLDVVPGAAEYALPFYTLEDALSADEKLRDLERKNFGKDSPISVTVVGCGYGGVELAATISERLRDRGIVQAINVEKTILPSAPEGNRETALRVLSSRKVQLLLGYFVRCIRKAVKGEVDHDDPANTVSVHATTAHHSESVILELQPSERGLQGQIIESDLVLWTVGSKPLLPELEHGDYPYELPLNGRGQAETDETLQVKGHPRIFAVGDASALRDKNGKLLPATAQVAFQQSDFAGWNLWAAINGRPLLPFRFQNLGEMMTLGKYDAAVSPSFVDGVTLDGVVGHTARKIAYLIRLPTNEHRVKVGVSWLAKSTVDSVASLQNMLSKVLSGN

>IbDExDH92

MKDPPPVLQSLYFGTEQSTHFLNNIRRYNNMFCFTSLGGRVDRSINIGNAPPVFRISGQNYHCIGSLVPGEGSTPKFAQLYIYDIDNEINNRINSVTRSKDGRTYNLPSANEVAALIVGDLDPSIGDLDILIESRIGQLKRINQLNHVYLPIQYPLLFPYGEDGYREDISFSDAWRQGHHGGRKRISPKEWSQVYDSELSRCNGNMQTIGYPHLFITFTCNPKWPEIERANSVSEIDAIISTEIPGPDADTVYHDVVGEFMLHGPCGQLLRITYISGCEATWRLFGYAIHYRTPPMERLNFHLEHQQNVIYGEDQTLDEIVENQTVKQSQFTAWFEAKKKYEDARSLTYAEFPRCGELYYLRCLLNLIRGPSSHDDIRTVAGVIHKSYRDACYEYGLLDDDKEYIDDITDSSYWASASALRRLFATLLSSSTISRPEVVWDAVWEFLAEDAQFHHRRCMNNLDFDKKQFALVELEKLLSLWGKSLRDFPDMPLPDETNIGFMENMLIAEELAYDKESLKIEHETLVTQLTDEQKNVYDSVMNDIDCNGGGLFFVYGYGGTGKTFVWRTLSSMIRSRGDIVLNVASSGIASLLLPGGRTTHSRFAIPLSLNEDSTCNISQGSDLAKLIIKSKLIIWDEAPMTHKHCFEALDKTMRDLLRFAIPGSAEKTFGGKTVVLGNDFRQILPVIPKATRPIVVGATINSSNLWTNCKVLSLTKNLRLWSLASEEDRKTVDWFSKWIANIGDGITWVVNNGLSKIDIPPRFLLNCGHDPIATIVESTFPSARYGMIDELDLEGRAILSPTLDVVDEINQYMCNMNTAKGRTYLSCDSLCKAE

>IbDExDH83

MKRRSIHQNEHPFDVYPFEAFRNGSWQPVERIRIQHGTFAMHVLDEGDLIEEEISISNVRIRSRNATSSDCACFLRPGLDISVFSTPHQSEDSDEENDGDEKMEPEPAWIDAKIRSIERKPHGFGCGCQFHISVYVTQGPPPIVKKTLSKEIKVVQLNQIAILQKIEQKPCEDKYYRWSLSDDCSTLQKFKLFTGKFCSDLSWLLVASAHKQVTFDVRSVHNRIVYEILKDENDIDATNSEYHSYAVNFKLENEVSTPIIVQFNPDIPERGPEGDVYEAGPLVVYDELRRSKRRFVQPERYLGCDDYLTEFDVEMTRLVGGKMYKSELEELPMALSIQADHAYQNGDIDKTLACYQQELRDNSLLSRNRNRSDAHTERKRVSGDKTSRQPHLAMVPLQLSAENNLFDQKENPLSFEDNEDLSAEIREIVSKYIYVNGSSAVEMKKSSLNLMQGRRWGQVKVSKLKFMGLDVKGGGIGSSKKARKKRNCHPSVRDSIYDIRSFRKGSISANVYRELIRRCMANIDETLNKEQPPIIDQWKEFQNTKSCQREPIEKASTNNEEELSELDMLWKEMELALASCYLLDDSEGTNLKSATELHMATKKGTRVCHHDFRLNEEIGIVCRLCGVVSTEIKDVPPPFMPATCSGSGKEHRPEEVVMESKQGDDADLDHFTIPVSSNKPSSEGEVEDNVWALIPDLRNKLRAHQRRAFEFLWKNIAGSIIPTQIDPESKKRGGCVISHTPGAGKTLLIIAFLVSYLKLFPGSRPLVLAPKTTLYTWYKEIIKWKIPIPVYQIHGGQTYKGEVLRQKMKLCPGLPRNQDVMHVLDCLEKMQKWLAHPSVLLMGYTSFLTLTREDSTYAHRKYMNQVLRQCPGILILDEGHNPRSTKSRLRKALMKVNTSLRILLSGTLFQNNFGEYFNTLCLARPRFVDEVLKELDPKYKKRVKGAKTRFSLENRARKMFIDKISKKIDSNKQRERKEGLNILKKLTTGFIDVYEGGSSDNLPGLQCYTLMMKSTTLQQEILVKLQDQRPIYKGFPLELELLITLGAIHPWLIRTTACSGQYFKEEELEALEKFKFDLKLGSKVKFVMSPDPSLSPPQRESLDLLPQHSSHQSVSRDIREELGGPSKVMLASITTCAEGISLTAASRVILLDSEWNPSKSKQAIARAFRPGQDKVVYVYQLLATGTLEEEKYKRTTWKEWVSSMIFSEDLVEDPSHWQAPKIEDELLGEIVEEDRATLFHMIMKNEKASNMGRLQV

>IbDExDH94

MAKSEIESNPRVEVKMRLIGKRNPDARRYNLPTVSEVAALIVGDLDPMLGHRDILVEGKSGQLKRINELNPSYLPLQYPILFPYGEDGFREDIQFSIPETSSNVRRRTISQREFFAFRIHERLNELNTLLFSKRLFQQFLVDAYTMVESSRLTYIRLNQKSLRCEAYKGLADALTRGEVDPKTQGKRIVLPSKFPGGARYMIQNYQDAMAICRWIGYPNLFITFTCNPKWPEIQRYIHGRGLKAEDRRDIVCRIFKLKLDALIREIRSGELFGEVSSAEIPDKDNDIKYYNAFEEFMIHGPCGAANKNSPCMVNGKCSKHFPKNFLQASTLDDDGYPKYKRSENGRTVTKSGTQLDNRYVVPHNRYLVLKYSAHINVEWCNQSRSIKYLFKYVNKGNDRVTAEFYKSTIDENCTEVIDEIGMYYDCRYVSACEATWRLFSNDVQFRTPSVERLSFHLPDCQSVVFDDDDTIESVLRRPTISQSMFPAWFEANKKYPDAQHLTYIDMPTKFVWKKTIREWQPRKRGFSIGRIFYVPPGSGEIYYLRCLLNIVRGPRSFEDIMTFNGVTHLTFKDACYARDIILSEESKKNYALIEIEKLLQVYNKSLTEFPPMPLPNLDDVSLLTNRLLLEEMSYDREFLAKESEKLSSQLTEEQRVAYDNIVEDVNKNKGGLFFVYGYGGTGKTFLWRALSAHLRSKGDIVINVASSGIASLLLPGGRTAHSRFSIPITVNEDSTCNIKHGSHLAELVVKAKLIIWDEAPMMNKHCFEALDRTMRDILKVNSHFGGKTVVLGGDFRQILPVIPKGTRQDIVAATINSSYLWSKCKVLRLTKNLRLSRAEQGLEQLEIEQFANWIASIGDGTLGGQNDGYANIDIPNNLLLQCNGDPIQIVVDEIFPNFRNGSCDATDMHNRAILAPTLDVVNSINEYMTDLHIAESKTYFSCDTVCRADSDSGILGDVHTPEFLNGLRASVTGESYIVACLPWSPKKKWTLTRRPAIVAGEEEVAATARLSCHGRQRRRSTACHGRWRKRYDANGGEWRQNGAVCVLGKGFDVLGSGKVVATSVAKISLKRRLIPAKALGRFARVCVEVDITKPLLAKFTVGGEVVPIEYEGIQMVCFSCGIYGHKQGQCRADEQKEGENVAGPNSDQGQNSKPPNMKEHPGTAPKSLPNIRTNQVYPPSMAQGPVPHQNPTRQPNMVSRGTFRGRGGKGEAPRRATAASQYTVVRGSNRGKQISTMVVHHSADAPVSSQSDEDDFELNEDPPDNARLFANTSEDLDDAMIDEAGHFDIDDSLMYDHQCF

>IbDExDH84

MEVKAEELSSTVAKRKSAEESSTGAEVPKEESASKRRNLTRTCVHEVAVPSGYSLCKDESIHGTLGNPVYNGEMAKNYPFKLDPFQEVSVACLERNESVLVSAHTSAGKTAVAEYAIAMAFRDKQRVIYTSPLKALSNQKYRELSQEFSDVGLMTGDVTISPNASCLVMTTEILRGMLYRGSEVLKEVAWVIFDEIHYMKDRERGVVWEESIVFLPPAIKMVFLSATMSNATEFAEWICNLHKQPCHVVYTDFRPTPLQHYVFPMGGSGLYLVVDETEKFREDNFVKVQDTFVKQHPANGSKGINAKSSGRIARGGNASGVSDIYKIVKMIMERKFQPVIIFSFSRRECEQHAMSMSKLDFNTEEEKDAVEQELVELLFQEGLVKALFATETFAMGLNMPAKTVVFTSVKKWDGDSHRYIGSGEYIQMSGRAGRRGKDERGICIIMIDEQMEMNTLKDMVLGKPAPLLSTFRLSYYTILNLMSRAEGQFTAEHVIKNSFHQFQYEKALPDIGKKVSKLEQEAAMLDASGEAEVAEYHKLKLEISQLEKKMMAEITRPERVLYFLLPGRLVKVREGGKDWGWGVVVNVVKKPPTASGSLPAALSALRGSTYIVDTLLHCSLGSSENESRPKPCLPIPGEKGEMHVVPVQLPLISALSKLRISVPPDLRPLEARQSILLAVQELEKRFPQGLPKLNPVKDMGIEDPEFVDMMSQIEELEKKLFAHPLHKSQDEHQLKSFQRKAEVNHEIQQLKSKMRDSQLQKFRDELKNRSRVLKKLGHIDADGVVQLKGRAACLIDTGDELLVTELMFNGTFNDLDNHQIAALASCFIPGDRSSEQIHLRAELAKPLQQLQESARRIAEIQHECKLEVNVEEYVEASVRPYLMDVIYCWSKGATFAEVIQMTDIFEGSIIRLARRLDEFLNQLKAAAHAVGEVGLENKFTAASESLRRGIMFANSLYL

>IbDExDH82

MGRKKQLRPHRSVGIRGKQAPGAELCDNNDARAEKDKLVEVDEPFFVEVDRSTWLSDEHMDISEIVLSDLSIKEEFYGYRLSDELYGDSRYLLRFRLDGVSQHLSRIKLGHWPVLSASSIVVELVAKHVKEGGEEHVVMVSGNLDGPDEGVSGLVHLASLKLLTLRPAMAVTFLEGLLSFRIRVEILRAAFEACETLLDNTRQLWKKSMISVMAWLRPEVTTSEARYGYNVIGDKDTGLGTDENSSASRKCAKLDVVSFYEAIKPSKEDPMLDDPLPNLVPELRPYQRRAVYWMVQREKGASEHSEPSKINPLISPLCMPLSLIDKSAAVYYNPFSGNVSLHPDSSFCYVSGGILADEMGLGKTVELLACVFAHQVTSSAIGSLSNSTQLEQDQRKNLKRLKRARVECICGSVSESIRYKGLWVQCDVCDAWQHADCVGYSPKKHKKMKASEEEMHRQDSSGKLKNRAKRKGIEEIVEMDGVYICRACSELIQATEAPVASGATLIVCPTPILPQWHAEIIRHTKPGSLRTCIYEGVRNSTLSETPLMDINELLSSDIVLTTYDVLKEDLSHDSDRHDGDRRFLRFEKRYPVIPTLLTRILWWRICLDEAQMVESNAAAATEMALRLHTVHRWCITGTPIQRKLDDLYGLLKFLKASPFDVFRWWTDVICDPYERGDEGAVAFTHKFFKPLMWRSSKGHVADELQLPPQEECVSWLSLSPIEKHFYQRQHETCVNDAHELIADLKDNIHKKKPQDSNDSLSGVVITNMDAAKLFNSLLKLRQACCHPQVGSSGLRSLQQSPMTMEEILSVLVGKTKVEGEEELRKVVVALNALAGIAIIEKNIPQAISLYKEALALTEEHSEDFRLDPLLNIHIHHNLAEILPQNSDGLETVQSAPGSSKESSFMTEGVDDDIQSSLKSETRSMEAANFTIKDLSNPVVDSATNYSGDGSSDVEPEKQRLRTSCEILKQRYLSVFNSKLYMAQQEFRKSFEQVCNAFTDRKNQHTAWWLESLHHIEQNKDLSSELIRKIGEAVSGTMNTNRASRIASCFRSITALKYFIQTGLDSLEGSRKTLLDRLLEIDQTMGNPRKEDIERVRYCPKCYANTEGPMCVHCELDDLFQAYEASLFRLNKGKYGEAITSAEEAVNLQKKMSALNRFYSTLSQSNKNPTSLTLENEDNGSKRDTGERVMVSKSPSDLEVVLGIIKSNSRGLLDREGMSAATKQLLLLEAMRKEYPQARYLSIAQAQVLRAYDEISMATSRLRLREDENDKSIDALDLGELDVASAEFSSEKFLALSSLSRVKGQLRYLKGLVQSKQKQQPESTDDTTSTQAMVTSRTSEENQNGSFTKAEEDACPICHEKLNSQKMVFQCGHVICCKCLIALTEQRSGHLGKPVTSWVMCPTCRQHTDYRNIAYAVDREHKSDKIPFDASENSEASITVQGSYSTKVEAVTRRILWINSKNSTAKVLVFSSWNDVLDVLAHAFAANDISYIRMKGGRKAHAAINHFRGQNSNSIGRGRSQDRQPDAKPVQVLLILIQHGANGLNLLEAEHVILVEPLLNPAAEAQAISRVHRIGQAKKTLVHRFIVRILSSPQYTYMRECKHNCEINPFVPLKQVKDTVEESIYKLNQSRTADSFVSGNRKNQDQPVLTLKDIESLFRVDQNPSGSLMHLPPSVAAALAAERRLAENTTSS

>IbDExDH90

MSMLYVLAKDISPQSYKKAIRLRLIRTYTVTEGSNESAIKSQECLFHDIEGTYVHASIHVDFIDQFSNLLKEGKVYPVKNFVVVSYYYQYKTTQHKYMMRFNQYTTIERHRRKGFPSLLFRIKPIENLLAGKVEEKQLIDVIGRVVEFYYPKDKVIAGFPTQLVDFLIEDSKGNRIKCTLWDEHVPAVMPFFNNHVDGHLIVLLQLCRAKIVDRQYEICMKGLRINLKNEHIRNPAKPISVLSVIHNEELEAQYCPSMVDDHDELSRMVEEDADDLESDESESGDEAISPSMQANSKLKDVANDQDEVDTMSIKRCLLDQFSSSQNLKKSRPLLIK

>IbDExDH85

MNKGARYGTPKYSGCCGHGKIVLPKMMRPPKRLFDLFFSHGEKQKEFLRYIRRYNNMFAFTSLGAKVDKSINIGNRPKFAQLYIHDTDNEVDNRISSFRTHLKIVKDIKHDLDDYNVLVKSFRCAKAVSRFLALIVGDIDPNMGSRDILVETNSGGLKRINKLNPAYLPLQYLILFPYGEDGYREDIQFHEVRMQKSGGRCRQFLVDAYTMVESSRSMYIRNNQKSLRCEAYKGLSDALTRGEVDTSKQVVYTIEFQERGLPHAHIIVFLERLSTPFTAESMDKFISTEIPDKEVDMEYYKAIEEFMIHGPCGLHRPKSLCMVNNKCSKHFPKKFVNVSSWDDDGYPIYKRSESSRIVEKNGVQLDSRYVVPPVERLSFHLPECQSVVFEDDDRIDNVLNRPTVNQSMFTAWFEANKKFDLAKQLPYIDMPTKFVWKKDIREWHPRQRGFSIGRIFYVPPGSGEIYYLRCLLNVVRGPTNWEDIKSYKGVIYPTFRDACYARGLLDDDKEYIDAIVKASQWSTAQSMRKLFVILLTSNLVNRPENVWDVVWQHLAEDVQFNKRKVLQQEDLCLSDDEKKNLALIEIERLLQSYNKSLKDYPHMPLPNYDDVVRCDNRLLWDELDYDRETLLRESQTMETLLTEEQNLCLAVICKNLVERDIVISVASDGIASFCFWRRRHLGFAIPIAVTEDSTCNITQGSNLAELIIQCKLIIWDEAPMMHKHCFEALDRTLRDLLRFTDSMLETGRDGTMGGPNDGYSSVLIPDEMLLPANGNDIATIVEEYISRTYLSCDTVCKSNAGNGILADMHTPEFLNGLKASGIPNHSLTLKVGSRLFWGIMRSNCLDTKDVNGPSDTRLPFKFQRRQFPLMLSYAMTINKSQGQTLTHIGLLLRKPVFVHGQLYVAASRISNPKGLRLLIANEASTPILNKLYVSVSDYINLPSNSKTGVLLGEVNKGSSPSIGGYEIVGLLGGGCYGYGYDPNECLRRKCGNNGPQISFPFRLKHIQPQECGYPGFDLLCTDKGETAMELPFPFLNATQPPFPIRGEAFFSNDLIIRGNTNSNSVLECAVIAGEVLGPFLVVVIVLVIYHIYSSNKMKKEDQVKIERFLEDYKALKPARYSYVDIKKITDRFSEKLGEGSYGTVYKGKLSDDVFVAVKVLNNSKGDGDGEEFVNEMSTIGSIHHINVVRLQIALGIAKGVEYLHQGCDQRILHFDIKPRNILLDQNFNPKISDFGQAKLCSKEKSIVSMTAARGTMGYIAPEVFSRNFGNVSYKSDVYSFGMLLLEMVGGRKKSDISESPETSQAQDYFPDWIYNILNRGEEVEIQIEKEEDSKIAKKLTIVGLWCIQWYPVDRPSIKVVIQMLEAEEPPSMPPNPFTSTDSSKPKANKPGKLFTSGLEIISELE

>IbDExDH105

MMLAEAAAVAAAVAPPPYVVNSFKFRRAMVLQPIPLAGSGNVVNLTSCRRKRREVQGSYTRRPMDTPGAYELIDNGTGEKVIVWGGVENDDDDSAIPSKELLSAWKSKSRTGGDVKAVLETVLCYVARLICTTEITARGCSRYKCSRREAELTSNKTLAGSFSRLKFHKMKALVKKSYTKEQEDDFRDHDEQNARVSATPEVDDFGEQERFDLLDDSSRSVQALDSEVLLQNSGEGWSRIPPIQLMKMMLGSFTFKIRSLMLNLSHKPFLLQKILLITWEDGVSDYLIESLRKQMFGFPSHIQAMAFERVIEGKSCIISDQSGSGKTLAYLLPLIQRLREEELQGLGKPLSQNPRVIVLTPTAELASQVLNVCRSFSKSGVPFRSVVATGGFRQRTQLENLRQELDVLIATPGRFMFLVKEGHLHLTNLNCAVLDEVDILFKDEDFETALQCLVTSSPVTAQFLFVTATLPVDIYNKLVENFPDCELITGPGMHRTSTGLEEVLVDCSGEEGSEKTPDTAFLNKKNALLQLVEESPVPKTIVFCNKVSFGIYSVSFLKGIYVRVLPFHAALDREMRLASMKEFHSPQPKNVSMFLVCTDRASRGIDFTGVDHVVLFDFPRDPSEYVPCWENSQRLEEKEKRSYLW

>IbDExDH99

MSAMEFKGEENHWNLGYEEQKKIKLPRRLSFGNDEHDSHLASGRTSCASATSFPLWAMSPGTPWARSPLHESPSPPLIYHCLASLHRREGNIFSIAVSQELIFTGSESSRIHAWKIPDCIEMGHIKAKAGGIHALLAYDKFLFTTHSDYKIRVWDVQVKENFQPKKITTLPRGRSFFLLPGRNIQQHKDYISCIAYNHYEKILYTGSWDKTVKVWKISKKHCVDSFVAHEGPVNAIVINQEDGCVFTCSSDGTTKIWRRVYGESSHILTMTLKFQPSPINALALYLSATDCFLYSGSSDGLINFWEKEKMSGRFNHGGFLQGHHFAVLCLASVGELILSGSEDATIRVWRRDGNSFHSCLAVIDGHHGPVRCLVATVETQDIMTGLLVYAASLDQTFKVWRVKVSPSEKVNSERSVVTQNSEIKECEMMSPVLSPSWVEKKVQGIHF

>IbDExDH102

MLLQLPRLTSSLCDPFDVDQAYLHRKTILQGLKPRSSASSSEESELARNIVYKWDEASPELRQAYKQFIGSVRELVGGEVVQDEFREVALSVYRHFSGPIPEGEEDRIISEKKLDLQKLVGYAIPNAVLHKAANLAQRLYELQENKHGTVPVLELEDGGGDNFEFGSDLVFQPPARFLVDASLEDLDFVVGHDKETSNYRNEYTHGYSFDGGHLPGAKFDLEWLRDACDRIVSESTSQLPRDELAMAICRVLDSEKPGDEIAGDLLDLVGDGAFETVQDLIMVRSIPAPSMLLSILSGVQHRKELVDAIHHGVSVLKADKMATSSQSASLVMALSSLLQASEKKNIFEDLIGRGEGPNSLATTALPQGTIRKHHKGYEEVIIPPTMTAPMKPGERLIEIKELDDLAQAAFQGYKSLNRIQSRIYPTTYNSNENILVCAPTGAGKTNIAMISILHENSLITLQQHLNITLEGYLHKNEFKIVYVAPMKALAAEVTMTFSKRLSPLNVTVRELTGDMQLSKNELEETQMIVTTPEKWDVITRKSSDMALSMLVKLLIIDEVHLLNDDRGPVESTQSMIRIVGLSATLPNYLEVAEFLRVNPETGLFFFDSSYRPVPLAQQYIDCGFLKARASGYGFCSFSQRHRKTADKLVEISQKNEDWELFRNEQHPQFELKKARNTNKEVVQLFDHGIGIHHAGPCLHCNLGLGVNLPAHTVVIKGTQIYDPKAGGWRDLGMLDVMQIFGRAGRPQFDKSGEGIIITTHDKRLVLGLGIHIFSSRMKMNPLAYGIGWEEVIADPSLSLKQRDLITDAARALDKAKMMRFDEKSGNFYCTELGRIASHFYIQYSSVETY

>IbDExDH107

MEGFWVSRYIIGCVERLNFHLEHQQNVVYGEDQTLDEILENQQLSEPIPRLAEELAYDKESLKIEHETLVTQLIDEQKNVYNSVMNDIDCNGGGLFFVYGYGGTGKTFVWRTLSSMIRSSGDIVLNVLRLTKNLRLRSLASEEDRHTVDWFSKWIANIGDKITGVVNNGLSEINIPPRFLLNCGHDPIATIVESTFPSARYGMIDELDLEGRAILSPTLDVFDQINQYMCNMNTAEGRTYLSCDSLCKAESDGENLSQVHTPEFLNSLRLFGLPNHSLILKVGAPVMLLRNIDHSLGLCNGTRLIVTRLTYHIVQAKIVNGTHQGTKVLIARMSLTPSDTRLPFKFERKQFPLMLAYAMTINKSQGQTLTHVRLLLKKSVFNHGQLYVAFSRVTHPDGLKVLALDEYGQTSFTTTNVVYKEVFNNV

>IbDExDH98

MCCFALENVTVLQELKVSVLKSPKSLADCLLNTGYLAFMATVRDSVKKVSGNHIEFRPIQSQAGGGLHSRGSSFNGCCSIQKGVNGSIPHNKKVKVVFVLGGPGSGKGTQCANIVEHFGYTHLSAGDLLRAEIKSGSQNGIMIQRMIKEGKIVPPGVTIALLQRAIQETENDKFLIDGFPRNEENREAFERITGIQPDFVLFFDCTEEEMARRLLNRNQGREDDNIVTIRKRFNVFKESTLPVIEYYKSKGKVQKIDAVKPPNEVFEAVKAVFTGGKVKQRCNGLDKVNIPCYAWRMMFRRSIVYKFKSCRCYTNMIPFSAMVSRTNASLPSILSFFLGSYDIANTSASLDRSAISLAITLAALLSMSDGSVVLRSLAFVVATDITLLVGRCTTLAWIKRFEKKTSSKNGLIYISVFCFHDFRLGLFAASLRDLLVEETEEEKRREDTCLGERFRRHGEKDVEIFSCGKDEDHERESGFSLFPLAQNSEEREKDREDGSRKVFYLENASEEENKWRGSKKSSHQNQQQAAAAPQQQQPSQPTKFGIQHFFERHTQNVLSQKSKARLDSRSSGSDTSAAALNDLRIANPSVQGHRNDSISIAENSVNAPDSMTNLRNVGLPNDASIMRSSKDAIESNKMHMNSAAPALHSSGSDPELRNTVVQIHSNSSQRLGKDLSSSSRIVEIENSKNTASLLGSESNNSSQNTPTHNALPVVVGDDANEAAEVTPEVCKSASVKRFKFSPGMLIKQSQDDGGDEITWRISPVNEATECSVKIREATGTGRLFTVKLVEFSAVFTEKGVSQVKTSPQPSGKVQKWLSSPPPKKAENSLVHPNKTTVTSVNVDQVMDIHAKNGKTNGEKNSAIANSQSPFKTPPSLTYCNDKSGKGSDANEVSDLLGSKHNKKALIELLDQVEDVISVESSVSEDNKISSGNDLHVRSDPVLPEARSNLRETSLLFLVLEVSEKHGHLESSGSKCSVKVLRLFNEQSGEERVVQLWDEWLYSVVAPGDTVHVIGEFDDKGTCDVNHEKNFLIVHPDILASGTRVAASFSCSRRAVLDERLKFNEYSAAALIGTLLHQIFQAGLIRESPTKEYLKEHAQVLLHKNLESLYACKVSENEFHKILIEAIPKILNWIHLFRDSEVLDIEEMAWAPKYGLKGMIDASVQAAMEHNAQVMLYTLLMSERYSTMIDYGLLYYLHTDQTQGITVRRSDLIGLLMRRNELANDLLRASTTQQLPPMIQSPNMCKGCRHLNVCSVYHKAYGGTTEGSGLGNVFDSLVNHLTTAHAVFLQKWDRLIDLEAKELEFVKKEIWSSHGSETEGSSSCLSPLVLGPSDKLAQKNLCTGNRFIYHFVHQSLPSVGIDQQNRHGSSITNNLECRLRNGDYVILSTEPGRLIIASGVITDTSNSHISVSFSKRLRLPGSRLRQDLHQQIWRIDKDEFTSSFAIMRFNLTQLFMQNEESIHLRKMIVDLEVPRFDSGCIFSQDPAISYIWSEKTLNGDQRRAILKILTAKDYALILGMPGTGKTSTMVHAVKALLMRGASILLTSYTNSAVDNLLLKLKAQGIDFIRIGRYEAVHEEVREHCLSTTNLCGIEDIKQRLEQIKVVGVTCLGITSPLLANKRFDVCIMDEAGQTTLPVSLGPLMFASKFVLVGDHYQLPPLVQSAEAKENGMAVSLFCRLSEAHPQAISALQSQYRMCKEIMELSNTLIYGNRLRCGSSEVENAKLTYSCSIAGPPWMKEVMNPNKPVIFINTDLLLAFETNDRKSLSNPIEAYIISEVSNKLVNTGISQEDIGIITPYNSQADLIRQAVSTSVEIHTIDKYQGRDKDCILVSFVRSNENPKSSVSTLLGDWHRINVALTRAKKKLIMVGSCATLSKVPLLKLLIETIEQQGGIFSVGKNDIRHCKVGIKRCSQTRSENGDL

>IbDExDH101

MYQVGMMMGIRYISESSRTCGEEWGALDSRYVVPHNRYLLQYRAHINVEWCNQSRAIKYPLKTPIKATIGSPQKFYKSTSNLCVFSPRCPSVPRLKDLASIYPECQSVVLRMTIIIDNVLNRPTVNQSMFTAWFEANKKFDSAKQLPYIDMPTKFVEEGYREPHPRQRVRGPTNWEDIKSYKGVIYPTFRDACYARGQKTCGMQCGNICQKMYNLTSAKCCNKKSYNKSLKDYPHMPIPNYDDVVRCQNRLLWDELDYDREELLKESQIMESKLTEEQKLVYETVVNDVDHQKGGLYFVYGYGGTGKTFVWRALSAKIRSRGDIVINVASSGIASLLLPGGRTAHSRFAIPIAVTEDSTCNITQDPIFGNGILADMHTLKFLNGLKAARIPNHSLTLKVGSPVMLLRNIDHSMGLCNGTRLIITRLADHVVEAKIVSGNHEGQIVLIPRMSMTPTDTRLPFKFQRRQFPLMLSYAMTINKSQGQTLTHVGLLLRKPVFVHGQLYAAASRISNPKAHAS

>IbDExDH109

MDGEARVSVNAADDLKPSYWIDACEDIPCDDFLHEYVNCDSHATATLASQTSNQEEPDPCFFGEIDHILENIKNGSSGDPPSTPCNVNGHENHASSPVTSETSCAFKDPKFNHRNGHLHMNNNVKPSESGFGRAWPECKGNGYSQLSDKGRYGKRARLSESMNERRNPDKPLGRKRLREFSDIDRRDRDQIKRREHQGFFKRERDWNEGRGYWERDKERNEMVFRTGLWEADRNREGKLPSGKSLEWNGGVEIKPEKPKEQPPEEQARQYQLDVLEQAKQKNTIAFLETGAGKTLIAILLIRSLYTDLQKQNKKILAVFLVPKVPLVYQQAEVIRERTGYQVGHYCGEMGQDFWDARRWQREFETKQVLVMTAQILLNILRHSIIKMESINLLILDECHHAVKKHPYSLVMSEFYHTAPKGKRPSVFGMTASPVNLKGCILFDFLFQVFLYSGILHGGVSSQVDCAIKIRNLESKLDSVVCTVKDRKELEKHVPMPSEIVVEYDKAATLWSLHEQIKRMEVEVELAAQSSSRRSKWQFMGARDAGVKEELRQVYGVSERTESDGAANLIQKLRAINYALGELGQWCAFKVAHSFLTALQSDERPNYQLDVKFQESYLSKVVSLLQCKLTEGAVAESNARVVDMDICKTLDSNMPDEIEEGELLDSYVVSGGEHVDVIIGAAVADGKVTPKVQSLIKVLLNYQHTEDFRAIIFVERVVAALVLPKVFAELPSLSFVKSASLIGHNNSQEMRTCQMQETIAKFRDGRVNLLVATSVAEEGLDIRQCNVVIRFDLAKTVLAYIQSRGRARKPGSDYILMVERGNLSHETFLRNARNSEETLRKEAIERTDISHLKGASNLVSTEALPGTVYQVESTGAVVSLNSAVGLIHFYCSQLPTDRYSILRPEFIMERAMRSQEVLLNIHAVCLDACKKLHEMGAFTDMLLPDKGSGTESEKVEENDEGDPLPGTARHREFYPEGVADILQVLAKIIRVGGFCRGKDDCDSSKFFHLYMYAVKCENVGSSKDPFLTQASEFAVLFGNELDAEVLSMSMDLFIARTVITKASLVFQGPVEITETQLASLKSFHVRLMSIVLDVDVEPSTTPWDTAKAYLFVPIAGGKSGDPVKGIDWDLVEKITRTDAWNNPLQRARPDVFLGTNERALGGDRREYGFGKLRHGLAFGLKSHPTYGIRGAVAQFDVVKASGLVPSRLTIKMCDTVKSSNSKLVMFDCCSRAEELIGRIVTAAHSGKRFYVDSVRYEMTAESSFPRKEGYLGPLEYSTYADYYKLKYGVDLLYRQQPLLRCRGVSYCKNLLSPRFEHSEGESEEIIDKTYYVFLPPELCFVHPLPGSLVRGAQRLPSIMRRVESMLLAVQLKDIISYPVPASKILEALTAASCQETFCYERAELLGDAYLKWVVSRFLFLKYPQKHEGQLTRMRQQMVSNMVLYQYALNKGLQSYIQADRFSPSRWAAPGVPPVFDEDTYEGESSLFDQEILNATRAETYHDVDGYGDEETEDGELENDSSSYRVLSSKTLADVVEALIGVYYVEGGKHSANHFMKWIGIQIDFNSNDINNKICPSNIPENILRSVDFEALECALNIKFNDKGLLVEAITHASRPSSGVPCYQRLEFVGDAVLDHLITRYLFFTYTDLPPGRLTDLRAAAVNNENFARVAVKHSLHVHLRHGSSALEKQIRDFVFEVQNELSKPEFNSFGLGDCKAPKVLGDIVESIAGAVFLDSGCDTAAVWRVFQPLLDPMVTPETLPMHPVRELQERCQQQAEGLEYKACRNGNIATVEVYVDGVQVGIAQNPQKKMAQKLAARNALVALKEKEAAEAANKGEDEEGKKKNCSQTFTRQTLNDICLRRNWPMPLYRCVHEGGPAHAKRFTFAVRVNTSDRGWIDECIGEAMPSVKKAKDSAAAVLLELITKWYSS

>IbDExDH104

MRFVIPKYYKVAQEFMMHGPCGSCRKNSLLMVNGRCSKFFLKKYVTTSVLDRDGYPIYLEATTAATINKNGINLDNRYVVPHNRYISPCEAAWRIFSFEIQFRNPSVERLSFHLPDEQSIIFMMVIMWIVSLIVRQLSQILTDTQKKNYALTEIEKLLTSCNKSLQDYPPMPIPDGPSNSLLVNPLIYEELSYDRQLLKEEHDLLVGNLTDEQFLIYNKVMYDVDTNKGGLFFVYGYGGTGKTYLWKTLSAALRQGRDCNQCQIQWYSFTIAPGGLTATQGDGKLGGNTEDDCNINIPSQFLLQKEILSNKLLTKHFHIHAKAQAQFRMHSHHETQNMVPSKHSRLPLLSNDAHIPSPFGQLAVEFTDSELRETAYEIVVGACRSSGSGRPLKFVSNSERSDSTSSSSSQSLQKSLTSSSASKVKKALGLKSKKKNRSDSAAADQAQNSASRKRASTVGELMRVQMKVSEQTDSRVRRGLLRVAAGQEVRFEHVYREQNKLADALAKKALCQPIHLYQLDEVPNDLWRLLQDDQIGASSHRRISTLGRRIESYILPLELLQQLKLSDFPSQQYYEAWQRRNLKVLEAGLLFHPSLPLHETDTQFQQLRGIIHGALVKPIDTGKYSDSMQALRNVASSLACRSFDGSVSQVCHWADGIPFNLRLYEILLKACFDVDDATSMIEEVDEVLEIIKKTWGVLGINQMFHNICFLWVLFHHYVFTGITGQDENDLLFAAENILLEVQKDANTTKDPTYSKILSSILGLILGWAEKRLLAYHNSYYRSNIDIMQSVLSIAISAAKILAEDMSHEYSNKRREVDVAYSRVDSYIRLNRFHLLNFILSNLPLRCWLSRGLLLFPVGKLILVENEKLISCRSSSKNQRNSLPILSILAQNISDLAFNEKEIYSPVLKRWHPLATGVAVATLHACYGDELKKFVSSINELTPDAVQVLIAADKLEKNLVKMAVADAVDSDDGGKALIQEMIPFEAEAVIANLVKSWIRTRVDRLKEWVERNLQQEIWNPRANKERVAPSGVEALRVIDETLEAFFLLPIPMHPALLPELLNGLDRCLQNYIFNIKSGCGSQSAFIPKIPSLTRCATGKKIGVFKKKERTNMVVRKNPHSGTLDSNDAFGLPQLCVRVNTLHHIRKQLEVLEKRSIAQLRDSGCVHNDNVSIGLGKSFELSASACIEGIKQLSETIAYKVVFHDLSHVFWDFLYVGNVSSSRTEPFLQELEKNLEIISSTVHDRVRTRVITDVMKASFEGLSMILLAGGPFRAFTIPDAAIIDEDFKFLMDLFWSDGDGLPSDLIDKYSVNLKGILQLLHTDTQSLITQFQCVIEDNYGASGKSMPLPPTSGRWSPSEPNTILPVLCYRNDKVATKFIKKHYNLPKKL

>IbDExDH100

MNYRFHNLLGAPYRGGNAVVANNSLLISPVGNRVSVTDLVKSETVTLPCQSSSNLRRIAASPDGVFLLAIDENNRCLFINLRRRTVLHRITFKHPVATAKFSPDGNFIAVAAGKLLQIWRSPGFRKEFFPFELVRTFADCNDRITSLDWSPDSDYILAGSKDLTVRLFCFSKWNKLNKPFLFLGHRDAIVGCFFGVDKKTNKVSRVYTVSRDGAIFSWGYSETDEKFDEMDCDVSEPESPGTPEQRQAQNEEVDYNSNAKKRKNFDGEDKSLDEEKGPWLHRLKWELLKKDYFMQTPAKLTACDYHRGLDMVVVGFSSGVFGLYQMPDFLGQLLVWEWKSESYILKQQGHYFDVWTVSSGFCFVTFSEHSNAVTALHFMPNNHCLLSASLDGTVRAWDLFRYRNFRTFTTPTSKQFVSLASDQSGEVICAGTLDSFEIYVWSMKTGRLLDVLSGHEGPVHGLVFSPTNAILASSSWDKSVRLWDVFDGKGAVETFPHTHDVLTVVYRPDGKQLASSTLDGQIHFWDPIEGLLMYTIEGRRDIAGGRLMTDRRSAANSTSGKCFTTLNYSADGSYILAGGNSKYICMYDIADQVLLRRFQITHNLSLDGVLDFLNSKNMTEAGPLDLIDDDNSDTEEGVDRQSRKKLAYDLPGSMPNHGRPIIRTKCLRIAPTGRSWAAATTEGVLIYSMDESFIFDPTDLDIDVTPDAVNLALSEGQTKRALILSLRLNEDSLIKKCIVAVSPADIPAAASSIPFKYLQRLVTAFADLLENCPHLEFILRWCQELCKAHGQSIQQNSRNLLPALKSLQKAITRLHQDLADTCSSNEYLLRYLCSASNNR

>IbDExDH106

MMLAEAAAVAAAVAPPPYAVNSFKFRRAMVLQPIPLAGSGNVVNLTSCRRKRREVQGSYTRRPMDTPGAYELIDNGTGEKVIVWGGVENDDDDSAIPSKELLSAWKSKSRTGGDVKAVLETVLCYVARLICTTEITARGCTSNKTLAGSFSRLKFHKMKALVKKSYTKEQEDDFRDHDEQNARVSATPEVDDFGEQERFDLLDDSSRSVQALDSEVLLQKLRRRMVKDSSDTANENDVRQLHIQNKESDAKSITQTLSASKDSVNHMGGWGKGNCRQWHLNVLLKERAALYLIKVDLERLWHIFFHLFNFREEELQGLGKPLSQNPRVIVLTPTAELASQVLNVCRSFSKSGVPFRSVVATGGFRQRTQLENLRQELDVLIATPGRFMFLVKEGHLHLTNLNCAVLDEVDILFKDEDFETALQCLVTSSPVTAQFLFVTATLPVDIYNKLVENFPDCELITGPGMHRTSTGLEEVLVDCSGEEGSEKTPDTAFLNKKNALCSWWRKAQSLRQLFSVTRKVENALKRFDRKGIYVRVLPFHAALDREMRLASMKEFHSPQSRTPLCSWFALTASRGIDFTGVDHVVLFDFPRDPSEYVRRVGRTARGAGGKGKAFIFVVGKQVSLAQRIIERNKKGHPLHDVPYT

>IbDExDH108

MALTFKIFFLIITLLHSPQPSSATTAPKIFKKLYAFGDSITDTGNTNSSTGPIFFTHVSNPPYGRTFFHRPTNRYSDGRLVVDFVAQALSLPLLPPYLDSKADRSFGINFAIAGCTAINYRFFERNNITLDITPKSLATQVRWFNSYLESVGCRDYKSTPKQCGEVFDDSLFWVGAIGINDYSYIFGSSVTTQTIQQLSINRTTGFLQDVSLLLVVVRTVLSVLSVQIHHQTLLKRGAKYVVVQGLHLAGCTTFNLYLSDEGDRDAMGCVATVNNQSRAHNAALQTRLAALRKQFPEATIVYADYWNAYKSILADHNIHGFTEPFKACCGSATEDLHFDIFTTCGSPGATSCDDPSKYINWDGGHLTEAMYKVVADKLLNGTFSHPPFSYLLRKKISSGH

>IbDExDH103

MLARTYCPFEIKGGPSSKHGKVSILIQLYISRGSIDTFSLVSDASYISASLARIMRALFEICLRRGWCEMTALILEFCKAVDRQVWPHQHALRQFDKDISADILRKLEERGADLDGLQEMEEKDIGALIKLCTRRKGVVKQHLGYFPSIQLSATVSPITRTVLKVDLILTPEFVWKDRFHGTAQRWWILVEDSSDHIYHSELFTLTKRMARGEPQKLSFTVPIFEPHPPQYYIRAVSDSWLQAEAFYTITFHNLALPEAHTTHTELLDLKPLPVTALGNGMYEALYKFSHFNPIQTQAFHVLYHTDKNVLLGAPTGSGKTISAELAMLHLFNTQPDMKVIYIAPLKAIVRERMNDWKKHLVSQLGKKMVGLMILDEIHLLGADRGPILEVIVSRMRYISSQTDRPIRFVGLSTALANAQDLADWLGVDENGLFNFKPSVRPVPLEVHIQGYPGKFYCPRMNSMNKPTYAAICAHSPTKPVLIFVSSRQTRSLHWTLFRKSTSTLDHRQNYASMFAASDEHPIQFLNMPEDALQMVISQVMDQNLKHTLQFGIGLHHAGLNDKDRSLVLVCTSTLAWGVNLPAHLVIIKGTEYYDAKAKRYVDFPITDILQMMGRAGRPQYDQHGKAVILVHEPKKSFYKKFLYEPFPVESSLKEQLHDHINAEIVSGTICHKEDAVHYLTWTYLFRRLIVNPAYYGLEDAEPGTISSYLSSLVQSTFEDLEDSGCIKISDDSVEPLMLGSIASQYYLKYTTVSMFGSNIGPDTSLEVFLHILSGASEYDELPVRHNEDKYNERLIDKVPYMVDNNRLDDPHVKANLLFQAHFSQSELPVVDYVTDMKSVLDQSIRIIQAMIDICANSGWLSSTLTCMHILQMVMQGLWFSRDSPLWMLPCMTNDLYNSLSKRGVINVQQLLDLSSANLQSIVGNSVASKLHQDLQHFPRVQVRLRIQRRDSGDNRSHTLNIRLENINTSRRTSKAFTPRYPKIKDEAWWLVLCNTTTSELYALKRVSFSDRLMTHMDLPSAATTFQGMKLLLVSDCYLGFEQEYPIKEYSNLQH

>IbDExDH113

MGGGSRSALGALVPSVKLEPLTAEESPPETVAQAQQMAIPAPEKEEADKDVLCPICMQVIKDAFLTPCGHSFCYMCIITHLKNKSDCPCCSHYLTAKQLYPNFVLNKLLTKTTARQIAKTALPVEQLRQAIEQGCDVSAKELESLLSLLSEKKKKMELEEAEANMQILFEFLQCLRKQKLDELHEKLVHIIDCPVHIPQTQKDLQYIKEDIRSVEKYRIELYRATGRCSSKMRILGDESSAKFPALLDRQSYGTTTNVPNAQGECIAVSNSVQTQITKAPFDSQLIQREIAQNGSDSQHAVARRRRVHAQFSDLQDCYLQKRRYWASKSQKQEERVSNGTKEGYNAGLADFQSVFSTFTRYSRLQVVAELRHADLFHSANIVSSIEFDRDDELFAIAGVSRRIKVFEFASVVNEPAADMQCPIAEMPTRSKLSCLSWNKYKKNYIASSDYEGIVTVWDVTTRQSVMEYEEHEKRAWSVDFSRTEPSMLVSGSDDCKVKVWCLRQEASALSIDMKANICSVKYNPGSSFHVAVGSADHHIYYYDLRNISQPLHIFSGHRKSVSYVKFLSNNELASASTDSSLRLWDVKDNIPLRTFRGHTNEKNFVGLTVNSEYIACGSETNEVFVYHKAISKPAAWHSFGSDTNEADAEIGSYFISAVCWKSDSLSMLSANSQGTIKVHVLAA

>IbDExDH120

MGMYCLANQLKAWNRRSAIKIRVMRTYCVMERRGSTQIKSREVIFHDEEGTVMHAHIPNDILPKFMNSFVEGSVYCVKNFFVVANWHTYKTSMHEYMLQFNGETIMKEYRSANFPRHMYRIRSFQSLRNIPSINDKELFDLIGRVVQIHAPQQKTINGNDARLIDFVIEDAQGNRLTCTFWDDHVAKIEPFYESPGNEPLYVLIQFCRLKFGVRDGDVKICSSYDVTQIHFNIDCPEMQQFKESMTELSQLTPMRSIASMSSMSFTNTHDDSSTQSLELTTINDLYDTEDFGDFWIAARGVNKCFKCNEIPDGSVRKYKLRLRVVDMKGTASFLIWDRECVDLLGIPAEDLYERNFNNLGHVKEITELKGRTMLLKISAKNEHYVRRNIPFPVVKIKTDQLLLQQLCPDLLALDENDFNSDGPSSEGDDKFMEGFESDEGESPVALLPPTSSTDCTTDGPIKRCLLDSLSSTKGGKKVKQSHVKL

>IbDExDH118

MTVVTKPTLCAHVTGMMQLSVMKVENVKQRCLPNALNYPMLEEYDFRNDTVNPDLDMELKPHAQPRPYQEKSLSKMFGNGRARSGIIVLPCGAGKSLVGVSAASRIKKSCLCLATNAVSVDQWAFQFKLWSTIREEQICRFTSDSKERFRGNVGVVVTTYNMVAFGGKRSEESEKIIEEIRNREWGLLLMDEVHVVPAHMFRKVISITKSHCKLGLTATLVREDERITDLNFLIGPKLYEANWLDLVKGGFIANVQCAEVWCPMTKEFFAEYLKKENSKKKQALYVMNPNKFRACEFLIRFHEQQRGDKIIVFADNLFALTEYAMKLRKPMIYGATSHVERTKILEAFKTSKDVNTIFLSKVGDNSIDIPEANVIIQISSHAGSRRQEAQRLGRILRAKGRLQDRMAGGKEEYNAFFYSLVSTDTQEMYYSTKRQQFLIDQGYSFKVITSLPPSDSGPELSYHRLDEQLSLLGKVLSAGDDAVGLEQLEEDTDDIALQKARRTMGSMSAMSGAHGMVYMEYHNGKKVHGHKSKPKDPAKRHHLFKKRFG

>IbDExDH116

MKEDVAEASKMLNKNWVLKRKRRKIPSGLDSSTVKEENSQPLESVLNNPSSKRGVKDEVASTRSSSKKKGNDGYYYECVVCDLGGNLLCCESCPRTYHIQCLEPPLKRIPTGKWECPSCNQKTDPLGPTSPLDSVSKRARTKVTTTGKSKNENKSSGSTKMSKNIGGSILDNRRLSSKEKSSLSQRVQKEKLDPSPNSVSSKTKLNHASGDGSAGCSSSYVSVEDESEEKKPISPGKGVTSLSNVIDSETNEEASVRRPRLSSTDKSPMDKCTTLMDSASRKDRKRKHDFFPVDSWKKRRTDKSNHTAKTKKGKSESNCAHPGANKSQRKLKSINHESSKILPEQDVGKDTVDEHLKEQVHVKKYIMTLLCQFSLLSALGMRHFQEKIVSEGASHPSNELGKVTVEPVTNEESGLDALQVDRVLGCRVRDADARNSCNTLVAPANDPQSEGSPVPKDENQLNEIASTDGSQGLADCPDEGRITENDIAKDKFQVYRRSMIKESKEGVNSVRTEDEVSGDYLANEEEFAKTSEKTSKETDSCVEIRDSNDNMECSQNFASNQLGNAKVVDTALETSSTSQKKHKGSTLAESSNSNGVTVSYEFLIKWVGKSNIHNTWLPESELKTLAKRKLDNYKAKYGTATINIFEEQWKIPQRVIAVRSASNGATEVFVKWTGLSYDECTWERIDEPVISKASHLIDLFHRFESQALDKDAIKDDTLKKRNEQQSDIASLTEQPKELQGVYFEFKARLPSLVLVPLSTLPNWMAEFALWAPELNVVDYHGSSKARTIIRQYEWHASNQNGSNKKTTSYKFNVLLTTYEMVLADLSNLRAIPWEVLVVDEGHRLKNSSSKLFGSLNTFSFQHRVLLTGTPLQNNIGEMYNLLNFLQPTSFPSLSSFEQKFNDLTTAEKVDELKKLVAPHMLRRLKKDAMQNIPPKTERMVPVELTSIQAEYYRAMLTKNYQVLRNIGKGVAQQSMLNIVMQLRKVCNHPYLIPGTEPETGSLEFLHEMRIKASAKLTLLHSMLKILHKEGHRVLIFSQMTKLLDILEDYLTIEFGPKTYERVDGSVSVADRQASIARFNQDKSRFVFLLSTRSCGLGINLATADTVVIYDSDFNPHADIQAMNRAHRIGQSNRLLVYRLVVRASVEERILQLAKKKLMLDQLFVNKSGSQKEVEDILRWGTEELFSDSSNTTGNGLGENLNNKIEAVMEIEQKHRRKTGSLGDVYKDKCAEISPKIVWDENAIFKLLDRSNLQSDSPDSNEAELESDMLGSVKASEWNEEATEEQAGEMMLANDDTSTQNLEKKEDNLIGSSTEENEWDRLLRVRWEKYQSEEEAVLGRGKRQRKAVSYREAYAAHPNETLSESGAEEDQEQKPEPDLEPEREYTPAGRALKAKYTRLRARQKERLAKRNAKETSAPVEGSTGTQYFAQLLQSQAQDGNQSALFIQPSEENSSALFLDGSSSGQTSEGRKNRTDSTVRLVKLPKHLGQPNLGEGPSYMNSEAENPLVIGLCAPNANPMESSQRKFSRSYNRKNRLGLGSDLPPSIAPCPTTSDEMGTKAHETISGRFKLPDPPLDVSQSRPKISLPDLYHPFNPHPLIFQQGKGSTVNLENSARSRVADVNEPHHDHPTMPLFPNLKFPMPDAPRCNQPEPEVPPTLGLGQMPSKLSPFPENHRKVLENIMLRTGSGSGSGNPFKKKSKMDIWSEEELDNLWIGVRRHGRGNWDAMLRDPRLKFSKFKTVEDLSIRWEEEQTKILDVPAFPGKGLKPPKSGKSSLFPSVSDGMMTRALHGSKFSGPPLKFQNQLTDLKLGFGDLPPSLSRLEHADHLSRISPLPPWNPDKYPLNVPGDLNAGPSDRLGAPSKFLMESQLLLSSLGTSSLGSLGVNCPPSFSLPQKEAEESASRFGKLSSILDRSLNFLPDSNNNVGNGESSGFALPSDVSKGKNIPQSKGKEVAECSSPKNKLPHWLQEAVNVPPKSAAPSLPPTLSAISHSLRLLYGEEGPTIPPFTVPAPLPSQPKDPRLSLKKKKKKKQKLHMIPEFLQTLKGSGDTLQSHLPGEKTGSSVVPQDQSSLMLLKSLASTSESPSVEPDLSLTTDINENTAPPPLSADEGTSRDTHEKANDTSAPGKSGSSEEDKDQSESRDSSQTQSDPARGRQHAVEEISSEGTVSDHLAIEP

>IbDExDH115

MDHSTINTQHNLAARDLLMDFDGVDNTIGTNDKTLLEPVQYADYGDAQYLCDSCGAIFWYDERMNKTVVRGKPKFSICCNQGKIKVPNKSTPPAAIGNLFFNGDDKSKHFLKHIRSYNNMFCFTSMGGRVDRSINRGGGPPTFRLNGQNYHLIGSLLPDDGNRPNFAQLYIYDTQNEISNRIESIRPNREDTTMHTEIVANIQDELDKHNVLVKSFRMARDFIDQNQPTNVKMKLIGKRHKDARAYNLPSVSEVAALIVGDIDPSMGPRDILVETKGGGLKRINELNPVYLPLQYPLLFPYGEDGYREDIPFNVVSGVGNNCRKELFQQFLVDAYTMVEAGRLLYVRTHQKRLRSESYSCLTDALTRGEVNPSAQGRRVILPSSFTGGARYMIQNYQDAMAICRWVGYPDLFITFTSNPRWPEVDRYLSSKGLRPEDRPDIMSRDRCQK

>IbDExDH121

MNKVQIKDSLGTNRVTAEFYKSTNDEQQNDVVDEIAMYYDCIYVSAYLCLSDDEKKNLALIEIELLLQSYNKTLKDYPHHMPIPNYDHAMHCDNRLLWDELDYDRDALLKDCQTMESQLTDKQKVVGDIVINVASSGIASLLLHGGRTAHSIFAIPIAVTEDSTCNITQGSNLTELIIQCKLIIWDEAPMMHKHCFEALDRTLRDLLRFTDQNAASRTFGGKTVVLGGDFRQILLVIPKGSRQDIVSASINFSYLWDSCQVLRLTKNLRLSNKESTVDLSIVQQFASWLAAIGDGTMGGPNDGYSNVHIPDEMLLPSNGNDIATIVDSIFPLFKAGGCQQQYISNRAILAPTLDVVNAINEYMTDLHVAENKTYLSCDTVCKSDSNNGILADMHTPEFLNGLKASGIPNHALTLKVGSPVMLLRNIDHSMGLCNGTRLIITRLSDHVVEAKIVTGNNAGQIVLIPRMSMTPTDTRLPFKFQRRQFPLMLSYDTCRLTIKETGLCSWFPCWPDDIALVPGPPAIDTDLQDDELQLDWNCEKGREIRLIPILFVAESCVFSLLWKRRKDNLMRRAEKPKPETLILDVVAPGPALIQHLVRSKALRLLEGWLLTQPGVSNDDKKKVMVGKVNSCVFDELLNMGKSLLERKRKGAEGKESYAEVALGLIAMMNLCKLIAFHRPDTVVFTFMLLGSYST

>IbDExDH117

MESISAPGHPPTSTEEEHGVEAQGTERNPQQPMPSNSVVSTKKRKVVVESRSKLDLDCYTVWTLNQAKFVNCKFVISLLQARLHVSSLCIATAHCLATHCVASPPRRHLPPPPPPPVRLCLGPRQSLRALDSRHQPQPTAHQVWSSTLLRFFPYYLLIKLAIIEGTNNIVALLASLLLFPMVMMDVDSTDLRPERSGEMREQDDSPGRERWGEDGLDALEENDLEKSRDSSKHQSKTSGRKEEKEHRSKNRERLREGEKEDKGLQKDRMSTRDRRKEDRDDTEKDKTRDRVREKDTDRDKYRDKERDRGREKDRRYHEKEKERDKDDLERDGGRGRDKERAKDKSRDKDKDKERTKDREREKEKDREKQREREREYDRDGSYRKLQDEGHNRSMHVGNDDMSRFNEEDGIDGDLSEQEMGLADAEDIRISKGESIGSQPAVSELEERILKMKEERLKKKPEGASEVLSWVNKSRQIEEKRKAEKEKALQLSKIFEEQDRIDQEESDDEEATRRTTEELSGAKILHGLDKVLEGGSVVLTLKDQSILSGDDVNQEVDVLENVEIGEQKRRDDAYKAAKKKKGIYDQSDELGSEKKMLPQYDDPDGRGEKKLEELRRRIQGVATKNHTEDLNSSSKILTDYYTQEEMLQFKKPKKKKSLRKKEKLDIDALEAEAISTGLGAGDLGTRNDKTRQALKEEKERAEAEMRSKAYQTAYTKAQEASKALRPEQAITTKAEEDDAVFDDDDEELRKSLERARKLALKKQEAGKSVPEAIALLATSNANNSTVDNNSNPAIGDSQENKVVFTEMEEFVWGLQLDEGRCDTNSPEAVITEDGGWTEVKETENNEPALEEDEELTPKEAFRLLSHKFHGKGPGKMKQEKRMRQYQEELKVKQMKNSDTPSQSVERMREAQEKLKTPYLILSGHVKPGQTSDPRSGFATVEKDLPGGLTPMLGDKKSGFTTSVISEVEATGASITFLLKIINLVPQVQRL

>IbDExDH124

MHRVASVGNTSNSSRPRKEKRLTYVLNDADDTKHCAGVNCLAVSKSSVPDGCDYLFTGSRDGTLKRWALAEDGATCSATFESHVDWVNDAVLTGGNTLVSCSSDTTVKVWNSLSDGICARTLRQHSDYGTCLAAAEANNNIFASAGLGGEVFVWDLEAALAPVSKSSDGTEDECSNGSQVYAPTAAKGHKESVYALGMNESGTLLVSGGTEKVIRVWDPRTGSKTMKLRGHTDNIRALLLDSTGRFCLSGSSDSMIRLWDLGQQRCVHSYAVHTDSVWALASTPSFSHVYSGGRDLSLYLTDLATRESVLLCTKEHPIVQLALHDDGIWVATTDSSVHRWPAEVQNPLKVFQRGGSFLAGNLSFSRARISLEGSTPVPVYREPSFSIPGTPGIVKHEILNNRRHVLTKDTAGAVKLWEITRGAVIENYGEVSFEKKKEELFEMVSIPAWFTVDTRLGNLSVHLDTPQCFSAEMYSVDLNIAGKPEDDKINLARETLKGLLAHWLTKRRQRFGSQVSVNGEVPPGKDISSRNLAVSRVEGSQGGPWRKKITDLDGTEDEKDFPDFFDFHVARTLESSTVQILTQGKLSAPRILRIHKVINYVIEKMVLDKPPLDSLNSDGTFVPGGPASGVGEFRSGLKPWQKLKPSIEILCNNQASLCNL

>IbDExDH112

MGHRTIHQQSQCPIPNISDDLGRNLQAEADTNLSIRRSQRLTFQKGKAVVNSQQLIIDNIQNDKVVSMPALTVAYHDIGDPSNICQNCNAIFWFEERMNKAARCGTAKYSGCCGHGKIVLPKMMRPPKRLFDLFFSQGEKQKEFLRYIRRYNNMFAFTSLGAKVDKSINIGNAPPIFRIHGQNFHLIGGLIPQEGNRPKFAQLYIHDTDNEVDNRISAFSMGEQNAKLHVEIVKDIKHELDEHNVLVKSFRCAKGNIDSNPGVDFKMRLIGKRNSDARTYNLPTVSEVAALIVGDIDPNMGSRDIMVETKAGGLKRINELNPAYLPLQYPILFPYGEDGYREDINFNEVIQQRSGGRCRVSQREFFAYRIHERFNEMSTILYARRLFQQFLVDAYTMDAMAICRHKGYPNLFITFTCNPKWPEIQRYMKKCNLNAEDRPDIVCRGFKMKLDCLVKEIRSGTLFGTVSAVVYTIEFQKRGLPHAHIIVFLERQSEPFTAASMDKFISAEIPDKEVDMEYYKAIEEFMIHGPCGLHRPKSPCMVNNKCSKHFPKKFVNVSSWDDDGYPIYKRSESSRTVEKNGVQLDSRYVVPHNRYLLLKYRAHINVEWCNQSRAIKYLFKYVNKGNDRVTAEFYKSTSDEQQNDTIDEIAMYYDACSACRQLASFSIDVQFRFPPVERLSFHLPECQSVVFEDDDRIDNVLNRPTVNQSMFTAWFEANKKFDSAKQLPYIDMPTKFVWKKDIREWHPRQRGFSIGRIFYVPPGSGEIYYLRCLLNVVRGPTNWEDIKSYKGVIYPTFRDACYARGLLDDDKEYIDAIVEASYWSTAFSMRKLFVILLTSNLVNRPENGWDAGWQHLAEDVQFNKRKVLQQEDLCLSDDENKNLALIEIERLLQSYNKSLKDYPHMPIPNYDDVVRCQNRLLWDELDYDREELLKESQIMESKLTEEQKLVALSAKIRSRGDIVINVASSGIASLLLPGGRTAHSRFAIPIAVTEDSTCNITQGSNLAELIIQCKLIIWDEAPMMHKHCFEAVDRTMRDIMRFTDSDAGNRTFGGKTVVLGGDFRQILPVIPKGTRQDIVSASINSSYLWESCQVLRLTKNLRLSNKESTADLKTIQEFASWLAAIGDGTMGGPNDGYSNVLIPDEMLLPANGNDIATIVESTFPLFKGGGCQLQYISNRAILAPTLDVVNEINEYMTNLHVAESKTYLSCDTVCRSDAGNGILADMHTPEFLNGLKASGIPNHSLTLKVGSPVMLLRNIDHSMGLCNGTRLIITRLADHVVEAKIVSGNHEGQIVLIPRMSMTPTDTRLPFKFQRRQFPLMLSYAMTINKSQGQTLTHVGLLLRKPVFVHGQLYVAASRISNPKGLRFLIANEDTDSSNYTTNVVYHEVFNNL

>IbDExDH122

MRLTKNLRLNSMQPGLDQQRMEEFANWLASIGDGTIGEDNDGYAEVDIPSQMLLKCNGDPIATIVNRTFPQFNGGISDGSCFHSGYFSTYITSVNEVNEYMSDLTIGEGKTYYSSDTACKADGSSTLFADDHTLEFLNTIRASGPPNHALTLKVGSPVMLTRNIDHRTMQCTHAEAFRMADQFDLASQLNPDFTTKAIPLHLVRTYAVPESNKTQQCTPKKPEGVKSIKCLFHDQELGKEEVDEKDSEKTLKQWPHCEEVSYGAIHKG

>IbDExDH110

MVMDSAIEKWWNSDESPRTRSSFASPSASYPKRRRRNQVDSVCCSVEHEHNKWKLSSKVVDRCDPFALCNLEEGLDYEKFGSVAKEIEDLIARRGQVLNKLYAHDISSPCGTLDVGRHCDEDSNVSISHVIDLEDEHQEHNLPPAMPIPTMDIPPSIPLVILDSDNEDCGSEMSSCPYQGNISKNTGDGIHKNDTMECDNAGHQTLLGGATLCSGNGKKKDGGVYVGVEDEENEQPDADGVDDIWKEMSFVLEYSKDTKVDLLSEDRAIKDGEEDCDHFFIMKDDIGTVCRICGYIEKSIETIIDLQFGKATRSTRTYRYEERKTVGPRSTEIQPDGTKSSKEDFMEEICAHPRHKKQMKAHQLEGFNFLLSNLVTDSPSGCILAHAPGSGKTFMIISFLQSFMARYPHARPLVVLPKGILATWKKEFRRWQVEDLALYDFYSAKAENRSQQLELLRHWSAERSILFLGYKQFSSIVCDNDTTNTAAECRMILLTCPSILILDEGHTPLNEETNTLTALEKVQTPRKVVLSGTLYQNNVREVFNILNLVKPKFLKLDTSKAVKNRILSRASISGRHNLVKDVSDREFYELVEHTLFEDKNLSRKVTIIKDLREMTSKVLHYYKGDFLEELPGLVDFTVLLKLHQRQKSEVAKLKKLKQKFKVSSEGSAIYVHPKLKCLCGSRERFDEKKIDMILEDLEEQEGVKTKFYLNLLQLCESRGEKLLVFSQYLLPMKFLERLTVKLKGYSMGKEIFMITGDADSGMRESSMEQFNTSADARVFFGSIRACGEGISLVGASRVLVLDVHPNPSVTRQAIGRAFSPGQQKKVYTYRLVASGSPEEEDHSTCFRKESIAKLWFEWNEYDGHCDFQMEEVDVKTCGDMFLETPCLSEDVISLFKR

>IbDExDH123

MIESGVSGSVEDLVAWDKCNPKLAGSTSFGSFDLNKVLEFGALLASGGQEYDLPADNSKNPKERLARQKQNLRRRLGYDGLDELKKGGLDVCEQFMDVNEMIRDEDLVMQRVNSPVTSVSSHYYSPRPQGNIRHFVANMVPSAKSRRPSARELNLLKRKAKVSSKDQTKGWSKDGEPDAPQPQDMTSPRGIQSDASGSNKMHLDTVSDEDGLESDADGCWPFQSFVEQLMVDMFDPLWEIRHGSVMALREILTHQGANAGAIMPDRSRDVGLGSILKDRVVEKQESVIDLNIQVPLDESEPAFKKPKIEDASPLVMDTIPSASMVGNIDNIQVKVEDVGLNLPVRQENGEVNFGSVKMEIQSNSNSESSFNNYMVEAKSSCEDNISLEKMDLPRNLPENCELMNLVKLARHSWLKNSEFLQDCAIRFLCILSLDRFGDYVSDQVVAPVRETCAQALGAVLKYMHPTLVYETLSILLQMQHRPEWEIRHGSLLGIKYLVAVRKLVLVIGEMLHDLLCYVLPACKSGLEDADDDVRAVSADALIPTAADIVSLKSEILHSIVMLLWDILLDLDDLSPSTSSVMNLLAEIYSQERMIPNGYGNLPSLEKQDLNLNEVVYQNDIGEGLSSLENPHMLSTLAPRLWPFMRHSITSVRYAAIRTLERLLEAGRKRSLCEASGSFWPSFILGDTLRIVFQNLLLESNEEILQCSGRVWRLLLQCPVNDLTDAAKAYFSFWIELSTTPYGSPLDTTKMFWPVALPLKSHFKAAAKMRAVKPEGDPYKNICFGSAEGTSLQEKNGDTSTHIGKIIVGGDVDISVTQTRVVTATALGVLASKLDDSSLQYVVDPLWNALASFSGVQRQVSSMLLVSWFKELKFKDISKSEGVITCISSNFGERTYEKMRNEARQLYHGTEASGMFKDILSSSKLDLESLSADDAVNFASKLSFLNNITTGEESAGRNIFDELESLKQRVLTTAGYLKCVQNNLHVTVSALLAAAVVWMSNLPAKLNPIILPLMASIKREQSKVFCPSEAVITGRNQKFICFPPGDDRSKVEGFISRRGAELALKCLCEKFGGSLFDKLPKLWDCLVEVLKPGNVEGLNPEDEKLISEAIDSVTDPQILINNIQVVRSIAPLLDETLRSKLLTLLPCIFRCVCHPHVAVRLAASRCITTLAKSMAVNVMSAVVENVVPMLGDMTSVHSRQGAGMLVNLLVQGLGVELVPYAPLLVVPLLRCMSDSDHSVRQSVTHSFATLVPLLPLARGVSPPAGLSDRLSRNQEDVKFLEQLVDNSHIDDYKLSTELKVTLRRYQQEGINWLAFLKRFNLHGILCDDMGLGKTLQSSAIVASDIAEHVAANTAENLPPSLIICPSTLVGHWEYEIEKFIDASLVTTLQYVGSAQERTSLRSQFNRYSVIVTSYDVVRKDVDSLRQVFWNYCILDEGHIIKNAKSKVTLAVKQLKAQHRLILSGTPIQNNVLDLWSLFDFLMPGFLGTERQFHASYGKPLIAARDPKCSAKDAEAGALAMEALHKQVMPFLLRRTKEEVLSDLPEKIIQDRYCDLSPVQLKLYEQFSGSDVKQEISSMVKVNESDTGQGSEAPKASTHVFQALQYLLKLCSHPLLVLGEKISESVSSVLSAFLPNGSNIVSELHKPHHSPKLVALQEILEECGIGIDASGSDSPVNVGQHRVLIFAQHKAFLDIIEKDLFHSQMKNVTYLRLDGSVEPDKRFEIVKAFNSDPTIDVLLLTTHVGGLGLNLTSADTLVFMEHDWNPMRDHQAMDRAHRLGQKKVVHVHRLIMRGTLEEKVMSLQRFKVSVANAVINSENASLKTMNTDQLLDLFTPADGKKAPNVSQSSDDKFEGDPKLTRGGKGLKAILGGLEELWDQSQYSEEYNLSHFLAKLNAGKWGKKGNGYDKCEPAECGCNGEEGVEHDGDGLGSSYVIIGAVLWRLALWGGKKLVYHAKLKRWRLRKL

>IbDExDH114

MMMANGAAEIRYPEEPSAAGGEDCENPEHLSIDLEALWGMLGDKNSDPSQSMLGDSPDEKPQGNSSSTLPFSATSFKPEALDCRIGVSNGSPSDLTGRPVIIDAPKFDSSFQVGSAVHVSDGSLSDWKLPGNGKCCSDIDNASQSASSNIQAMLSSIITESSGVELDMPSYDEPLSASNPAETFDYLGGNWGQYPDILSADIPSSSSSLLQQDFQYKSDFKLLPCNGDKMLNSKEEEQEPPTRYTSTDSMIGNEDLTGSTDEVWDGPTIANSDIKDLNFNSCGVDNSKFLTVMKASDQQHGQSSRFCTKSAHIKDRKDELINRKPFGAAETVIAKELSNPHFPRHLPFHSSKMDILYGKHQNKDMFPEFVNPGYHSNVFIDGTSEMPILINHDHGPDLLVGEKHLSLPTSSVKNQLGCARTENTHISGKTFDSHMATFTPQVFQISSYAEKPYADTDDDDDDDDVCILEDLSAPARPNPCVSKGKSLVTFQHATFSETFNHAGVGQMRLKLNNEQSVFQAALQDLSQPRSEDTPPDDALAVPLLRHQRIALSWMVKKETMNPHCSGGILADDQGLGKTISTISLILKERPSSVRVSTAISKQTEAETLNLDEDTAVDTQLPSKSDSCQANGSPITGAKKGRAAAGTLVVCPTSVLRQWSDELHNKVTSKANLSVLVYHGSNRTKDPLELAKYDVVVTTYAIVSMEVPKQRLVEDEDDETGKRTEGSMELSYSKKRKNPPSSGKKSSKLKKGMDSELLDTYTRPLARVAWFRVVLDEAQYIKNHKTQVARACWGLRAKRRWCLSGTPIQNAIDDLYSYFRFLRFDPYSSHKVFCASLKVPIQKNSSSGYKKLQAVLKTVMLRRTKGTHIDGEPIIALPPKRIELKKVEFTDEERNFYCKLEADSRAQFAKYAAAGTVKQNYVNILLMLLRLRQACDHPLLVGGSNSSSVWRSSTDMAKKLPQEKQIELLNCLEASLAICGVCNSLIQHSALMRTAIGCVLEDPPEDAVVSICGHVFCNQCICEQLTGDDTHCPAQNCKMRLSVSSVFSKSILVGSLSDQPNVVTDLDCPGSRPCKTSDFSSMGCSSYDSSKIKAALEVLLSLSKPQESTPRIVSRCDQGQMSCSGDSQVKMNLDTNGYSKCSTKIAGEKALVFSQWTGMLDLLEACLKTSSIQYRRLDGTMSIAARDKAVKDFNTIPEVTVMIMSLKAASLGLNLVSACHVLLLDLWWNPTTEDQAIDRAHRIGQTRPVTVLRLTVKDTVEDRILALQQKKREMVASAFGEDENGARQTRLTTEDLEYLFRI

>IbDExDH111

MHRILTDDCSSDGGELLVDGSGSVVGDGVRLHRRYGYTKLLILHCFIVPIAPLYILGCDLALMNTRNAIIQVPERKGGETIECKDYFMIKRYVQHKSRDNVKNNNFVVMGRKRKPPSTSNLNKWYKGLLCVDEHSLEATRVAYMRGIALPVIITAEQLRMHNKKFKGRMDCDVEVGELQTLNARNLILAFDEVDATSSSEKNMPDVMYADFGDPEYRCEHCHSFFGLKKGSINRRNQDSKNNIHVKALVADIKEVLDENNALVKSFLMAKSRLNQTPVEVKMRLIGKRNTDARRYNLPTVSEVAALIVGDLDPMLGHRDILVEGKSGQLKRINELNPSYLPLQYPILFPYGEDAEIPDKDNDIKYYNAVEEFMIHGPCGAANKNSPCMVNGKCSKHFPKNFLQASTLDDDGYPKEWQPRKRGFSIGRIFYVPPGSGEIYYLRCLLNIVRGPRSFEDIMTFNGVTHLTFKDACYARGLLDDDKEYIDAINEASQWSSGHSMRKLFVILLTSNSIIRPENVWNATWNHLSEDAEYYHRRVPQNTDIILSEESKKNYALIEIEKLLQVYNKSLTEFPPMPLPNLDDVSLLTNRLLLEEMSYDREFLAKESEKLSSQLTEEQRVAYDNIVEDHTWPKGDIVINVASSGIASLLLPGGRTAHSRFSIPITVNEDSTCNIKHGSHLAELVVKAKLIIWDEASMMNKHCFEALDRTMRDILKVNSHFGGKTVVLGGDFRQILPVIPKGTRQDIVAATINSSDLE

>IbDExDH119

MASDQVIAELVGMGFEFSDITDAVKAVGPSVSNAIEYILDGSHRNKGTSTSSTCVDPNTNSRQKRASNALHAFDRMRQSNIKESLKSACKPIKRKYNDEPVSLCESEALQRTLESPTELLPVMDSDMCNAHETSVPSNCKDEEVFGQDWEKKVKTLLVKHFGFSSLKSFQKQSLEAWLNHRDCLVLAATGSGKSLCFQIPALLTGEVVVVISPLISLMHDQCLKLAKHGISACFLGSGQIDRSVEKKAMNGLYSIIYVCPETILRLIKPLQNLAESRGIALFAIDEVHCVSKWGHDFRPDYRQLSILRENFRVDTLKSLKFDVPLMALTATATNCVREDILKSLKMSNETQIVCTSFFRPNLRFSVKHSRTSSLASYQKDFQEIISTYSTMKKFRKNNLISPDLDDVTAISSSTSDCSIPELEALLENDVDNINDDAFSENDDEIGLPMRHSLPASKERELSVEFLEDESDLFQDVDDLDVSCGEFHGQPIKDFCASVPLRTVDLPSKPEERLKLQHEPLEYGPTIIYVPTRKETLSIAKFLSKFGVKAAAYNAKLPKSHLRQVHKEFHEDYLQVVVATIAFGMGIDKSNVRRIIHYGWPQSLEAYYQEAGRAGRDGKLADCVLYANLSRIPTLLPSKRSEEQTRQAYKMLSDCFRYGMQNSQCRAKMLVEYFGEKFGLQKCLLCDVCINGPPELQNLKAEAAILMQIVYAHYGQSSCADISYDGDIIRHNGKRSKLYEKPNIREIVSRIREQHQEFTTSEVLWWRGLSRILEAKGFIRESDGKNHVQIRCPELTELGRRFLRCDSGEEAFNVYPEADMLLSTATAKAQGKPYSSFAEWGKGWADPEIRRQRLQRKRSWKSPKKRKSHKPLPSTNTVRGRLTAKLSAKK

>IbDExDH126

MKKLTGSTILRSGKKQKTTDKVVDSPAFTPVNPIVNVVDSERLYGGFMMCSPRKPINMSTNVNVHPTSFEDNYVATRGTPLSATTNVTSNPNSYALNVEIPPVTPLSVITDVNMRNIPSPFTQCSFNISNMSTSTSSSSQPLADVSNGGRVDRSINTGNAPPVFRISGQNYHCIGSLVPGEGSTPKFAQLYIYDTDNEINNRINSVRQDNNSGDIREDIVEKLKNMLDQNNVLVKCFRMARIEIQSNPVVEVKMNLIGRRSKDGRTYNLPTANEVAALIVGDLDPSMGDLDILIQSRTGQLKRINQLNPAYLPLQYPLLFPYGEDGYREDISFSDAWHQRHHGGRKRISPKEYFSFYIHERVNENHTLLYSRRLFQQYLVDAYTMIESARLIYIRTHQKALRCEAYQGLSDALTRGELDPTGRGKRIILPSSFTGGARYMIQNYQDAMAICRQIGYPHLFITFTCNPKWPEIERYVAHRGLKPEDRPDIICRVFKMKLDAMIEDIKHKSVWRHMWRETIAISHAEFMSSSTNGHNDEVVDEINMYYDCRYISACEATWRLFGYAIHYRTPPVERLNFHLEHQQNVVYGEDQTLDEIVENQTVKQSQFTAWFEANKKSEDAFSHLCEFPSCGELYYLRCLLNIIRGPSSHDDIRSVAGVIHKSYRDACYEYGLLDDDKEYIDGITDSRYWASASALRRLFATLLSSSTISRPEIVWDAVWEFLAEDAQFHHRRRMNNPDSDKKQFALVELEKLLSLWGKSLRDFPQMPLPDETNIGFMENMLIAEELAYDKESLKIEHETLITQLTEEQKNVYDSVMNDIDCNGGGLFFVYGYGGTGKTFVWRTLSSMIRSRGEIVLNVASSGIASLLLPGGRTAHSRFAIPLSLNEDSIATYRKVLRLTKNLRLRTLASEEDKRTVDWFSKWIANIGDGITGVVNNGLSEIDIPPRFLLNCSNDPIATIVESTFPSARYGMIDELDLEGRAILSPTLDVVDQINQYMCNMNLRRSNLFKF

>IbDExDH127

MARSEIQNNPRTEVKMRLLGKRTKDARTHNLATVSEVAALIVGDLDPKMGFRDILVESKSGKLKRISELNPAYLPMQYPILFPYRDDGYRDDIQFAAIRSQLDGARNNQKALRCEVYKGLSDALFKGNIDPSTQGKRIILPSSFTGGARYMIQNYQDAMAICRWIGYPNLFITFTYNPKWPEIQRYIGVRDYYKAVEEFMLHGPYGVARSNSPYMVNGRCTKYFPKRYVVPHNRYLLLRYKAHLNVEWCNQSRSIKYLFKYINKGNDRVTAEFYKSSTDVDGVEVVDEINMYYDCRYVSASEATWRLLSYDIQCRTPEVERLSFHLRDSQTVYFQDDEDVESILNRQIFGQNMFTEWFEANKKYSKERLLSYIEMPNKFVWKKNVRQWQPRQRGFSIGRIFYVPPGTGELYYLCCLLNIVRGPQSFQDSKTYNGTKYNTFKDACYAHWLLDDDLEYIDAIKEASQWSTAHSLRKLFVTLLMENTMGRPEYVWNEVWTYLVEDVQYNRRKVLNQQYVFLNDQEKKDFALLQLEKLLLLYNKSLKDFPDIPLLSEELSVRADNRLILEELSYDRDSLAKESELLHSKLTDEQRSIYDAVISDVYSNKDGLYFVYGYGGTGKIFLWGALSAYIRAKGQIVINVASSENLRLNSMQCRLDQQRMGEFANWLASIGDGTIGEDNDGYAEVDIPSEMLLKSNGDPIATIGNITFLDFNGGEGKTYYSSDTACKADGSSMVGSPVMLMRNIDHSLGLCNGPNDGTRVLIARMTITPSDTRLPFTFNRRQFPLIGCPEPRLRRNLFPPLHQKASAGRRKKRDLRFGGDHRPASFVGRPPWSGERSKVRRQSRVLDSATSGGSRRELLFLRFFEAVLALLAEINCVDLNEVNYGGVNVDNLDVNGDGEQNDNIDVGDEKDDDEKECENNDNQDQFQRKKRKWVSKAHANFSEVTSKDGSIKLQCIHCKTLLSKSSSAATSHLWNHLSRCIQKKLQTKNQKTLQFQNAKSKFETPPLSDGVDIIDGVMKSTKDWGIEHKIFTISVDNASNNDVAVRIAKETFSRSHKLLLGGKLFHVRCTAHILNLVVQDGLSTIKTVVDDVRNSVDLSINRVRLLKFSEIVHHLGNPVKKSILDCVTCWNSTYEMLVVVITVKDAFPIFAQRESSYKCCPSLEDWRKIEHVLSILEVFYEATQVISGTNYPTSNVFLGVVWRVNHVLNENEFHHEELIRDMIEKMKTKFDKYWGDCNLLMSIGAILDPRYKMRLIDFAFNQIYNANDARINVMKVRDALHDLFYIKK

>IbDExDH125

MNPVNAHERPRRVPRTRNQWDDFYVKFNEEKKRKRRKLSESGPSTDRVGGDGSERGKAEKNGVGDDQVEVVVGEERKGSRRSGEAQKASSLGRNKEMGMADIEFVDDFVDKEKEMMDVKFVEDLVKGEGEKEMEMMDDEWKKEKGKIRKERKRRKEGRKEEGEWEGEGDKLESENDTKDLAKDLAVESENDTKGLPVVESASSDDDVIFIGETYPLYKNRTNRVEPISVSDDKDDERKGNSQLVLVGEPNPTPNVILLKPGSASGSKVGSVDGRGPEVIPYDDVASSTSKRRVDSSVSLSSTDSSDFDDFESSSSSSSSEDDDSDDGDFSFTVPSSVLKRADGKGKAAEAGEKRKGIVSRLRSSSTPKPLNKDERKKDEDEDVGGGEDGEQKQTTIVKKRKQRARGLNVKDILLNTVLEKESNLNERLQRPEMNAPPLPLKFRFEDEDEEPPVKEEWEVEVDNLFADLDMGRLQTENGSSAPQTVEKENAKADEANDCCHPEGHHCILDEQIGILCKHCLVVFLEMKHLYPDFAMKSTQRYERRFVDRSEHSEDAEFELGNIPVNNCARGVSGKTVWDLVPAHIKERMYQHQIDGYEFMWKNIAGDLEIENLKVLPSDKGKGCIISHAPGTGKTGLSVVFLQAFMKLFPMCRPVVIAPRSMLLTWENEFVKWDADIPFHNLNNPKLSGKENVTDVQGSNWKIFKRKSKGRELNRTLKLYSWANGSGILGITYRLFEKLAGESAEDEKVRRILLKYPGILVLDEGHTPRNDESLMWRALSKVETPLRIILSGTPFQNNFQELYNTLCLVSPEFSVPTPSSTRNFLQKGKAVRNKWTSITSSIVNDENGRRIEELKAMISPFVHVHKGHILLEKLPGLRDALVHLKLTEMQQRLLDLVSKKNFIEQDNLMTLISVHPSLAPENICKNGEKDLELDPTAGVKTNFVFELVKLCSAHGERVIVFSRLIEPLSLIKQQLLHHIKWSENEEILYMDGKIDAKYRQDRISAFNNPASVAKVLLASTASCCEGINLIGASRIVLLDVVWNPSITRQAISRAYRLGQTKVVYVYNLISSTFEARKYECQARKDRMSELVFSARENHSRTENPSAADVILEAMAENERLRGMFELIVHQPKTQQMLLADPTEGPKLADQP

>IbDExDH128

MVVVVVEATVVDVDGSVDDGGGVVAVGGVSNKPSALILCKPLLHVQNLGFCFIRPLQHSPTVNKCPRSVLPVESQISDEGEDDEEEEDEEEEEEEEDDDADDEEAAEEYGVISAEVSDGGEESEYESFDAANSIDVAREDKLKFEEFKWQRVERICRDVREFGEGIIDANELASVYNFRIDKFQRLAIQAFLRGSSVVVSAPTSSGKTLIAEAAAVATVAKGRRLFYTTPLKALSNQKFREFRETFGDSNVGLITGDSVINKDAQFQENNSFGSCPDLSQHADHLLTDHRMLNGLVGMVSSDSGLLHVDVIVLDEVHYLSDISRGTVWEEIVIYCPKEVQLICLSATVANPDELAGWIGQIHGRTELVTSLRRPVPLTWHFSTKPALLPLLNEKGTSMSRSLALNYLQLDESEATLQKGERSRRRNSRKRGSDVNPLSKNEISALRRSQVPQIMDTLLQLKARDILPAVWFIFSRKGCDAAVQYLENWLAAHHAGCLPLWKSFIEELFQRGLVKVVFATETLAAGINMPARTAVISSLSKRGDSGRVLLSSNELFQMAGRAGRRGIDELGHVVLVQTPYEGPEECCKLIFSGLQPLVSQFTASYGMVLNLLAGAKVTRGSMESDELKISRGGRTLEEARKLIEQSFGNYAEKRLRTELRRRMELERMFSLKPLLKELEDGHLPFVCLQYNDADGVQHLVAAVYLGNVDALSASKLKNVVHHSDLFAINMEVESNENGGIEGEDNSKPCYHVALGSDNSWYLFTEKWIRTLYRTGFPNVALAHGDALPREIMTELVEKGDMQWQKLAESGFGGLWCMEGSLETWSWSLNVPVLSTLSEDDEVLEFSQAYKDIVECYKDQRNKVSRLKKKIARSEGFKEYKKIVDMAGFTEEKIRRLKVSNVIHESRALDINTHVIFPLGETAAAIRGENELWLAMVLRNKLLLGLKPAQLAAVCGSLVSEGIKLRPSKNNSYIYEPSAIVLDVINLLEEQRTSLLELQEKHGVRIPCCLDSQFSGMVEAWASGLTWKEIMMDCAMDEGDLARLLRRSIDILAQVPKLPDIDPLLQSNAKSASSVMDRPPISELAG

>IbDExDH140

MLPMDHPTMETNQQESDKIDKAIDDEKVSVEEQEELDAPVLQEVSTLELNTQQWGEIESEDDDHIEIVWEDEKPTQGKTLTPTLIESSYNVCEDDSLILHDNFDDCDKDSFCSWESDSLQFCDDLGSEDDIELLGIIEGERERRERIVTIDNPKEQEWRENALDEIDGGKSVHWETYKDKFLGDGTYDEKGEEGRSILKYLRIVPCAYLLINYVFPFFAFVFKDVLHLGAFLEAKVHGTLVMTYELNRIKHGRQKPGDEEGDRDHACSAWSQAWNLSSAHRTTTRQRRTKAKRAENEEAVKHEFPAACGGGNIQVVSKPSGSESLGDSGSPSNSSISPSLNRKDEIRENGVQSLPNRTKHVRLPPILTRNGGIVFTLGRNDLARTRYFNSTVRSSEMVTVVLPNSPNRIDLENEKPQFLVHSNIQSPGSGNGLQQSNHYVFGVFENSINSPLSIITHKTGSAELTQVRVPFNDLTNGKYDGQCSLWTPTHTNSDRLRSNLSFDYCRNLEDEFAQVLQETTPVTCGFKVTYHNIGDPTNICEHCNAIFWFEERVNKSVRNRTPKYSSCCGHGKIKLPKMRIPPKRIFDLFFTKGQNQKEFLQHIRRYNNMFSFTSLGAKVDKSINMGGLKRINELNPAYLLCVSILFRMAKASEEDIQFNVTRNQHGGGRVRVSQREFFAFRIHERLNEVSTMLYARRLFQQFLVDAYTMVESSRLLYIRNNQKALRCEAYKGLSDALTRGEVETSKQGKRIILPSSFTYGARIFKLKLDSLVKEIRSGNLFGVVTTALHIDMPTKFVWKKNIREKWRSERLNYDRVALRDESELMEKQLADEQTIVYDTILHEIGNIMGALPYLGIRCGIASLLLPGGRTAHSRFAIPIGLLQKIPHRHHSGKRIAVNCQVKLIIWDEAMMHKLLPRHLICNTDFFLVSDRDKSDPYYYLPVSHINTVGQYSGTRSEHCRIPRGVEHETTLACIAGAVVGAMNEARSRTCVYILGRPEGPLGVAIKGAFNAGRRTFGYFDPTVAKEAEPRPLSCFGLPLGSVNNRLRSNYTVCTPIGIPHPTSYILLTNGLKPAAVWRSYNIHIATTGHLARGIKPVGELSRKLLNSLETKGLKT

>IbDExDH139

MGAQVDSQAAAGGVGGGSASASLASPVAADQDHIESTKTLICALNFLSRNLPLPQDVFDAVSSIFRAGEDDATDDCAAGEADNGLHKTSSHIGSGMATYGDLMADFEDSLLKEKSSRASGSLLKRSKEIRNQSLIHSRLTELEELPTSRGEDLQSKCLLELYGLKLLELQRKVRSEVSSEYWLRAHCINPDKQLFDWGMMRLGRPVYGIGDAFAVESDDPLKKKRDAEKKRQEPRVETKKRKFFADILNAARELQLQVQAAQKRRKQRNDGVQAWHGRQRQRATRAERLRFQALKADDQEAYMKMVDESKNERLRLLLQKTNDLLGRLGAAVQRQKDADHDGIEPLEGLDADLTASKTETPGHSVPEEDEDIVDGESTRDGKANDLLEGQRKYNSAVHSIQEKVTEQPTMLQGGELRPYQIEGLQWMLSLFNNNLNGILADEMGLGKTIQTISLIAYLMENKGVTGPHLIVAPKAVLPNWINEFQTWLPSAVTVLYDGRLDERKALREEYSGEGKFNVIITHYDLIMRDKAFLKKIHWNYLIIDEGHRLKNHECALARTVAGYRIRRRLLLTGTPIQNSLQELWSLLNFLLPNIFNSVENFEDWFNAPFSDKCDVTITDEEELLIIRRLHHVIRPFILRRKKDEVEKYLPGKTQVILKCDMSAWQKLYYHQVTEEGRVGLGTGTGKSKSLQNLSMQLRKCCNHPYLFVNQYNMWQKEEIVRASGKFELLDRLLPKLRKAGHRVLLFSQMTRLMDILEIYLQLNEFKYLRLDGSTKTEERGSLLKQFNAPDSPFFMFLLSTRAGGLGLNLQTADTVIIFDSDWNPQMDQQAEDRAHRIGQKKEVRVFVLVSVGSIEEVILDRAKQKMGIDAKVLASLFVIDSVFGIACAAQDRREMLEEIMRKGTSTLGTDVPTEREINHLAARSEEEYWLFEKMDEERRQKERYRSRLMEDYEVPDWAYVQADSNQGKGKGFLHDSANLTGKRKRKEVVYVDTLSDQQWMRAVENGEDFSKHSKKKRQEHQPAADNNPLPSNTREHQQQSARIDALPNKWSDQQPVPNDTSLSINTGGEKKAQDTKSETVSLVNEATSEDTIGTTSKRFKPVAAAPAPTPSQRDEYNSLTGNFDGLTWKALKRKRSSLA

>IbDExDH137

MGASGSEGFGSIYPTPIAARFPAVDSFPKGRKRMKTSEVPTNQISSLCWREEFEERLLKRSSGILDYSDPYSISNLWESLECGKFGSVTKEIEELMAQSRCYIDSFYARDPTLPYKFLELEKNHITENKGHQISTSVIDLEDERVARSVPVARFVPPAQLVPSAGPLLILDSDDEDNKKPNCTFEGIPSINTVGGSYLKDHLVQDSPGTKTPRGSANLAFQTEKIKDKGMYVGVEDDSETEDGNDANFDGLDDIWNEMSFAIECSKDVTVDASSNKDKAEDEDEDEECEHSFILKEDIGYVCRICGVIKKSIESIIDYQYSKSSKNARTYRYEGRTTKDSGPRGCIMAHAPGSGKTFMIISFLQSFMAKYPFARPLVVLPRGILGTWKKEFLRWQVEDIPLYDFYSVKADNRAQQLEVLKQWAGERSILFLGYKQFSVIVCDNEGSRAAIACQEILLTVPSILILDEGHTPRNQDTDVLTSLEKVQTPRKVVLSGTLYQNHVKEVFNILNLVRPKFLKLEDSKAIKRRILSRAVISGKRNLIKKGSDNEFFGLVDFTVILKLHPKQKIEVAGLKNLRRKFKISAEGSALYMHPQLKSLSKNSVKERIDEEKIDMIVDNLDVREGVKAKFFLNLLALCESHKEKLLVFSQYLLPLKFLERLTIKFKGYCIGKEIFMITGDSDNEVRESSMERFNTSADARVFFGSIKACGEGISLVGASRIIILDVHLNPSVTRQAIGPAFRPGQEKKVYTYRLIASSTPEEEDHTTCFRKESIAKMWFEWNQYYGLDDYEMEKMDPKQCGDEFLETARFSDDIVALYKR

>IbDExDH136

MKFRWVMTIAMQHYKYVQKKLVLKASMSHTLIGDLEHPNKDKSGSDLTMDSLIGSHNHHRVGRVLQTSLPTPNTDFTNENDGNNEPHIQTALAPSTTHTAVEQVGFQKHVLAKYRATTICFTSMGGKVDGDRSTMVVVHRINGQNFHLIGSLLPMDGAQPKFAQLYIHDTENEINNRVDSIRHEGGSSSLHIDVALARMLGPIICLLISRFFAIVGDLDPDMGFRDILVESKTGKLKRISELNPAYLPMQYPILFPYGEDGYRDDIHLRQIVVNYLDQGNAFNTTREYFAFRLHERRSELSTLLHSKRLFQQFLVDAYTMVESGRLMFIRNNQKALRCEQYKGLSDALFRGDVDPSTQGKRIILPSSFTGGASMESSPDAIDKFISAEIPDIQIDKDYYKAVEEFMVHGPCGVARVNSPCMVNGRCTKYFPKRFVDSSTFDQDGYPIYRRRDDGRTITKNGIQMDIRYVVPHNRYLLMRYRAHLNVEWCNQSRSIKYLFKYINKGNDRVTAEFYRSSSEGNAVEVVDEINMYYDCSQTVYFQDDEDVETILNRQTLGESMFTEWFEANKKYSEARLLSYIEMPNKFVWKKLRGPQSFEDIKTYNGKEYNTFKDACYALGLLDDDLEYIDAVKEASEWSTAHSLRKLFVTLLLANTMGRPEYVWNEGGLYFVYGYGGAGKTFLWRALSAYIRAKGQIVINVASSGIASLLCTEDAQHTLDLQYLFQSMKSQLAIFIKGSRQDIVSSLSIHHTFGSIAKLCSNKEFEVMMYYGEDNDGYAEVDIPPEMLLQSNGDPIATIVNSTFPQFNGGIIDGTCFHSSAILAPTLQVVNEVNEYMSDLTIGWVAVMLMRNIDHARVCNGTRLVVTRLAEHVVEGSILAGPNAGTRVLIARMTITPSDTRLPFKFNRRQFPLMLSYAMTINKSQGQTLSNSSKTLLRKPHSFAVLLWLHLQTREMGKVKGKHRLDKYYHLAKEHGYRSRAAWKLVQLDSKFSFLRSSQSVLDLCAAPGGWMQVAVERVPVGSLVIGVDLDPIRPIRGAIAVQDDITTPKCRATLKKLMAENQGRSYITFDLVLHDGSPNVGGAWAKEATSQNALVIDSVKLAAELLSPKGTFVTKVFRSQDYSAVLYCLRQLFEKVEVDKPLASRSASAEIYIVGFKYKAPAKIDPRLLDFKHLFQGGKEPPKVIDVLRVTKQKRHRDGYEDGASILRKVCSAVDFIWSEAPLDILGSVTSISFDDPACLPIREHTLTTEEVKALCDDLRILGKQDFKHILKWRMHIRKALSPSEKSITPSISVEPESKEDEDEKILNEMEELTFAIERKKKKEKRLQAKRQAKEKARKALGVQIDATGDGYGDQDLFSLSSIKGKKDLVAVDNDEYLEPGEGNSEDSESDAEAQEDTSSDVDSGRRAYDEKVEELLDEAYESYVARVEGKTKQRKRTKRAYEKDDELLEGDNDDAMVHSDQDMDNDQGEHELNPLVVPLEDAPTQEEIAAQWFNQDVFAEPDEQDILDKYDSEDEMQIDEPGKSAKKSRQMGKDASEKQTIGVTRKSKSSVLQVPSSEAAEDFEIVPAPPTDSSDSSSSDDSDDDDINNKAEILAYAKKMLTKKQREEMLDDAYNKYMFHDEGLPKWFADEEIKHRQPIKPITKEEVAAMRAQFKEIDARPAKKVAEAKAGKKRAAHRKLEKIRRKANSISDQADISDRSKTKMIDQLYKKVGPKKPEREYVVAKKGVQVRAGKGKVLVDRRMKKDARKNGINKKGKGKNGKQKGKGSMKAKGKKGEARQNSRK

>IbDExDH130

MATLSSSSSAFIPTQRHHRLCNHTSQSATLLFTKPSNFSLLPFPSVSRCRHLRRRRSTPSSSPLVVCAASLKEKIDGLNKTWSNITSLNHWVVREYGRLVNSVNVLEPQFQKLSDEQLSAKTLEFRRRLREGESISHIQAEAFAVVREAAKRKLGMRHFDVQIIGGAVLHDGAIAEMKTGEGKTLVSTLAAYLNALTGEGVHVVTVNDYLAQRDAEWMGRVHRFLGLSVGLIQTRMTADERRLNYGCDITYTNNSELGFDYLRDNLATSREQLVMRWPKPFHFAIVDEVDSVLIDEGRNPLLISGQASKDAARYPVAARLAELLMRDLHYNIEFKDRSVELTEEGIFLAEMALETNDLWDENDPWARFVINALKAKEFYKQDVQYIVRNGKALIINELTGRVEDKRRWSDGIHQAVEAKEGLEIKADSVVVAQITYQSLFKLYPRLSGMTGTAKTEEKEFLKMFKTPVIEVPANLPNIRKDLPIQAFATERGKWVHACEEIEFMFRLGRPVLVGTTSVENSEHLSTLLRKRKIPHNVLNALPKYAAREAEIVAQAGRKNAITISTNMAGRGTDIILGGNPKASMLAKEILEDSLISSLTQDAPQTDIDGELNLKKVLSKIKVGPSSLALLAKTALMAKYVCKNEGKKWPYEKAKSMISESIEMSQSMELEELQKLADDQSEMYPLGPSMALTFLSVLKDCESHCLNEGLEVKRLGGLHVIGTSLHESRRIDNQLRGRAGRQGDPGSTRFMVSLQDEMFQKFNFDTEWAVKLISRITNDEDTPIEGDALLKQLLSLQISVEKYFFGIRKSLVDFDEVLEHPSKWFLGKLLRDFTGFAGKTLNDSFSEITEEALLDSLLQLHDMGSVTIDDFCVPNLPGKQNPFRGIRGKTASLKRWLAICADDSTKDGKYRVTVNFLRKYLGDFLIASYLDVIQESCYDSEYVKRIEASVRLLGPGKVLVLQMQPMCNFASFYLVRPLIDLRAVLLKTLDSFWRDHLVNMNRLSSAVNVRTFGLRDPLEEYKIDGCRFFISMLSATRRLTVESLLRYP

>IbDExDH145

MAPKKKQQQQKQKQKASSSSSSKGKAPSSTGPKLQISAENESRLRRLLLNSGRSTAPAPVDDSLSQAQKAKKLRSIYEKLSCEGFKDDQIERVLSALKDGATYEAALDWLCLNLSGDELPLKFAGGTLNSNEGSVGIISTAREDWVPSTDSSAAGTREEISEVFITTKEQRVNETLDSVERSQADWIRRYMEQQEEDESESESGLFADASSKQSLQSRRSQETIVQEFHTARLEAIRAKETGDKKGQEQAGRTIRKIKQEISALGLSETILESEYESFSHQALQDLSCPSMSSENLEADVVTLQNGEDCIASSLHELELNVDKKVAGLSAEEDSVSVSIPEKTESEGESEDVELGSFLFEEASAAELPAEVLERQKKEKLRELLSEKNLEKLEGIWKKGDPQKIPKAFLHQLCQKSGWDAPKYTKALGKRNISSYTISILRKASGRGKSRKAGGLITIELPEQDQTSSNAEDAQNMVAAYALHQLFPDLPVHLPITEPYASIVLHWKAGESLTDVVEDQEERRASFVNSLLNADGSGIIVPLSVTNNPTENKIQQPQVTEDKTTSSDSKVKKVNQRKESESVYLRQEQENKKKMKKYQRDMFYHTLSKLKMFRCAMTGQFPSYPESPYPDSLFTLFLALQLSSMEMAVGILCSLIMQGDSSLLPCPALKADMLKSRASLPIAELKDDILHSLEKSDILVVCGETGCGKTTQVPQFILDDMIESGHGGYCNIICTQPRRIAAVSVAERVADERVESSPGSYDSLVGYQVRLDSARSERTKLLFCTTGILLRMISGNKDLSGITHIIVDEVHERSLLGDFLLIVLRNLVEKQSAHGTPKLKVVLMSATVDSHLFSQYFGHCPVITAQGRTHPVSSYFLEDIYENINYRLASDSPASMNYGAPTKEKNAPIGNHRGKKNLVLSAWGDESLLSDDRVNPYYDPSIYQTYSEQTRKNLRRVNEDVIDYDLLEDLVCHIDETYPDGAILVFLPGVAEIHVLLDRLSASYRFGGQSSEWLLPLHSSIASEDQKKVFLRPPENIRKVIIATNIAETSITIDDVVYVVDCGKHKENRYNPKKKLSSMVEDWISQANARQRRGRAGRVKPGICFCLYTHYRYEKLMRPYQIPEMLRMPLVELCLQIKLLSLGNIKIFLSKALEPPKEEAITSAISLLYEVGAIEGNEELTPLGYHLAKLPVDVLIGKMMLYGAIFGCLSPILSISAFLSYKSPFVYPKDERQNVERAKLALLGDKLGGETDSCDSNLQSDHLLMMVAYKKWEKILRESGAKAAHQFCSSYFLSNSVMHMIRDMRVQFGTLLVDIGLIDIPKNFQIAGKRKEKLDSWLSDASQPFNMHANHNLILKAILCAGLYPNVAATEEGISTSALGSLKQNTGPTARSQPLWFDGKREVHIHPSSMNSTLKAFQYPFLVFLEKVETNKVFLRDTTVISPYSILLFGGSINVQHQSGIITIDGWLKIRAAAQTAVLFKELRLTLHGILKELIQNPQSASTKDNEVIRSIIHLLLEEDKPSM

>IbDExDH134

MERLSSAKMKAWPKDDFTDIVISWTLEDIFNQNLFKDQVEKIPETFNSADNYLCSFLFPLLEETRAQLAASMEVMDRAPFAEVIALDAPYRTLPGDIVVISDNRPVSASDLDRARSNWTLGSVVNIVDDEDDGANISTNFRVKMPTDLAANLEKYEGFHIVFLENITTHKRIWNALHMRKNMNIIDSVLYTNGEVSGIVGLLICSVLLTLIFTSAHSIYTAEKKCYLCSPHPDNIGCTDRIGTGMFSKLNESQANAVLTCLERVKCDQISHVDLIWGPPGTGKTSTISILLYMLLKMNCRTLICAPTNVAITQVAARVIKLVQESFKGESSEKYLLYPLGDVVLFGNKDRLKMGGDIVEIYLDNRVDRLVECLGPLKGWRQCIDSTVHFLEDCVSDYDIFVENELKMRELNVKGETLKDSIKPTSFLKFIKLRFEEHMRSAKLKEVFSQPVSSKPFVNTSSLLCLRIQCISILKMLLHSLGVLELPSALNKASIKEFCLRSSSLVFCTASSSYKMHSTEMEPFNLLVIDEAAQLRECESIIPLQLPGLKHAILVGDECQLPATVCSQVCSEAGFGGSLFERLSSLGHSKLLLNVQYRMHPAIGHFPNLSFYHMQVLDAPNVRSKAYERQYLQGNMFGPYSFISVPSGKEESDDFGHSKRNMVEVALVIKILQNLYKFCSSRKKLSVGVISPYTAQVVAIKDRIGRKYDNLNGFSVKVKSIDGLQRTNVALTRARHCLWILGNERTLVDSNSVWEGLVLDAKDRQCFFCAAEDSDLLKTVIDVKKELDQLDDLLNADSILLKNQIWEVLLSDNFRKSFKNLVSSRLRMAVLNHLCKLASGWRPKRKCVDLVCESSFQVVKQFKVEGYYIICTIDIQKDLNYKQVLRAWDLLPLDEVGKLVRRLDSIFAMYTDDFIDLCKLKCFDGVLEVPKVWPGSYDLVRFKNPSERVADNSNDGVVEGSSYAENSRVSESLLLMKFYALSSGIVNHLLSDNHGEEIDIPFEVTDEEKELIQSGRSSFILGRSGTGKTTVLTMKLIQKEQLHQLALGGVMKVETNEISKYAGESSFSRAEIAMMSQSEAEAKKPTLRQLFVTVSPKLCFAVKKQVSNLKCFVDGGKFSAGNSVMDMDDLDGFSHFKDIPNSFVGIPDSKYPLVITFHKFLMMLDGTLWPSYFDRFHDTSTLSLNRISRSVTLDTFIRGKEVNFDRFCCLYWPHFNSELSKNLDPSRVFTEIISHIKGGLQVCETDGAKLTPQTIARGVDFRFEDVRTLFYEEFIMKLKGDGLPARKDKGHLAGVSCLLQNFRTHAGVLRLAQSVIDILYHYFPLSIDALAPETSLIYGEAPVLLKPGSDENAIVTIFGNSGSMSGKMVGFGAEQVILVRDEAAKKEVSDFVGKQALILTIVECKGLEFQASEMARACCFFALFFQFLSGCSLFHSDVMLYNFIGSSPLRNHWRIVYEYMKQRDLLDSEFHQCFPCFSEARHAILCSELKQLYVAITRTRQRLWIYESVEEFSKPMFDYWMKMCLVEVREVDDSLAQAMRMASTPEQWKSRGIKLFWDKQYEMAIMCFERAGESKWEKRLKLPESGKLHYRKRIQNPLSSIPPCGKLLKYLSLLDGSIYLNKCGEQEHEKAAECYTLARCYETAAKIYARQNCFSECLSVCSKGRHLYDMGLEYVEYWKQHAQERRKEIDGIEQKFLESCASDFFKCNDKKSMMKGRVGNYAEAAELAQLKGDLQREVVLLAWPLKSFESKHELLKKAISFASNESDAFYESVCTEARILSHDPSSLCELRRTLSATHKCGSLRGEILCLRKIIDVHTQIDVTKYKWEEKFPVDLKYPDDTLFCDQLSKDFGKYKGYGEFCLNYFGVRRQFTDMKVTYLLANPDAEWVKVNEVLAVGLNVLETIESLYCFSTELLSRFSQSMYLVNIYIIAKDMPCKKYDAKLRKFFQLSSMEYFGKVFPLDYHEALEENIISLRGTEVSRSLLEEFIVNDLSGKGKLTHGQIGRLMMIWLGSAEPSDELCNKIFERIRDGSNWKAFINILRSVREPLNQSASADSQEASSVDPYNLLVHRFHEALNETYQINWQNFEDYISPHCFVYLVERFLILSFCPSGFFYTTKSSFLEWLIFQNPGVSVIAGFQTSCPSSEIFYNSVTSMVHWLLFHNLETAHWIARSKIQSSNYHKLLVLRLVVILCLLCMNSSSQEPWIALFDALKSPYITSELPREFNKVFRRGGKHTAFVDRVKISEALRVIGNPALFVDFKQNTASSVCPNATYLGIGPNSCRADIMEMLFPRKSVTSPVQKSMKNSCCLLPLIADLDIETSVLPSPDDASAQN

>IbDExDH143

MACLSATLLNSLSWTARHTLLKASLSSSYIEVETAKKEGVLTLQELCQGHVPEHIIGRMEEVGYVVPTKVQQEALPVLFAGCDCVLHAQEVARNVPVFGLQNPCAKLLKLFHFTTISHFCICSGKTLAYLLRIFSVINTQRSAVQALIVVPTRELGIQSEELNSGQKSCTIMALLDGGTLKRHKSWLKAEPPTIVIATLGSLTQMLEKNILKLDAMQVLVIDEVCSKGERYLTLLSLLKSDEPHSAIVFVGVQSEKSKKAGDPPPTTLLLNFLKSSLMGFSEISLLEEDMNFNQRAASLTELQQGSSHLLVATDIAARGVDLPGTTHIYNFNLPKDAVNYLHRAGRAALEGCMAEVESSRKGRKWQKERNGDCKRRPLNVEPLKQLKLSIQTTLFPSLGWINLLRSGQ

>IbDExDH131

MATLSSSSSFIPTQRHHTSQSATLLFTKPSNFPLLPFPSVSRCRHLRRRRSTPSSSPLVVCAASLKEKIDGLNKTWSNITSLNHWVVREYGRLVNSVNVLEPQIQKLSDEQLSAKTLEFRRRLREGESISHIQAGLSLEHYMSLHMFHLHLWHNSFINLTGRVEDKRRWSDGIHQAVEAKEGLEIKADSVVVAQITYQSLFKLYPRLSGMTGTAKTEEKEFLKMFKTPVIEVPANLPNIRNDLPIQAFATERGKWVHACEEIEFMFRLGRPVLVGTTSVENSEHLSTLLRKRKIPHNVLNALPKYAAREAEIVAQAGRKHAITISTNMAGRGTDIILGGNPKASVLLFFIQFQYSRNEQEFAEAWNRPMALHSYQKDFESHCLNEGLEVKRLGACMCIHARRIDNQLRGRAGRQGDPGSTRFMVSLQDEMFQKFNFDTEWAVKLISRITNDEDTPIEGDALLKQLLSLQISVEKYFFGIRKSLVEFDEVLEAACGLNIKLPMEKIEDIDAGDVYNELAVMEYAEDIYKFYNKAEANHLCRRHLQVLLKWKIEDLEVYKSCLDRDGFVMGDGVLLLEELEHITLTFTGVVLCIERAIANSGICRDDVSYINAHATSTQAGDLTEFQALLRCFGQNPEIGRLLREEFEELHTEYERKVLYETCEGSPISKGTLQPDMWGVTPSNQWDWVALRANIEKNEVRNSLLAAPMPTASTSQILGNNECFEPHTSNIYSRRVLRNVLVLRMWMELLLQMEVPRKPPSQKRATMKPIKPEAVIVISPDAKEEAACGLNIKLPKEKIEDIDAGDVYNELAVLILADAMGLGKTVMTIALILARLGKGIPDNQELAEDMAITQHSRNRRIKGGTLIVCPMALLGQWRCDELEAHSKPDSISVFVHYGGDISNNPKVIAEPDVVLTTYGLLTAAYKANNVQDLYSLLCFLHVEPVQLGMRPYENVDQRALKLIKAILRQLVLRRTKETKDKEGRFAVEGDVAGGTRRRVVKQKRLPKSKIEKLLDVFRQVMFASGFPGILTSFLVEGIASLALQSWKPFMQRLIRAHIMLLDTKVHFAVLDKTSNAAKEILKVVPLLNGYGAFSLVFPEHQITPNPRSAENITLAIGAPCSHEHKYRQWSAAISLGLISSFLHVTDRKQKVEYINALLEVLSVSKSTLVKGACGVGLGFSCQDLLTKSGGEDNFHLNDFDSNITPKFLGQCSDDFEEDVWGVAGLILGLGSSVGAIYRAAPIVMAGFLSQSRTDGCTLHQSLLMASCIGAGSLLGTILNGSLHSMKGEHVKDLPALFRKSYSDPNPPLIHLGATIGAVNALGSGAGTLIQDHPSFSSHATNNQKESSSVNVPLLSSPALEPDLTSLVQEIFLVAQNPDADQLQHTVLKLSLWLSHLKHPGIEDIDAGDVYNESAVVEYAEDIYKFYKEAEANHLCRRHLQDIMLCGGSDSVIIPTGVGGFIACRALSQRNNDPTTALYLWIDRDGFVMGEGAGVLLLEELEHEVSRKEVQQSMLNFSVEEPHPEVTGVVLCIERAIANSGVCRDDVSYINAHATSTQAGDLTEFQALLRCFGQDPEELGFDYLRDNLATSREQLVMRWPKPFHFAIVDEVDSVLIDEGRNPLLISGQASKDAARYPVAARLAELLMRDLHYNIEFKDRSVELTEEGIFLAEMALETNDLWDENDPWARFVINALKAKEFYKQDVQYIVRNGKALIINELTGRVEDKRRWSDGIHQAVEAKEGLEIKADSVVVAQITYQSLFKLYPRLSGMTGTAKTEEKEFLKMFKTPVIEVPANLPNIRNDLPIQAFATERGKWVHACEEIEFMFRLGRPVLVGTTSVENSEHLSTLLRKRKIPHNVLNALPKYAAREAEIVAQAGRKHAITISTNMAGRGSDIILGGNPKASVIGTSLHEGLEVKRLGGLHVIGTSLHESRRIDNQLRGRAGRQGDPGSTRFMDEMFKTPILISLQDEMFQKFNFDTEWAVKLISRITNDEDTPIEGDALLKQLLSLQISVEKYFFGIRKSLVEFDEVLERRRRRKQRRNETSTLPRLVTSSGDQPGVRKAWRLFPQGFRQKFVLFDLALVSPFHHGVTAARLQQLFRFCPPLFLSFGAAENSNSNGDLWTSIFSGEGSCGEQWWPAKISGSESWVFCVDGQRVSRECGLAYARSYWPTDEEECGLIMVWGEWRPCGVWPCFFPLEALLVQQSELCTDLAQRDSRCMQGVVDEISFNYVDPQKHPSKWFLHKLLRDFTGFAGKILNDSFSEITEEALLDSLVQLHDMGSVTIDDFCIPNLPGKQNPFRGIRGKTASLKRWLAICADDSTK

>IbDExDH132

METVSSSSPKGDFTDIVFSWTLEHIFNDNLYKDQVEKIPETFTSAEHYLGSFLYPLLEETRAELAASMEVMDKAPYAEVIALDGVKRHGEPLYDVNVDVWKNRVRDEREPYRTLPGDIVVIADNRPVSASDLNRAGWNWTLASVVSIVDDEDDDANISTNFKVKMPTDLTANLEKYEGFHIVFLENITTHKRIWNALQVRKNMKIIDTVLDAYGELIPFIKYVFINLFILVIFVALKAEEKCCLCSLHADNIGSTESIGTSMFSKLNESQANAVLTCLERVKCDQISHVDLIWGPPGTGKTSTISILLFMLLKKNCRTLVCAPTNEAITQVASRVVKLVYESFKDEPLKENWLCPVGDVLLFGNKDRLKLGVGTEEIFLDYRAEKLCECLGLKGWRHCILSTIHFLEDCVSDYEIYVDNELIKMRELRDKGKTLEESAHPKSFAEFIKSCSEDTSSLVCLRSQCLSLLKTLLHSLGELDLPYASNKYLIKDLCLKTSTLVFCTTSSSYKIHSIKMEPFNVLVIDEAAQLRECESVIPLHLPGLKHVILVGDECQLQATVHSKVSYEACFGRSLYERLSSLGHSKLMLNVQYRMHPAISYFPNVSFYHGQVQDAENVKGKTYERKYLQGRMYGPYSFISIPCGKEELDDIGHSRRNMVEVALVNKIVKDLYEFWRSTGQKLSVGVISPYTAQVVTIKDTIGRKYDNLNGFAIKVKSIDGFQGGEEDIIIISTVRFNSSGSIGFMKSLQRTNVALTRARHCLWILGNERTLFDSNSVWKGLVLDAKDRQCLFSADEDSGLSKTILDVMKELDQLDDLLNADSIVFKSQRWKVLLNDNFKRSFKNLVTSSMKMAVLNLLIKLAGGWRPKRKGVDLVCETSSQIVKQFKVEGYYIVCTIDIQKEAKYTQVLRAWDLLSLDEAGKLLKRLDGMFARYTDDFINLCKQKCLDGDLEVPKSWPASLDLVRFKNHGERLADSSNDCVVDVDFEIDGDLNGEVVGSSVIEVRADAENAIEGSPDEHDFHQAPNVKVDHDLSGRDVVSIEEPETVSNSEPDEPYVGQEFESEAAAHAFYNAYATRVGFIIRVSKLSRSRKDGSAIGRALVCNKEGFRMADRREKVVRQRAETRVGCRAMILVRKVSSGKWIVTKFVKEHTHPLTPGKGRRESIYDQFPNEHDKIRELSQQLAAEKKRSATYKRHLEMIFEHIEEHNQSLSKKIQDIVNNVREMESRDQQNQQNRR

>IbDExDH129

MEDVYGSSDEEYCDQVYNDDDSDYEGLYLDKDCDSGRAPSCKIITKDSLLAAQKEDLQRLMDLLSIKEYHARTLLIHYRWDVDNVFTVFVEKGKERLYADAGLTIECKDDCSLSESTADMTCEICFDDISAAMTTVMDCGHSFCNDCWTEHFIVQINEGRSKRIKCMADKCNAICDEGKIRDLVRARDPKLAEKFDHFLLESYIEDNKQVKWCPSTPHCGNAIRLDECDENCEVECACGQQFCFGCSSETHSPCSCLMWEMWMKKCGEESRSVDWITANTRYCPKCSKPVEKNGGCNLVRCICGQPFCWLCGGATGMNHTWDSIEGHTCGRFKEAENKKVIDSRKQIFRYSHYYSRYKAHTDSLKAEASMEQKLQEKVLNLELKGLASKDFSWVTNGFYRLSQSRQLLSYSYVFAYYIFGDELYENDMTQREKDIKQDLFEDQQQQLETNIERLSMCLDESFDDFPEDKVVQMKMKIVTLSGVIDNFCKKLYDCIESDLLIHLQSNHNVAPYSSSGAVKASELEDTFSTSVTL

>IbDExDH146

MAVDKSKLDEEHQACIVRFYKIVLSWDYLRILKDSDNYNKKGKKGGASGVREVKDTYSDVDDYIATFEPLMFEEVKAQIIQGKKDDEGGTEWTQAIVAECNELNGFHLPTVICADVESISQNDLLLLSNKKFEEGKPLPTAYAFAFVEHRQQDKVKVRMHLNGEYKQYNTDKIDACPRLLNMRPLISEIQKYLYVLKICSLSTIAREYVALWSISSLPFKDLILSAAESNSDNDDRAWKISKPLNEYIEANHNKSQLEAINVGLSRRTFVLIQGKIEYFKMWTRIFHPGQCTSLPSTNLYVVHGILQNLYLNDILQIGYTHWLRASPWLAGLNPREQEMPKDGDDDFFPQPAMSCRKYRVRVLICAPSNSALDEIVLRVLNTGIRDENDHVYNPKIVRIGLKPHHSVQAVSMDYIVEQKMAGMDMQTTSDKQKQGGTGKDKDSIRASVLEEAVIVFSTLSFSGSPLFSKLNHGFDVVIIDEAAQAVEPSTLVPLANGCKQVFLVGDPVQLPATVISTVAEKLGYGTSLFKRLQKAGYPVQMLKTQYRMHPEIRNFPSREFYDEALEDGPDVEEQTKRAWHNYRCFGPFCFFDIHEGKESQPSGSGSWVNVDEVEFVLTIYRELVTRYPELKSSSKLAIISPYRHQVKLIRQKFRETFGLDSEKVVDVNTVDGFQGREKDVAIFSCVRSNKDRGIGFVADFRRMNVGITRARASVLVVGSASTLKRGDKHWKNLIESAEQRNSFFKLQPLQKFWVMRVLAPPYQLRLLLCMEYMLQFGIEAMSKLKTTWARDSARQGMPEAHTGDMDTEMPVETTAVDAADQEQGDEPEWGDGGDEGMDGGGGADED

>IbDExDH142

MACLSATLLNSLSWTARVHGPHHLYPNRPNHTFVVSMNGMVHSMREHTLLKASLSSSYIEVETAKKEGVLTLHELCQGHVPEHIIRRMEEVGYVVPTKVQQEALPVLFAGCDCVLHAQTGSGKTLAYLLRIFSVINTQRSAVQALIVVPTRELGIQVTKVARVLYAKSEELNSGQKSCTIMALLDGGTLKRHKSWLKAEPPTIVIATLGSLTQMLEKNILKLDAMQVLVIDEVCSKGERYLTLLSLLKSDEPHSAIVFVGVQPPRLGASRLRRKEFSDGVFGISLLEEDMNFNQRAASLTELQQGSSHLLVATDIAARGVDLPGTTHIYNFNLPKDAVNYLHRAGRAGRKPFSDEKCYVTSIITPQEHKMEIFHNFFGKGEGKNYLKLVQLQVTSTSSYRCYVQQPWEGCMAEVESSRKGRKWQKERNGDCKRRRYGKPLEAVEAKHSNDVVSIPRVDQFAAIRRMTGVVKSVD

>IbDExDH138

MASIGDTMSALGILILLIPIVQSRCKRAEPSSSIRWLELELVKDGSARVRARARSNSRIDARARARQFSSCSSSARAARLFELEFEFDFELKFNIGKEAQPLPSAFRLCLPAIDHGPPKGHRRNSTASLRLRPLPSAFRLPSASRNRRPTDRSSTAEASVFALCHPPSDFPLLLSINDRRTEAAPPPPPPSPTGSARSRTATLAGMSTANSSVPQNPNNSSSVVGESNEAVQRHESRTTAIFDFLKQVKFELDFELEIELEIELELEQSSSSSSINGRARSSSSSSSSLSFLNELELEPPQVRARLGSISFDLRVAVWHSPLSHPVIMGASGSEGFGSIYPTPIAARFPTVDSFPNRRKRLKTNEAPINQIPSLCWPEELEERLLKRSLGILDYSDPYSMSNLWGSLECGKYGSVTKEIEELMAQSRHCIDSCYARDPTLPYKFLELEKNHTTEYKGDQSATAVIDLEDEHVARNVPVARFVPPAQLVPSAGPLLILDSDDEDNKKPNCTLQGVLSINTVGGSYLKDHLDSPGTKTPKGSANLAFQTEKIKDKGMYVGVEDDSETEDGNDANFDGLDDIWNEMSFAIECSKDVTVDASSNKDKAEDEDEECEHSFILKEDIGYSAKNARTYRYEGRTTKDSGPSENLFEPNKPSHEFELAEISAHPRHKKQMKPHQVEGFNFLLNNLVTDNPGGCIMAHAPGSGKTFMIISFLQSFMAKYPFARPLVVLPRGILGTWKKEFLRWQVEDIPLYDFYSVKADNRAQQLEVLKQWAGERSVLFLGYKQFSVIVCDNEASRAAVACQEILLTVPSILILDEGHTPRNQDTDVLTSLEKVQTPRKVVLSGTLYQNHVKEVFNILNLVRPKFLKLEDSKAIKRRILSRAEISSKRNLIKKGSDNEFFELVEHTLLKDENVTRKATVIQDLREMTRKVLHYYKGDFLEELPGLVDFTVILKLHPKQKIEVAGLKNLRRKFKISAEGSALYVHPQLKSLSKTSVKERIDEQKIDMIVDNLELREGVKAKFFLNLLALCESHKEKLLVFSQYLLPLKFLERLTIKFKGYCLGKEIFMITGDSDNEVRESSMERFNTSADARVFFGSIKACGEGISLVGASRIIILDVHLNPSVTRQAIGRAFRPGQEKKVYTYRLVASSTPEEEDHTTCFRKESIAKMWFEWNQYYGLDDYEMEKMDPKQCGDEFLETARFSDDIIGLYKSYFTDPRFVVDLQFDYLIISFVMYVEHWIPSVCHLAETWQWTSFDYVHLCYRTNTYMKIYASSIKPMASLEEWPLSDMDPPLPPHQWKIYASSIKPMANLDEWPLSDMDPPLPPQYTARPGRPKKLRKRKIKENVGSNSHALTEEAQDVIDEVVGHLQNTVESEYMEGDDSAFVEVDFLTQDGVPVYTPTWMMGVGSSLSGGNF

>IbDExDH144

MPMPMPTCCNSGIAAVPSGKGFFVGSPAFTPVNPIVNVVGSERLYGGFMMCSPKKPINMSTNVNVHPTPFEDNYVATKGTPLSATKNVASNPNSYALNVEIPPVTPLFVIIDVNMRDISSPFTQCSFNISNMSTSTSSSRQSLADVSNVERTLDPQCNRALLSTVRNSNKVSSRTDVRNLNSDYEHVASHNNGYGGRVDRPINTGNTPPVFRISGQNYHCIGSLVPGEGSTPKFAQLYIYDTDNEVNNCINFVRRDNNSCDIREDIVEKLKNMLDQNNVLVKCGAKMEGHIIYQALMRQIGYPHLFITFTCNPKWLEIKRYVAHRGLKPEDRPDIICRVFKMKLDTMIEDIKTQKLFGDICGVIYTIEFQKRGLPHAHILLFAKTINRANSASEIDAIISAEIPNPDADTEYHDVVGEFMLHGPCGQLHGYPIYRRRDNGRIIARNGIELDNRYVVAHNKHMLLKYRAHINVEWCNQSRSIKYLFKYVNKGNDRVTAEFMSSSTNGRNGDVVDEINMYYDCRYISACEATWRLFGYAIHYRTPPVERLNFHLEHQQNVVYGEDQTLDEILENQTVKQSQFTAWFEANKKYEDARSLTYAEFPSKFVWKQDLREWQPRKRGFSIGRLFYVPPGCGELYYLRCLLNLIRGPSSHDDIRTVAGVIHKSYRDACYEYGLLDDDKEYIDGITDSRYWASASALRRLFATLLSSSTISRPEVVWDAVWEFLAEDAQFHHRRRMNNPETVRSYGVGEIVIFVGKKPRLSKCHFRDETNIVYGKHVDSQELAYDKESLKIEHETLVTQLTDEQKNVYDSVMNDIDCNGGGLFFLGISSLLLPGGRTTHSRFAIPFSLNEDSTCNISQSSDLAELIIRSKLIIWDEASMTHKHCFDALDRTMRDLLRFVIPGSAEKTFGGKTVVLGGYFRQILPVIPKATRPIVVGATINSSYLWTNCKILRLTKNLRLRSLASKEDRKTVDWFSKWIANIGDGITGVVNNGLSEIDFPPRFLLNCGHDPIATIVESTFPSARYGMIDELDLEGRAILSPTLDVVDQINQYMCNMNTAEGRTYLSCDSLCKAESDGENLSQVHTPEFLNSLRLSRLPNHSLILKVGAPVMLLRNIDHSLGFCNDTRLIVTRLKDHIVEAKIVNGTHQGTKVLIVQMSLTPYDTRLPFKFQRKQFPLMLAYAITINKSQGQTLTHVGLLLKNRSLITSLPPLRICEQLQPTTKTEDFSSPATADKVLLPNSR

>IbDExDH133

MERVVFSWTLEHIFNEDMFKDKVEKIPETFTSAEHYQGSFFYPLLEETRAELAASMEVMDKAPYAEVIAIEHGPYKTLPGDIVVISHKRPMAVSDLNRAGWNWTLASVVVSWITDDEKDERRMVMMPISTNFKVKMPIDLTADLEEYEGFHIVFLENVTTHKRIWNALHMRKNMKIIDMVLYRNGEAEEKCCLCSPHSDNIGSTGSIGTSMFSKLNESQANAVLTCLERVKCDQISHVDLIWGPPGTGKTSTISILLFMLLKKKCRTLVCAPTNVAITQVASRVVKLVYESFKDEPLKENLLCPVGDVLLFGNKDRLKLGVGTEEIFLDYRAEKLCECLGLKGWRHCILSTIHFLEDCVSDYEIYVDNELIKMRELRDKGKTLEESAHPKTFAEFIKSCSEGTVSPLRRCMLILCTHLPRHFIQEKNFQAIISLICLLDSLNGMLFQEDMGSDELTSVFSQPVISDVSPESFADTSSLVCLRSQCLSLLKTLLHSLGELDLPYASNKYLIRDLCLKTSTLVFCTTSSSYKIHSIEMEPFNVLVIDEAAQLRECESVIPLHLPGLKHAILVGDECQLPATVHSKVSDEAGFGRSLFERLSSLGHSKLMLNVQYRMHPAISYFPNVSFYHGQVQDAENVRGKTYERKYLQGRMYGPYSFIKYPCGKEELDDIGHSRRNMVEVALVNKIVKDLYKFWLSTGKKLSIGVISPYTAQVVTLKDTIGRKYDNLNGFAIKVKSIDGFQGGEEDIIIISTVRFNSSGSIGFMKSLQRTNVALTRARHCLWILGNERTLFDSNSVWKGLVLDAKDRQCLFSADEDSDSIIFKSQRWKVLLSDNFKRSFKNLVTSRMKMAVLNLLIKLAGGWRPKRKGVDLVCETSSQIVKQFKVEGYYIVCTIDIQKEVKYTQVLRAWDLLSLDEVGKLLRRLDGIFAMYTDDFINLCKQKCLDGDLEVPKAWPASYDLVRFKNLGERLADSSNDCVVDGRSYIENSRVNESLLLMKFYCLSSGVVHHLLSDNQGEEIDIPFEVTDEEKEVIQFGRSSFILGRSGTGKTTVLTMKLFQREQQHQLALGGVMKVEANEISEYAGESSFSRRGSTWKSQSEVDIKRTTLRQLFVTVSPKLCYAVKQHVSHLKRGEISAGNSVMDLDDLDGFSHFNDIPNSFVDIRIVSILCMSRAATVESFIRGKGVSFDHFCCLYWPHFNSQLTKNLDPSRVFTEIISHIKGGLQVCENDGAKLTREGYISMSENRTSTLNEKKREVIYDIFLGYEKMKMERGEFDLADLVNDLHLRLKSENLNGEKMDFVYIDEVQDLTMRQISLFKYICQNVDEGFVFSGDTAQTIARGIDFRFEDVRTLFYEEFMMKLKGDGPPARKDKGHLAGVSCLLHNFRTHAGVLRLAQSVIDILCHYFPQSIDALAPETSLIYGEAPVLLKPGSDENAIVTIFGNSGSISGKMVGFGAEQVILVRDESAKKEVSVLVGKQALILTIVECKGLEFQASEIAIACNSFLDVLLYNFFGSSPLSNQWRVVYEFMKQRDMLDSRFHQCFPSFCEARHTILCSELKQLYVAITRTRQRLWICESIEEFSKPMFDYWMKMCLVEVREVDDSLAQAMRLASTPEQWRSRGINLFWEKNYEMAIMCFERAGERNWEKRAKATSLRDAADRMRDSNPNVSCTYLREAAEIFESIGRFESAAECFCDLKEYERAGTIYLKKCGEGEHKKAAVCFTRAERYESAADIYAKHNCFSECLSVCRKGHLYDMGLKYVEYWKQHAQERGIEIDGIEQEFLESCASDFFEHNDRKSMMKFVKAFKSLDHKRRFLKPLDCLDELLLLEEESGNFAEAAELAKLKGDLLREADFLGKAGNFSKASSRILWYVLANSLWVRGCCAAWPLKKFESKDELLKKAILFASNESDAFYESVCTEAKVLSHDPSNLCELRRALSALQKCGSLKGEILCLRKIIDVHTQINATKYSWEDKFPFDLKYPDDTMFCDQLSVGTLCHFWNLWKRNILEVLESLNCLEVQDFGKYKGYGEFCLNYFGVRRQFTDMKVSYLLLNPDAEWVKKVNQSFLRQSKNMVSIDVRHFIIAARNYWQNEVLSVGLKVLETLESLYSFSMRSPSLFSQSMCLYLEKVFPFDYQKPLEENIISLRGTQIGRLMMIWLGSAEPADELCCKIFERTREDSNWKAFINILRSVREPKNETASAGSQEASPVDPCKAGFLLVSRFHEALNETYQIHWQNICDYISPHCFLYLVERFLILAFCSSGFFYTTKSSFLEWLIFQKPGVNVIAGFPTRLPSSEMFYKSITSMVHWLLFHKIETARWIEKSKIKFRNYHKLLVLRLVVILCSLCMNTSSGEPWDALTHALKTHYISSELPREFNAVFIRRGGKHTAFVDRVKIAEALRVLGNPALFVNLKENTPSSLCPNTFYVGIGPNSCREDILEMLFPRKSVTSAVQKSMKNSCCVLPLIADKTSLLPSPDKASKQNQAQKDEEVKSTLQMKWHILEEELVSSRTGETENEGKARILTTTTCFKSSEDLNECIQFMTAVVNYMSGKETQSGEDADMLKEAESMLQELKQLSDKLNTRDGGEVTLMDSERDRAANQGSAEVVDEDAGNVVSESGGVKNQGERNKAKAKNKSKKRKGKSGRR

>IbDExDH141

MMSRCDNRLLWDELDYLIAEALLKESQIMESQLTEEQKLVYEIVVNDVDHQKGGLYFVYGYDGTGKTFVWRALSAKIRHEDSTQHHPRTLCDLLRFTDSNAGNRTFGGKTIVLGGDFRQILPVIPKGRSPAMLLRNIDHLMGLCNGTRLIITRLSDHVVEAKIVSGNNAGGRKLFEVTTMSIKIYFYTPILYDILGVGNPEISCPAKGSGPFVRRGSTVDCAPPKCSAWLLEI

>IbDExDH135

MERLSSAKMKAWPKDDFTDIVISWTLEDIFNQNLFKDQVEKIPETFNSADNYLCSFLFPLLEETRAQLAASMEVMDRAPFAEVIALDAVKKHGEPLYSVKVDVWKNRVRDDREPYRTLPGDIVVISDNRPVSASDLDRAGSIWTLASVVNLVDDEEDGANISTNFKVKMPTDLTANFEKYEGFHIVFLENITTHKRIWNALQMKKNMKIIDSVLYTNGEAEKKCYLCSPHPDNIGCTDRIGTGMFSKLNESQANAVLTCLERVKCDQISHVDLIWGPPGTGKTSESSEKYLLYPLGDVVLFGNKDRLKMGGDIVEIYLDNRVDRLVECLGPLKGWRQCIDSTVHFLEDCVSDYDIFVENELKMRELNVKGDECQLPATVCSQVCSEAGFGGSLFERLSSLGHSKLLLNVQYRMHPAIGHFPNLSFYHMQVLDAPNVRSKAYETVSPGKHVCSRKKLSVGVISPYTAQVVAIKDRIGRKYDNLNGFSVKVKSIDGFQGGEEDVIILSTVRSNRSGSIGFLSSLQRTNVALTRARHCLWILGNERTLVDSNSVWEGLVLDAKDRQCFFCAAEDSDLLKTVIDVKKELDQLDDLLNADSILLKNQIWEVLLSDNFRKSFKNLVSSRLRMAVLNHLCKLASGWRPKRKCVDLVCESSFQVVKQFKVEGYYIICTIDIQKDLNYKQVLRAWDLLPLDEVGKLVRRLDSIFAMYTDDFIDLCKLKCFDGVLEVPKVWPGSYDLVRFKNPSERVADNSNDGVVEGSSYAENSRVSESLLLMKFYALSSGIVNHLLSDNHGEEIDIPFEVTDEEKELIQSGRSSFILGRSGTGKTTVLTMKLIQKEQLHQLALGGVMRVETNEISKYAGESSFSRAEIAMMSQSEAEAKKPTLRQLFVTVSPKLCFAVKKQVSNLKCFVDGGKFSAGNSVMDMDDLDGFSHFKDIPNSFVGIPDSKYPLVITFHKFLMMLDGTLWPSYFDRFHDTSTLSLNRISRSVTLDTFIRGKEMKRGEFDLADLVNDLHRRLKSENLNGDKMDFVYIDEVQDLTMRQISLFKYICKNVDEGFVFSGDTAQTIARGVDFRFEDVRTLFYEEFIMKLKGDGLPARKDKGHLAGVSCLLQNFRTHAGVLRLAQSVIDILYHYFPLSIDALAPETSLIYGEAPVLLKPGSDENAIVTIFGNSGSMSGKMVGFGAEQVILVRDEAAKKEVSDFVGKQALILTIVECKGLEFQASEMARACCFFCLDVMLYNFIGSSPLRNHWRIVYEYMKQRDLLDSEFHQCFPCFSEARHAILCSELKQLYVAITRTKQRLWIYESVEEFSKPMFDYWMKMCLVEVREVDDSLAQAMRMASTPEQWKSRGIKLFWDKQYEMAIMCFERAGESKWEKRAKASRIREAAYRKRDSKPNEFYTSLRQAAEIFESIGRFESAAECYCDLNEYELAGSIYLNKCGEQEHEKAAECYTLARCYETAAKIYARQNCFSECLSVCSKGRHLYDMGLEYVEYWKQHAQERRKEIDGIEQKFLESCASDFFKCNDKKSMMKFVKAFKTLDHMRMFLKPRDCLDELLWLEEESGNYAEAAELAQLKGDLQREVVLLAKAGNFSKASSRILWYVLVNALWVRGCWAAWPLKLFESKHELLKKAISFASNESDAFYESVCTEAKVLSHDPSSLCELRRTLSATHKCVCLRGEILCLRKIIDVHTQIDVTKFSWEEKFPVDLKYPDDTLFCDQLSVGTLCHFWNLWRRNILDVFESLKCLEVQQDFGKYKGYGEFCLNYFGVRRQFTDMKVSYLLANPDAEWVKVNEVLAVGLNVLETIESLYGFSTELLSRFSQSMCLALEENIISLRGTEVSRSLLEEFIVNDLSGKGKLTHGQIGRLMMIWLGSAEPSNELCNKIFERIRDDSNWKDFISILRSVREPLNISTSDDSEDPCNAGCLLVDRFHEALKETYQINWQNIYDYISPHCFLYLVERFLIFAFCSSGSFYTTKSSFLEWLIFQKPGVSVIAGFPTGLQSSEIFYTSITSMVHGLLFHKTETVCWIKKSKIQFRNYHKLLVLRLVVILCTLCMNTSSGEPLDALTHALKTHFIFSELPREFNAVFRRGGKHTALVDRVKIAEALRVLGNPALLVNLKENTPTSVCPNSFYLGIGPNSCREDIMEMLFPRKSVTSAVQKSMKNSCCVLPLIADHDRKTSLLPSPDKASIQNQAQRDEEVKSTLQMKWHILEEELVSLRTGETENDGKERISTTTTCLKSSEDLNECIQFMTAVVSYMSGKETQSGEDAVDMFKEAQSMLQELKELSDKLDTSCVDEEEKGADVFKLRKSLQTRKPRLESFLNNIVVGKDGGEVTFVDSERDGAANLCGAEVVDEDAGNVVGTAFGGGEKNQGDR

>IbDExDH149

MELLRVVIQCLFLLILFSKTSFSLTPHKIPRLNPFYRSILRRQYRSSSSSDRTTSATSDTQFKTYYYNQTLDHFNYGPQSYATFKHRYIINSNFWGGAQSNSPIFAWLGAESSIDSDPLGIGFLTDNAPRFKALLVYIEHRYYGESIPFGTMEEAMDDDTTRGYFNSAQALADYAEVLLYIKKEYSAQDCPVIVFGGSYGGMLASWFRLKYPHVSLGALASSAPVLYFDDITPQNGYYSIVTKDFRRPFRNKSAIKVRVVHTYIVMERKGSTEIKSKEIVFHDEQVRQNQNWMFETLKDEDLLHQQYWKSMETQGYCPKEYISKLTLIMNVNINDLCLSDDEKKNLALIEIERLLQSYNKSLKDYPHMPIPNYDDVVRCENRLLWDELDYDREALLKESQIMESKLTEEQKLVYETVMNDVDHQKGGLYFVYGYGGTGKTFVWRALSAKIRSRGDIVINVASSGIASLLLPSGRTAHSRFAIPITVTEDSTCNITQGSNLAELIIQCKLIIWDEAPMMHKHCFEAVDRKLRDLMRFTDSDAGNRTFGGKTVVLGGDFRQILPVIPKGTRQDIVSASINSSYLWESCQVLRLTKNLRLSNKESTADLKTIQEFASWLAAIGDGTMGGPNDGYSNVQIPNEMLLPPNGNDIATIVESTFPLFKEGGCQLQYISNRAILAPTLDVVNEINEYMTNLHVAESKTYLSCDTVCRSDAGNGILADMHTPEFLNGLKASGIPNHSLTLKVGSPVMLLRNIDHSMGLCNGTRLIITRLADHVVEAKIVSGNHEGQIVLIPRMSMTSTDTRLPFKFQRRQFPLMLSYAMTINKSQGQTLTHVGLLLRKPVFVDGQLYVAASRISNPKGLRLLIANEDPDSSNYTTNVVYHEEVSESCYQTIKESWSIIDKIASKPYGLSILRRKFKLCQDLNSSWELKDYLDEIYCEAAQYNSPPEYPVTMVCGAIDGAPKEAHILDRIHLWCRSPKGINLATMFPERRPERMMKFGDGIGNELVIRIKGNDSMFNPTPFNLQRYSQFCIDNFGIPPRPHWVTTYYGGHDIKFVLRNFGSNIIFSNGLRDPYSIAGVLENISDTILAVYTQNDTQIAYGLIKEQEGHPRVLDDYISFVGLLVVPCYSGFSYDEKEEVRVLLRHDPKFWNLT

>IbDExDH157

MVTTTSSVHSMDISFEDSQPPTQGRLYVHNRTREVMVSTVPGQPVPYIVMSNSDHEFPFNPTLHLSSPTNASSFISGVIAAAEPGAGPEVADRIATFAFGVVSGHGWVSGLSMRVNLHTTDVFFDHEVSGFEEDDEMDDEGFGSYDSSESLGGMSERELCMLRMEQFTGDEGECCICLEGFMEGAVITPLAPCSHRFHHSCILQWLRSNPTCPICRRRPTVPV

>IbDExDH158

MEEYLGKTFEKEFEGLGIVKGTVRSYESESGLFEVVYEDGDSEELDAIEKRRRIGEEAGNSGCNLAAAAADGMGESSGVSSDGHEKGGVTLNLNLNDHHPMLNFDLNGDVDLNDDGGGGVGRVDLDLNLNEGLDLNTGVDVSVEYNTLGRVEENLSRSNVIDLNADPNEDANCAFAKDDSLECVSLVIKEKSHSFDLNLEAGEEEPTNLDVKCDMKDKGITVPQADSSSQKHERPQFLKEVELNLVDTGPAGVGCGNGISSISGKGRRGRKRKSLSDAPDDMTEPMLRRSTRKARRAGLSSHDNDTETVVPDAANDPLSSPAQSVVSDEKLTVSSHEDSEERNFLPPKPELPPSSDKLDLEGISVLDIFSVYALLRSFSSLLFLSPFELEDFVASIKCNTPTVLFDSVHVSLLQMLRKHLESLGSESSESSSNCLRSLNWDLLDSVTWPIFLVEYLLTHNSGIKVAFDICHLKLFESDYYKQPTSVKIEILHCLCDDVIEVEAIRSELSRRTVITKPNIDFDQNMKLDSIKKRRVTTDLATGSCLTEDVDDETDDWNSDECCLCKMDGNLICCDGCPAAFHSKCVGIASSLLPDGDWYCPECIINRKVPWIKVGKSIRGAELLGIDPYGQLFYSCCSYLLVSDSWGDGSSFKYYDRNDLPAVIRALKSSEVVYRTLLVAISKLWDASSLIDGATSDLYSQNKVVCEDFPMMPLQQNNDIVNGEKPMEMVMISTCSGDQGCEKSESVDLSAKMEKQLGSSEESTDLSQAQLSNQNSSRSGMQYDSESKQQYIDAYVNFYSFAHSASSIVEELTHKPSDKSTEDALRSEEEIISAQLKAISKRSADFCWSNIQNLNVDARKEKCGWCFSCKVPECKRNCLFLMNDTGPAPERFSSEALCASKRNTRKGHLVDIMYHIICIEDRLHGLLLGPWLSPMYSQMWRESVLAASDVASLRIPLLNLELNLRQLALSAEWFKNVDSLATIGSACHIVTNRGRVSSRHGMGKRLSRQLFSWKVLPRSLASKAARQGWALKLRYNYYRVLRFECTGGCKKIPGILYPDGAELAKRSKFVAWRAAVETSGSVEQLALQVRDLDAHIRWNDIGNVNILTMIDKESKKPDRFLPEIVLKYGSMLEESLDKRKRYWLEETYVPLHLLKAFEEKRIACKSNKLSPIKPKKSENDKCGHCNRDVLISCHGPLCAQLSRGQKESPISCILHALLLVPLSLFSTKQSILSDQSKSYLVKRLKEEISMSFQADSVGFFTGNGEDREFLEMGREAVSCQYCKGFFHKRHVRKSTGTIPAQCVYTCYKCLDGKHVKSKTKVKLGTKKNKNTSKILMALHSRTKRRRAKILSVQEKNIKKKIGMTSGKFMKSRRGRPKILSVQEKNIKKKIGMTSGKFMKSRKRTSKKPTEAIQKKKRTQFFHIYWLNGLLLSQKPNDERVALFRSKKVFVLSGPLGATVDSPKCSLCGELKSTLALNYIACEVCGDWFHGDAFGLTSERLDILIGFKCHKCRGSNHPVCPCMPIIRGSEAKLVGLKSDAMIESANLRHLEEKFQSHVESNRSCLSGDDDKKQLSNVTNVDNEEDGSLHVIGIAQTTLVMDGSGRTDIQLQDEPILLNENFDNSSQKDQKPPEEWAFPNDKSSIEGDAMEIDDAPIGLWSKTWRQRKPCYHSGMMVNRLALNIVACLGVFGFRSEVENGCSEAPDLAFGSQ

>IbDExDH153

METSIVAATDSNQPSGSDSNSEVKKRSWDQREALPDEPKCVICGRYGEYICDETEDDICSLECKKILLSRIEKSQKPTPCPPLVRFPAIDDCYYVRDDDKKSESKSLASDQAELLRRKLNIVVKGDLASPPIMSFGSCDIPRKLLDNLEAAGYEMPTPVQMQAIPAALMGRSLLVSAETGSGKTGSFLIPIVANTVKLNLACSERKQKPLAMVLAPTRELCIQIEEHAKVLGKGMPFKTALVVGGDAMARQLYRVEHGVSLIVGTPGRLIDLLTKREIELDSISMFVIDEVDCMLQRGFHEQVMQIFRALSQPQILMYSATISREVEKMASSMAKHLTVISVGQLNKPNGAVKQLAIWVESKQKKQKLFDILTTKRHYKPPVIVFVGSRLGADLLSEAITISTGLKAVSIHGEKSMKDRREILRSFLVGDVPVIVATGVLGRGVDLLSVKQVIVFDMPNSIKEYVHHIGRASRMGEEGTSIVFINEENKKLFPELVEILKSSGAAIPPELANSKYSVGSFSIGRHQKRRKYGT

>IbDExDH154

MESTDSKHEIVIVGGGICGLATALALHRKGLKSIVLERSKTLRSEGGGIGILPNGWRALDQLGIGSHLRTVALPLQRNPIGKKPYQTESCQPTPSPNSALTIAKHPLHRSPSQPPPPAAPSVTIDQFEQQFSDAIAVDQFGHQFRLRRRAVRPPVQRHHRLPPPSPPSTISPIHPPTDLKPQSTPPPFLLRRCYPTPFLRRRCLTHDCHVLSDLMLQFLLHMDEPLPLKQLIDELSASKRSEDDEIHKLFTYIMYIRPPKCIGADYSNIVLLFIKQQRETELLVHASGRRNDSVLVILVPTRELALQRSQVCKELGKHLKIQRRKFASANRDFSCSAPNEVRLCELLRLFSQTTRSAKLPASWGYNLGLPRRGREIFLVEDKEQKIENVSGDVRCIARNDIIRSLADALPAGTIRFGCEVSSVDYHSVTKFTRLLLSNGGYIEAEILIGCDGGRSIVAELLGLKPAKEFGIVAIRGLTTYPNAHSLPIEIHRMIKGGVRVGILPITHDLVHWLALRLGTARQTLLHPAVFELLFAGTSWKNKSVGELQMPGPSGSNNPLGGIHHFPEFLQGNDSGK

>IbDExDH148

MVAAAMGRVERRGRKRRRNDVQSVEADQDGKKRMVGTRSKVLVGRYVSKEFEGSGTFLGKIMSYDSGLYRVNYEDGDCEDLDSSELKGVLIEEDELDRDWLERKKKLDKLVSNKEVTTTDCQVENAILPADVAVQTTAPPSSNLGSADPYKVEAGQVDDNAGSMSDLSDEDEVLDLISNVETPVIPPPELPPSSGNVGVPEEFVSYLFSVYSFLRSFSIQLFLSPFALDDFVGSLNCPSPNTLLDSVHVALMRVLRRHIEKLSLDGSGRASKCLRNLDWSLLDSMTWPIYLVDYLMIMGYINGPVWKGFSAHVLEREYYSLSVGQKLMVLQILCDDVLDSEEVRAEMDIREESEFGMDSDGTAVAPMSGPRRVHPRYSKTSAFKEQESMEIIAKDHDMRSSHNASSLSSKVSSTDAGADVDHDGNGDECRLCGMEGTLLCCDGCPSSYHPRCIGVCKMYIPDGNWYCPECRINELRPTPIRGTSLKGAELFGVDSHGQIFMGSCDHLLVLKDSANSESCFRYYNENDISRVLQALNTNVQHHALYSEICKGIREFWGIPFNVLPHTRVTEIGTEITNRIEDSGCSVPSLDNLACCASEISLENTHFHKYPKELVLNEASGRIVHPDNGNFTVRQASEHMNSVPLKQIPGRPTVCAGSVSQQVDFPRQTQKDNTVLIETASCTSRNSSNCIGYDSVHGLGAVPSERSFKNPTEGGLYTGTSFKAQGYANNYLHGDFAASAAANLAILSSEENQCSESLSLEAPKLMSVDIQLQTKAFSSAATRFSGQILKKNLLKFLGKAASNATKGAMKILATVRPAKSGDGNLPGIATYIILMEESLRGLTVGPFLSAAFRKNWGKLAEEATSCYSIKSLLLELEENIRTVALSADWVKLVDGSSESSVSQNASSVTGSTHKRKPGRRGRKPSAVSVVAADEGQGGKRKIPGIYYSEGSETPKRNRRLVWRAAVEMCKTASQLALQVRYLDMHVRWSDLVRPEPSLQEGKGLETEASAFRNAFICDKAMVDNEDGKEKYWFSESRIPLYLIKEYEATLVKNLPSPIDNPMNAFSKWSKKSSKTFRGDIFSYLSWKRDGNDKHSCASCQTDVLLRNGIKCSMCEGLCHKQCTISSMLKINEDVQCIITCKNCHQSSSVTRCGISNESPTSPLLLQGQDFSNVDTVRKGGVLGSSNRLSVSTGGNGRSSGVKQTSSANPKAKTKSWGVIWKKPNATDTGADFRKKNILRKGDPIDGPGPDCHLCRKTYNSNLIYIHCETCQNWYHADALELEESKISNVVGFKCCRCRRIRIPICPYLDPDSKKQLTEKRIRSRPSKADDLGMDSDSGIISEKYQEEEPCTPMVHLKEEISSIMDDFHSLSSMGKITKQDPEAYCENTASLSGRGPKKLPIRRHVKHENYLDSSFANNPSNDYSSTNFGENTGEVITPCVDWGAKLPVRRRPMTSDDPDYSFADPQVQLSTNLGVEWNTSTNGCEDGASLNCDGLSYEDMEFEPQTYFSFNELLESDDCAPFDGGSGNVTEKWENASELPHDEMSTIPIESDAPAVVPCMMCSHTDPSPTLCCQTCGIQIHGHCSPWVEQPSTESVWRCGSCREWR

>IbDExDH152

MSVGTDSDDLQALATAQRRELMAAEAMESDFEFAFHLQLQEALNASLSLQPSTSTDLPAIPPDHKPSSSAAVAAAVDGESLSYTAALSQELVKFQQELDDQKLSVTEFKKIRDDLHRRIHDQKFAEEIMRIPEDEWEDWGGDFERPFGEGSSKSVNSEVFRVYFKGLIEKCGPKHVLGGIGVAICDSSDQLLFELSKPLLGSDLNRHCVEFKALIEGLNAALSLELKRVVFYCDYRPIHRFVTGHWSPKQRKVAALVNQVNALRGKFVVCNPSFVARNEIKFVFKSAREALDSQVKKQAESTASRAGRETCVICLEDVEAAEIFSVDGCMHRYCISCMKQHIEVKLLHGVLPVCPHEDCNSELRIDSCRKILTPKLINILNQRIKEASIPDGEKVYCPYPKCSELMSKSESWEYSRGAVVRSILINGARTCTKCKSQFCINCKVPWHDKMSCNEYKRMHPSPPEDVKLKSLAAKNLWRQCVKCNHMIELAAGCYHMTCRCGYEFCYTCGAEWKDKKQTCTCPLWDEDYIIDSDEDDDFEDDDYYDDMEDFGSESDYDEVDYW

>IbDExDH151

MDKAPMTHKHGFEALDKIMRDLLRFVIPGSADKTFGGKTVVLDNDFRQILPVIPTTTRPIVVRDTINSSYLWANCKIANIGDGVAGVVYNVTSKTSIVHLSASTHFPIKLTPSNFHVWSRQVHSTLIKMNLDGYIDGMTVAPDQFTDAAEKVVNPLYLVWSCSDAIQPVISSAKLAHDAWSRLKASYANASRGRVVSLKAKLAKNPKGGKIVSEFLHEMRAITDELALVQSPVSEEDLVVHVITQLGDEFSSIVSFNTTTLSTPPHQLWLFDSGASHHATSTPSTLQSFVDYGGPDEIHLGNGNRLPITHTGHTILHNSVCNLSLKNVLCVPQIFAREYISCRGRTVHDVYCVPSWSPPQINAASLLVSDRHHRVSHPSHRNSNVDVSKLASHSKTNMPTLSQSLFPSRRPPLPGLSIGALSSSLGNSNSVPPPALSVPAAPLSSHDAYVVLPENPLSQIEGPSNSHSLVPQEVPSLPSTSTNSSPSPSTTGQPTLSSSSSPHRSVPENSLSTRPSRPIRQHRHNPKFYCDAYINFTTLHPISSVLEPSSIAQALKDSSWRQAMFDEYEALMRNGTWELVPWSNHTPISFKWVFRVKRKSDGSVDRFKARLVARVFLQAPGRDYFETFSLVTKPVTICLLSLALSSNILATFVDRVRPCMASSRHLRLVYVDDIVLTGNNSSFLDSFVRQLSARFSLKDFGLLSHFLGVEVASTPMNSSTMLVLDDGSPMADASLYRQLLGLLQYLALTRHDISFAKAVSRSSTEAGYRALATAAADVLWVQTLLFELGVSLARSPILLCDNLGATYVGAPVMLLRNIDHYLGLCNGTYLIVTRLAYHIVEARIVNETHEGTKVLILRTSLSPYDTRFPFKFQVISLTSFQIRSLVELKNGTKAFGNGSGAEFLAVGEASSAHRKRKLLDNARRGSDDSAKAAVVAVQLEHLVPNSGWDEHDERERRHREQQRRAREQWCQAFALAAGNNAASPSAASFDCNTVRSSGEKLYQQLKPLHFFPLWPFSPF

>IbDExDH147

MDVGDLSNGTASEPPKGSTSRLTPLIIDELSGLSLGDDEEAPVEESKPVKDPRRIARKYQINLCEKALEENIIVYLGTGCGKTHIAVLLMHEMGHLIRKPKKNVCVFLAPTVALVEQTGIDLQSSSRSIVAYFAELAQAKVIEESVDFKVATYCGNLKHLKNHHDWEKELEHYEVLVMTPQILLQNLSHCFIRMEKIALLIFDECHYAQTESNHPYAEIMKVCNGLNLGLQILYLNSMHACIFSLISCSVCYTFLMFPKVCLLHIGASIQGLENVLRAKVYSVEDKDELDQFVASPKVNVYYYRSTTDGPVMIFSNKLERLKHQFVTELHKQSGDQTILRNTKKSLQRCHSHLIFCLENLGLWGALQASRILLKGDNYESYELFEPERNGSVNSLCDNYLIPAAAIFASGCNRDGVTNDLELLEEPFFSKKLLLLIQILSNFRVVPNQEKKFHLLAEQLYSKMWVQALKATFLKRKEQHFFRAIHILLLVRPDMKCIIFVNRIVTARSLSCILQNLKLLTSWKCGFLAGVHSGLKSMSRKNTNTILDKFRSGELNLLVATKVGEEGLDIQTCCLVIRFDLPETVASFIQSRGRARMPISEYAFMVNSDNLRELQLINHFKRDEAQMNEEILTRKSSGAFSDFNEKTYKVDATGATISSASSISLLHRYCSKLPHDEFFIPKPQFYYIDDTEGTICRIILPANAAIHQIESSPHSSIEAARKDACLKACKTLHELGALSDYLLIDPDDRDDSMQDFSDSEDSDDEGSRKELHEMLVPDAFKESWSETESPVCLHSYYIKFSPNPVDRDYKKFGLFVKAALPRDAESMRLDIHLARGRSIMTVLFPHGVVKFDANEIKLAEQFQQMFLKAILDRKELIPEFVSLERKGPRDSSSTFFLLLPVNLHKDNKFFVDWKLVRRCLSSPIFKTPEDVKDDGISQFHIQLQLANGPKSRSDVVNSLVYMPCKEKEAFFFISDIVVNKDGYSLFKDSRDHVTHYADQYGIHLLYPEQPLLKAKPLFCPDNLLRKKGNTDLCKSVILVPVNVGSRWYHINGMWSGYNFLIIEMREKEEYFVELPPELCQLKIIGFSKDIGSSLSILPSFMHRLESLLIAIELRKQLAAAFPEGGEVTLDHVRPSSLFMVAHFFLKHHVTSEASVIWSIFEHCLNQGMNLDQVLEALTTEKCLEHFSLERLEVLGDAFLKFAVGRHIFLLNNALDEGQLTKKRSKIVCNSNLHKLAIKSGLQVYIRDHTFEPDQFYALGRPCPVICSKQTEDSIHMLSNSSTMTNGANSEVRCSKSHRWLMKKTIADVVEALIGAFIVDGGFKAATAFLKWIGIQVDFSASQVINVCSASTRFMALADKIDLTGLEKLLDHDFVHKGLLIQAFIHPSYNHHGGGCYQRLEFLGDAVLDYLITSYIYSVYPKLKPGQFTDLRSASVNNKTFADIAVCRSLYRHIICDAPNLRESITSYTNFMGKSASEKGEIEKPYCPKVLGDLVESCMGAILLDTGFDLNYVWEIMLSLVDPTSSFSKLQLNSVRELHEFCQSYGWDLKFHKIKNDGQSGIEAEVIGKDVSTSASATNRPIKTAKEMAAEHTLKLLRAKGYKSKIKPLEEVLKTAVKMEPKLVGYNEVPCLVTAEFGNLTLQQISESSCNINTRPLTEVLSKNFHAKAKPMRNFVFSDETEGHSRQAIGCNDGSADSHVTGASRSATAKSFLHEICTANCWKPPVFQCCDESGPSHLKRFRFKVTVEIEEKARVLVAFGEPQLRKKDAAEHAAEGALWFLKSEGYL

>IbDExDH150

MYGGALYSLSPSPGRKLKSFTWGEIRNSENLTASASLACPLRSRNPAGRRHTPSGTLPPQGSETQQRYFVDNRAVRRTESKSHTYENLRVLALDYKVGGDEGTVTVAAMYLAIQMVHKTRSGKKINVDKANGNGSSSSGMGKMDHLGLRKSAQEIPSRTSKSKLLEKQPPSTPCTQIKSRRPEKPNTLSPLRTPDRDKSHILSCSLVSKLSKEELNLSATKKEKSVKQVTMESEKVSTSGNQNICLKRKRIDGRSFKSMFKMQRRRDAIPVAIDEVSLANESPRLISDSKLVGDHAAQADEVDSLSSSEGLNTGNLPEGCSTATKNLPDLIGSAPTSGGFNSQNGEHSIQRKAADRQIVCGLVYMTTQSTLAEHDKHSFTGSCGVCLKRKRLDHDSPKVDLHMCCNAKDGFCLESLVHSGIPGSSRTILLHPLAFHNATGIPGSYRTILLHPPTFHNVTGIPGSSRTILLHLPTFRNATGICKLPPPLLPELPPPYPATDGHHQPLPESFVGHHCLPQLPLPEKIERRRHGRGRFERVQGERQSFLQPDLLGFQRRAYWNKLRPERWSEKASRAPTRRSSSQRRRVTAPLPPAVATPCLDQCPLGSDTIVGVESTVILGSAERHCAGQSEGANMDSQVDNHTNVCAVCKQGGKSLWVISYVFAVRRCYHLSCLGPNLDDIPSGIWHCLWCVKKKIKYGIHSVSKGIESIWDSREVEEVSGAKGLPDLHFVFGNIRTQRQKQYLVKYQGLAHVHNHWLPEAQLVFEAPSILEDFKHKDVGLRWNSEWTKPQCLLKKRSLFFSKLHGGEQILDAGDKLDCQCEWLVMWQGLDVESATWELENADFLCSSHGLSLIREYEICQGKQDMMFSQLTASDNRLELSKFIEYWLPVQVSNLQLEQYCNTLLANINALCSHSKKSDPVGVLNEVLLAVRKCCIHPYILDQTVVPLNKGLSPSEILDVGIKASGKLHLLDKMLSEMKSQHLRVVVFYQSIGLVATLGNILDDFLRGRFGDNSYERVETSLNTSKRQAALNRFRKEESGLFVLLLEKRICNTSIKLSSVDGVIIYDGDSNPQNDLRLLQKLSFVSQSKPIRVFRLYSCYTVEEQALVITKQNPNADNLQSLNRNVKNTLMWGASHLFSRLDEYHASDSQASALDISCGQLLLNDVVKEFCAIISQNSETDDIHNSVISKVLKSLGPCITDIPLFGEQKFKYTCGEEPQVFWKKLLEGRNPQWRHISGPTPRNRKRVQYFEDSSCSLEVSNDDAGKKRRKTVNSSVDAISTSPVSEGSQPAALNEGPSTIKAANQSLPTSTACQNSAHTGDHASNSSCSLSHEVNVEPVERVALPDEQKSLHIYLKTEMAKVFEVLKLSDGIKHMVGIFLDFIMENHRVSREPATLLQALEISLCTPNSLVSFPPFHDAPMVDSSTFLHEEDIGLIFFYCRVAASLLKQKLNENEMLMLTKEHLGFECTEDEATNVYRKLRSLKKAFLQHLEKNEITSRHSEPALVFVTGGLSKGMENVTKENVVAQQTTTLKDKVVESETEKMIKKVQSKCDMRMSRLKEKQQEEIENFKKDCEEKRVAIEKHYRVQFAIVRAVYCETPLGKSNLKNLNNRLSREIEELNCHKDFKMKEFEQKHNKEINEEIQNTAFWVSKAELCSTEDAVTNLHLYASDSRDVDEYLQESAGNNISDSRDGTGDQDIADTDNNSISNSREDNGNLQDIEDDNREISDSRDDVRNLQDIADVNDNPTNVASRDDIGNLEDNVDINNHNNNNSNPANVASADDIGHILDNADFNDPPGVASVSECHLEANEPINILNESESSMAPSSVPATTTDVSSATVSEKPMATISPAEALVSSLNQPNEVGNIDDHCEDTVPVVPLTSEKHTIDESSLGEHANGFSIEVHECARTEDVIPNHPSEVHGIAHNGVLRHEHPAGASITTMVKNCTPANNTDCLNNDGNENAIAHKSIDGNSTSQEQLLVALPSLQAGACSDDNGLLLQTQVRSGTSRSVLPHLASNELQFNQGQLSEFEAGSPRVDDARQVTESILIENSEPQQLPSGGNQPPPGEAAYESPNHESNDLHCSETSPQLAEVETAAEPLCNADSQGRVSSELHASVHLPNMTLDCSPTDSSVSRIDCQPSGELHCSSENTQTLPHEVRITSELPNQAILQPLNLTSIQGPNNMRMHHAHFMPSLNSPIRMLVNPLSKELERIHREIEQAVKSHEDWKLQQRSDCEKEIKEMIERIHNKYETKIMEGENAFFLRKNELEGNQNKVLMNKILAEAFRSKCLDPRPTVLPQMQQAVPSSYMQQQHYLQHASMQQPTLRSPVGLSSAGQQTTNLACMSMRPPQSIAGFSSAGQPMVVQQTSVQSLQGANHSTELSREYRPPPMSRSIPTAAVSSSSPSAASQPLSSLQSIPRSAAPSAVPARPLVINDISPSAGNLRLAGDIRAPAPHLHQFRPTSVSAASPSPLPTAMPGCNVAANVPSSSPSFTHVAPQHLTPSPSLPRVAPQVQIRSHSVPRGVTSQHQTPSSSRPWVSPHRPWVAAPHQTSSSSLPLVAPQQPQIVSSNLSHQKVPVPAPPQLQPPTVNKNSEPNNRVLTTGNSPLSALLLRDLDSQPWPRPSNYLPPLPEICATFDSIDLSDFGTTGNAAGTSAAEPNAVADVVCLSDDD

>IbDExDH155

MEEDEEDRVLFSSLGVTSANPEDIERNIFGKTENGFDASNEAARSMEVEATEHNATSSTKKTDMCNKLRAVEVEIQAVKSGFERLENFRRNDEQVSDREDGHEKRDIETEQSTTQAPLNDLDLQRALAADRLKSLLKTKAQLKKDISDLSNDSTQDTLSVLRDIVKEQPKPKQRLKEAKSPSKNKKKRIKSASLDDDDDFDAVLNAASSGFVETLKGFERQIQEPGQSSRHSLQDNSDLVSTSLANAIQSISEAAQSRPTTKLLDSSLLPKLEPPTYPFQTPRKHVKISQSAENGRNDKKRKRRPIPGKKWRAKVSRENKDEGPVIKQFLFLVKDVEISSYEDEKDDEDMDDERQPYVMLEGGLKIPETIFDKLFDYQKVGVQWLWELHCQKAGGIIGDEMGLGKTVQVLAFLGSLHFSNAYKPSIIVCPLTLLQQWKREAKKWYPSFRVEILHDSVSVNDLPDKKKGSKSHESDSESEESLDVDVEKNLSFRNTKKWDTLINHVLGSDSGLLITTYEQLRLLGEKLLDIEWGYAVLDEVHRIIMTGSPIQNKLSELWSLFDFVFPGKLGVLPVFEAEFAVPISIGGYANATPLQVSTAYRCAVVLRDLIMPYLLRRMKADVNAQLPKKTEHVLFCSLTSVQRSIYQSFLASTDVEQIFDGSRNALYGIDVMRKICNHPDLLEREHSCRDPDYGNPERSGKMKVVAEVLKLWKDQGHRVLFFSQTQQMLDILENFLVTGGYTYRRMDGATPVKQRMALIDEFNNTDEVFIFILTTRVGGLGTNLTGANRVIIFDPDWNPSNDMQARERAWRIGQKKDVTVYRLITRGTIEEKVYHRQIYKHSLTNKILKNPQQRRLFKARDLKDLFVLNDDGENGSTESSSIFSQLSEDVNITGPQHPDQEKDKSIKTKPADHGTAMERDNNSGTNGEEKADNNNGDADDDTNNKVSAMDHDAIVNAHDEEKLKLEEQASRVAQRAAEALRQSRMLRSQESVAIPTWTGRSGTAGAPSSVKKEIWRNGIAAGASAGKALSSAELLAKIRGTQERAISDGLEHQLNSGSSSNSRTRPAENGASRPSHGSAGMQPEVLIRQICTFIQRKGGSTSSASIVEHFRSRVSSKDLALFKNLLKEIATLKKTPNGSFWILKPEYHEE

>IbDExDH156

MSTSPTSSHSSSLPEPFSSGSSSSSSSSTFPPLPITAMREKNRRKNPGEPRHSHRRRYWLRVITNEVILSLTGKSSQVPQFLLDENLGPILCTQPRRFAVVAVAKMVARARNCEVGGEVGYHIGHSKVFSASSMIVFKTAGVLLDEMREKGLKALKYKVIILDEVHERSVESDLVLVCIKQFLIKNNDLRVVLMSATADIARYKEYFGDLGRGERVEVLAIPSSDQHVIYQRKVLYLEQVAKLLKRESEHLSSDYCSGSSPSTAEAEIKPEVHRLIHDLIIHIHKNERDIEKGILVFLPTYYSLEQQWFLLKPFSNCFKVHILHRSVDIEQALMSMKILQSHRKGNLSYLGYQFMLSCSIMNHIKFCTFYDIEVILATNIAESSVTIPKVGYVIDSCRSLQVFWDHNRKTDCAELVWVSKSQANQRQGRTGRTCDGFVYRLVTGSFYSQLEDYEPPSILKLSLRQQILLLSCAESKAINDPKVLLRKALDPPDPDVVDDALNLLVHIHALQKPSPRSRPEPTFYGRLLSSFTLSFDSSVLILKFGAIGMLREGIILGILLDMQPLPILRPFGQDTLFMEFIDNYFTGNSKSTGLTGRKEVICMGNLCAFQFWQCVFKDKYRLERLLLLLKHDITDETQTLLPKIEEEWCSVHNLVLSALRQVAEIYDEILGSLHRYRPEFLVKSDGLPSYYYANNFQHTCDLMSDQYEDAAGALVINKLLEQDPGIRKCTAMRMQSSENISGNHHAEDYYDGHITGEASLCKFFIKGLCNRGSQCLYSHSLQAKKPVCKFFFSLQGHSSQRIKILILHFICDFFPEPSDGCVLVLDDTDLYFSSNIAYHFNPSSIIATTSLQDESTLDDRPAGVRVLWGLPHPQQTIMPSCRESAIPWNDVKCLLWFPKFDAEHLEGQKSVVQAFFEHLATRMLENVLYGVEVIITMNNIHFSYFQSVYDGWSFRLKSWLGVFLFLRSHSLLMKEVWGDFMMLLPPEGKCSCLRPSPMFSRCILPTPTIGSIASLWLSRFASGVLIASLVPSSTSAILAYRPTGTPETPLRRSSAQRLLSPLTVCRMPIWAK

>IbDExDH162

MEISVSQDAISTILANPSPESGSDLPEIVVQVVDLKPTGNRYMFTASDGKMKLKAILQSSMSSEVISGAIQNLGLVRILDYTLNDIPMKNEKYLIVTKCEAVSPPLEAEYKAEAKSEGAGIILKPKQEIDVKSVKESETGGITLKPKEETVAKSAAQIVHEQNGNMAPTARMAMTRRIHPLVSLNPYQGNWTIKVCVTSKGNMRSYKNARGEGCVFNVELTDQDGTQIQATMFNEAAKKFYDKFELGKMYYISKGTLRVANKQFKTVQNDYEMTLNENSQVEEASNEAAFIPETKFNFVPIDELGPYVNGRELVDIIGVVQSVSPTMSIRRKSNNEMIPKRDITIADETKKTVVVSLWNDLATSVGQELLDMADKSPVVAIKSVKVGDFQGVSLSALSKSTVLVNPDVPKAKDLRAWFDAEGKETPLASVGSGLSPSSKNGSWSIQTERVSLLHITSNPSLGEGKPVFFRIKAQISFIKPDQAMWYRGCKTCNKKVTEAIGSGYWCEGCQKNDEECRLRYIMVMRVSDASGEAWLSVFNEQAEKIIGCSADELDRVKSQDGESVFQEKLKEATWTPYLFQVSVAVQEYNNEKRQRITVRGVAPVDYVAESKYLVEEMAKMNIISSA

>IbDExDH159

MRKLTKSAVIGTKRKHPSNEKGSIRNGTYTLPLIDLTNVQSTSRIASQRCQYTPTNFMSGGQLTSTDVTKVQSTGGISSKGFQDTPSNLRSVGHLTSSVVTKVQSMPPKSSQVLADTPTNFSSATHFTSSVSKKVQSTGGISSKVFQDTPSNFRSVGHLMSSVVTKVQSMPPISSQVLADTPTNLRAAINLTSSVNTKVQASRGRSSQSFQVNTTNCHAIGQLLSQNLTTETRDATNRMRSNVTIHPARNLEEEFSATLQDTSKVMQENGVVVGSVQHEENNVPKCIPTINGVESTCPLPSTSLDVDNVQSAPPINTNVQTSSRVDINNTIVIPPAPDVIYNDIADPNNICDHCHAIFWYGERINKEVRHGIPKYSTCCIHGKIKLPRLGVPPARLYNLFFGQGDKRIEFLKNIRMYNNMFSFTSLGAKVDTSINIGNAPPIFRINGQNFHLMGGLLPQDGSSPKFAQLYIHDTENEVENRINAFRGDQPSTETHVQIVQDIKQDLDDHNVLVKCFRWAQNHIKSNPQSEFKMRLIGKRNGDARTYNLPTVSEVAALIVGDLDPALGHRDIIVETKAGYLKRINELNPAYLPLQYPLLFPFGEDGYREDIQFNLDRLRTTGAEIRFRNENSLRIGYMSVSVNYLRYYMQRDFSSSSWSTHTQWWNQAGYYISEIIKERYAVRHTRVYDTELSGCHMAICRAKGYPNLFITFTCNPKWPEIQRYMLRLNLKPEDRPDILCRVFKIKLDELVKEIRAGKLFGKVVVVVYTIEFQKRGLPHAHILIFLERRPQGFTADMIDQFISAEIPDPIMDNAYFNAVEEFMIHGPCGRERPKSHCMANNKCSKHFPKKYADVSTLDDDGYPIYRRRSNGQVVDKNGIKLDSRYVVPHNRYLLLKYKAHINVEWCNQSRSIKYLFKYVNKGNDRIRTAMAAWRLLSFEVRSHPPVERLSFHLPDCQSVVFQDDDRIENVLSRPTVYQSMFTAWFDANKKFQSARELAYIDMPTKFVWKKDIREWHPRKRGFSIGRIFYVPPGSGEIYYLRCLLNVVRGPTNFDDIKSYQGVVYPSFMDACYARGLLDDDREYIDVINEASLWSTATAMRKLFVVMLTSNLVARPENVWSQVWHHLAEDLLFNRRRLFSNDNLSLSDDEKKNLALIEIERLLQKYNKSLKDYPHMPIPIIEESWNHNNRLLCDELDTIDALLIGKLETQLTDEQSLVYNTILNDVQEHKGGFFFVYGYGDTGRHLFRKHYPQNKVEGKWSLTLHLAVLLRCYCQAVGQLTQGLLYLLPLQKTLHATYLQQILPVVPKGSRQDIVSATINSSYLWASCQVLRLTKNLRLNTDQQGANIEDIKEFSQWLAQIGDGILGGPNDGYAKPTLDARILNEYMTDLHVAESRTYLSCDTVCKADSNHGILSDMHTPEFLNGLKASGIPNHALTLKVGSAVMLLRNIDHAMGLCNGTRLIITRLSEHVVEAKIVAGHNAGQIVLIPRMSMTPTNTRLPFNFREKTISVSVGICHDH

>IbDExDH161

MTRGYIIKFPSLDNAVQYSDIGDLSNICDHCNAIFWGNNDDGKLQEDIVEDIKQDLDEHNVLVKTFRIAKSKMDNNPSVEYKIRLIGKRNGDARTYNLPTVSEVAALIVGDLSPTSGNRDILVETNSGSLKRINELNRAMHDGFNEVSTIVCQETFPTIFVDAYTMVESSRLLFIRNNQKALRCEAYKGLSNALTRGEVDLTSKVSESSYHPTSWVFKMKLDCLVKEIRAGHLFGVVTAVVYTIEFQKRGLPHAHILIFLERVQSLSTAACMDNFISAEISDKEEDYEYYKAVEEFMIPGVWTTQASPCMVNNKCSKHFPKKFVEVSSWDDEGYPIYKRSDNGRFVLKNEVQLDSRYVVPHNRYLLLKYKAHINVEWCNESRSIKYLFKYVNKGNDRVTAEFYKSTNEDGQDEDDDKIEDVLNRPMVNQSMFTAWFDANKTFDAAKELAYIDMPTKFVWKKDIGQWHPRKRGFAIGRIFYVPPGSGEIYYLRCLLNVVRGPTNFEDIKTFEGVTYPTFRDACYARGLLDDDKEYIDAINEASEWSTTKAMRRLFVILLTSNLVSRPENVWNAIWHHLAEDMQYNRRKALQDKDICLADEEKKNLALVEIERLLQLYNKSLKDYPKMPIPNFDDSWLVHNRLVFEELAYDRAALRKTFVWKVISCKIRSMGDIVINVASSGIASLLLPGGRTAHSRTMRDLMRSKDPHSEGKTFGGKTVVFGGNFRQIVPVIPKGYRQADIGDGKVGGPNDGYADVQIPSEMLLPSSGDNISTIVDTIFPLFKERGCDEEYMKSRAILAPTLDVVTPSMNI

>IbDExDH160

MGMYCLANQLKAWNRRSAIKIRVMRTYCVMERRGSTQVKSREVIFHDEEGTVMHAHIPNDILPKFLNSFVEGSVYCVKNLFVVANWHTYKTSMHEYMLQFNGETIMKEYRSANFPRHMYRIRSFQSLRNNPSINDKELFDLIGRVVQIHAPQQKTINGNDTRLIDFVIEDSHGNRLTCTFWDDHVSKIEPFYESPGNEPLYVLIQFCRLKFGVRDGDVNICSSYDVTQIHFNIDCPEMQQFKESMTELSQLTPMRSIASMSSMSFTNTHDDSSTQSLELITINELYDTEDFGDFWIAARVNGVERPSDWFYISCPKKGCNKALKLSEGVNKCFKCNEIPDGSVRKYKLRVRVVDMKGTASFLIWDRECVDLLGIPAEDLYERNLNNLGNIKEILELKGRTMLFKISAKKEHYVRRNIPFPVVKIKTDQLLLQQLCPDLLALDENDFNSDGEISEGDDKFLEGFESDEGESSIALLPPTSSTDCTTDGPVKRCLLDSFSSTKGGKKVKQSHVKLEKID

>IbDExDH165

METTTAAGGSGKGGGKGGKGRKKSNKSNKKANNGGWPKGVDEATRIRISEILQRFRSSNEEAEREFVLNRNAVAATNLYALNADHGKQRQVFVFNKGKKIDSQRGKETLTCLKFSEGAQAILKDLFSRYPPGVEETTSYMSEKHNKKANTLYGLRDDIFRKPAMDKSDIAKEVELLASRVENNRDLRQIFERRSKLPIASFKDTITSSIESNQVVLISGETGCGKTTQVPQYILDHMWAKGEACKIVCTQPRRISATSVSDRISTERGEHLGDTVGYKIRMESSGGRHSSIVFCTNGVLLRILVNTSKLNKKALQGEKDDVSEITHIIVCQPEVMGVDNGKDEIHERDRFSDFMLAIIRDMLPLYPHLRLILMSATMDEERFSKYFGGCPIIRVPGFTYPVKTFYLEDVLGFVKSVENNHPDNTTASLNSEASVLTEEYRVALDEAINLAVSDDEIDTLLDIISSEGVSEAINYQHSLTGVTPLMVFAGKGSINDICTLLSLGADCHLKAKNGTTALDWALQENQTEAAEIIKKHIEKSFSNSEEQQLLLDKYLSTVHAELIDDVLIEQLVRKICMNSKDGAILVFLPGWDVINRTRDRLHKSAYFKDTSKFSIIPLHSMVPSVEQKKAFKRPPRDCRKIILSTNIAETAVTIDDVVFVIDSGRVKEKSYDPYNNVSTLQSSWISKASAKQREGRARRCQPGICYHLYSKLRAVSLPEFQVPEIKRMPIEELCLQIKLLDPDCNVEEFLQKTLDPPVYETIRNAIIVLQDIGAITIEEKLTQLGQRIGSLPVHPLTSKMLLIAILLNCLDPALTLACASEYKDPFTLPMLPNERKKADAAKAELASLYGGRSDQLAVVAAFECWRGAKQKREESRFCSQYYVSSGTMKMISRMRKQLQSELVRNGFIRGNMSEYNLNAQDPGILHAVLVAGLYPMVGRMLPSPKSGRRNVIETAGGDKVRLHPCSTNFKLSVKKLEQPIILYDEITRGDSGLHIRRCSIIGPLLLLLLATEIVVAPANHKNDVGDDSESDYESGCEDDSEEEKRKSDLSNGENVMSSPDNRVMVVVDRWLPFFLTALDVAQIYCLRERLSAAILYRVTYPSRELPETLCASMYAFACILSYDWMNGISLPCESIDSLTTMVGSAEISESPSGKKNVEADKNYLKSLLCHDSLSGNHNMPTSRGASSSQSSAPQHLLSGPGSARPHKMPTSLDASSSQSSAHRYFLRPRISKAGHSMRQHGNGSSQLLQVVCDAQDRSRVGFLSPKTRGTIVV

>IbDExDH164

MQRPRRLEAKITVTNFRKSIRSDSLQFGIHERKKQCHVAGLVNSESDDSKETTPSCFPGSGNSAFVVQKDSYCKIEEISGVKKLGEKRLMPSMDIINGPAPKYRKVSGNEKDMLASDSKARFSEINRKGPCGKSGSKKLKKYCLEDDDLLISAFIKKPTSKSANNRPSGKPKPLRKQKSKKDGCRLLLQSLNRGGKNFVEGEWPSVALRNVLSWMIHFGVVCIGEVIQYRNLKDDSVVKAGVITRNGILCHCCGEELSITKFKRHAGFKLKNSCSNLFLESGKPLMLCQLEAWSSEYKAKKAGLQTVQFDETDQNDDSCGHCGGGGELICCDNCPSTFHQACLYAQEIPEGSWYCPRCTCQICKDVVKASESSSSSSAMKCMQCEHQYHKACLKHKGFKRGVASEIWLCGERCEKVYSGLTSLIGEVHLLPDGFSWALLRCNPYDQKIHPGQRYVALNAECNSKLAVALTIMEECFLPMVDPKTGIDMMAQVIYNWGSQFPRLNYYGFYTVVLEQDDVLVSVASIRIHRATVAEMPLIATCSKYRRQGLCRRLMDCIEKMLKSFKVETLVISAIPSLVKMWTQGFGFSPLEDEEKRSMSNDNLMVFPGTVWLKKPLYEALEADQPTGSKEVDVAIVGDHCKLNLSPHCSSAGTETRDPDREEMQLGNEGGICLHSRCSICSTEVSVGNQASLLEGH

>IbDExDH166

MPKKDAARRELLDRWRGIEEEDDDADGDFGAHKRRRLHQLKEEWFSDAFKFLIHLPKGDHIWCCSWDLMGPLLETFYNYYKDEHRDSPLKLLWNRVSEELRHCTQCICQYHQAQEMYNTEYESSSIGPLLDVLRTLDEERISQHLKEINARIAQGDSVLGNDNGEIVSVMFEVLMFPILLDDDSLANEFQIFIEAVDDSHELTLGGHQQYPGVYALLFLKNRRARSIGFRLAGQMNRLRQSIDLDPLQPLLKRYISFLETEISSPPQTSRPRVHLERLTVWLGIKALLGFLEPPAFEEGILDRYPIFLSLVLNHISDDSPEFSYAVNCLRLLFEMLGYKLWLRTTLSPSVMRNTLLGQSFHTRNEKSHKEIFDLFLPFLQSLEALQDGEHEKQRRNFLYFLLHQVTASSNFSILMRKKACQIAFLIVHRGYKMNPPNPPYECAHMWGPSLVSSLKDLSLHSSLRQPAFDLIQTIIVSDASAMVASILSSLLHASNERGMPTNFDEDDDEGNLLDHDIEEKDATCWSEFCVQSKMTSSLYGSWMCIPMLWFEVLVEIDPLVLPVSIAKAVFWALSRLAMVEPENNSERSLSLENWLKTCTSEISHAFGWKVPSGSNDGGDGMVSKNSVNVSTVCKPLVKTFKRFSVNYISRMERGELRKQWTWEPMMGNSLILLLVDPNDNARHVARCILEQVSDTRGLTCGLQFLCSSPSSLSAIFVGLRHALKLVQLDSVLSDFQSLHHLFFVFSKLLREGNSSAKPMVGSSSEMSNISNFSSQGGFLKQPVFDEQPGKVIGQSSVSFALSEKFSCLLSEIAWLAIGKCLSEGKANVDQKASQSMLSISSVARTANYLISLLSILATCIVCFIIPADPLCLDDLHPRILEILPVILEKLHGNLAMIFNSAIGLKWLCDLIDWGKSSLPVVVRYWKQTFISLLGLLKGFCSGISALAIVDIEKLISCDKTPIEELTEQVARLSVSLMNKGSFDVKKTNVSREHSSFKKLLPSGDCSAANAQASSVDVKKLHVNTDVLAGKEGGDLIVLSDDENESETPMSYEHSSFKKLLPSGDSSASDAQATSVAVKKLLVSDSDVLVGKEGAKSIVLSDDNNEPKTYISKDVNSHFGSSQTLFDDKVVSANADEQVVYPGPVKGTNSRIDKAMNPVVASKPGLESDMVEGRTAASIKSKVIHEKRKDVDTKLLREHTKENDTLMTQPSSTWKDSSDESMIFKTKDQKDNKEAIETGVTVLQELVQDSEIDLEFGFSKLGRRRQTLTIKPSISGPKRQVIQLDLPVKNRSSLFRVDGRVKRFKSARLDDWFRAILELDFFATVGLSVTSEEDNQKFNKLKQVPVCFESAEEYIEIFRPLLVEEFKAQLLSSFQEANSVEEMSCGSLSIMSVERIDDFHIIRCVRDDFDNTGSKSCLENDLILLTRQPLQNSAPDVHIVGKVERCEKDNKKRSSIIVVRLYLQSKASHLNKARKLLVARSKWCISRLMSITPQLREFQALSSIKEIPLLPVILNPTNHGHSAICSNNLSKLSRPLHQVFKMEYNESQQEAISSAIGPFDLKKDFELSLIQGPPGTGKTKTILAIVSGLLSFCKMKDTRTLTAAPKPTSLSSSTSRPHISNAAAIARAWQDAAFARQLKEDVDKNKDNMGSCSRGRILICAQSNAAVDELVSRITREGLYNRDGTIYKPYLVRVGNAKTVHSNSLPFFIDTLVDNRMAEEKMNVNDAKNDMSKDRVTVLRSNLEKLADTIRSYEAKRANLREGNSDSKSLFEGEACNADGMKELSGAELEARLRVLYGKKKEMYTDLAAIQARERKANEETKALRHKLRKAILKEAEIVVATLSGCGGDLYGVCSESVSGQRFSSSSESVLFDAVVVDEAAQALEPAALIPLQLLKSNGTRCVMVGDPKQLPATVLSNIASKYFFQCSMFERLQRAGHPVVMLTEQYRMHPEISRFPSLHFYNGKLLNGNLMSTKSAPFHETNGLGPYVFFDVVDGKELHGKNSGTQSLYNECEVDAAVELLKSFKRRYASEFVGGRIGIITPYKCQLSLLRSRFSSAFGSSVTAEMEFNTVDGFQGREVDILIFSTVRAAGACSTDQGYSSKIGFVADVRRMNVALTRAKLSLWIFGNARTLQTNRSWSALVKDAKERNVIISARKPYSSIFKSTSKENRTSENPETHLKIQTHTEMVDDMNDGADQRKKTLNSKFDRKRRHTDIGPPTNVAAYNSKHDAKIKKRRATDNCDSFKKDLVSATVENSDVRNCKPANSTVGENHRETSETLEKKPHKKQVNNDKADKQGARYSDVRNCKPEQSAVGGNQVQTSETRGKKTCDKQIKEKADKQRDKSTDKLGSSLEDMEKVRDKGLKHSTSIASKRYKEPSDNASPKGTDGKDGNGVRTQIEKATVSERKQKRDAVEALLSSSLISSNKPKSSIKSLPAKRNSAANTEGPGIRPSKTRKGEAFSRDQGSYYSGAAPRLAVVHALLPLGKHGVDGENSSILVGEFHTCYQRLKAKKRFRTWS

>IbDExDH163

MLKRLRRSILKHEWAILIIDESHHLRCSKKKSEPEEIKAVLDVAVNIKHLILLSGTPSLSRPGLLGKTKYEFAKTYCSIKLVKGCQGKAYHDFSKGIRLEELHMLLKQTVMIRRLKEHVLVQLPPKRRQIIRLVLKKSDIANAMASTRVSSGNAPSLDASGTDAESTVVDIPDKTAGNSGNQECTSKLSEQELGIAKLSGFCEWLSIHPVIAEIGDEEAIGANCSSQKMIIFAHHHKVLDGVQEFISEKGINYVRIDGNTPAFDRQSAILSFQNSKEVLVKIAIVGILAGGSGLNLSAAQNVVFLELPKEPAHMQQDTTDEIRWQSLNRSLHRVSSTMNGKYDALQEIEVDDVSYLGTNGKIKEKRKNFIPNEEKNEVCLLQPTKIQNPCYNESDKMVTESNQEQNESAVYISSSRNQMTLSWMHYFCPKMGKQAVTKSKIDTLGCTSDESDSEDSDHNQEEKLNVALMESKVHDCGSVKRIESNNGSFIHLKSLRFEVSPYTGRIHLYSCIPGKDTRPRPLFENFRQEELDLLHASVDDKEKAYKCIKDDPAYSHVLQSFVNEWNSLRPIKQKKLMGKPLQLPLSTELCYLNENINYDNEGLLKGRSKKRTTPLDEISHPLPSNATWKSVRLVGSNKKERVYTQGWSDKDEPLCKLCQSVCKKSNAKEPEYFEDLFCSLDCHEEYRLRTSRRFIRERLSEIEHGICSSCKLNCHKLVKHIRPLLHDKREAHIKKVAPQIAKRKKLLQRLVAEPNEGNAWHADHIIPVYKGGGECLLENMRTLCVACHADVTAAQHNERRKERLKAKKHLRNIMRDLKTVVMPDQVENKTEDSRLSIDREDVEDDHLLINIPGSAYSGSTSSTTGNQGKASDSSTGKENRSKAYRLPRSRSGKYTSSSPTMRKSASTNGSTSATAQLKVYERMEAKEVCLDVLKDRIKNLGYSVAGALIYYYCDGILKLLQSDNDVIAILNEDNPGNILELWVDTMDELDLVDAEIEVPEGDMSDGEKSIEVECLGVNEQGNVGVEDDLYSDEDSVLESKNEDEGKWPIFRASTDMKDPHFSLGMTFASKQQFREALQNYAFNNGKEIKTLKNEKDRVIVKCTQEGCPWRIGLRKVIGSLSWRILNMIDEHEECSWVWENSMVKSTIAKKIRKCDEEDNFKKLWSYHAEITRTNPKATCIIKLSELGDQAGNERFLRMYVCWEASKLGFKHCRGLIGVDGCHLKAATGGMLLTADDLDITENDEDAICFISDKQEGLLPAFKGVFPNVEHRFCVRHLHANMKVAGFQGKALKDCLWACARATTVNSFHTTLSKLRGLDEDAYQWLGDKSPSEWSRAYFSTKCHCDMLVNNICESWNASLLEARDNPIIECLEVIRKMLMSKLFEQRQKAADWKALICPTIVRKLKQIEKEAAGYLAQQCDFFKFEVGQLYADQQEVDLESKKCSCRKWELTGIPCKHAICAIWKKFGRGATPPLPPLYTAKVGRPRKLRKKSAGELTKDGISVSRQHIQLHCSLGREAGHNLRKCPSNPNKKLFIPPNKSPTTFLLHLDSSTKVDLDKEDIEDLQGGSGQQHQQEDLQGGSGHQHQQEDLQHVSGQQQHHEDDQPSLAGEMQQHDDFNGSTQQQQHDDHNQAKSKGKAKLNIRPSNRKKPYYTRSKTTLKSKFFGNKADPIDIE

>IbDExDH169

MCVQSRDNNTFDMGADDSHMVCDDILDKQNSTCFFFWDDDSIEICGTLINDCEDSLLFDDEYVCFNFWEDDESFNLYDNGATSEGDDVSFYSCEDEGDFSSIIEWEDEMRECEEEMVENEGIVSFDIIESVERGDVAKHEIENGIINDVLQQHGGLYFVYNYCGTGKTFVWKTLSAKILPVIPKGSRQDIVQATINSPYLWETSWLADIGDGKLRGPNDGYAKIQIPSEVLLSSTGNNIATIVDSIFPMFKERGCDEEYMKSRAILAPTLDVVNAVNEYMSDLILLRVGSPVMLLRNIDHSLGLCNGTRLVITRPPDFKWLDLQPVWINMIVMESLGLSRASFQVARSSKEELLCLLTKWPPDFKWLDLQLSFDAAAAITEDSSANTTAIHCTGGIERRGGRIGFVGAEGFLKNGGGIRAKTVEFSTLVSRAAEESHVSLKELVEKSRSADLPIPTRSVGFSSTSSRPSSACFASMSSPSGVNRLVSIEARILQIFCAFVD

>IbDExDH171

MESGRSEIAESSRVEKSITSRESYASNENCPPCTYCNARRFQYEPPGFCCSSGQVILVSNEMPSLLKQLFSEKDEVSHQFQACVRTYNNTFAFTSLGIPNYNKKLTRRNKGIYTFKVQGQMYHFINDLVPGDQPPKNLQLYFFDIDHEVENRIKGADRMEASVVENLIGVLGRNPYSQFFINLKDTSSLDDCNIVIHSNASLDQRVYNMPTSSQVAAIWVDEQDGSGQNNRDIRVFRRTGQSHSVKYYYGCYDPLQYPLLFPHGESGWHEGISKVPKSLTLRFCVGLECQKIFMLKRKVNDFPSVGLEGGTWRCVKHSGVY

>IbDExDH176

MSNRPNYQGGRRGGAGRGRSGGGGGGRGAGAGAAVAEAEVGSSAGGTPPGELSVFMEVMNENEWWGKIEQFKRGGEQEMIIKRNFSRDDQGKLSDMAYQLGLHFHAYNKGKALVVSKVQLPNYRADLDERHGSSQEIRMSTEIERKVGNLLSSSQASVSDGNSSSRHSYTANVSSNAVHAATAAAALESDAAKERLNTELKQKQEKLRATDNVKAMMSFREKLPAFKVKSEFLKAVASNQERLAVGKTTQLPQFILEEEIASSRGANCNIICTQPRRISAISVAARISSERGENLGETVGYQIRLESKRSAETRLLFCTTGVLLRQLVRDPCITGITHLLVDEIHERGMDEDFLLIILRDLLPRRPDLRLILMSATINAELFSKYFGNAPTIHIPDVDIDSHYKGYSMTTRQSLETWSGSQLDLGLVEATIQHICCHEGDGAILVFLTGWDDISKLLDKIKVNNFLGDPRKFLVLPLHGSMPTVNQREIFDRPPPNVRKIVLATNIAESSITIDDVVYVIDCGKAKETSYDALNKLACLLPSWISKASAHQRRGRAGRVQPGVCYRLYPKLLHDAMLEYQLPEILRTPLQELCLHIKSLQLGAISSFLNKALQPPDPLSVQNAIELLQTIGALDDAEELTPLGRHLCTLPLDPNIGKMLLMGSFFRCLNPALTIAAALAHRDPFVLPINRKEEADAAKRSFAGDSCSDHIALLKAYEGWKDAKRQGQERAFCWENFLSPITMQMMADMRMQFLDLLSDIGFIDKSKGPQAYNEYSNDLEMVCAILCAGLYPNVVQCKRRGKRTAFYSKDVGKVDIHPASVNASVHLFPLPFLVYSEKVKTSSIFIRDSTNISDYTLLMFGGNLIPSKSGDGIEMLGKLRGELDKLLKRKIEEPGLDISTEGKGVVAAVVELLHSQNVRY

>IbDExDH170

MKLLRQKRPNRVLRGSAVDNLHRGFHCRGRWCEQVSEDHVAAREIDEAGRNFERDGKGCERRVRTRELNQSQRAAFDTIIAWVYSSSGGVFFVDGPGGTGKTFLYRCLLAKVRSNHDIALATATSGVAASLLPGGRTAHSRFKIPINGDDKFICNIGKQSAEAALLRECKLILWDEATMANRRNIENVETTLRDITNCDKIFGGKVVVFGVSQVPSLPPHKLILKKNCPVILLRNINLAEGLCNGTRLICDDFRDHVIRCVIASGNNSPVGQTINLTSIGPELTMEYRKKLYSNLGLFCPYGHEELAIYEDIDQCDLFIGRDLSFNMLNGERLDLEDNDPEYETEEYGGPDYDDRGIEYEEEVEDANEDLESEEDDDHVGEEHGQMVDEAEEDEHDEVVKERRKRKELEIFIGGLDKDATEDDLRKVLSEVGDVTEVRFMMNPQTMKNKGFAFLRFTTVEQASL

>IbDExDH180

MMISNGTANIWSPEELRESASGGAAAAAKELKSLNFPLKWSTWRCCGTCLRRRNPILRRMKNGLKGIEIVLDDIASTNLLMSRQEDSSLSFAPHDSRSGVLDGSLHSKERAVIFDPPLHSCSGSLTDLQPIQGHGQFCSDKDNAFYSSASNIQDDRLSHSHLIAENNLKAPVTSNMVESSWMEIETPQYNESHALFSSGQISDLLDEPWEQYSDMLLGDSLQFPSCSDFDFGILPCDGDNMLNVKDEEWGPSTWDAGSNNIVSREGLTRRTNEGVVDGYSNTRDINVDFQHACKSELHFTGYKNRETDCKVATQSWAYDPSYVFSRKQEVCAEDLKDDIIDRKPFSDADAALSNEPPNPFSPCLLPSNSGKSHLIYRKDENEGMSHESGGPRNHLYLVDNTTLENPFLVQNDCALNGLVGDKYGSLPSTSVKQGLDCVKTENIHSLRKTNDSCLLTTAHQSVQSSSLAQRTCAATGDDDVCILEDISASARPNPCPPNGKSLVNLQHATFSETSTRVGAGYMRFKPNGEQFVFRTALQDLSQPRSEDKPPDGVLAVPLLRHQRIALSWMVKKETTNPRCSGGILADDQGLGKTISTIALILKERSPSVKTFAAFTKQTEAETLNLDDDDDAPTKSNNGANSCQPNGCSATVAKPSAHAKGRAAAGTLIVCPTSVLRQWFDELHNKVANTANLSVLVYHGCNRTKDPFELAKYDVVLTTYSIVSMEVPKQPLVDDEDETQKHTTDSLNGVSSSRKRKSPLNSGKKSLKGKKGTEGELLDTDVRPLARVAWFRVVLDEAQSIKNHKTQVARACWGLRAKRRWCLSGTPIQNAIDDLYSYFRFLRFEPYSSYKLFCSSLKVPIQKHPTSGYRKLQTVLKTVMLRRTKGSQIDGEPILTLPPKRIELKKVEFTQEERGFYCRLEAESRAQFAKYAAAGTVKQNYVNILLMLLRLRQACDHPYLVGGSNSSLDWRSSIEMAKKLPQEKRILLFNCLEASLAICSICSDAPEDAVVTVCGHVFCNQCICEHLTGDDTHCPATDCKTQLSFSSLFSKKILTDSASDQPSLLNNPDYTGSESGETSQPYSFGCSYGSSKIKAALEVLQSLSKSRDCPPMVSSPGCKDEGNCYSENTSDSGSGVSHVKMNSGLDGNSNHSTKIPGEKALVFSQWTGMLDLLEDCLKKSSIQYRRLDGTMSVAARDKAVKDFKTIPEVSVMIMSLKAASLGLNMVAACHVILLDLWWNPTTEDQAIDRAHRIGQTRPVTVLRLTVKDTVEDRILALQQKKREMVASAFGEDENGSRQTRLTVEDLEYLFRH

>IbDExDH179

MAFLSAFLRCILLVSLSLSSSSSQFADGCYTSIFAFGDSLTDTGNYLSLCLKGLQTYDRFPFIGLPPYGETFFDRPTGRCSDGRIVLDFIAEHYGLPHVQPYFGGEKLNREAGVNFAVAGVPALDVEFHQERGIEFRNNISMRTQLKWFKDLLPSLCKNSSCKETFKKSLVVFGAFGGDDYGNTMFQKSIEDAQSIQPAIVDAITSAIEELIELGVVNLMVPGMLADGCLAITLSFFYTSYNVQDYDPVTGCLTWLNKFDENHNNLLQTALVGIQERHPHVSIVYADYYNANLQLYRSSEKFGFIKRALKACCGGGGPYNYNSSVPCGYAPSHACADPSSYIN

>IbDExDH178

MNIDLATTIHKMERALPSLENSSNARITDTYINTPNNGKGVQTQVTCISGVFKNATNSPLSILTNSGNLIKSSIICMNNTPEASHELKGLKKKQRPPLQDLTNGDPNELRFHGMVYPTQFNIAIPYNNDGTLSNSALTRVNGSITDVSQAIENGKSHVNHAVEDHLVVRNLAQEFAHVDNEHEQLPLPNEVSQFNNNGHPTCPCFEPNLGAYMDIGDPNNICEHYNAIYWFEERVNKAVRSGTAKYSTCCGHGKIKLPKMSVPPKRIFNLFFERGTKRNEFLKHIRRYNNMFSFTSLGANVDKSFNLGNCPPIFRIYGQNYHLMGGLLPELGKRPKFAQLYIHDTENEVENRINSLSCAEMKDKTHVDIVRDIKQDLDEHNVLVQSIRWAKTQIDNNPQAKMVIVKISVLFSPTEQAALGLRVSQREYFALRMPRGSGWRCCMPNDYINSFRLMPTPWGEVDTNKQGKRIILPSTFTGGARYMIQNYQDAMAICRHKGYPNLFITFTCNPKWPEIQRFMEKCNLNAEDRPDIVSRVFKMKLDALVKEIRNGKLFVVYTIEFHKRGLPHAHILIFLERLTGGFSVNHMDEFISAEIPNKEQDPEYYKDVSTLDDDGYPIYRRRDNGATVERNGVHLDNRYGVPHNRYLLLKYKAHINVEWCNQSRSIKYLFKYVNKGNDRVTAEFYKTTNDENENVAVDEITMYYVNSYLRLAATWRLLSFDVQQAPTVERLSFHRRCKQDCQYNRRLVLKDTGLRLSDDGKKNLGLLELELLLQVYNKCLKDYPQMPIPNYDDALLANNRLLFDELNYDRQAMKQESEEMESQLTDEQFVVYDTILSDIQNHGGGLFFVYGYGGTGKTFVWKALSSKIRSVGDIVLNVASSGIASLLLQAAEQLTQGLPFQLQDTRTPRATLVKLMTRRGNKNIEQFATWLAKIGNGTLGGRNDGHATIEIPTKMLLLSDGDYISKIVDSVFPTFRQGGSDFQQMENRAILAPTLDVVNAGVNESSISGQSSYRLELSRQRSTSPAASASSFHRCASLPRYELDANNITVVKSGDERHRSHRRRSFPLPPASAIELERLLLQLLKVIEPDGIKKAKCNHCHQQFTLTKSGTTSSLKRHQAKCVTRKTNLKMIDQQMKLNFLPSEGVSSSIPPLHPGKFDMELMRESATHWIMMHEHPFTILEEVGFNIMMKRDCWKSKNQKIEYMVVTGHWIDSSWRLQKRVLSFINIPPPRGGFQISDAIFKCMKEWGILCTS

>IbDExDH175

MENRNPIADVSDSIPPDHKLCGFLRAVLRIKVNPNDDLADALPLGSFCRIAGDAHYDVHFVTDNGVVLAPINSPDPDGSGDATAVPSTSKNKYTKKKKKEKKKKKSNSMEVIVDTPSTSKKNNKWSRIGMVNGSLSVVHQLHALVAHKCLSIIARVVCVAAENGEARAVLLVDVYLPVALWSGWRFPKSASAAAALFRHVSCDWKARGSILEYAKLGDEDYCSIWNLSDCHALGCKHHCSAPDPSKKKLFELHEIFKSLPSIVKKVDPDTSRINAADPSRPGIWLVADDILVNILSSLDPIDLVRVSATSHHLRHLTASIMPCMKLKLFPHQQAAVDWMLQRERDVRPFPHPLYMDFVTEDGFVFYLNVVSGEVVTDVAPKVNDFHGGMFCDEPGLGKTITLLSLILKTQGTLAEPPDGVEVIWCSHNGDQKCGYYELRSDNDAGVLPANRVMGQKARRGMFSPDKLMPKRSLQSSLPLRSTFVDSAKCVTDPEIKLLPVTFSTPPTCATRCTRSWSHVKRNLLLAYEESALSPEEKNPNKSFNKRKHVSNGQSENKTKKQHSQSHELSSTQKKLKKFNVDNLEHDETWVQCDACRRWRRVDDASVMDTSGAWFCSMNTDPLFQTCSAPEESWDSKQPVTYLPGFHTKGTPGGMEENVSFFINVLKDHYTFIDSVTKKALTWLAKLSAERLSEMEAAGLVYTVVDIGVPHPYNRIFQAFGLVKRVEKGFTRWLYPRALVNLVFDLDALRIALCRPLNSFRLYLSRATLVVVPSNLVDHWRTQIQKHVRPGQLRVFAWTDRRKPSVHNLAWDYDVVITTFNRLSAEWSPQKKSALMQVHWLRIVLDEGHTLGSSLSLTNKLQMAVSLKATNRWILTGTPTPNIPNSQLSHLQPMLKFLQEEAYGQNQKSWETGIIRPFEAEMEEGRSRLLQLLHRCMISARKKDLLAIPPCIKKVMFLNFTQDHARSYNELVETVRLNILMADWNDPSHVESLLNPKQWKFRSATIRNVRLSCCVAGHVCVTDASQDIQETMDILVGNGLDPSSEEYAFIKYNLQFGGNCMRCKVWCRLPVITPCKHLLCLDCVSLDSEKCTFPGCGNLYEMQSPEILTRPENPNPKWPVPQDLIELQPSYKQDDWNPDWQSTSSSKVTYLVHRLREIQEANRLLVQTIEDEGANSVNDIRLPFLRSNISMTLHGPESDICQVLPEKVIIFSQFLEHIHVIEQQLTIAGIEFASMYSPMHSANKIKSLATFQHDANCLALLMDGSAALGLDLSFVSRVYVMEPIWDKSMEEQVISRAHRMGAARPIFVETLAMSGTIEDQMLKFLQDPDEFRSLLKEVHDKQGRDGSRLHRTVHDFAENNYLARLSFVRTSCSET

>IbDExDH182

MGGLRLAATNHAVVRQFIVGLQLHPKQTNKAIRLRCVRSYVVSEMKRETTIRSQECVFHDQQGMFIHVHIPKDLVGKYNDIFVEGKVYGLRNFLCITNFFKYKTSMLRYMIKFKHDTLVKEYKRVNFPQTMFRFKSFEDILSKKEIDEKMLIDVIGRVVEIYSPVEKVIAGKLSRLIDFVLEDTSQRQVKCTLWDDHVDELSLYFNSAVADPLILLIQLCWAKIMDNGEVRICSSFDATLLFFNHSCKEFRELRNRYNTKLTPLRCIQSSSRLGVVARQRRRRKDLSRRNPKCLFHGRGRGCPAAQCRGCPVAVCGCAAVQQRRRREMEEDGGVEGVVDWYYVSWKDNYCKKKLIEKGGMMVCGGCKSSWHEGVGIKEFPSWDVYHLREMKMLLPDCSNLLGSNERDGIYGDGHCVSGFLFFDEEDCVAIEEVSRSGLEKVTDLEDFEEGFGVDGVDVTLKRSLLKDFDRCGSSKKSKGIVVNEEK

>IbDExDH168

MDSQVNNLYDTASQPDTGNDAYTFLEFNTPGRTLITRFQELSQPIRASAWPTPSDSLVSESPDRPRSSDASPSAKSRGGGEEEWRQWKWYCGVTNPACVVRCNVPSCRKWFCNSRGNTSGSHIVNHLVSLGRCAVKDVAKKSLSDCDVKVNSLNTKMNFNLKKGTGPSKAQRGFISAKTESVVVLLCREPCLNVNALKDMNWDLSQWCPLIDDRCFLQWLVKISAQQINKVEELWKTNPDATLEDLEKPGVDDEPQSVLLNYLTILFYNIYGRLTAKSTFIYYYHPVSLQYQNIFEPLIKLEADYDKMMKESQSKDNVTVSWDIGLNKREDNELRLVPGDELRLRYSGDAAHPAWQSVGHVIKLTALEEVALELRASQGAPADVCHGFSVDFVWKSTSFDRMQGAMKTFAVDETSVSGYIYHHLLGHEVEMQMVRNALPRRFGAPGLPELNASQVGSSNCLHLQICASKPISLIQGPPGTGKTVTSAAIVYHMAKQGQGQVLVCAPSNVAVDQLAEKISATGLKTYYSFTGCKALCQVREAVNSPVEHLTLHYQVRHLDTSEKSELHKLQLLKDEQDVICCTCVGAGDPRVANFRFRQVLIDESTQSTEPECLIPLVLGAKQVVLVGDHCQLGPVIMCKKAARAGLAQSLLALCYLVSSQLGCRYNITPFSKCIEFQVQYRMHPALSEFPSNNFYEGTLQNGVTVNERQSSGIDFPWPVPNRPMFFYVQMGQEEISASGTSYLNRTEAANVEKIVTTFLKSGVVPNQIGVITPYEGQRAYIVNYMSRNGSLRQQLYKEIEVASVDSFQGRKKISSFYLVLGVMNIRLGIGFLNDPRRLNVALTRARYGIVILGNPKVLSRQPLWNSLLTHFKVYNDRRVFFSGGPGNPSDTFGSPASSSSNGDKRNSRSRVLNHHKVWHSLKAKDKQGNKPKTCLDSRLNNIALMNKSRAPVPPYHGGPMQPYAIPTRGAVHGPVGTVPHVPQSGSRGFGAGRGNSNTPIGSHLPHQQGSQQPIGSLGSNFNFPALDNPNIQPSAAGPLTQPGYVSNMAVQGPSQTFRDGFSMGGMSQDFLGDDFKSQGSHVPYSASDFSTQASQSGFGVDYATQGPHSGLPGSFVNQNAQTGYSHFNSASEYMSQDYMAHGTQGLFTQAGYNEPSQDDASQKHFGMPNSTPLQSQFHLFTEFAKSTLLPAIHSLQHTYKHAKFSASTTATTLSGSRFTEPEPSLPWLKEQNLVDLMGYSFVSSNSVCVVKCDGVTCPIRCGAVFANNSGHGNTAAHPVLNRRKTNPYYLLQVLY

>IbDExDH181

MGSVKRKSIEDPSDTSRPEKQQREDSLLGLDEQVACVHDVSYPEGYVPHAPSSAKTEESLKPAKEFPFTLDPFQSEAIKCLNNGESVMVSAHTSAGKTVVALYAIAMSLKNNQRVIYTSPIKALSNQKYREFKEEFSDVGLMTGDVTIEPNASCLVMTTEIWRSMQYKGSEVTREVAWIIFDEVHYMRDRERGVVWEESIVMAPKNSRFVFLSATVPNAKEFADWVAKVHQQPCHIVYTDYRPTPLQHYMFPSGGDGLYLVVDEKGKFREESFQKALNALVPASEGARKGKMENGRRVWLLVKRMAKMDLNNDDEKVNIESIFWSAMDMLSDDDRKLPQVSNMLPLLKRGIGVHHSGLLPILKEVIEILFQEGFIKCLFATETFSIGLNMPARTVVFSNVRKFDGDKFRWISSGEYIQMSGRAGRRGIDDRGICILMVDEKLEPSTAKLMLKGSADPLNSAFHLSYNMLLNQIRCEDGDPENLLRNSFYQFQADRAIPDLQKQAKILAEERDSILLEEEDSLEDYYSLLQQHKSLKKDVRDIVFSPKYCLPFLQPGRLAGVYCTNTNENAPSFSIKENVTWGVIINFEVVKGLSKDDEDKKPEDANYTVDILTRCMVHKDELGKKSTKIVPLKDPGEAAVVSVPLSKIESLSSVRLKIPKDLIPLEARAHTLKKVSEVLSRYAKEGGMPLLHPEDDMKVKNSSYSKAARRIEALESQFEKHDISKSPIIEEKLKVLHKKKELTARIKSIKKALRSSSVLAFKDELKARKRVLRRLGYISRDDVVELKGKVACEISSADELTLTELMFNGVFKEIKVEEMVSLLSCFVWQEKLQDAQKPRDELELLFTQLQDTARKVAKVQLECKVQIDVENFVSSFRPDIMEAIYAWAKGSKFYEIMEFTQVFEGSLIRAIRRLEEVLQQLIQAAQSIGETELESKFEDAVSKIKRDIVFAASLYL

>IbDExDH174

MLDQNNVLVKCFRMAKSEIQSNPVVEVKMNLIGRGSKDGRTYNLPSANEVAALIVGDLDPSMGDLDILIESRTGQLKRINQLNPACLPLHPLLFPYGEDGYREDISFSDAWRQGHHGGRKRISPKEQIGYPHLFITFTCNPKWSEIEWYVAHRGLKSEDRPDIICRVFKMKLDAMIEDIKTQKLFGDICGAEIDAIISTEIPDPDADTKYHDVVGEFMLHGPCGQLRKNSPFMINEHQQNVVYGEDQTLDEIVENQTVKQSQFTAWFDANKKYEDACSLTYAEFPNDDKKYIDRITDSSYRASASTLRRLFATLLCSSTISRPEVVWDAIWEFLPEDAQFHHRRRMNNPAEELAYDKESLKIEDETLVTQLTDEQKNVYDSVMNDIDCNGGGLFFVYGYGGTGKTFVWRTLSSMIRSRGDIVLNVASSGIASLLLSGGRTAHSRFAIPLSLNEDSTCNISQGSDLAELIIRSKLIIWDEAPMTHKHCFEALDKTMRDLLRFAIPSSAEKTFGGKTVVLGGDFRQILPVIPKAMRPIVIGATINSSYLWTNCKVLRLTKNLRLRSLASEEDRQTVDWFSKWIANIGDEITGVVNNALIVTRLTDHIVQAKIVNGTHQGTKVLIARMSLTPSDTRLPFKFQRKQFPLMLAYAMTINKSQGQTLTHVRLLLKESSLITGSENPPVKFQQNFKRRSPVTKKATARSPSPKEGDHCGPLLKSEKVTSVVAFSNLRRRLQWSCAFSNLRRRPQWLPFAGEGDHCGFFLDEKDANGR

>IbDExDH172

MSSGGRGGAGLNPNANPDFKPRNPHQYVQRSPVPNQQHFQNQQTQQWLRRNNELGADFTADEVEKTVQSEGIDASSQNWKAQLKIPPPDTRYRTEDVTATKGNEFEDYFLKRELLMGIYEKGFEKPSPIQEESIPIALTGSDILARAKNGTGKTAAFCIPAIEKIDLDNNSIQVIILVPTRELALQTSQVCKELGKHLKIQVMASTGGTSLKDDIMRLYQPVHLLVGTPGRILDLTKKGVCILKDCSMLVMDELHSPVTVKDFKDRFLHKPYVINLMDELTLKGITQYYAFVEERQKVHCLNTLFSKLQINQSIIFCNSVNRVELLAKKITELGYSCFYIHAKMLQDHRNRVFHDFRNGACRNLVCTDLFTRGIDIQAVNVVINFDFPKNAETYLHRVGRSGRYGHLGLAVNLITFEDRFNLYKIEQELGTEIKQIPPHIDQAIYCQ

>IbDExDH167

MNGRKIYTRRQWDNYYREWNEEKKRKLSQSCETSGFSGNGSSEHGCCEKGVDGNGKSSEFAEGIDVPGDGSTSTMLGKIVGEKSRLSGSSSNGDNFSFKEGLNRNKKKRRKGGSEMNDPGDYTELLIDSTDPHLDSSGTAVDKNMKGKEKEQRVSGSLLDKKNAYFDTERWRKNKKGKVKEEIVTDAVNVSSEDEDEDEDETLTVGEEEIETDADNVNSEDEDEDGDETLTVGEEGIETDDDNVSSEGEAEDEDETFTVGEEEILTDDDNVSSEGKDEDEDETLTAGEEEFTLQSILSEKTDNRVEESSKKVEEKAVLVDNFSTDSGESRGSPLSEKHSDYDEDYLEFLEEYLAVPPSCKEKSENGVDTNNEAVDDIKCRSDVGDASEKKDNDDCSESFALSSDSTYKLEKWAEKKKKMNKKGKEKGEIGDHGSRSVSKCEMKEKDPVNVRNPLPASKENWGSEPTSDEDKKDDCRMRNHVNKDKKNVEHPSKRTKLKHGLGELNFKKILLNSISKNVDIPKNNLQFCNENIPGQLPLKFRFEDEDPTPPEKMEWEKEIDSLFVDLQTGLQEVKDSLTTTQPSVNEDNVSAEKHDDSAVCCEKGNHLLILDEQIGLICKYCSFVRLEMKYILPEFYGTFFLGRLKEQCILINARGMHNLDAPGTGKTRLTIVFLQSFMKLFPDSHPVIIAPRSMLLTWEDEFRKWDVDVPFHNVNNPVLSGRENSISHTFRNEFKSTESKRMLKVYSWAEGSGILGITYRLFEQYVRVKENKEDDMLRKILLRVPGLVVLDEGHTPRNDDSLIWKALSKLETPRRIILSGTPFQNNFDELYNTLCLVSPKLSNFSSGSQLKKMRRTKAAKKKWDRLTSSIGKNKNDDIKELKRIIAPLVHVHNGSVLQERLPGIRSTLEEMEKLELNPDAGVKTEFLFALIRLSVSHGEKVIVFSQYIAPMELIKKQLKSVFDWVEGREVLHMDGEIDGLFEGINLVGLQELFCLDVVWNPSVKRQAISRAYRLGQKKIVHVYHLISSTMEARKYACQVKKDFISELVFSTSDGLLCKSGLPVSEDKILEAMILHPKHRHIFDRLGHDPKESDLMDTFDFVE

>IbDExDH173

MQSGGGAQQGGGAAAGHGRGTAASASASPSSSSSASHLDQQQQQQQQQQRQAGSVHGFLGVNNFAAPSSSMQLSQQSRKYIDLGQQHNSPNLRNEGQNRSQGFEQQMFNPIQQAYLQYAFQNVQQKSAIGLQSQQQMKMGIFGNTGKDQDTKTPNTKVQDLVSLQVTSQSQASSSKMPPEHFTHEKQTDHGHQSMSDQRTELKPPSQPTLLGQVAMKHMQAPQGQQNIQSMTNNPLMVAQIQAIQALAFERNIDLSNPANAPMMAQLIPLMHSRMIQQQKASESNMPLQSPSVHVPKQQVNSPRVANDSSPHANSSSEASGQSSSAKNKQTVTSGPLGLAQSAASVNHSNNIPVQQFSVHGRENQLPPRQPMMGGNGLTPHLSQTTVSSMPGMESTLMAKAASTAVETMQIQQIKQVNRPLMQSATSSQDGSSGNPPPSQSGTLPQLQQPHLGFSKQQLHVLKAQILAFRRLKKGDGTLPRELLQAIVPPALDLQMPQVLPPNAPSNQDRSATRGTEDPVRRLEHTEKGTQLMIPAEGLNSSKEETSVEGQASAATVNLQTKTNVAKESTSVFPAAKEEQQTTGYSAKKPAQTSPVTQPKDAGAVRKYHGPLFDFPVFTRKHDAFGSSMMVPNNNNLILGYEIKDLIAEEGTEMFKRKREDNIKKIGEILSLNAERKRIRPDLVLRLQIEEKKLQLADVQARIRDEIEQQQQEIMAMPDRPYRKFVRLCERQRQDLARQVQASQKAIREKQLKSIFQWRKKLLEAHWAIRDARTARNRGVAKYHERMLREFSKRKDDDRNKRMEALKNNDVDRYREMLLEQQSGMPGDSGERYAVLSSFLSQTEEYLHKLGSKITATKNQQEVEEAANAAAAAAKAQGLSEEEVRAAASCAREEVMIRNRFSEMNAPKDGSSANKYYTLAHAVNERVLRQPSMLRAGTLRDYQLVGLQWMLSLYNNKLNGILADEMGLGKTVQVMALIAYLMEFKGNYGPHLIIVPNAVLVNWKSELHTWLPSVSCIFYVGGKDQRAKLFSQEVCAMKFNVLVTTYEFIMYDRSKLSKVDWKYIIIDEAQRMKDRESVLARDLDRYRCQRRLLLTGTPLQNDLKELWSLLNLLLPEVFDNRKAFHDWFSKPFQKEGPAHNVEDDWLETEKKVIIIHRLHQILEPFMLRRRVEDVEGSLPPKVSVVLKCRMSAFQSAVYDWIKSTGTIRVDPEDEKRRAQKNPIYQPKTYKTLNNRCMELRKTCNHPLLNYPYLNVSKDFLVNSCGKLWVLDRVLIKLQRTGHRVLLFSTMTKLLDILEEYLQWRRLVYRRIDGTTSLEDRESAIVDFNSPDTDCFIFLLSIRAAGRGLNLQTADTVIIYDPDPNPKNEEQAVARAHRIGQTREVKVLYMEAVVDKISSHQKEDEFRSGGAVDSDDDLAGTDRYIGSIESLIRNNIQQYKIDMADEVINAGRFDQRTTHEERRLTLETLLHDEERYQETVHDVPSLQEVNRMIARSEEEVELFDQMDEEFDWAEEMTRYDQVPKWLRASSKEVNAAVAHLAKKPSKNILLSGGIVAESSEMASESERRRGRPKAKKFPVYTEIDDENGEFSEASSEERNGYSVHEEGELGDEDDEFNGTIGAPQVNKDHSEDDRVSADGYEYHQAPGNTRNNHIPEQTGSSGSSAESQRLKQKVSPSVSSQKFGSLSALDARPTSRAKRVADELEEGEIAVSGDSHIDLQQSGSWIQGRDEGEEEQVLQQPKIKRKRSIRIRPKHAAEKQEEKSSEKVPVLSGKSSQLPLQVDRKYDLQIRNGRGPKIHGDPSAIKDDQTDSSFKGRRNLPSRKNSHLVKVQGLVKSGRSAPPEEAAEQMRQNWESKVMNTTGASGGGVKMSKAIQKCKNVVINLQRRINKEGHQIIPLLTDLWRRIEGDMDMADDNLLDLQTIGLRVDENEYSGVLEFVSDVQLMLKSAVQYYGFSPAVRTEARKVNDLFFDILKIAFPETDFREARNSVSFSGPATMTASGSSTRQMLGQNKRNRLVSDAEADAGPTHKPQTRGSLHATEDSKSSRSHVSQKEARLGVGSSREQDDPRPLTHPGELVICKKKRKDREKSVMKTGNMSGGPVSPTGVSRNAKNSGCVSMMKDSWLNQQGVQQQGQGWANQSPQLTSGGSSGGGSSVGWANPVKRMRSDAGRRRPSHL

>IbDExDH177

MVTEARCCYLPRRWSSLVRKMSLLLTLVLEDGRRRCAKQLQLQSLYSSGTDLSHVQSSQLPRWPSRRLAAAAAVVVEEGEVAAVAEAEVGSSAGGTPPGELSVFVRKLPRYMEVMNENEWWGKIEQFKRGGEQEMIIKRNFSRDDQGKLSDMAYQLGLHFHAYNKGKALVVSKVQLPNYRADLDERHGSSQEIRMSTEIERKVGNLLSSSQASVSDGNSSSTHSYTANVSSNAVHDARGATTLENDAAKERLNTELKQKQEKLRATDNVKAMMSFREKLPAFKVKSEFLKAVALNQVLVVSGETGCGKTTQLPQFILEEEIASSRGANCNIICTQPRRISAISVAARISSERGENLGETVGYQIRLESKRSAETRLLFCTTGVLLDQLVRDPCITGITHLLVDEIHERGMDEDFLLIILRDLLPRRPDLRLILMSATINAELFSKYFGNAPTIHIPDVDIDSHYKGYSMTTRQSLETWSGSQLDLGLVEATIQHICCHEGDGAILVFLTGWDDISKLLDKIKVNNFLGDPRKFLVLPLHGSMPTVNQREIFDRPPPNVRKIVLATNIAESSITIDDVVYVIDCGKAKETSYDALNKLACLLPSWISKASAHQRRGRAGRVQPGVCYRLYPKLLHDAMLEYQLPEILRTPLQELCLHIKSLQLGAISSFLNKALQPPDPLSVQNAIELLQTIGALDDAEELTPLGRHLCTLPLDPNIGKMLLMGSFFRCLNPALTIAAALAHRDPFVLPINRKEEADAAKRSFAGDSCSDHIALLKAYEGWKDAKRQGQERAFCWENFLSPITMQMMADMRMQFLDLLSDIGFIDKSKGSQAYNEYSNDLEMVCAILCAGLYPNVVQCKRRGKRTAFYSKDVGKVDIHPASVNASVHLFPLPFLVYSEKVKTSSIFIRDSTNISDYTLLMFGGNLVPSKSGDGIEMLGGYLHFSASKSVLELIKKLRGELDKLLKRKIEEPGLDISTEGKGVVAAVVELLHSQNVRY

>IbDExDH189

MDISEMPIPGESSMGLMENMLIAEELAYDKELLKTEHEKLGTQMTDEQKNVYNSIMNDIDSNGGRLFFVYGYEGTGKTFVWRTLSSKITNRSDIVLNVASSGITSLLLPGDMTTHSRFSIPLSFNEDSTTRVVYNGLSDIDIPAQFLLKCGPNHIATIVESTFPSARYGMLDESHLEGQAILSSTLGVVDQINQYMCGMNTVEG

>IbDExDH188

MDVICQAKSGMGKTAVFVLSTLQQIEPVAGQVAALVLCHTRELAYQASICSFNFIVFLSTYLPDIKVAVFYGGVNIKIHKDLLKNECPHIVVGTPGRILALARENSLSLRNVRHFILDECDKMLESLDMRRDVQEIFKMTPHDKQVMMFSATLSKEIRPVCKKFMQDVSARRSPTVGLDLVLGMLQSIISCSLHLLHEAPNMLYYDVYSFQPMEIYVDDEAKLTLHGLVQHYIKLTEMEKNRKLNDLLDALDFNQVVIFVKSVNRAAELNKLLVECNFPSICIHSGMSQEERLTRYKGFKEGHKRILVATDLVGRGIDIERVNIVINYDMPDSADTYLHRVGRAGRFGTKGLAITFVSSSSDSDVLNQVQERFEVDIKELPEQIDTSTYSTAIVMKRAQSPSRIASD

>IbDExDH191

MYPSVQKRSPRGPFRTTWEAGTQLLWKSRRGALPKDTSVAVCTIEKANSLVNRLLEEGRLSELGIIVIDELHMVGDRSRGYLLELLLTKLRYAAGEGNTESSSGESSVADWLDAALYQTDFRPVPLEEYIKVGNTIYNRKMEIVRTMSKAAELGGKDPDHIVELCNEVVQDGHSANFCSSRKGCESTARHVAKYLKKFSISPHNNQTELFDIDSAIDALRRSPAGLDPILEETLPAGVAYHHAGLMVEERETVESCYRKGLVRVLTATSTLAAGVNLPARRVIFRQPRIGCDFIDGTRYRQMAGRAGRTGIDTKGESVLICKPEEIKRILRLLNDSCPPLHSCLSDDKNGMIHAILEVVAGGIVQTANDIHRYVRCTLLNSTRPFEDVVKSAQDSLRWLCHRKFLEWSDDTKLYTTTPLGRASFGSCLSPEESLVNVSHCYDFMLFEEFLLLSLGLLTLPAAVVLDDLLRAREGFVLASDLHLVYLVTPINLEVEPDWDLYYSRFMELPTLDQSVGNRVGVQEPFLMRMAHGAAPVRSFNRSREGNKGLQEVPVPEVCNAFKVTRGMVQGLQESAGRFASMVAVFCERLGWQDLEGLVAKFQNRVSFGVRAEIVELTTIPYVKGSRARALYKSGLRTPQAIAEASLPEIVKALFESSSWSEQGSVQRRMQLGVAKKIKNGARKIVLEKAEEARIAAFSAFKSLGLDVAPLSRPLLSTAAGNADRKQSSTSSDEKSTSSFVHPEHINHVVSTSAKEGNEKINRVISAAGEENSKGRFSIQYNIAYGEAGEPVSAAEGYRSINSENETSTTLPLQFNSNEEKAYGEDIGHMVQKLHDRDGTSTRNDKNALESGPVNAVRSPGGFDTFLDLWDAAGEFYFDIHFNKRSELSTNVPFEIHGIAICWQNSPVYYVNFPKDLFWSNSKKNLLLSNVFGDNDALSPKHQWEMALQRWDRIRTIMGKNNVKKFTWNLKKQIQVLECPAVSILRFGSVNAMAKAEGLNLNEGYYILSPVHLQDAFDLCIVAWILWPDEEKGSSLSLEKEVKKRLSSEAAASANQNGRWKNQMRLLKPLTAVEIPLVDILSDMELWGIGVDMEGCLRAREIVRRKLKCLEREAYMLAGMNFSLNTPADIANVLYNHLKLPVPNSDDKGKQHPSTDKHWKAGNGSFVFLSSQVEHCFIIWGRCMNPCSFSALDEHPIIPVIKEHRTLAKLLNCTLGSICSLARLSMRTQRYTLHGHWLQTSTGTGRLSMEEPNLQCVEHMIDFKMNNNDKADGESDADHYKINARGLLCAYSADYSQIELRLMAHFSKDSSLIELLSNPHGDVFTMIAARWTGKSESIVSSKERDQTKSSDEAIEKFKASKALFPVLLPGYKKQFLSVVRKGFYGLLLILWSSYVETLNGRKRFLSKIKFGNSKEKSKAQRQAVNSICQGSAADIIKIAMISVHSAIVKDIPKSRSNSALPENFDMLKGRCRIVLQVHDELVLEADHSVVKEAGMLLIAWKMQHHFLLAKLRPPRLNLEFKLPILVFNWIVKVDSIGAE

>IbDExDH184

MSHSQDFKRFKYNGNSSSQEPPKSLWSLLDIRNFGRVMEMTMDIHSLFSSQPIPSGHGKKRCSRYTCLQPQFFIQVVCDVWLGFDFGIPGSFQHLVLPENIPLPTVLVDVQPLPVAALRNPAYEAFYQEFKHFNPVQTKVFNVLYNSDDNVLVAAPTGSGKTICSEFSILRDHQKGLSDSVMRVVYIAPREGLAKERYSDWKKKFGDGLGVRVVELTGETTTDLKLLEEGQVVISTPEKWDALSRRWKQWKHVQQVSLFIVDELHRIGDQGGHVLEVIVARMRYIASQLEKKIRIVALSTSVIQDADFANSEALLDKEKHIYTSIYTAIVQHVSSGKPAIVYVPSRKRVNLAVFRLKTYLRSQCANRVELPFVESIEEHELKEALKYGVCYLHEGLAKTDQDIVKTVFKNGLAQVCVISSSMCCGLPFSANLVMVMGTQHCDGRENGATGKDYCRNFMFEALFPVESQLQHYLHDHLNAEVVSEVIQNRLDAVGYLGRSFMYRRLTQNPNYYNLQGVVDDRSWLDHFSVIVRKTSSDLVARRCVTCFADYLLSPNNLGQLQSTNSFLFGPGEEELIRSLVNDHRYSFENPKYSDPCVKANALLQAHFCRQMVGEENLAADQQQVILYASRLLGAMVDVISCNGWLSLALLAMEVSQMVTQAMWECDSMLLQLPHLTKELAKKCQENPGKSIETVSDLLEMEDGERRELLQMPDTQLMDIARCSDRFPSIDVSYQVVDGENVRASGEDINLKVTVGRTTEVAPVLAPRYPVKKYEGWWVVVGDPKSNKLLAIQRVMMSVQRKSMVILNFAAPAKAGKKTYTLHFKCDSYVGFDKEYVFTVDAKV

>IbDExDH187

MQGLLKVVVVSGETGCGKTTQLPQYILESEIEAARGATCSIICTQPRRISAMAVSERVAAERGENLGESVGYKVRLEGMKGRDTRLLFCTTGILLRRLLVDRNLEGVTHVIVDEIHERGMNEDFLLIVLKDLLHRRPELRLILMSATLNAELFSSYFGGAPRIHIPGFTYPVRSHFLENILEMTGYRLTPYNQIDNYGQDKMWKMQKQTLRKRKTQIASAVEDALETASFREYSPRTRESLSCWNPDSIGFNLIEHVLCHICRRERPGAVLVFMTGWDDINALKDQLQANPLLGDSSRVLLLACHGSMASTEQRLIFDKPEDGVRKIVLATNMAETSITINDVVFVLDCGKAKETSYDALNNTPCLLPSWISKASARQRKGRAGRVQPGECYHLYPRCVHDTFAEYQMPELLRTPLQSLCLQIKSLQLGSISEFLSKALQPPESLSVQNAIEYLKMIGAFDEDENLTLLGLNLSMLPVEPKLGKMLILGSIFNCIDPIMTIVAGLSLRDPFLMPFDKKDLAESAKAQFSARDFSDHLTLFRAYEGWKDAERNQSGYEYCWKNFLSAQTLKAIDSLRKQFFHLLKDSGLVDSGESCNIWSHDEHLVRAIVCAGLYPGICSVVNKEKSISLKTMEDGPVLLYSNSVNGLEPRIPYPWLVFNEKVKVNAVFLRDSTAVSDSMVILFGGNITKGSANGHLKMLGGYLEFFMNPTLATTYVRLKRELYELIHEKLLNPKLNLGDHDELISAVRMLVSEDQCEGRFVFGRQIPSSAIKSRKDAEAGMVSSKGNGGDNPKSHLQTLLVRAGHQSPSYKTTQLKHNKFRAVVMFNGLDFVGQPCGSKKEAEKAAAGEALQWLTGETQSSQKTVEHMSAILKKSKKKQLHASRWRCDFLPASVRGWEGSWRHPHLLVPKLTKSNSAAPSIFIYIESHRSGVVTTTPSGCG

>IbDExDH186

MAPVDPHSFTDSDHPLTTHVSLTLYFDFPSSTISAATRLTLAAPFSGELTLDTRSLSISAVLDPLSLSPLPYSLSPSPPDPVLGQSLTVTVSDKAEVLILSKTSADSSALQWLSPPQTFNKTHPFVYTQCQAIHARSIFPCQDTPAARTKYSAKLNVPRKLSAVMSAKHVDRRPPVAAEAGGACEDSIWCAEDRIVEEFVMDQPIPPYLFAFAVGELGFREVGPRTRVYSEATPAVLDAAAEEFAGTEEIIRVGEKLFGAYEWERFDLLVLPPSFPYGGMENPRMVFLTPTVIKGDATGAQVVAHELAHSWTGNLITNKNNDHFWLNEGFTTYAERRIVEVVQGEERAAMNIGIGWRGLVEDVERFKDNLEFTKLKTNQAGVDPDDVYSSVPYEKGFQFLWRIERQVGRPAFDEFLKKYIATFKFQSIDTDMFLDFLKANIPGIGDEVDLKLWTEGTGIPPDAMEPVSDLYSKIVSLAEEFKLGRMPREDEVADWHGQEWELYLENLPKSVEASQVRALDARYRLSESKNYEVRVGFLKLGISAGCREYYSEVEKTLKEVGRMLYLRPLYTALVQGDGKEEDKVFARRVFSEACDCYHPIAKGVVEAIFAKFG

>IbDExDH193

MQKEEFQHTAYATNAYVKVGPLAKQLLQGKKVIKLEVSNKQVGSSNFIKSSKRSRSSGLESMLDELRKELAAIHGSMFPHSVLSTQLMCTISMQKPDSVEELEKIIGKLKVEKYGSRILQEIKSYESKPEALESVDGEQGASKKLRSGKKALVVIESREEEL

>IbDExDH183

MSYSQDFKRFKYNGDSSSQEPPNSLWTLIDMENFGGFKKPTMDIHSLFSSEPIHSGQGKKRYKHQLSQYFIQVVSDVWLGFGIGFPGSFRHLVLPENIPPPTVLVDVQPLPVAALRNPVYEAFYQEFKHFNPVQTKVFNVLYNSDDNVLVAAPTGSGKTICSEFSILRDHQKGLSDSVMRAVYIAPREGLAKERYSDWKKKFGDGLGVRVVELTGETATDLKLLEEGQVVISTPEKWDALSRRWKQWKHVQQVSLFIVDELHLIGDQGGHVLEVIIARMRYIASQLEKKIRIVALSTSVVNARDLGEWIGATSNGLFSFPIGPKLVPLRKLIFRGADIPISENLQNMHKHFYTAIVQHVSSGKPAIVYVSSTEHVKEAAFYLETYLRRDKLLLALVELPFVESIEEYELKEALKYGVCYLHEGLTKTDQDIVKMVFKNGLAQVCIISSSMCCGFPFSANLVVVMGTQHCDGRENGATGKDYCRNFMFEALFPVESQLQHYLHDHLNAEVVSEVIQNRLDAVGYLGRSFMYRRLIQNPNYYNLQGGVDYTSLLNHLHVTVKKTSSDLGASRCVTFDSDFLLSPNNLGVIASNYYINYTTVDRFSSSLTPTTKLKGLLEILTAASEYQQLPIRPGEEELIRSLVNEHRYSFENPNYSDPRVKANALLQAHFCRQMVGEENLAADQQQPCTPRYGSEPNGDPSHVGFDSMLLQLPHLTKELAKKCQENPGKSIETVSDLLEMGDGERRELLQMPDTQLMDIARCSDRFPNIDMSYQVVDNENVRASGEDISLKVTVGRTTEVGTSFGT

>IbDExDH192

MASDAPRARIDQFFASKKKRKFESPALKSPRVNKDAKISIEGSPGTKGSLESYLVNSKDNSTPLHEAGGSAVKRNLTLDLGLISKHGKEVAVSSRQAHSQGFETTGEGQKFEVLSNTGFAARELPKDSLESTGVQENLELKQFASDFLSLYCSEVPPSESLPLQQNVHPNKRHASPSTCDLDVMTSKGRRPFTADGSHSSVEDNAASSLISILKIPVIMWLLSLHMQLCVPVNVDRLAEPQISLRKCNNVPVAEATESLTPGLSATKHVAGTPSSGHRTSIFSPGETFWNEAIEVADGLFAGNKKVAYQVTKETEALNTRHEILSSNNLKNGGCGNKSNKLIEGIADKVSGVGMVPAIVPIRKIGKELDKEVSPLPVKHFDFALEDKIFSQSKNCQILSGQQAQHVSAANQYVHSTCNEAQVNGSSHEENVGTSKYAASKSKVEVFVQDNGSPMFNTPTERSGDIKAISESSQDDTPSSFALQKDRLDLSNWLPPEICNVYKKKGISKLYTWQSAYIAVRVFALIFTLGAFIPKVDCLHVDGVLENRNLVYCASTSAGKSFVAEILMLRRRILSKGKMAILVLPYVSICAEKADHLEVLLEPLGKQVRSYYGNQGGGALPKDTSVAVCTIEKANSLVNRLLEEGRLSELGIIVIDELHMVGDRSRGYLLELLLTKLRYAAGEGNTESSSGESSGMSSGKADPAHGLQIVGMSATLPNVAAVADWLDAALYQTDFRPVPLEEYIKVGNTIYNRKMEIVRTMSKAAELGGKDPDHIVELCNEVVQDGHSVLIFCSSRKGCESTARHVAKYLKKFSISPHNNQTELFDIDSAIDALRRSPAGLDPILEETLPAGVAYHHAGLMVEERETVESCYRKGLVGVLTATSTLAAGVNLPARRVIFRQPRIGCDFIDGTRYRQMAGRAGRTGIDTKGESVLICKPEEIKRILRLINDSCPPLHSCLSDDKNGMIHAILEVVAGGIVQTANDIHRYVVLDDLLRAREGFVLASDLHLVYLVTPINLEVEPDWDLYYSRFMELPTLDQSVGNRVGVQEPFLMRMAHGAAPVRSFNRSREGNKGLQVKLECKSGVSNHSVLSDEQVLRVSRRFFVALILTRLVQEVPVPEVCNAFKVTRGMVQGLQESAGRFASMVAVFCERLGWQDLEGLVAKFQNRVSFGVRAEIVELTTIPYVKGSRARALYKSGLRTPQAIAEASLPEIVKALFESSSWSEQGSVQRRMQLGVAKKIKNGARKIVLEKAEEARIAAFSAFKSLGLDVAPLSRPLLSTAAGNADRKQSSTSSDEKSTSSFVHPEHINHVVSTSANEGNEKINRVISAAGEENSKGRFSIQYNNAYGEAGEPVSAAEGYRSINSENETSTTLPLQFNSNEEKAYGEDIGHMVQKLHDRDGTSTRNDKNALETGPVNAVRSPGGFDTFLDLWDAAGEFYFDIHFNKRSELSTNVPFEIHGIAICWQNSPVYYVNFPKDLFWSNSKKNLLLSNVFGDNDALSPKHQWEMALQRWDRIRTIMGKNNVKKFTWNLKKQIQVLECPAVSILRFGSVNAVAKAEGLNLNEGYYILSPVHLQDAFDLCIVAWILWPDEEKGSSLSLEKEVKKRLSSEAAASANQNGRWKNQMRKAAHNGCCRRVAQTRALCLVLWKLLISEGLLKPLTAVEMPLVDILSDMELWGIGVDMEGCLRAREIVRRKLKCLEREAYMLAGMNFSLNTPADIANVLYNHLKLPVPNSDDKGKQHPSTDKHCLDLLRDEHPIIPVIKEHRTLAKLLNCTLGSICSLARLSMRTQRYTLHGHWLQTSTGTGRLSMEEPNLQCVEHMIDFKMNNNDKADGESDADHYKINARDFFVPTQENWLFLTADYSQIELRLMAHFSKDSSLIELLSNPHGDVFTMIAARWTGKSESIVSSKERDQTKRLVYGILYGMGSKSLAEQLGCSSDEAIEKIQSFKGSFPGVASWLQEAVSICREKG

>IbDExDH190

MTNAAWPSHPPIGVSRGMSEAASASAATTHYYCCVVHHSLGSITPPFRPALQHRYCYLNLFITFTCNPKWPEIQCYMGIRGLRAEDIPDIVARVFKMKLDALIKEFKSGKLFRPVKALIYTIEFQKRGLPHAHIVIFLASTAIVSSPEEMDSLISAEIPDKENDLEYHQAVEDFMIHGPCDGYPLYRRRDDGKTVKRNGIELDNRYVVPHNRYLLLRYKAPMNVEWCNQSRSIKYLFKYVNKVNDRVTAEFYQSATYADGVEVVDEINMYYDCSGELYYLRCLLNIVKGPTSYNEMRTYNGVEYQTFKEACYARGLLEYDNEYIDAIEEASQWSSAYSLRKLFVTLLMSNTMVSLESMWNKVWLLLADDAQHNRRRLFNQSDLFINDEENKEFALLELSKLLLVYNKTLRDFPNMPVINEANIVAVENRALSAFIRAKGHIVINVASSGIASLLLPGDGNIGVDNDGYAEFDIPSELLLHSNGDPISTIVQSTFPNFTGANIDGSCFKNSAILAPTLEVVNEVNQYMSNLTQGEGKTYLSSDTTCKVDGSSSVLAEVHTPEFLNTISASGLPSHSLTLKVGSPVMLMRNIDHTLGLCNGTRFSVRDFTAQRRLLCAHLILSATTGAIASQKRARKSYQLLGGAGSLVPLCLSSSLQKSIGSIEKYDYKSCDYRRYSTISKSKQESLPQHDLLSFIQSTINKHEGPSHCWLNGIALKKNFFKKEGITLVIVAEFFQGSSLSQHDLFIMLDKVKLLQQRYPFLPVMGYQYSTSPLLSKDDHTHLLRRVMKEYITFPILLSNKNFPKITGGTCCIIFRGLMSPIIYQGKEVGSMILDNASFYHAADNCLYIADSENHAIRRADMGRRILDTLYPAMNSNKDSNRLWSWIFGKLWSRKDIEAKSDEFPPKTLLFPWHISKCQNDLFVLNRNLQTLWILDLASGALQEIVEGFSNISEICGHLILEKSNILKQIPNDLLKQLMHTDCSLEGIPYAGLISSIATFQDDLIICNTVGQEVLKFNIKSATLSTFQFSNFSILGLPYWFSFPLERVCATKDVLSGLYVDHAELFNLLPGKVDIKLSIEIPKSFELVEPLIESCIWLQARGAATVVSEAERISTSEKVCAAQQWYDELDHRTFWESELESNKEVHSSTESSVEVLSSSPSEVVPEGKVLIDCSINSSPGTSEVIISAALYLKLRKTADTSMDSREQKAAKIADSLDPTRRDSYLKCAHSLTGIPRPTSNSSSPKLASCPCGVPYNVSRWRLPQVHGGEGWRKGVFRCAPFPSYGRGWRRGEFRSVFLDGIRFR

>IbDExDH17

MKSGRCNESERAENNLKRRNAYSAMDPDKKRKMLDERNEKRRAKMKEVQSLQIRPVNADCPPCTYCNARRFQYEPPGFCWSSGQVVLVSNQMPSLLKQLFSEKDEVSRQFQACVRTYNNTFAFTSLGIHNYDKILTRRNKGIYTFKVQGQMYHFINDLVPGDQPPKNLQLYFFDTDHEVENRIKGADRMEASVVENLIGVLGRNPYSQFFRNLKDMSSLDDCNIVIRSNASLDQRVYNMPTSSQVAAIWVDEQDGSGQNNRDIRVLGKTGQSHSVKYYYGCYDPLQYPLLFPHGESGWHEGISKVPKSSQNSSSICEGILQPRGFSSIQALLHRENENFVGQSKRDTVSCREYYAYRFQIRPNDQSMLLHSGRLFQQFIVDTYIKIETQRLDYFRTQQRDVRTESMQGLVDRIAYGETNASNVGRRVILPVSFIGGPRDMLSLYLFRDGPFIRTA

>IbDExDH51

MAAQASADLSHNEDDLLCAENAESEGENFRSSDLGRPHGYLSGEARIERAWAHWKKLGQPKCIVAPMVDNSELPFRMLCRKFGAQAAYTPMLHSRIFTENDKYRSLEFTTCKVGGLDAHKEPAIPHSKGQGSIFCPCPQSRPIIITLPPSTTRSLHGEDRPLFVQFCANDPDILLEAARRVEPYCDYVDINFGCPQRIARRGNYGAFLMDNLSLVRSLVEKLANNLSVPVSCKIRIFPDLQDTINYAKMLEEAGCALLAVHGRTRDEKDGKKFRANWDIIKAVKNSVRIPVLANGNIRHMDDVQSCLEETGVEGVLSAESLLENPALFAGYMTTEWMNGSIGIKEDGTVDQAELVVEYLKFCEKYPVPWRMIRSHVHKMLGEWFKLQPDVREDFNKQYKLTFEFLYGLVNQLRERGVRIPLYVKNPNNEGMSTNGIVA

>IbDExDH71

MKVASLIVRDLDPTMGDLDILVKSRTGQLKIINRLKPPDMLLQYPLLFPYGEDSYNEDIIFSNAWRQRHPGSKTRISPKEYFSFYIHERLSTQLNFKKRGLPHGQILIFVKRMNRANCADEIDALISTEIPDPNADVEYNVAVSEFMLLGPCGEIPKNSPSMVDVNKGNDRVTTEFMSSSTNARNGDVVDEINMYYDCCYISACEATWRLFGYVIHYRTPPVERLNFHLENQQNVVYGEDHLDLDDIVENQTINHDDIRTFGGVIHKSFKDACYEYESLDDDKEYIGDNADSSYWATAYAL

>IbDExDH185

MERSVTPDAILAILANPSPGSGSDLPELIVQVVDLKPAGNRYMFTANDGKMKLKAILQSSLSSEVISGAIQNLGLVRILDYTLNDIPMKNEKYLIVTKCDAVSSPLEAEYKTGVKTEENGIVLKPKQEYVAKSAAQIVHEQNGNIAPPARLAMTRRIHPLVSLNPYQGIWTIKVRVTSKGNMRSYKNARGEGCVFNVELADQDGTEIQATMFNQAARKFYDKFELGKVYYISKGTLRVANKQFKTVQNDYEMTLNENSEVEEAINEAAFVPETKFNFVAIDELGSYVNGRELVDVIGVVQNVSSTMSIRRKSNNEMIPKRDITIADETKKTVVVSLWSDLATNVGQELLDMADKSPVVAIKSLKVGDFQGLSLSAITRSTVLVNPDMPEAKKLRSWYDCEGKETSLTSVGSGLSSSAKGVSWSMYSDRVSLLHITSNPNLGEDKPVFFSVKANISFIKPDQAMWYRACKTCNKKVTEAIGSGYWCEGCQKNDTDCSLRYIVAMRVSDASGEAWLSVFNEQAEKIIGCSADELDRLKSQDGDSSAYQMKLKEATWVPYLFRVSVTPQEYNNEKRQRITVRAVAPVDFAAESRYLLEEMAKMNICV

>IbDExDH12

MRLQKSTSNNDYAQLKEFSEWIAKIGDGKIEGQVDECEYIDIPEHILLQYSADPIKAIVENMYPSFSSIIDDPSYLQKQAILATLGVVHSINEYMSSLNTSEGSTYLSCDSTCKSDANIGMLADVHTLEFLNGIKCSGVLNHALTLKVGTQVMLLRNIDHSIGLCNGTRMIIIKLGNHVLEGRVLSGAVAWRLKLPPSRSPCLGKTHRFPSQGIFRTAIFTMASSSAPFQIIDDDDDFDWEAAVREIDVACEATAAAAAVNVPFSSSSSSAGASTSNYAPIKQTHQKLNGAPARQSTLDKFMGFSSKVKNAEPAPHNSNGFGDINENDFDNDGSDGKGCCVPIDIEAAKTWIYPANLPCRDYQLSITRTALFSNTLVALPTGLGKTFIAAVVMYNYFRWFPEGKIVFAAPSRPLVMQQIEACHNIVDIPQEYTIDLTGQTNPTRRASLWKEKRVFFVTPQVLEKDILSGTCMVKHLVCLVIDEAHRATGNYSYCVVVRELMAVPVQLRILALSATPGSKQQAVQNIIDNLQISTLEYEMKVPMGKDAVEINNLLSDVIRPFAARLSALGVLQNRDYLAVLSSHGIKYAFEMANDKLQQGASGRLLSRNETLLKAKLLMQKTVDHGAPSPKLSKNAGNTKDILDELANIGPSVKATEFIGQSSDEDVMVNEWYSSNYFTTPFPSSVTNVDLSDLICHFRKWQSIKGTVTKVQQAVLEKFRAGVYNVIVATSIAEEGLDIMEVDLVICFDSNVSPLRMTQRMGRTGRKHEGRIPHVIRPEVQHVKLLIETFVPRGKKAKVAHPVQIPTLENKLSDNEIDLLAKYFNSSGESLSKPSLIAYRHFQAFPSRVHRVSHSFRTEMLIDAMQHLEGLAFSSYAKASSEVETPGNLCMRVEAAETYENGEEGKPIPKLTAFRSLYTIKKVPCSHIFMEYSYFQFLVSDLETFGCSPEEDCERELSENDKDPAKTTVEVINSVKEFTGKNSHTHLSLFDSELVTVDDLGNVLVSPLPKEDVCDRMDSNDLIHEKTMKFKGVFDDLGERMEENVQASRLCNMDEWQEEVFQSDRVLQTPVCKVKPKDSRTAEDPEAILDNTDIKKLSNDSEDVAALSPRLTNFILSGVVPESPLNSPDRENDEGQKLNSDDLMGSLQKSNQAVHYSTTIGENVLPSQINETFTPLQRRDKNGEREASRDSRSSLQNFYGNQTPFEKLSSPSCSKDWYLESQHKSERVGQKQFRRLRKLGEMKQVKDARAFIEVEAEVSSEGLVSDDEDEEDCNSYDDSFIDDRINLTAADTQADSSRMDMMAIYRRSLLTQSPMAQLPKAFSHQTPESMAPRCITNVTMSSSGPKYHPTPQAGLESSTRGSEATPCATTHSLEEKESKIENRKRKASPCETSPIPARNLENDFLLQPETGGGRSSPLRVQERKNEDMVLFDDDDDEFFRGIDLDAVEEEAARILRHKSQNQATSIPIPRNIDFFSDAPSFDLGI
