# Supplementary material for: Genome-wide systematic survey and analysis of the RNA helicase gene family and their response to abiotic stress in sweetpotato
Source: BMC Plant Biol. 2024 Mar 16;24:193. doi: 10.1186/s12870-024-04824-z (PMC10944623; doi:10.1186/s12870-024-04824-z)
Supplement: Supplementary file 1 — Supplementary Material 1. [file 12870_2024_4824_MOESM1_ESM.zip › Supplementary data/Supplementary Table.5.docx]

**Supplementary Table.5 Specific primer sequences used for qRT-PCR analysis**

| **Primer code** | **Primer sequences (5' →3')** |
| --- | --- |
| IbDExDH25-Q-F | ATCGAATAGAGGCCGCAAAGG |
| IbDExDH25-Q-R | GGGAATGCAGGAGGAAGAACG |
| IbDEAH32-Q-F | GTAGAATTGGACATGGCTGGC |
| IbDEAH32-Q-R | CAACTCTCACACCTTGGCACC |
| IbDExDH36-Q-F | AGCAGTAGAGTCGCTTCGCCC |
| IbDExDH36-Q-R | CGCTGTTCTCCCTTGCGTTTA |
| IbDEAH42-Q-F | GAGATTCTTTCACCCCAGTTAGGC |
| IbDEAH42-Q-R | TTCATCAACTCCAACCATTATTCC |
| IbDExDH47-Q-F | CGAAATCATTACCAGGACAGCAAG |
| IbDExDH47-Q-R | TGAGGCGTTGAGTTAGAAATGCAC |
| IbDExDH48-Q-F | TGTTGGAGAAAGTTCAGGGATTAT |
| IbDExDH48-Q-R | GACGAAGCAAACCTTAGTAGATGA |
| IbDEAH53-Q-F | TGATAATTTGATGTCACTTTTGGG |
| IbDEAH53-Q-R | AAAGCCTGTGTTTTGTGGTTAGAC |
| IbDExDH96-Q-F | CACGAGCCCAAGTGATAAACG |
| IbDExDH96-Q-R | CAAATACGCCAAACAAGCAAACT |
| IbICE1-Q-F | TGGAAGCTGAGGAAGTGGAGTG |
| IbICE1-Q-R | GAGGAAGGAGAGCAAGAGGAAG |
| IbICE2-Q-F | TTGGAAATCAAGAAAGCGAGG |
| IbICE2-Q-R | CATTGTTGTTGTTTTGTGCCC |
| IbMYB15-Q-F | AGCGATTGCGTGGTCTCC |
| IbMYB15-Q-R | CTCGCCGTCAACCGTAGC |
| IbCBF1-Q-F | CCAGCAAACCAAAACCAA |
| IbCBF1-Q-R | ATGTCAGCAAGCAATCCG |
| IbCBF2-Q-F | GCCGCCCTTCTACTCTCCAA |
| IbCBF2-Q-R | GAACACCGCCTCTTCATCCA |
| IbCBF3-Q-F | TTCCGTCCTAAAGAATGCGA |
| IbCBF3-Q-R | ATGCTTCCAAGAAACCCTCC |
| IbCOR-Q-F | AGAGGGAGAAGGGAAGAGTGT |
| IbCOR-Q-R | CCTTTATCGGTCCTAACATCG |
